# Genomic Expansion and Subgenome Expression Divergence of Sugar Transporters During Polyploid Evolution of Bamboos

**DOI:** 10.3390/biology15181614

**Published:** 2026-09-13

**Authors:** Binao Zhou, Mingzhen Lv, Weixin Yan, Xinyi Luo, Wenjing Yao, Shuyan Lin

**Affiliations:** 1 State Key Laboratory for Development and Utilization of Forest Food Resources, Nanjing Forestry University, Nanjing 210037, China; bnzhou@njfu.edu.cn (B.Z.); lmz011213@163.com (M.L.); yan030706@njfu.edu.cn (W.Y.); 18770030230@163.com (X.L.); yaowenjing@njfu.edu.cn (W.Y.); 2 Co-Innovation Center for Sustainable Forestry in Southern China, Nanjing Forestry University, Nanjing 210037, China; 3 Bamboo Research Institute, Nanjing Forestry University, Nanjing 210037, China; 4 College of Life Sciences, Nanjing Forestry University, Nanjing 210037, China; 5 College of Forestry and Grassland, Nanjing Forestry University, Nanjing 210037, China

**Keywords:** Bambusoideae, sugar transporters, whole genome duplication, subgenome expression, gene retention

## Abstract

Sugar transporters play a pivotal role in photoassimilate partitioning and biomass accumulation in plants. However, how whole-genome duplication (WGD) has reshaped the evolutionary trajectory of the sugar transport system in Bambusoideae remains elusive. Here, we systematically identified and characterized the SUT (Sucrose Transporters) and MST (Monosaccharide Transporters) gene families across 14 bamboo genomes representing four major lineages: herbaceous, temperate woody, neotropical woody and paleotropical woody bamboos. Our results suggest that WGD acted as the primary driving force behind the expansion of these gene families, with woody bamboos possessing significantly higher gene counts than herbaceous bamboos. Notably, extensive gene loss was observed in hexaploid lineages, aligning with the gene dosage balance hypothesis. Phylogenetic and sequence analyses demonstrated that core genes were subjected to purifying selection. Intriguingly, 21 groups of identical protein sequences across species were identified in paleotropical woody bamboos, suggesting high sequence conservation. Transcriptomic and syntenic analyses further unveiled the mechanisms of post-polyploidization expression divergence: homeologs exhibited marked expression asymmetry across subgenomes. Distinct from the broad expression pattern in herbaceous bamboos, which rely on SUT5 and a few STP (Sugar Transporter Protein) genes, woody bamboos have achieved fine-tuned expression partitioning and tissue-specific specialization during different culm developmental stages through significantly expanded SUT and STP family members. This study reveals an evolutionary pattern characterized by “WGD-driven expansion, post-polyploid fractionation, and subgenome expression divergence.” This pattern suggests a potential link to the enhanced sugar allocation efficiency required for the explosive growth of woody bamboos, offering valuable gene resources and a foundation for future functional studies in Poaceae crops.

## Simple Summary

Bamboos include both herbaceous and woody species. Woody bamboos can grow remarkably fast, sometimes more than one meter per day, which requires a very efficient system to transport sugars from leaves to growing stems. In this study, we compared the genes that encode sugar transport proteins in 14 bamboo species. We found that woody bamboos have more copies of these sugar transport genes than herbaceous bamboos. The extra copies are not simply redundant; many of them show tissue-specific expression and are active at different stages of stem growth. This more specialized expression pattern may help woody bamboos deliver sugars more effectively to rapidly growing parts, supporting their explosive growth. Our findings provide new insights into how bamboo growth is regulated at the genetic level and may offer useful gene resources for improving sugar transport and yield in crops.

## 1. Introduction

In plants, sucrose, fructose, and glucose serve dual roles as essential energy substrates and as signaling molecules that regulate vital life processes, including seed germination, vegetative growth, flowering, fruit ripening, and stress responses [1,2]. Among these, sucrose is the primary form for long-distance transport due to its chemical stability and low osmolarity, whereas glucose and fructose predominantly participate in local metabolism and signal transduction (e.g., via the SnRK1 [sucrose non-fermenting-1-related protein kinase 1] and TOR [target of rapamycin] pathways) [3,4]. During periods of rapid growth or environmental stress, the spatiotemporal allocation efficiency of soluble sugars dictates both biomass accumulation and adaptive resilience. Consequently, the directional transport from source organs to sink organs constitutes a critical physiological bottleneck affecting plant competitive ability.

Long-distance sugar transport occurs primarily via the phloem through two main pathways: the apoplastic pathway, which requires active transmembrane transport via transporter proteins, and the symplastic pathway, which involves diffusion through plasmodesmata [5,6]. In this process, MSTs and Sucrose SUTs located on the plasma and vacuolar membranes play core roles. They regulate sugar loading, unloading, and compartmentalization, directly influencing the carbon sink strength of sink organs and the overall growth potential of the plant [7,8,9]. MSTs belong to the Major Facilitator Superfamily (MFS) and comprise seven subfamilies—STP, TMT/TST (Tonoplast Monosaccharide Transporter/Tonoplast Sugar Transporter), VGT (Vacuolar Glucose Transporter), INT (Inositol Transporter), PMT/PLT (Polyol/Monosaccharide Transporter or Polyol Transporter), ERDL6 (Early Response to Dehydration-Like 6), and pGlcT (plastidic Glcose Transporter). These subfamilies are highly differentiated in terms of tissue specificity, substrate preference, and regulatory mechanisms, collectively forming a sophisticated monosaccharide transport network [10]. SUTs are also members of the MFS family and can be classified into five subgroups based on phylogenetic analysis and their distribution in monocots and dicots: SUT1 is specific to dicots, SUT3 and SUT5 are specific to monocots, while SUT2 and SUT4 are present in both [11].

The Bambusoideae, a highly specialized subfamily within the grass family (Poaceae), is widely distributed throughout tropical, subtropical, and temperate forest ecosystems. Comprising over 1700 species globally, bamboos represent a clade within the grasses that has evolved lignified stems, exhibiting remarkable morphological diversity [12]. Plant height varies tremendously among species, ranging from herbaceous bamboos only tens of centimeters tall (e.g., *Olyra latifolia*) [13] to giant woody bamboos exceeding 30 m (e.g., *Dendrocalamus sinicus*) [14]. This marked interspecific variation offers ideal natural material for dissecting the genetic basis of plant architecture establishment. Many woody bamboos can achieve astonishing growth rates during their peak growth period, elongating by tens of centimeters or even over one meter per day. For instance, Phyllostachys edulis (Moso bamboo) can reach a height of nearly 20 m and completes its vertical growth within just 35 to 40 days after shoot emergence [15]. This explosive growth relies on efficient photoassimilate mobilization and a robust sugar transport system. Notably, bamboos have undergone multiple WGD events during their evolution, resulting in lineages with varying ploidies [16]: diploid herbaceous bamboos (HB, 2n = 20–24), tetraploid temperate woody bamboos (TWB, 2n = 46–48), neotropical woody bamboos (NWB, 2n = 40–48), and hexaploid paleotropical woody bamboos (PWB, 2n = 70–72). We hypothesize that polyploidization may have contributed to the expansion and expression divergence of key sugar transporter gene families such as SUT and MST. Whether these changes have influenced sugar allocation efficiency and growth rates among bamboo lineages, particularly in hexaploid groups, remains an open question that we begin to address through comparative genomic and transcriptomic analyses.

Currently, research on bamboo sugar transporter genes is largely restricted to single species or single gene families. For example, Gu et al. [17] identified 50 SWEET family members in the *Dendrocalamus farinosus* genome, analyzing their phylogeny, expression patterns, and cis-acting elements to verify the function of key genes (e.g., *DfSWEET14*) in sugar transport and drought resistance. Gao et al. [18] isolated the sucrose transporter gene *BoSUT2* from *Bambusa oldhamii*, finding it localized to the plasma membrane and likely involved in sucrose loading in leaves and leaf sheaths. Ou et al. [19] identified nine SWEET family members from the *Bambusa emeiensis* transcriptome database, suggesting that *BeSWEET1a-2* functions primarily in source tissues while *BeSWEET4-2* functions in sink tissues.

Consequently, there is a lack of systematic cross-lineage comparisons, and few studies have explicitly linked these findings to the evolutionary context of polyploidization. To address this, we utilized the genomes of 14 bamboo species covering the four aforementioned lineages to systematically identify the SUT and MST gene families. This study aimed to characterize how this critical source–sink regulatory gene system has been reshaped by recurrent allopolyploidization events. Specifically, we investigated (1) the contribution of WGD to gene family expansion, (2) the patterns of gene retention and loss following polyploidization, and (3) the transcriptional divergence of duplicated genes across subgenomes. These analyses allow us to formulate hypotheses regarding how the evolution of the sugar transport system may have contributed to the divergent growth strategies of herbaceous and woody bamboos. In contrast to previous evolutionary analyses focused on the whole-genome scale [20], this research characterizes the expansion and divergence patterns of a specific functional gene family during polyploidization in detail, bridging a critical knowledge gap in the evolution of the source-sink regulation system in woody bamboos. The resulting patterns provide a foundation for future functional studies and offer candidate gene resources for source–sink co-evolutionary research in Poaceae crops.

## 2. Materials and Methods

### 2.1. Acquisition of Rice, Arabidopsis, and Bamboo Genome Data

Whole-genome sequences and gene annotation files for rice (*Oryza sativa*) were downloaded from the Rice Genome Annotation Project (RGAP) database [21] (https://rice.uga.edu/ [accessed on 7 January 2025]). Data for *Arabidopsis thaliana* were obtained from The Arabidopsis Information Resource (TAIR) database [22] (https://www.arabidopsis.org/ [accessed on 7 January 2025]). The genome data for *Dendrocalamus brandisii* were retrieved from the Supplementary Material of the corresponding publication (DOI: 10.6084/m9.figshare.24455197) [23]. Data for *Raddia distichophylla* were obtained from the BIG Genome Warehouse under accession number GWHAAKD00000000 [24]. Genome data for the remaining 12 bamboo species (*Ampelocalamus luodianensis*, *Bonia_amplexicaulis*, *D. sinicus*, *Guadua angustifolia*, *Hsuehochloa calcarean*, *Ph. edulis*, *Dendrocalamus latiflorus*, *Melocanna baccifera*, *Ol. latifolia*, *Otatea glauca*, *Raddia guianensis*, *Rhipidocladum racemiflorum*) were downloaded from BambooBase (https://bamboo.genobank.org/ [accessed on 13 January 2025]) [25]. Detailed assembly quality information for the 14 bamboo genomes is provided in Supplementary Table S1, with the assembly completeness evaluated via BUSCO using the poales_odb12 dataset.

### 2.2. Identification and Classification of Gene Families

Reference sequences for the SUT and MST gene families were retrieved from the rice and Arabidopsis databases. Hidden Markov Model (HMM) profiles for each gene family were constructed based on these reference sequences using the hmmbuild command from HMMER v3.4. Subsequently, the hmmsearch command was used to search for corresponding gene family sequences within the bamboo genomes based on the constructed HMMs, with an e-value threshold set at 0.0001 (notably, all identified hits possessed e-values well below this threshold) [26]. The candidate sequences were then subjected to domain verification using the NCBI Conserved Domain Database (NCBI-CDD) (preview version) [27].

The following conserved domains were used to classify the candidate proteins: GPH_sucrose for SUT; MFS_GLUT6_8_Class3_like for ERDL6; MFS_HMIT_like for INT; MFS_GLUT_like for pGLcT; MFS_PLT for PMT; MFS_STP for STP; MFS for TMT; and MFS_GLUT10_12_Class3_like for VGT. SFP9 and SFP14 were classified as members of the ERDL6 subfamily based on their phylogenetic relationships and conserved domain characteristics. The final gene names were assigned according to phylogenetic relationships with the corresponding *Oryza sativa* and *Arabidopsis thaliana* homologs.

### 2.3. Prediction of Physicochemical Properties, Structure, and Subcellular Localization

Physicochemical properties of the SUT and MST proteins, including amino acid count, molecular weight (MW), isoelectric point (pI), Instability Index, Aliphatic Index, and Grand Average of Hydropathicity (GRAVY), were analyzed using the ProtParam tool on the ExPASy server [28] (https://web.expasy.org/protparam/ [accessed on 5 February 2025]). Secondary structures were predicted using the SOPMA method available on the NPS@ server [29] (https://npsa-prabi.ibcp.fr/cgi-bin/npsa_automat.pl?page=/NPSA/npsa_server.html [accessed on 8 February 2025]). Subcellular localization predictions were performed using WoLF PSORT (https://wolfpsort.hgc.jp/ [accessed on 11 February 2025]) [30].

### 2.4. Phylogenetic Analysis and Visualization

Sequences from each bamboo species were combined with those from Arabidopsis and rice for each gene family. Multiple sequence alignments were performed using MAFFT [31], followed by sequence trimming with trimAl to remove poorly aligned regions [32]. Phylogenetic trees were then constructed using the Maximum Likelihood (ML) method implemented in IQ-TREE2 [33], with automatic model selection and 1000 bootstrap replicates for reliability assessment. The resulting phylogenetic trees were visualized using the Interactive Tree of Life (iTOL) (https://itol.embl.de/ [accessed on 15 April 2025]) [34].

### 2.5. Chromosomal Location and Duplication Analysis

The replication types of SUT and MST gene families in the 14 bamboo species were analyzed using MCScanX software with default parameters [35]. All-versus-all BLASTP searches (e-value < 1 × 10^−5^) were performed first, and the resulting blast outputs were used as input for MCScanX to classify genes into five categories: Singleton, Dispersed, Proximal, Tandem, and WGD/segmental. The chromosomal locations and synteny relationships of the genes were visualized using Circos (v0.69) software [36].

### 2.6. Calculation of Ka and Ks Values

Orthologous gene pairs were identified by reciprocal best BLASTP hits between species pairs (e-value < 1 × 10^−5^). Paralogous gene pairs were identified based on syntenic relationships derived from MCScanX. For each pair, protein sequences were aligned using MAFFT, and the protein alignments were converted into codon alignments using ParaAT v2 [37]. Ka and Ks values were calculated using KaKs_Calculator 2.0 with the YN model [38]. Because the total number of gene pairs was limited, all calculated Ka/Ks values were retained without applying filters for extremely low or saturated Ks values. No formal statistical test was performed to compare Ka/Ks against 1; therefore, we describe values below 1 as “consistent with purifying selection” rather than “significantly less than 1.”

### 2.7. Transcriptome Processing

Transcriptome data for the bamboo species were obtained from the NCBI Sequence Read Archive (SRA) under accession numbers PRJNA948693 and PRJNA649947. A detailed summary of the BioProjects, tissues, developmental stages, and replicate numbers used for each species is provided in Table S6. The raw reads were first processed with fastp to remove adapters and low-quality segments [39], then aligned to their respective reference genomes using HISAT2 [40]. Transcript abundance was then quantified using StringTie [41], yielding expression levels in Transcripts Per Kilobase of exon model per Million mapped reads (TPM). To enable valid cross-species comparisons and mitigate potential batch effects inherent in public SRA datasets, we avoided direct comparisons of absolute expression values. Instead, tissue specificity was calculated based on the TPM matrix using the tspex tool [42]. This approach converts TPM into a relative tissue specificity index, thereby standardizing heterogeneous datasets for robust evolutionary comparisons. We acknowledge that differences in experimental design, sampling, and sequencing depth among datasets may still influence cross-species comparisons, and our conclusions are therefore restricted to broad patterns of tissue preference.

### 2.8. Statistical Analysis

Due to the small number of species in each lineage and the confounding between ploidy and lineage (e.g., HB are diploid, NWB and TWB are tetraploid, PWB are hexaploid), gene-count comparisons among lineages were descriptive and were not subjected to formal hypothesis testing. Therefore, the term “significant” was avoided in this context. For expression data, tissue specificity indices were compared qualitatively across species.

## 3. Results

### 3.1. Genome-Wide Identification and Expansion Patterns of SUT and MST Gene Families

Using HMM (Hidden Markov Model) profiles, we identified a total of 114 SUT, 110 ERDL6, 47 INT, 68 pGlcT, 6 PMT, 512 STP, 100 TMT, and 30 VGT genes from the 14 collected bamboo genomes (Table S2). The PMT gene was exclusively identified in the *Ot. glauca* (NWB) and PWB lineages, with only one copy present in each species. Additionally, no VGT genes were identified in *Ra. distichophylla* (HB).

**Figure 1:**
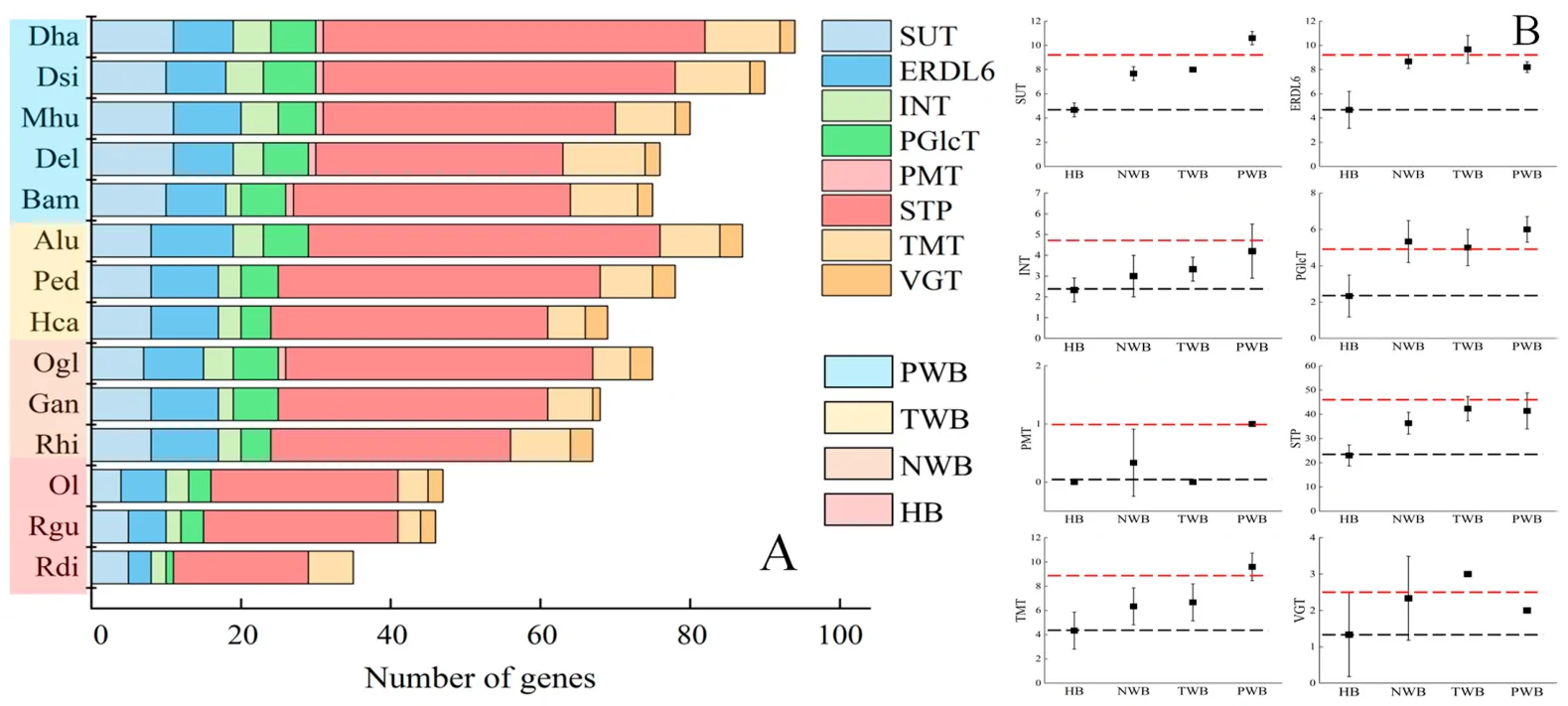
Distribution of gene numbers in eight gene families across four bamboo lineages. (**A**) Gene numbers of eight gene families in four lineages (HB, TWB, NWB, and PWB); (**B**) Average gene number per gene family in each lineage. The black line represents the average value for HB, serving as the reference baseline; the red line represents twice the value of the black line. Alu: *A. luodianensis*; Bam: *B. amplexicaulis*; Dha: *D. brandisii*; Dsi: *D. sinicus*; Gan: *G. angustifolia*; Hca: *H. calcarean*; Ped: *Ph. edulis*; Del: *D. latiflorus*; Mhu: *M. baccifera*; Ol: *Ol. latifolia*; Ogl: *Ot. glauca*; Rdi: *Ra. distichophylla*; Rgu: *Ra. guianensis*; Rhi: *Rh. racemiflorum*.

There was marked variation in gene copy numbers among different species, which correlated with species ploidy, ranging from 35 genes in the diploid herbaceous bamboo (Rdi) to 94 in the hexaploid woody bamboo (Dha) (Figure 1A). Overall, woody bamboos (tetraploids and hexaploids) possessed significantly more genes than herbaceous bamboos (diploids). To precisely investigate the relationship between gene count and ploidy, we compared the average gene number across the four lineages (Figure 1B). Compared to herbaceous bamboos (2×), neotropical and temperate woody bamboos (4×) had approximately double the average gene count, consistent with their ploidy levels. However, although paleotropical woody bamboos (6×) had the highest average count among the four lineages, it was markedly lower than a simple theoretical triple value derived from herbaceous bamboos. This pattern is consistent with post-polyploid gene fractionation, but we emphasize that this is a descriptive observation rather than a formal test of gene loss. Without a robust reconstruction of ancestral gene content across the reticulate history of these lineages, the extent and timing of gene loss cannot be precisely quantified. A similar pattern was observed in the SUT family and most MST subfamilies.

The physicochemical properties and secondary structures of the proteins within each gene family showed high conservation across the 14 bamboo species (Figures S1–S16). Subcellular localization predictions indicated that localization was not lineage-dependent; both SUT and MST proteins were predicted with high probability to be localized to the plasma membrane (Figures S17–S37).

### 3.2. Phylogenetic Relationships and Duplication Mechanisms of SUT and MST Genes

**Figure 2:**
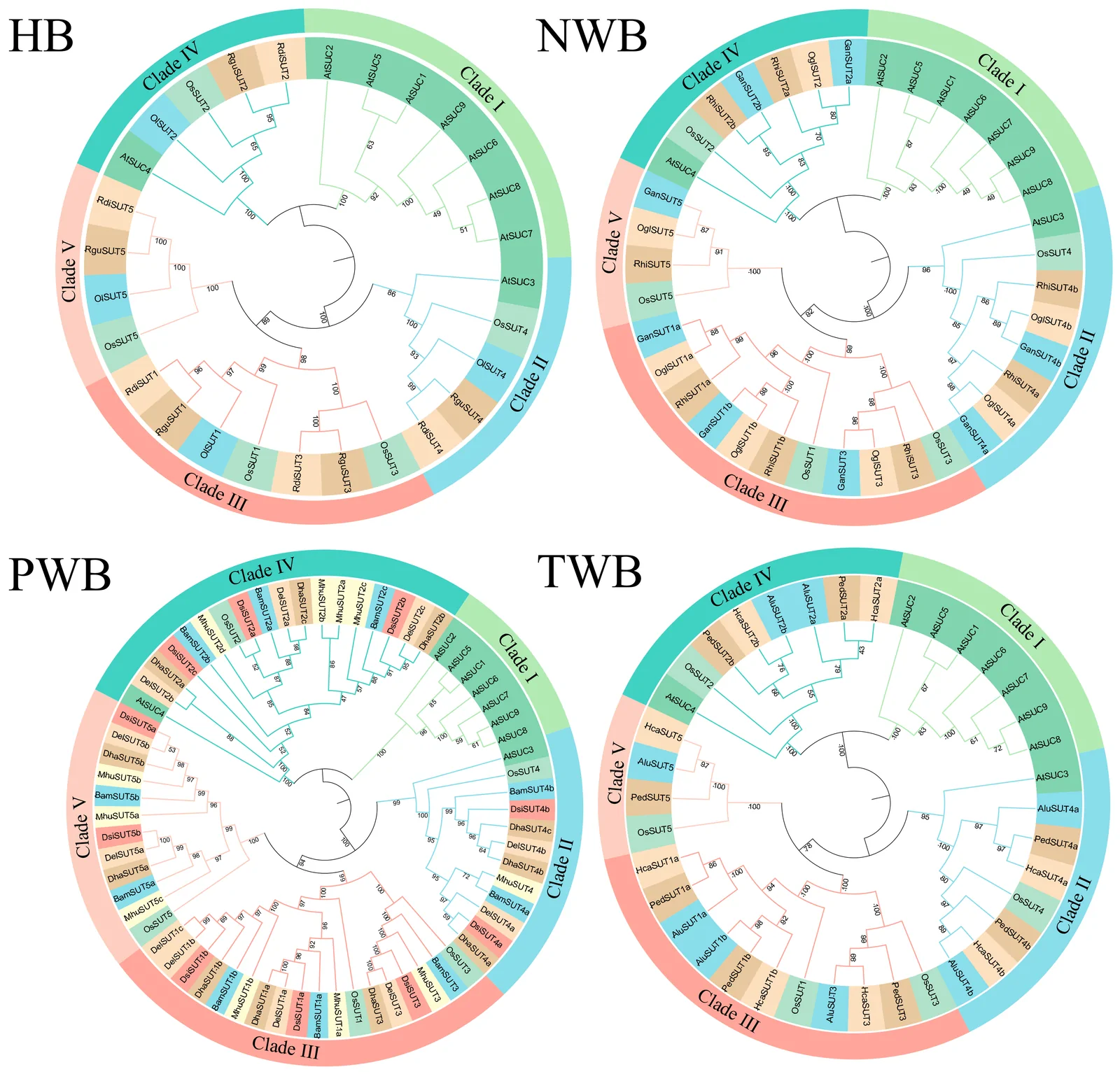
Phylogenetic tree of the SUT gene family from 14 bamboo species across 4 lineages.

To understand the evolutionary relationships of these genes among the 14 bamboo species and model plants (rice and Arabidopsis), we constructed ML phylogenetic trees for the eight gene families (Figure 2 and Figures S38–S44). The SUT family was divided into five clades (Figure 2): Clade I contained only Arabidopsis SUT proteins; Clades II and IV contained proteins from rice, Arabidopsis, and bamboo; while Clades III and V contained rice and bamboo proteins but lacked Arabidopsis sequences. The ERDL6 family was divided into three clades (Figure S38): Clade I contained only Arabidopsis sequences, while Clades II and III contained sequences from rice, Arabidopsis, and bamboo, with bamboo sequences distributed across both clades in all four lineages. The INT family was divided into three clades (Figure S39); apart from Clade II, which lacked bamboo sequences in herbaceous lineages, all three clades in other lineages contained proteins from rice, Arabidopsis, and bamboo. The pGlcT family comprised three clades (Figure S40), with all lineages represented across all clades alongside rice and Arabidopsis. The PMT family contained only six genes across the 14 bamboo species (Figure S41): one in each PWB species and one in Ogl (NWB); no PMT genes were found in rice. Bamboo and Arabidopsis PMT sequences formed distinct, highly supported branches. The STP family was divided into four clades (Figure S42), all containing sequences from rice, Arabidopsis, and bamboo: Clade I mainly comprised bamboo *STP1*; Clade II contained bamboo *STP2*, *STP7*, *STP13*, and *STP14* homologs; Clade III mainly comprised bamboo *STP3*; and Clade IV contained bamboo *STP5* sequences. The TMT family was divided into three clades (Figure S43), with Arabidopsis sequences found only in Clade I, while rice and bamboo sequences were distributed across all branches. The VGT family was divided into two main clades based on Arabidopsis references (Figure S44): Clade I (containing *AtVGT1/2*) and Clade II (containing *AtVGT3*), with bamboo sequences distributed in both.

Notably, several gene families exhibited high protein sequence conservation across bamboo species. A total of 21 gene groups sharing identical protein sequences were identified, including both cross-species orthologs and within-species paralogs (Table 1). Specifically, there were 2 groups in the SUT family, 2 in ERDL6, 3 in pGlcT, 1 in TMT, and a maximum of 13 groups in the STP family. In contrast, no such fully conserved sequences were found in the INT, PMT, or VGT families. These genes sharing identical sequences were predominantly distributed within the same phylogenetic clades. For instance, in the STP family, 13 groups showed 100% sequence identity between species, further suggesting the sequence conservation of key members within this family.

The vast majority of these identical protein sequences were found in PWB, with no cross-lineage occurrences observed. Only one group was found in temperate woody bamboos (*PedpGlcT1a* and *AlupGlcT1b*). Interestingly, *PedpGlcT1a* and *AlupGlcT1b* were both located on the 01C subgenome with highly similar genomic positions, suggesting a common evolutionary origin and conserved genomic structure.

To further evaluate the evolutionary selective pressures acting on these gene families, we calculated the nonsynonymous to synonymous substitution rate ratios (Ka/Ks) for orthologous and paralogous gene pairs in four representative bamboo species: *Ol. latifolia*, *Ph. edulis*, *G. angustifolia*, and *D. latiflorus*. As shown in Tables S4 and S5, the Ka/Ks values for both orthologous and paralogous genes across all investigated gene families were below 1. These values are consistent with purifying selection acting on these genes during their evolutionary history, which further explains the exceptionally high sequence conservation observed among the bamboo species.

To investigate the evolutionary trajectories of SUT and MST families during bamboo polyploidization, we analyzed synteny among *Ol. latifolia* (Ol, 2×), *Ph. edulis* (Ped, 4×), and *D. latiflorus* (Del, 6×). Taking SUT genes as an example (Figure 3), they were concentrated on a few chromosomes, showing a non-uniform distribution. In *Ol. latifolia*, few SUT genes were detected. In *Ph. edulis* and *D. latiflorus*, SUT gene numbers increased significantly and were widely distributed across multiple syntenic blocks. Although *D. latiflorus* has a higher ploidy than *Ph. edulis*, both showed similar SUT gene counts and chromosomal distribution patterns, showing no linear increase corresponding to ploidy. Furthermore, subgenome localization suggested that most SUT genes in *Ph. edulis* and *D. latiflorus* were located in highly conserved syntenic blocks, often appearing as pairs or clusters on chromosomes from different subgenomes (Figures S45–S48). Gene localization and synteny for the remaining families are detailed in Supplementary Figures S49–S74.

Gene family expansion is always accompanied by gene duplication and loss. We analyzed five duplication types (Singleton, Dispersed, Proximal, Tandem, and WGD/segmental) for the eight gene families using MCScanX (Table S3). Overall, expansion was dominated by WGD, with Tandem duplication playing a supplementary role in some families like ERDL6 and PMT. Gene counts were significantly correlated with ploidy and lineage (HB < NWB ≈ TWB < PWB). SUT expansion was primarily WGD-driven, with some Dispersed duplications; PWB had the most WGD-derived genes, while TWB species stably possessed seven genes, and HB had significantly fewer. ERDL6 expansion was driven by WGD, Dispersed, and Tandem duplications; Tandem types were common in HB, NWB, and TWB but only detected in Bam within PWB. INT was similar to SUT but with a higher proportion of Dispersed types due to lower total gene count, and Proximal types appeared in HB. For pGlcT, HB genes expanded via Dispersed duplication, while the three woody lineages relied mainly on WGD. The PMT family was highly conserved, with single WGD-derived copies detected only in Ogl (NWB) and the five PWB species, showing lineage specificity. STP expansion differed significantly between herbaceous and woody bamboos: woody bamboos were driven mainly by WGD (reaching high numbers like 32 in Dsi and Dha), accompanied by Tandem, Proximal, and Dispersed types; whereas HB relied primarily on Dispersed duplication, with far fewer WGD-derived genes. TMT had many WGD-derived and few Dispersed-derived genes, with occasional Tandem types in Rdi and Del. The VGT family was small but showed expansion driven by WGD and Dispersed duplications, with single Proximal-derived genes in Del and Dha, indicating high stability and minimal expansion.

### 3.3. Transcriptional Expression and Expression Divergence of SUT and MST Genes

To explore the functional evolution of SUT and MST families across lineages, we systematically analyzed tissue-specific expression patterns in 14 bamboo species. Overall, many genes showed marked tissue preference and subgenome-specific expression. Taking SUT and STP as examples, we selected representative species from each lineage: *Ol. latifolia* (HB), *Ph. edulis* (TWB), *G. angustifolia* (NWB), and *D. latiflorus* (PWB), and performed heatmap analysis (Figure 4, Figure 5, Figure 6, Figure 7, Figure 8, Figure 9, Figure 10 and Figure 11; see Figures S75–S104 for others).

In *Ol. latifolia* (Figure 4), *OlSUT1* was enriched in seeds and vegetative leaf blades; *OlSUT5* was specifically highly expressed in internodes; while *OlSUT2* and *OlSUT4* showed balanced expression across tissues. In *Ph. edulis* (Figure 5), the 8 SUT genes showed diverse patterns. *PedSUT5* was mainly expressed in floral organs and shoot apical meristems; *PedSUT1b* preferred rhizomes, roots, and mature shoot internodes; *PedSUT1a* was high in spikelets and seeds; *PedSUT3* was specific to pre-anthesis spikelets; while *PedSUT2a/2b* and *4a/4b* were relatively constant. *PedSUT1a* and 1b, as paralogs on different subgenomes, showed marked expression divergence, consistent with the partitioning of ancestral regulatory roles or the acquisition of new expression specificities. In *G. angustifolia* (Figure 6), *GanSUT1a* was highly expressed in branch buds and root apical meristems; *GanSUT1b* was detected only in intercalary shoot internodes but at extremely high abundance; *GanSUT3* was high in intercalary shoot internodes, culm sheaths, and vegetative leaf sheaths; *GanSUT5* was high in source organs like leaves; others lacked obvious preference. Paralogs *GanSUT2a* and *2b* (on different subgenomes) had similar patterns, whereas *GanSUT1a* and *1b* showed distinct subgenome expression bias. In *D. latiflorus* (Figure 7), *DelSUT1b* and *DelSUT3* were abundant in lateral buds; *DelSUT1c* was high in lateral buds and shoots; *DelSUT1a* was the highest in leaves and roots but widespread. Genomic analysis showed that *DelSUT1a* is on subgenome B, while *1b* and *1c* are on chromosome 3 of subgenome C; this positional difference aligns with their specialized expression profiles, suggesting expression divergence.

In *Ol. latifolia* (25 STP genes, Figure 8), *OlSTP1a* was high only in vegetative leaf blades; *OlSTP1g* high in apical shoot internodes; *OlSTP1c/2/5d* were undetected; six genes (e.g., *OlSTP5f*, *5h* and *5a*) were ubiquitously expressed; 12 genes (e.g., *OlSTP1e*, *3d* and *3b*) were high in leaves and mature internodes; *OlSTP5c/5e* were marked in internodes and seeds. In *Ph. edulis* (43 STP genes, Figure 9), *PedSTP1o/5k* were undetected. Six genes (e.g., *PedSTP1n*, *2* and *1c*) were specific to pre-anthesis spikelets; *PedSTP5j* was specific to primary spikelets. Fifteen genes (e.g., *PedSTP3g*, *13b* and *5e*) were high in roots, seeds, and leaves; others were ubiquitous. Subgenome analysis suggested diverse fates: *PedSTP5j/5e/5c* are all on chromosome 04C but showed three different patterns, whereas *PedSTP5d* and *5l* (both on 4D) had similar patterns. In *G. angustifolia* (36 STP genes, Figure 10), *GanSTP1a/2a* were undetected. Seven genes (e.g., *GanSTP1j*, *5f* and *1k*) were high in apical meristems and shoot internodes; five (e.g., *GanSTP3g*, *13b* and *3f*) were specific and highly abundant in vegetative leaves and sheaths. Nineteen genes (e.g., *GanSTP1e*, *3d* and *13a*) were high in leaves, rhizomes, and buds but low in internodes. *GanSTP1h/5c/2b* were high in branch buds, with *5c* and *2b* also high in mature internodes. Subgenome analysis showed *GanSTP3g/3e/3f* (on 10C) had similar patterns, while *GanSTP5a* and *5c* (on 04C) diverged functionally. *GanSTP5b* (04B) differed from 5a and 5c. In *D. latiflorus* (33 STP genes, Figure 11), *DelSTP1d/1e/1b* were undetected. *DelSTP1a* and *5c* were specific to the stem and root, respectively. Seven genes (e.g., *DelSTP7b*, *5i* and *5a*) were high in leaves and culms. Fifteen genes (e.g., *DelSTP13a*, *5n* and *1g*) were high in shoot shells and roots but low in internodes and stems. Six genes (e.g., *DelSTP1f*, *5d* and *3a*) were high in roots, leaves, and lateral buds. Subgenomically, *DelSTP1d/1e/1b* (on A, B, C subgenomes) shared the same silenced pattern, whereas *DelSTP5m/5g/5h* (also on A, B, C) exhibited different expression patterns.

Given that the culm is the most distinct feature of bamboo, its rapid elongation and lignification demand immense sugar transport. Beyond the SUT and STP analysis, we comprehensively compared the eight families’ expression in herbaceous vs. woody culms. Genes highly expressed in the culm were mostly ubiquitously expressed, with few exhibiting culm-specific high expression, showing clear functional complementarity or specialization across lineages. Specifically, SUT function in herbaceous culms relied on *SUT5*, whereas woody bamboos utilized *SUT2* and *SUT4* (expanded via WGD) to share the transport load via ubiquitous expression. The STP family, being the largest, showed the most marked differentiation: herbaceous culms relied on *STP3* and *STP5*, while woody bamboos significantly expanded these subfamilies, likely for fine-tuned regulation of sugar accumulation and transport at different developmental stages. ERDL6 and pGlcT families demonstrated a trade-off between gene number and expression breadth; ERDL6 was ubiquitous in herbaceous bamboo but dominated by *SFP9* and *SFP14* in woody bamboo (with *SFP9* potentially reinforced by paralog expansion).

The smaller pGlcT family compensated for low numbers in herbaceous bamboo via broad expression, while showing specialization in woody bamboo (e.g., *GanpGlcT1c* specific to rhizome and culm sheath). The PMT family was unique: identified only in Ogl and PWB, its expression was low in culms but extremely high in roots and rhizomes, suggesting a possible role in underground transport processes that may indirectly support aerial growth. However, this remains a hypothesis based solely on expression localization, and functional validation is required. The relatively conservative INT, TMT, and VGT families showed stable expression of key members (*INT1/2/3*, *TMT1/3*) across tissues and lineages, performing basal physiological functions. VGT, with only 1–3 homologs per species and little differentiation, likely maintains basal vacuolar glucose homeostasis.

## 4. Discussion

### 4.1. Gene Family Expansion and Selective Loss Caused by Whole-Genome Duplication

Polyploidization, or WGD, involves the doubling of chromosomes and all genes within a genome. It serves as a major driver of species diversity and provides abundant genetic material for plant evolution [43]. However, following duplication, most replicated genes are eventually lost, with only a fraction of homologous genes being retained over long periods [44]. Ma et al. [45] proposed a model for the origin and polyploid evolution of bamboo. According to this model, the earliest ancestor of bamboos diverged approximately 40 million years ago (Mya), forming the ancestral lineages of herbaceous and woody bamboos. Subsequently, around 32 Mya, the ancestor of woody bamboos differentiated into two diploid ancestral lineages, designated as B and C0; C0 later evolved into C1 and C2. Around 30 Mya, lineages B and C0 hybridized to form two additional diploid ancestral lineages of woody bamboos, A and D. By approximately 21 Mya, lineage B hybridized with lineage C1 and underwent polyploidization, giving rise to neotropical woody bamboos (genome composition BBCC). Later, no later than 13 Mya, this tetraploid (BBCC) hybridized with lineage A and underwent another round of polyploidization, forming the hexaploid paleotropical woody bamboos (genome composition AABBCC). Finally, no later than 12 Mya, a third hybridization and polyploidization event occurred between C2 and D, resulting in the evolution of temperate woody bamboos (genome composition CCDD).

Genes typically exhibit dosage effects, where expression levels are directly correlated with copy number. Large-scale structural variations in chromosomes often trigger genome-wide feedback regulation, which subsequently affects gene expression in a cascading manner. When feedback regulation interacts with dosage effects, it manifests as gene expression compensation, maintaining expression levels close to normal diploid states despite changes in chromosomal dosage [46]. In this study, the number of SUT and MST genes identified in woody bamboos (TWB, NWB, PWB) was significantly higher than in herbaceous bamboos (HB) (Figure 1). Compared to herbaceous bamboos, woody bamboos generally possess greater biomass and height, necessitating a more robust sugar transport and allocation system. We hypothesize that the expansion of these sugar transporter families provides the critical molecular basis to meet this demand for efficient carbon allocation [47]. Detailed analysis of the relationship between gene count and ploidy suggested distinct evolutionary trajectories across lineages. In tetraploid woody bamboo lineages (4×, TWB, and NWB), the average number of SUT and MST genes was approximately double that of the diploid herbaceous lineage (2×, HB), indicating minimal gene loss following the hybridization and polyploidization of diploid ancestors. However, in the hexaploid woody bamboo lineage (6×, PWB), the gene count for each family was significantly lower than the theoretical triple value calculated based on herbaceous bamboos. This discrepancy suggests that extensive gene loss occurred in the PWB lineage following its WGD events [48,49]. This phenomenon is reminiscent of the gene dosage balance hypothesis, which posits that polyploids tend to lose duplicated genes to restore stoichiometric balance in protein complexes and regulatory networks [50]. However, our data are consistent with this general model of post-polyploid genome evolution but do not directly test dosage sensitivity for these specific transporter families. Furthermore, because the gene count comparisons are based on extant species rather than reconstructed ancestors, and because ploidy is confounded with lineage identity, these patterns should be regarded as preliminary. A more rigorous test would require integration of syntenic homeolog reconstruction and phylogenetic comparative methods across a larger sample of species.

Furthermore, gene retention rates varied unevenly among different SUT and MST families. While SUT and STP subfamilies expanded significantly, the expansion of PMT and VGT subfamilies was limited due to their inherently low initial numbers. Notably, PMT genes were found only in specific species and lineages (Ogl and PWB) with very low copy numbers, while VGT was absent in Rdi. The exclusivity of PMT implies that its function may be highly specific in certain bamboo lineages or functionally redundant with other transporters [11,51]. Regarding the absence of VGT in Rdi, while this might reflect a unique sugar utilization strategy in this herbaceous bamboo, it could also be attributed to incomplete genome assembly or annotation gaps [24]. Despite variations in copy number, the physicochemical properties and subcellular localization of the identified proteins remained highly conserved (Figures S17–S37), indicating that while gene dosage was modulated by evolutionary forces, the fundamental biological functions of these transporters were strictly maintained [52,53,54].

### 4.2. WGD-Driven Gene Duplication and Purifying Selection

Unlike other eukaryotic genomes, plant genomes tend to evolve at a faster rate, resulting in higher genomic diversity [55,56]. Plant genomes are rich in duplicated genes; WGD events have occurred multiple times during the past 200 million years of angiosperm evolution [57,58], and genomic sequencing continues to suggest new WGD events [59]. Gene family expansion is inextricably linked to gene duplication events [60]. WGD contributes a massive number of gene copies simultaneously, serving as the primary source of gene family expansion [61], while tandem duplication creates clusters of closely related homologous genes in local regions [62].

Analysis of duplication types based on MCScanX clearly suggested differences in expansion patterns among lineages (Table S3). In HB, genes mostly existed as Dispersed or Proximal types and were few in number. In contrast, woody bamboos (NWB, TWB, PWB) showed a marked increase in members of families like SUT, pGlcT and STP, the vast majority of which were derived from WGD. Particularly in the STP family, PWB species such as Dsi and Dha possessed up to 32 genes, several times that of herbaceous bamboos, consistent with the complex allopolyploid origins (4× and 6×) of woody bamboos. However, different gene families showed distinct tolerances for duplication types and expansion. In woody bamboos, STP and SUT families not only retained a large number of copies via WGD but were also accompanied by Tandem or Dispersed types. Generally, WGD retains core, complex genes, while Tandem duplication retains genes that need to evolve rapidly to cope with external pressures. This suggests distinct dosage effects for these sugar transporters; high copy numbers likely help meet the demand for efficient photoassimilate transport required for the rapid growth of woody bamboos. Conversely, the PMT family was strictly limited in copy number, with only 6 genes identified across 14 bamboo species (Figure S41), all belonging to *PMT4*, and none found in rice.

Another intriguing finding of this study was the identification of 21 groups of genes sharing identical protein sequences (Table 1), a phenomenon relatively rare in rapidly evolving gene families. Of these, 19 groups involved identical sequences from different bamboo species (e.g., *DelSUT2c* and *DhaSUT2b*), while 2 groups consisted of within-species paralogs (*DelSUT1b*/*DelSUT1c* and *DhaSTP1h*/*DhaSTP1i*). Although phylogenetic analysis (Figure 2 and Figures S38–S44) showed clear evolutionary divergence among bamboo, rice, and Arabidopsis, the existence of these completely identical sequences within bamboo lineages suggests that specific genes are under strong functional constraints that limit sequence variation. The STP family possessed the most conserved genes (13 groups, all cross-species), followed by pGlcT (3 groups, all cross-species).

Several explanations may account for this high sequence conservation:

Strong purifying selection. These genes may perform indispensable functions, such as high-affinity transport of monosaccharides during rapid shoot elongation. Key domains (e.g., transmembrane regions, substrate-binding sites) may be under strong selective constraint, where most amino acid substitutions are deleterious [12]. For instance, the complete identity between *DelSUT2c* (6×) and *DhaSUT2b* (6×) suggests that the coding sequence of this homolog has been tightly constrained, despite complex genomic rearrangements in hexaploids.

Recent divergence. Species within the PWB lineage may have diverged relatively recently, so insufficient time has elapsed for mutations to accumulate.

Conserved genomic context. The observation that PedpGlcT1a and AlupGlcT1b are both located on the 01C subgenome at similar positions suggests that certain loci may reside in genomic regions with lower substitution rates or stronger structural constraints.

**Figure 3:**
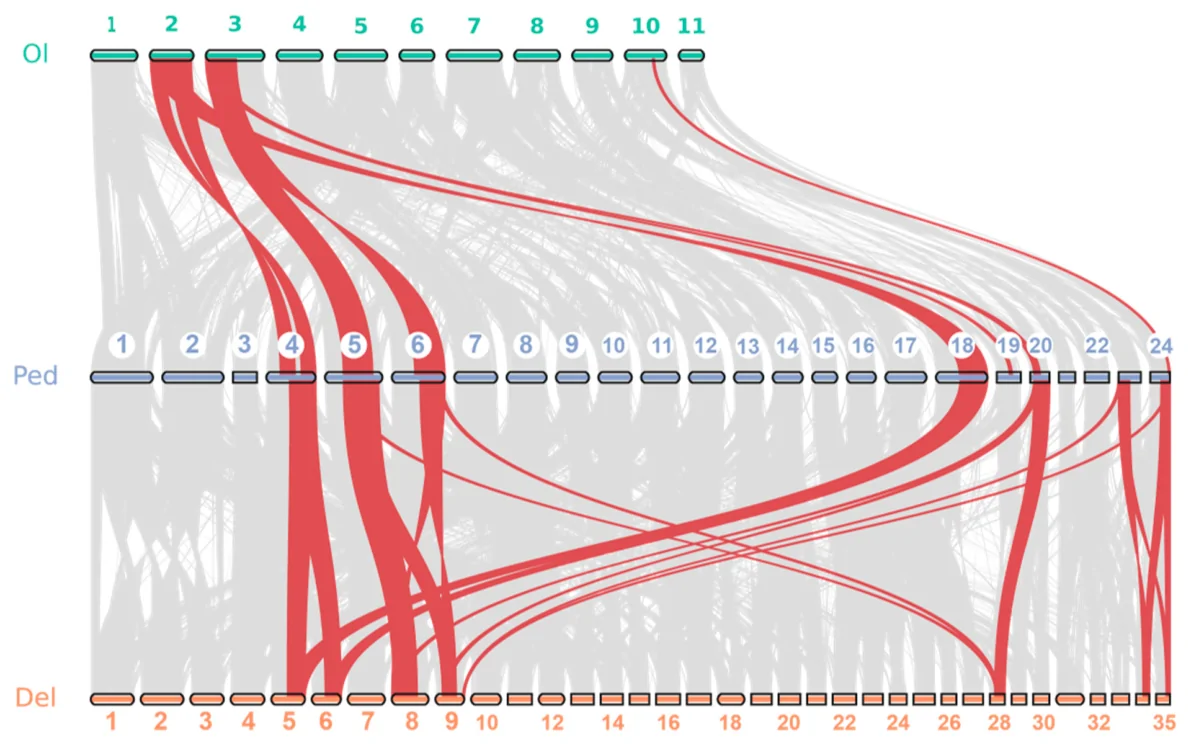
Syntenic relationships of SUT genes among *Ol. latifolia* (2×, Ol), *Ph. edulis* (4×, Ped), and *D. latiflorus* (6×, Del). Top, middle, and bottom are the chromosomes of Ol, Ped, and Del, respectively. Colored bars represent chromosomes, and the numbers above/below them are chromosome numbers. Gray lines indicate all collinear gene pairs between genomes, and red lines indicate collinear pairs of members of the four highly conserved gene families SUT, ERDL6, pGlcT, and STP.

Among the 21 groups of identical sequences, the majority (19 groups) involved cross-species comparisons, and these were predominantly distributed within the PWB lineage. The two within-species paralog groups were also from PWB species. This pattern is consistent with lineage-specific sequence conservation. High sequence identity, combined with observed conserved synteny (e.g., SUT genes located in highly conserved syntenic blocks, Figure 3), further reinforces the conclusion that the core sugar transport mechanism in bamboo has remained exceptionally stable since the divergence of woody bamboos. This stability likely ensures the reliability of sugar allocation during bamboo’s unique explosive growth phase.

### 4.3. Asymmetric Expression and Expression Divergence of Homologous Genes Among Subgenomes

Polyploidization is invariably accompanied by rapid reprogramming of transcriptomic networks. A major consequence of WGD is the generation of redundant gene copies, which subsequently follow three main evolutionary fates [63]: (1) one copy accumulates deleterious mutations and eventually loses function, becoming a pseudogene; (2) one copy acquires a novel beneficial function and is retained by natural selection (neofunctionalization), while the other retains the original function; (3) both copies lose partial functions of the original gene through mutation but complement each other to fulfill the total original function (subfunctionalization), resulting in the retention of both. Functional asymmetry of subgenomes or transcriptomes has been shown to play a key role in allopolyploid speciation and environmental adaptation [64]. Wang et al. found that evolution and domestication enhanced the expression dominance of the A subgenome in tetraploid wheat (BBAA), with homologous genes evolving from common regulation to differential regulation [65]. You et al. [66], through resequencing and transcriptomic analysis of 376 tetraploid cotton accessions (AADD), suggested expression asymmetry of homologs among subgenomes and found that coordinately optimizing homologs in subgenomes could maximize agronomic traits. Mao et al. [67] found asymmetric expression of GA-related genes: in large-sized woody bamboos (e.g., *D. sinicus*, *Ph. edulis*), A and C subgenome expression dominated, whereas in small-sized woody bamboos (e.g., *B. amplexicaulis*), B and D subgenome expression was higher.

**Figure 4:**
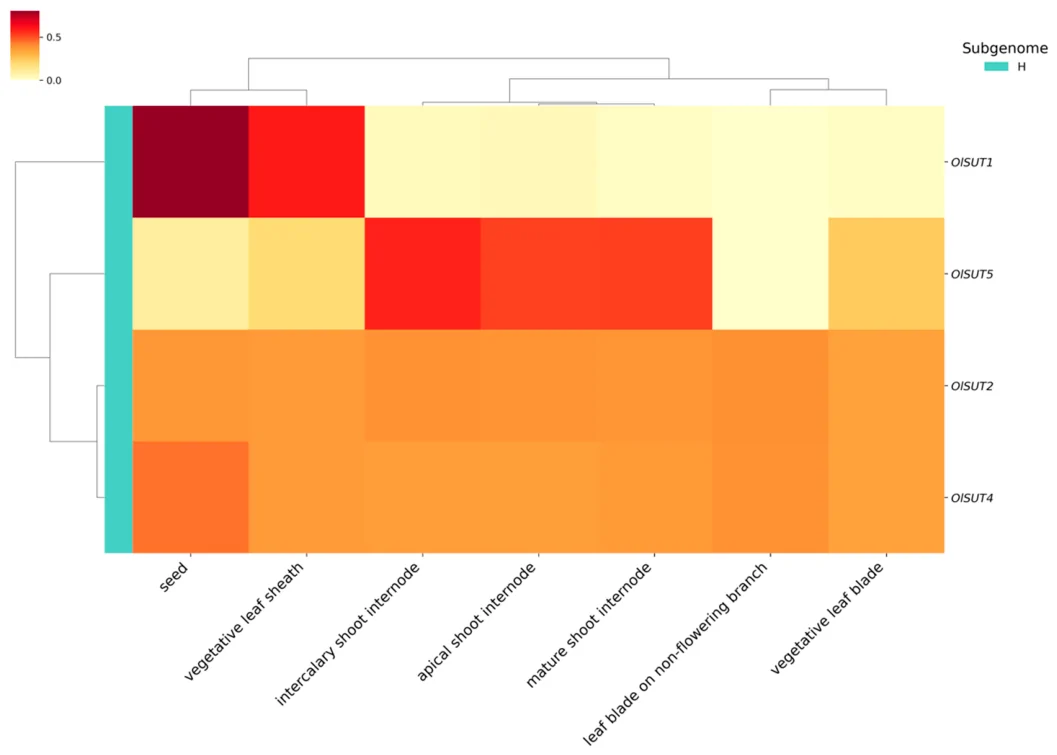
Herbaceous Bamboo, Ol. latifolia SUT Transcriptome.

**Figure 5:**
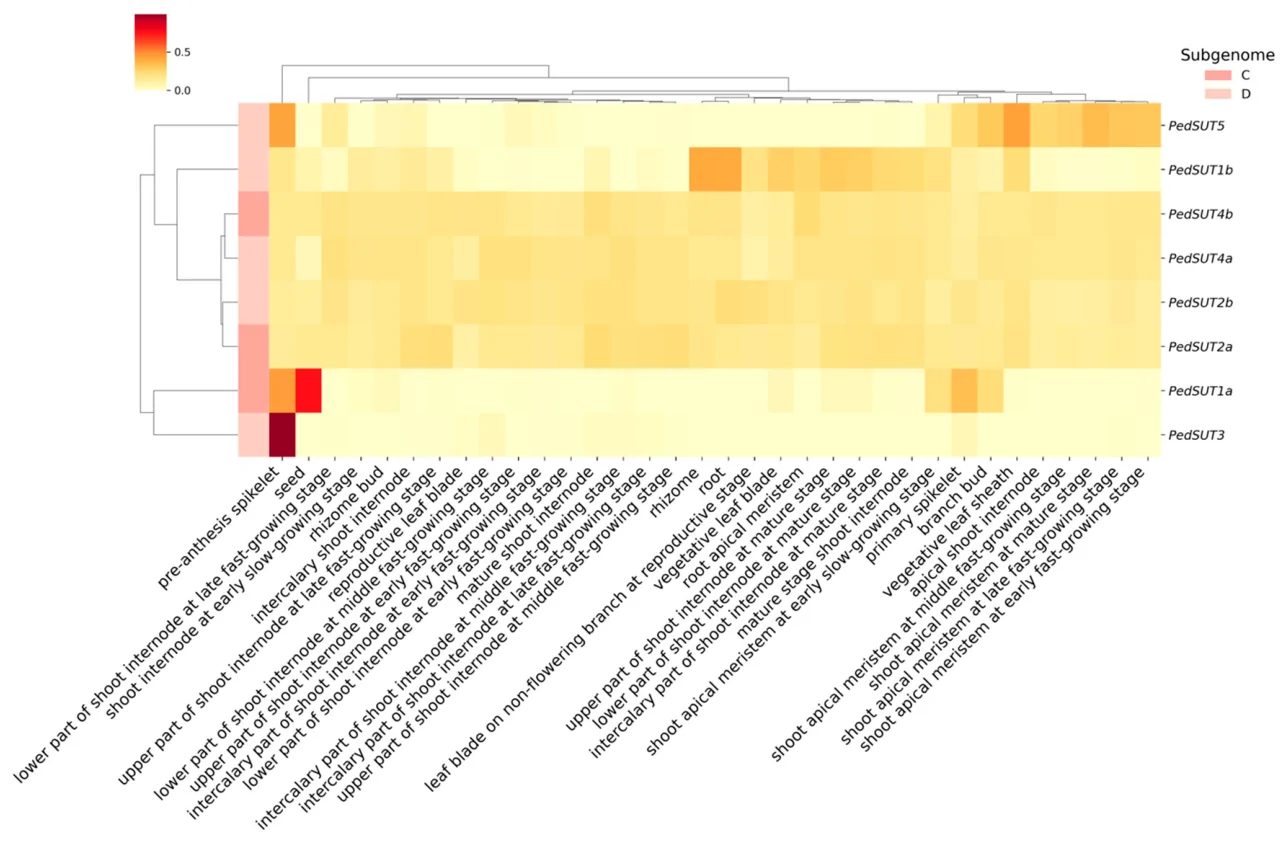
Temperate woody bamboo, *Ph. edulis* SUT transcriptome.

**Figure 6:**
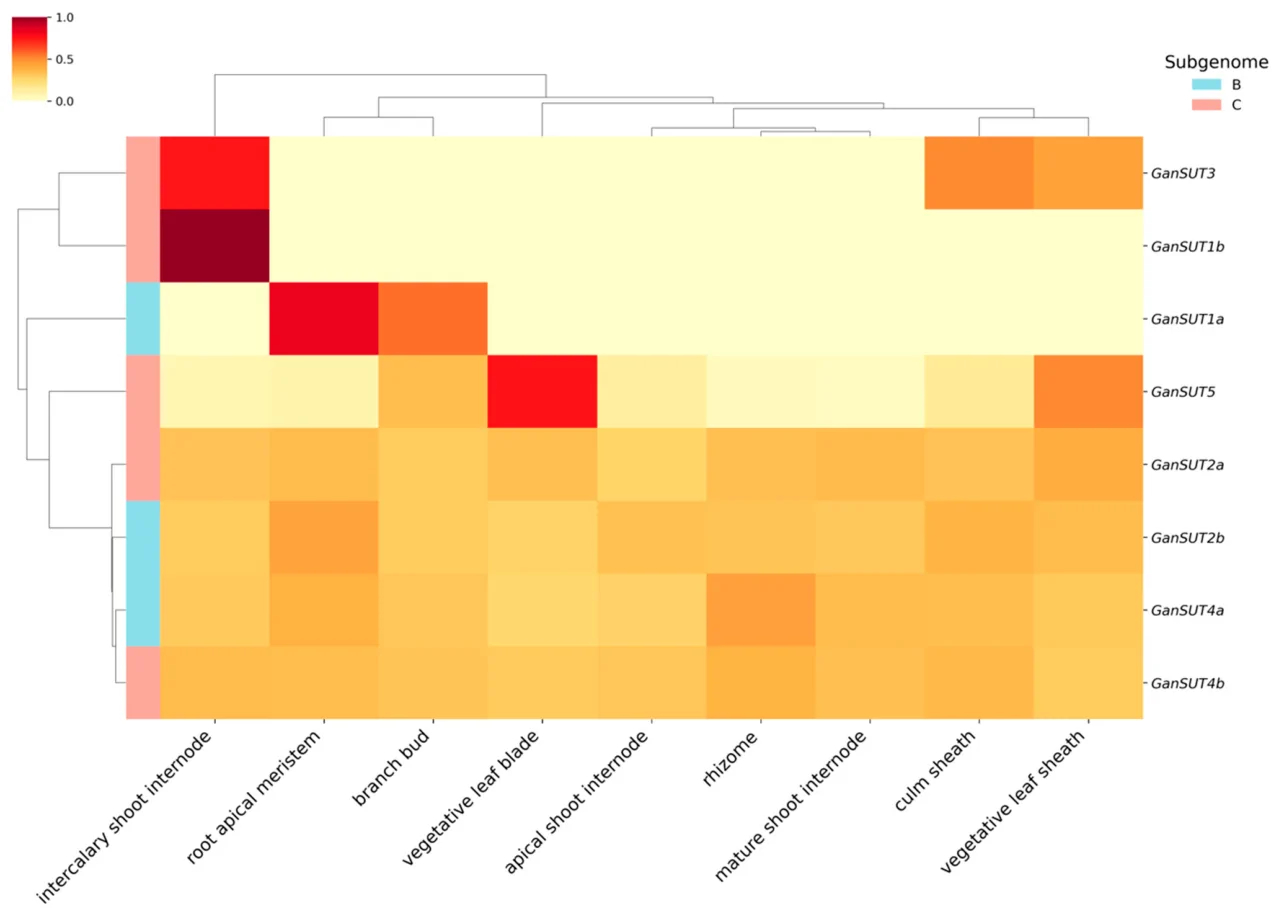
Neotropical woody bamboo, *G. angustifolia* SUT transcriptome.

**Figure 7:**
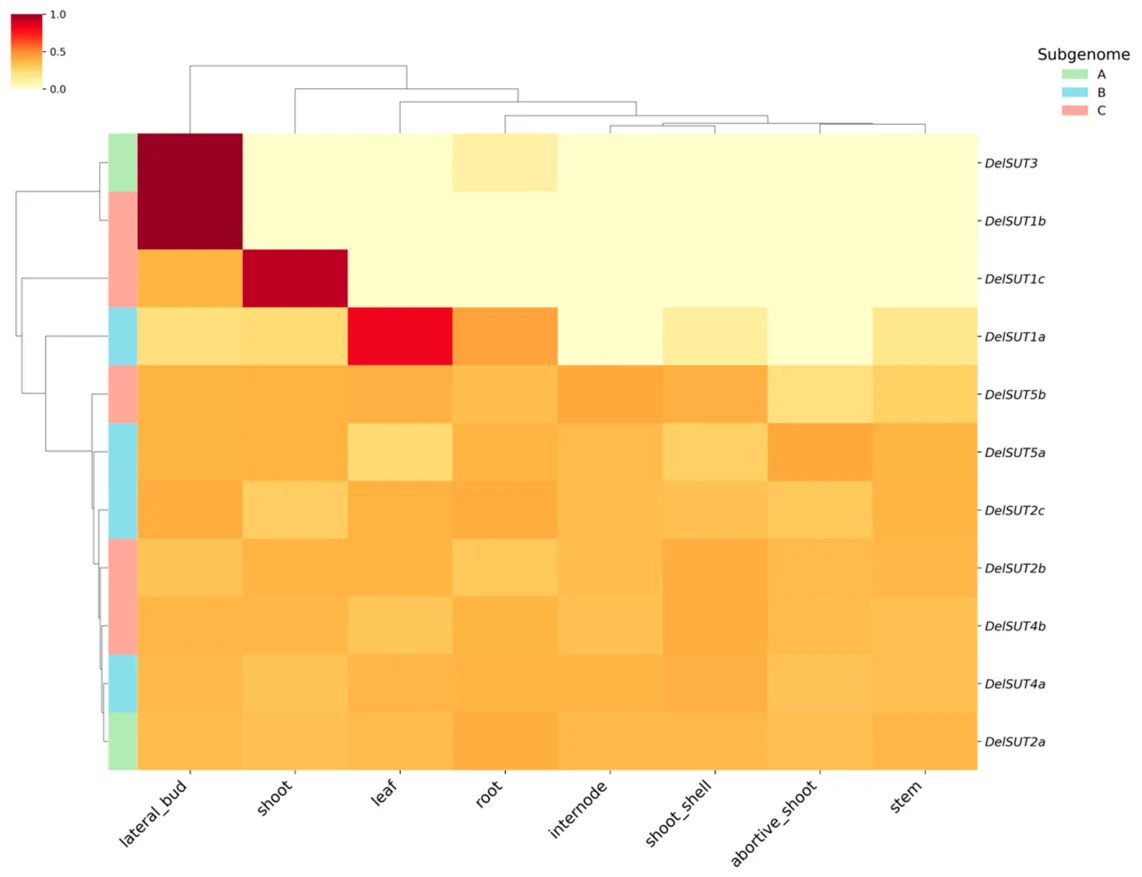
Paleotropical woody bamboo, *D. latiflorus* SUT Transcriptome.

**Figure 8:**
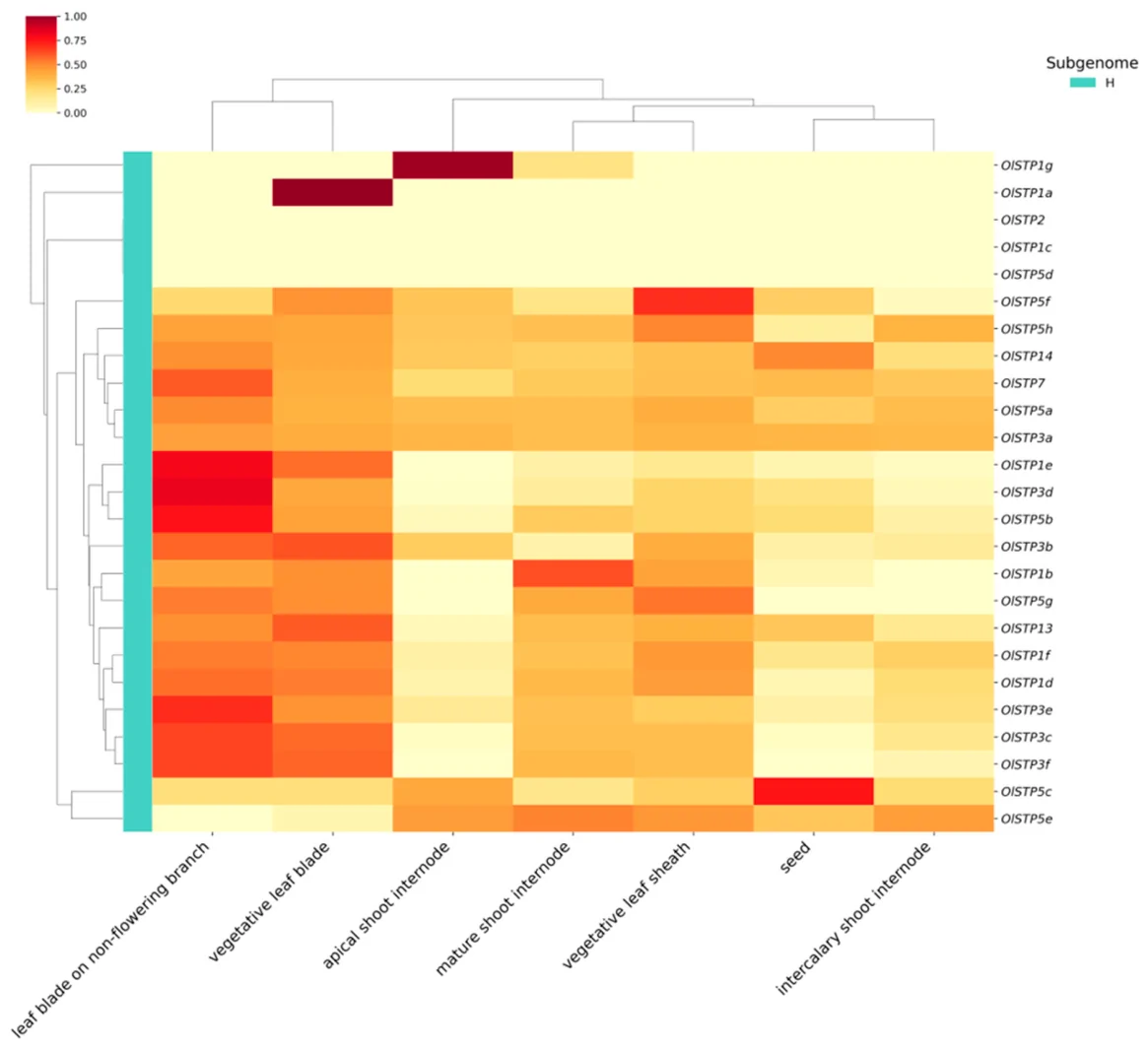
Herbaceous Bamboo, *Ol. latifolia* STP Transcriptome.

**Figure 9:**
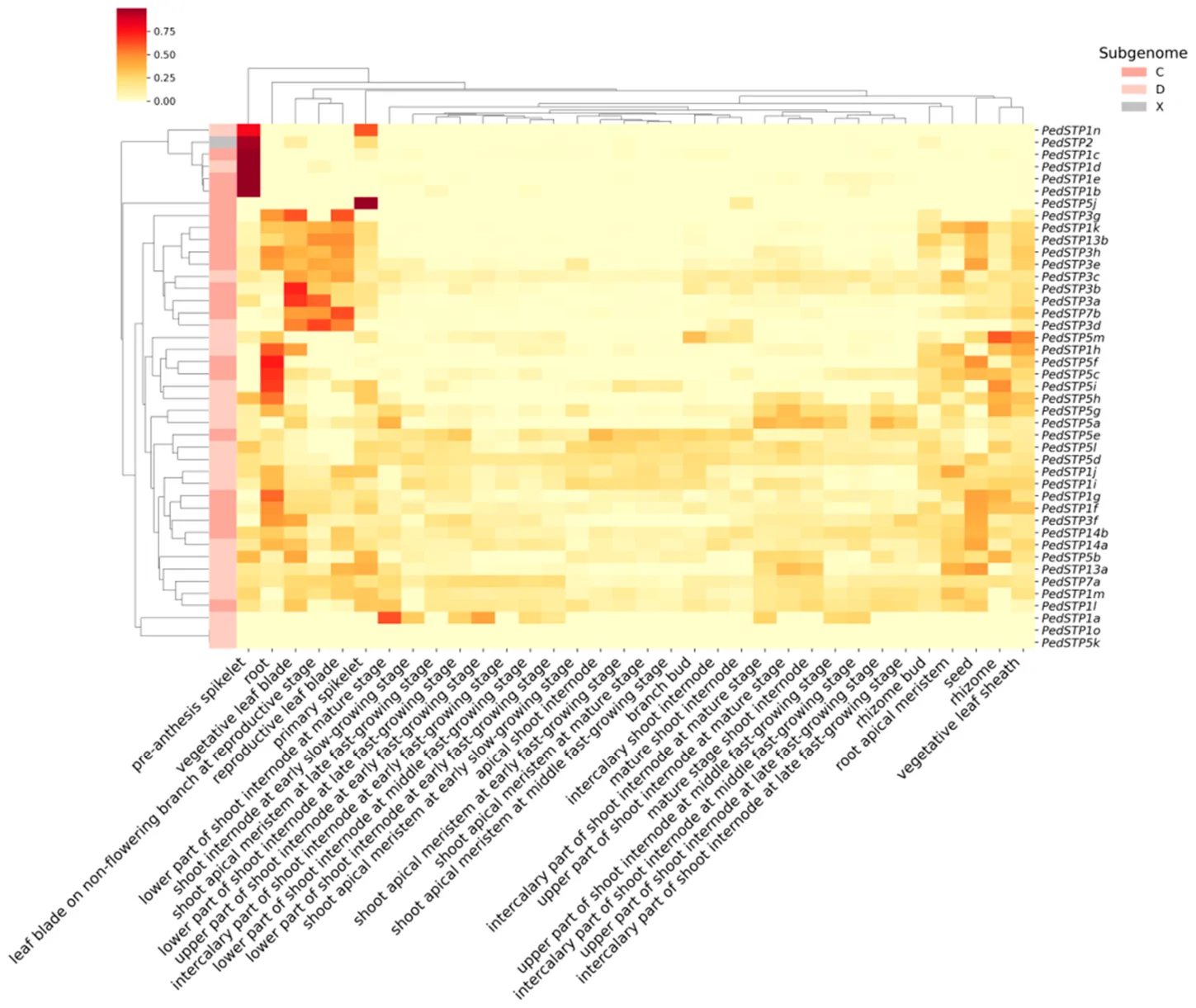
Temperate woody bamboo, *Ph. edulis* STP transcriptome.

**Figure 10:**
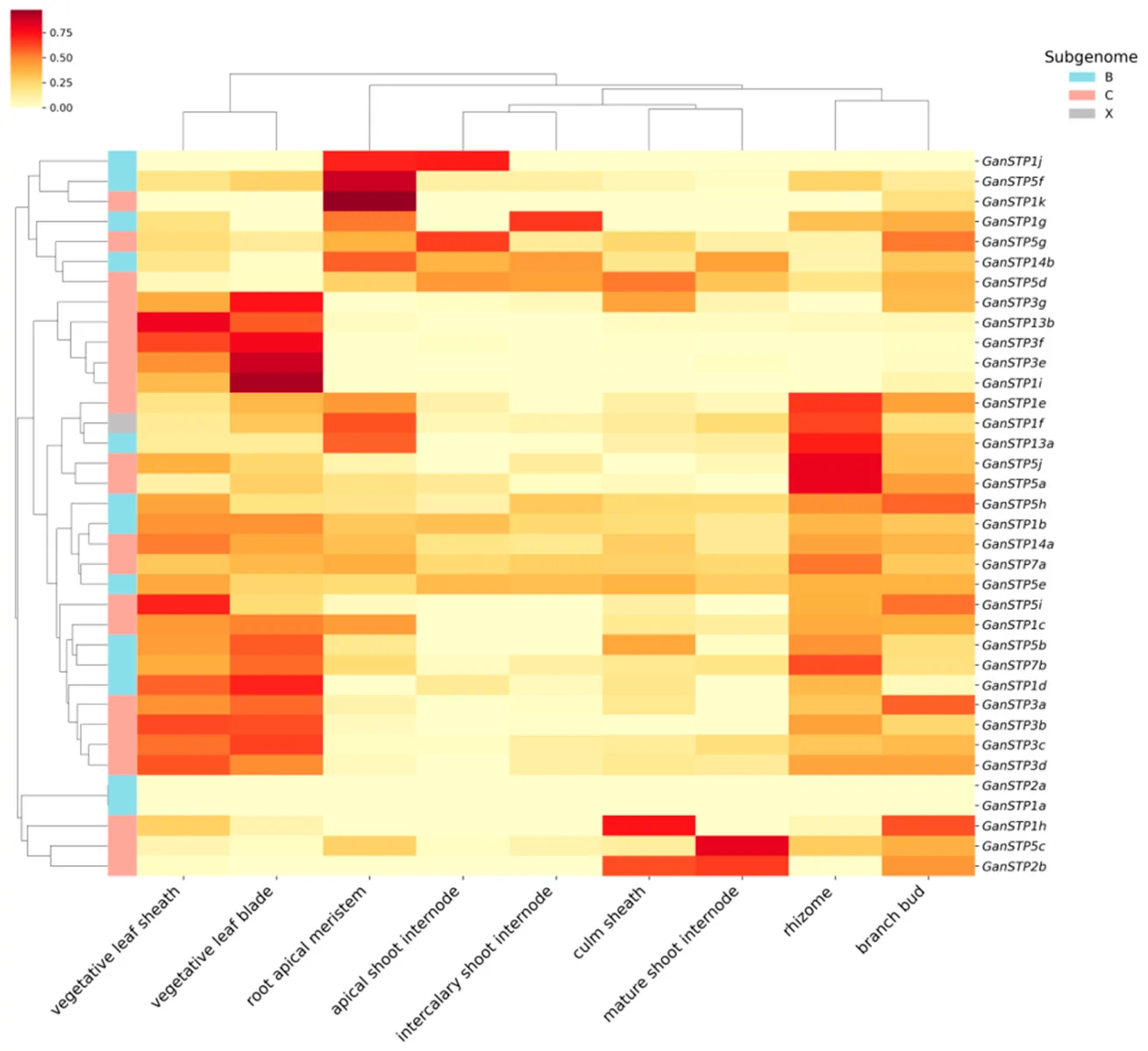
Neotropical woody bamboo, *G. angustifolia* STP transcriptome.

**Figure 11:**
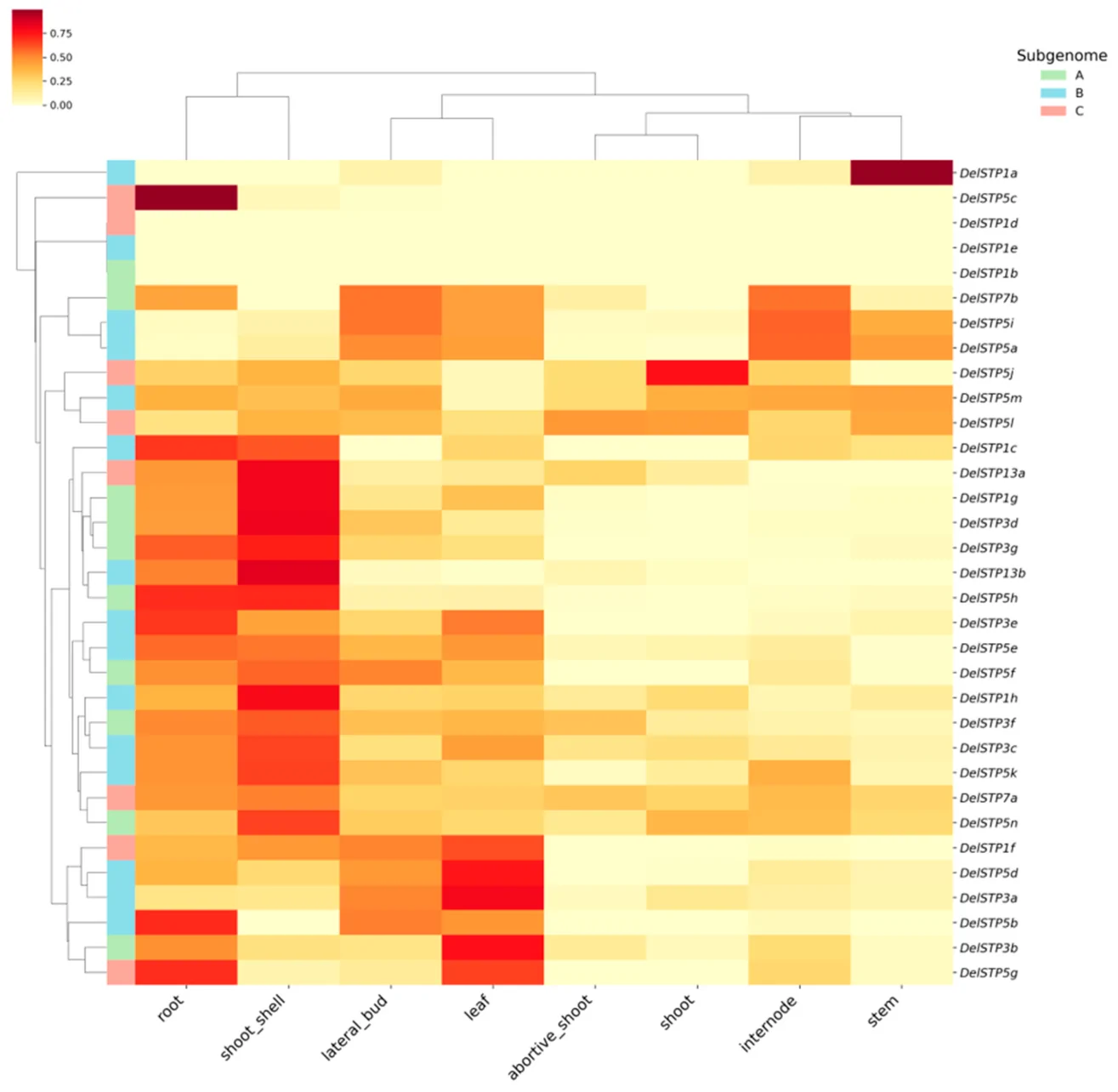
Paleotropical woody bamboo, *D. latiflorus* STP Transcriptome.

In this study, comprehensive expression profiling of SUT and MST genes across four bamboo lineages (Figure 4, Figure 5, Figure 6, Figure 7, Figure 8, Figure 9, Figure 10 and Figure 11) suggested widespread expression asymmetry among homologs from different subgenomes. For instance, in the tetraploid *Ph. edulis*, the homologous pair *PedSUT1a* and *PedSUT1b*, located on different subgenomes, showed significantly different tissue specificities. *PedSUT1a* was enriched in reproductive organs (spikelets and seeds), while *PedSUT1b* functioned primarily in vegetative tissues (rhizomes and roots). Similarly, in the hexaploid *D. latiflorus*, three *DelSTP5* paralogs distributed across subgenomes A, B, and C (*DelSTP5m*, *5g*, *5h*) displayed distinct expression profiles. This specific correspondence between subgenome location and expression profile is suggestive of the subfunctionalization of homologs following WGD, where ancestral expression patterns may have been partitioned among homologs on different subgenomes. Further subgenome localization analysis suggested that in the *G. angustifolia* STP family, although *GanSTP5a* and *GanSTP5c* were both located on chromosome 04C, their expression patterns differed; meanwhile, the homolog *GanSTP5b* on the 04B subgenome exhibited expression characteristics distinct from *GanSTP5a/5c*. This indicates that duplicated genes in bamboo have undergone expression remodeling not only between subgenomes but also within the same subgenome. This multidimensional expression divergence has greatly enriched bamboo’s genetic information, enabling adaptation to complex environmental demands.

Despite the general trend of differentiation, we also observed marked expression redundancy. For example, in *G. angustifolia*, although *GanSUT2a* and *2b* are on different subgenomes, they maintained similar expression patterns; three *GanSTP* genes on chromosome 10C (*GanSTP3g*, *3e* and *3f*) also showed coordinated expression behavior. Similarly, *PedSTP5d* and *5l* (both on chromosome 4D) in *P. edulis* showed convergent expression. This preservation of multi-copy genes in spatiotemporal expression is likely not simple redundancy in expression but is hypothesized to maintain the high efficiency required for sugar transport [68]. Additionally, we observed potential non-functionalization at the transcriptional level. In *Ph. edulis* and *D. latiflorus*, certain STP genes (e.g., *PedSTP1o/5k*, *DelSTP1d/1e/1b*) were undetectable in all tested tissues. The simultaneous absence of detectable expression for three *DelSTP1* homologs (*DelSTP1d*, *1e* and *1b*) across all three subgenomes in our sampled tissues is consistent with transcriptional silencing or expression restricted to specific developmental stages or stress conditions not captured in this study [69].

### 4.4. The Role of SUT and MST Gene Families in Shaping High Bamboo Diversity

As one of the most diverse lineages within the Poaceae, bamboos exhibit remarkable divergence in morphology (herbaceous vs. woody), habitat (tropical vs. temperate), and growth habits. The morphology of ground tissue cells within bamboo internodes plays a crucial role in culm morphogenesis and lignification [70]. Notably, parenchyma cells with distinct morphological structures often perform specialized physiological functions. The ground tissue of woody bamboo culms is composed of long cells and short cells dispersed among them. Long cells are characterized by thick, lignified secondary walls and undergo programmed cell death during culm maturation, whereas short cells possess only thin primary walls and retain vitality throughout the life of the culm [71,72]. This unique dual-cell system necessitates a highly coordinated sugar transport network. Consequently, the expansion and expression diversification of the SUT and MST families identified in this study provide the molecular basis underpinning this complex physiological mechanism.

Woody bamboos exhibit remarkable growth rates, with some species capable of elongating over 1 m per day during the rapid elongation phase. This extraordinary growth rate imposes dual physiological demands: (1) cell wall biosynthesis requiring substantial UDP-glucose for cellulose and hemicellulose synthesis in thickening secondary walls, and (2) osmotic regulation and turgor maintenance needed for rapid cell expansion. We hypothesize that the expansion and expression divergence of specific sugar transporter families observed in this study may have contributed to meeting these demands.

During intensive culm elongation, lignified fiber cells undergo massive secondary cell wall thickening, requiring substantial amounts of cellulose and hemicellulose synthesis. UDP-glucose serves as the primary substrate for cellulose synthase complexes (CESA) in the Golgi apparatus and plasma membrane [73]. Hexose transporters (STP family) deliver glucose from the apoplast or phloem loading zones to developing fiber cells, where hexokinases phosphorylate glucose to glucose-6-phosphate, subsequently converted to UDP-glucose for cell wall polysaccharide biosynthesis [74]. In parallel, rapid cell expansion requires substantial water influx, which is driven by osmotic gradients maintained through vacuolar sugar accumulation. TMT proteins localize to the tonoplast and mediate vacuolar glucose/fructose uptake, playing critical roles in osmotic adjustment and turgor pressure generation during cell expansion [75]. This dual strategy, if functionally validated, could be interpreted as an evolutionary pattern that may have facilitated rapid culm elongation. However, establishing a direct causal link between transporter copy number/expression and growth rate will require transgenic or mutant studies.

Comparative analysis of gene expression patterns between herbaceous and woody lineages highlights an evolutionary strategy where sugar transporter families expanded to facilitate tissue-specific expression differentiation. For instance, within the SUT family, *SUT5* is the predominant gene expressed in the culms of herbaceous bamboos. In woody bamboos, however, WGD-driven expansion of the SUT family has facilitated functional redistribution. *SUT2* and *SUT4* generated numerous homologs that share the functional load of *SUT5* in the culm, exhibiting broad expression patterns. This expression redundancy may help maintain the robustness of sucrose unloading within the massive culms of woody bamboos. The STP family, being the largest of the eight families analyzed, exhibited the most profound expression divergence. While *STP3* and *STP5* genes predominate in herbaceous bamboos, these subfamilies underwent independent expansion in woody bamboos, generating a large number of homologs that likely assumed more specialized functions. This aligns with the findings of Jin et al. [20], who observed that WGD-expanded genes were specifically expressed in bamboo shoots, suggesting these genes acquired novel functions via expression divergence to support rapid shoot growth. These homologs may regulate hexose transport at distinct stages of culm development, potentially contributing to precise carbon allocation between energy utilization and cell wall material formation.

Other MST families exhibited a pattern characterized by complementarity between ubiquitous expression and tissue-specific specialization. In herbaceous bamboos, the ERDL6 and pGlcT families tended to compensate for low gene copy numbers through ubiquitous tissue expression. In contrast, woody bamboos displayed clear tissue-specific specialization. For example, the pGlcT member *GanPGLCT1c* evolved a specific expression pattern restricted to the rhizome and culm sheath. Notably, PMT family genes were identified exclusively in the Ogl and PWB lineages, potentially representing a lineage-specific adaptation. While PMT gene expression was low in the culm, it was extremely high in roots and rhizomes. We hypothesize that within the complex vascular systems of tropical woody bamboos, this gene family may facilitate the transmembrane transport of specific polyols or signaling molecules. Although the observed expression pattern suggests a possible role in rapid culm growth, this hypothesis remains to be experimentally validated. Furthermore, the TMT and INT families maintained broad and stable basal expression patterns across all lineages, likely functioning as transporters essential for maintaining intracellular sugar homeostasis and turgor pressure during rapid growth.

## 5. Conclusions

It is worth noting that genome assembly quality may influence the accurate identification and copy number estimation of gene families. Bamboos are predominantly polyploid species characterized by highly similar subgenomes. During the de novo genome assembly process, such high sequence similarity can occasionally lead to assembly collapse, where highly conserved homeologous or paralogous regions are erroneously merged into a single sequence. Although 13 out of the 14 bamboo genomes analyzed in this study exhibit high assembly completeness (BUSCO scores > 90%) (Table S1), this does not guarantee the correct resolution of all highly similar homeologous regions. The only exception is *Ra. distichophylla*, which has a relatively lower BUSCO score (74.5%). Nevertheless, the key members of these gene families were still successfully identified in this species. Therefore, while the major trends in gene family expansion and expression divergence reported here are likely to reflect genuine biological patterns rather than widespread assembly artifacts, we cannot exclude the possibility that some individual copy numbers are over- or underestimated due to unresolved homeologs. More accurate quantification will require future assemblies with further improved contiguity and phasing of subgenomes.

In this study, we performed systematic genome-wide identification and comparative evolutionary analysis of SUT and MST sugar transporter gene families across 14 bamboo species covering the four major lineages of Bambusoideae. The results indicate that WGD was the primary driving force behind the expansion of sugar transporter gene families in bamboo, resulting in significantly higher gene counts in woody bamboos (tetraploids and hexaploids) compared to herbaceous bamboos (diploids). However, gene retention was not a simple linear accumulation; the extensive gene loss observed in hexaploid Paleotropical woody bamboos supports the hypothesis of genomic dosage balance following polyploidization.

Through in-depth analysis of gene sequences and structures, we found that despite complex reticulate evolution, core members of the SUT and MST families remained highly conserved in physicochemical properties and subcellular localization. Specifically, the identification of multiple groups of cross-species identical protein sequences within the paleotropical woody bamboo lineage suggests that key sugar transporters underwent purifying selection during evolution to maintain stability in their basal functions of sugar loading and unloading.

Transcriptomic and synteny analyses further suggested the mechanisms of expression divergence in duplicated genes following polyploidization. Homologous genes exhibited marked expression asymmetry across different subgenomes. This subgenome-biased expression and tissue-specific differentiation (e.g., the fine-tuned division of labor of SUT and STP families across different developmental stages of woody bamboo culms) constitute an efficient carbon allocation network enabling high biomass accumulation and rapid elongation in woody bamboos. In contrast, herbaceous bamboos relied more on the ubiquitous expression of a few genes. Furthermore, the lineage-specific distribution of families like PMT suggests that different bamboo species may have evolved unique sugar transport strategies to adapt to specific habitats.

In summary, this study is the first to elucidate the evolutionary trajectory of the bamboo sugar transport system within a cross-lineage polyploidization framework, clarifying the evolutionary pattern of “WGD-driven expansion—dosage balance screening—subgenome expression divergence.” This not only provides critical molecular evidence for understanding the unique rapid growth mechanism of woody bamboos but also offers valuable candidate gene resources and a theoretical basis for source-sink regulation and yield improvement in Poaceae crops.

## Figures and Tables

**Table 1:** Highly conserved genes in bamboo species.

SUT	Gene1	Gene2		
1	*DelSUT2c*	*DhaSUT2b*		
2	*DelSUT1b*	*DelSUT1c*		
ERDL6	Gene1	Gene2	Gene3	
1	*DsiSFP9a*	*DhaSFP9b*	*DelSFP9b*	
2	*DelSFP14*	*DsiSFP14*		
pGlcT	Gene1	Gene2	Gene3	
1	*DhapGlcT1a*	*BampGlcT1a*		
2	*DsipGlcT3b*	*DhapGlcT3c*		
3	*PedpGlcT1a*	*AlupGlcT1b*		
STP	Gene1	Gene2	Gene3	Gene4
1	*DsiSTP5f*	*DhaSTP5b*	*DelSTP5m*	
2	*DsiSTP5d*	*DhaSTP5m*		
3	*DsiSTP14a*	*DhaSTP14a*		
4	*DelSTP3f*	*DhaSTP3f*		
5	*DsiSTP2a*	*DhaSTP2c*		
6	*DsiSTP1k*	*DhaSTP1b*	*DelSTP1e*	
7	*DelSTP3a*	*DhaSTP3b*		
8	*DelSTP1d*	*DhaSTP1a*		
9	*DhaSTP1h*	*DhaSTP1i*		
10	*DsiSTP14b*	*DhaSTP14c*		
11	*DelSTP5a*	*DsiSTP5a*		
12	*DelSTP13a*	*DsiSTP13a*		
13	*DelSTP1f*	*DsiSTP1d*		
TMT	Gene1	Gene2		
1	*DelTMT3f*	*DsiTMT1c*		
PMT				
No genes				
INT				
No genes				
VGT				
No genes				

## Data Availability

Genome and transcriptome data sources are described in Materials and Methods 4.1 and 4.7. All derived gene lists, HMM seed sequences, sequence alignments, phylogenetic trees, duplication classification outputs, Ka/Ks gene-pair lists and values, and processed expression matrices generated in this study are included in the Supplementary Materials (Supplementary Data S1–S8, Supplementary Figures S1–S104, Tables S1–S7). Analysis scripts are available from the corresponding author upon reasonable request.

## Supplementary Materials

The following supporting information can be downloaded at: https://www.mdpi.com/article/10.3390/biology15181614/s1. Figure S1: Physicochemical properties of SUT gene family proteins in 14 bamboo species; Figure S2: Physicochemical properties of ERDL6 gene family proteins in 14 bamboo species; Figure S3: Physicochemical properties of INT gene family proteins in 14 bamboo species; Figure S4: Physicochemical properties of pGlcT gene family proteins in 14 bamboo species; Figure S5: Physicochemical properties of PMT gene family proteins in 14 bamboo species; Figure S6: Physicochemical properties of STP gene family proteins in 14 bamboo species; Figure S7: Physicochemical properties of TMT gene family proteins in 14 bamboo species; Figure S8: Physicochemical properties of VGT gene family proteins in 14 bamboo species; Figure S9: Secondary structure of SUT gene family proteins in 14 bamboo species; Figure S10: Secondary structure of ERDL6 gene family proteins in 14 bamboo species; Figure S11: Secondary structure of INT gene family proteins in 14 bamboo species; Figure S12: Secondary structure of pGlcT gene family proteins in 14 bamboo species; Figure S13: Secondary structure of PMT gene family proteins in 14 bamboo species; Figure S14: Secondary structure of STP gene family proteins in 14 bamboo species; Figure S15: Secondary structure of TMT gene family proteins in 14 bamboo species; Figure S16: Secondary structure of VGT gene family proteins in 14 bamboo species; Figure S17: Subcellular localization prediction of SUT gene family proteins in 14 bamboo species; Figure S18: Subcellular localization prediction of ERDL6 gene family proteins in 14 bamboo species; Figure S19: Subcellular localization prediction of INT gene family proteins in 14 bamboo species; Figure S20: Subcellular localization prediction of pGlcT gene family proteins in 14 bamboo species; Figure S21: Subcellular localization prediction of PMT gene family proteins in 14 bamboo species; Figure S22: Subcellular localization prediction of STP gene family proteins in A. luodianensis; Figure S23: Subcellular localization prediction of STP gene family proteins in B. amplexicaulis; Figure S24: Subcellular localization prediction of STP gene family proteins in D. latiflorus; Figure S25: Subcellular localization prediction of STP gene family proteins in D. brandisii; Figure S26: Subcellular localization prediction of STP gene family proteins in D. sinicus; Figure S27: Subcellular localization prediction of STP gene family proteins in G. angustifolia; Figure S28: Subcellular localization prediction of STP gene family proteins in H. calcarean; Figure S29: Subcellular localization prediction of STP gene family proteins in M. baccifera; Figure S30: Subcellular localization prediction of STP gene family proteins in Ot. Glauca; Figure S31: Subcellular localization prediction of STP gene family proteins in Ol. Latifolia; Figure S32: Subcellular localization prediction of STP gene family proteins in Ph. Edulis; Figure S33: Subcellular localization prediction of STP gene family proteins in Ra. Distichophylla; Figure S34: Subcellular localization prediction of STP gene family proteins in Ra. Guianensis; Figure S35: Subcellular localization prediction of STP gene family proteins in Rh. Racemiflorum; Figure S36: Subcellular localization prediction of TMT gene family proteins in 14 bamboo species; Figure S37: Subcellular localization prediction of VGT gene family proteins in 14 bamboo species; Figure S38: Phylogenetic tree of the ERDL6 gene family; Figure S39: Phylogenetic tree of the INT gene family; Figure S40: Phylogenetic tree of the pGlcT gene family; Figure S41: Phylogenetic tree of the PMT gene family; Figure S42: Phylogenetic tree of the STP gene family; Figure S43: Phylogenetic tree of the TMT gene family; Figure S44: Phylogenetic tree of the VGT gene family; Figure S45: Chromosomal location and synteny of the SUT gene family in diploid herbaceous bamboos; Figure S46: Chromosomal location and synteny of the SUT gene family in tetraploid temperate woody bamboos; Figure S47: Chromosomal location and synteny of the SUT gene family in tetraploid neotropical woody bamboos; Figure S48: Chromosomal location and synteny of the SUT gene family in hexaploid paleotropical woody bamboos; Figure S49: Chromosomal location and synteny of the ERDL6 gene family in diploid herbaceous bamboos; Figure S50: Chromosomal location and synteny of the ERDL6 gene family in tetraploid temperate woody bamboos; Figure S51: Chromosomal location and synteny of the ERDL6 gene family in tetraploid neotropical woody bamboos; Figure S52: Chromosomal location and synteny of the ERDL6 gene family in hexaploid paleotropical woody bamboos; Figure S53: Chromosomal location and synteny of the INT gene family in diploid herbaceous bamboos; Figure S54: Chromosomal location and synteny of the INT gene family in tetraploid temperate woody bamboos; Figure S55: Chromosomal location and synteny of the INT gene family in tetraploid neotropical woody bamboos; Figure S56: Chromosomal location and synteny of the INT gene family in hexaploid paleotropical woody bamboos; Figure S57: Chromosomal location and synteny of the pGlcT gene family in diploid herbaceous bamboos; Figure S58: Chromosomal location and synteny of the pGlcT gene family in tetraploid temperate woody bamboos; Figure S59: Chromosomal location and synteny of the pGlcT gene family in tetraploid neotropical woody bamboos; Figure S60: Chromosomal location and synteny of the pGlcT gene family in hexaploid paleotropical woody bamboos; Figure S61: Chromosomal location and synteny of the PMT gene family in tetraploid neotropical woody bamboos; Figure S62: Chromosomal location and synteny of the PMT gene family in hexaploid paleotropical woody bamboos; Figure S63: Chromosomal location and synteny of the STP gene family in diploid herbaceous bamboos; Figure S64: Chromosomal location and synteny of the STP gene family in tetraploid temperate woody bamboos; Figure S65: Chromosomal location and synteny of the STP gene family in tetraploid neotropical woody bamboos; Figure S66: Chromosomal location and synteny of the STP gene family in hexaploid paleotropical woody bamboos; Figure S67: Chromosomal location and synteny of the TMT gene family in diploid herbaceous bamboos; Figure S68: Chromosomal location and synteny of the TMT gene family in tetraploid temperate woody bamboos; Figure S69: Chromosomal location and synteny of the TMT gene family in tetraploid neotropical woody bamboos; Figure S70: Chromosomal location and synteny of the TMT gene family in hexaploid paleotropical woody bamboos; Figure S71: Chromosomal location and synteny of the VGT gene family in diploid herbaceous bamboos; Figure S72: Chromosomal location and synteny of the VGT gene family in tetraploid temperate woody bamboos; Figure S73: Chromosomal location and synteny of the VGT gene family in tetraploid neotropical woody bamboos; Figure S74: Chromosomal location and synteny of the VGT gene family in hexaploid paleotropical woody bamboos; Figure S75: Diploid Herbaceous Bamboo SUT Transcriptome; Figure S76: Tetraploid temperate woody bamboo SUT Transcriptome; Figure S77: Tetraploid neotropical woody bamboos SUT Transcriptome; Figure S78: Hexaploid paleotropical woody bamboos SUT Transcriptome; Figure S79: Diploid Herbaceous Bamboo ERDL6 Transcriptome; Figure S80: Tetraploid temperate woody bamboo ERDL6 Transcriptome; Figure S81: Tetraploid neotropical woody bamboos ERDL6 Transcriptome; Figure S82: Hexaploid paleotropical woody bamboos ERDL6 Transcriptome; Figure S83: Diploid Herbaceous Bamboo INT Transcriptome; Figure S84: Tetraploid temperate woody bamboo INT Transcriptome; Figure S85: Tetraploid neotropical woody bamboos INT Transcriptome; Figure S86: Hexaploid paleotropical woody bamboos INT Transcriptome; Figure S87: Diploid Herbaceous Bamboo pGlcT Transcriptome; Figure S88: Tetraploid temperate woody bamboo pGlcT Transcriptome; Figure S89: Tetraploid neotropical woody bamboos pGlcT Transcriptome; Figure S90: Hexaploid paleotropical woody bamboos pGlcT Transcriptome; Figure S91: Tetraploid neotropical woody bamboos PMT Transcriptome; Figure S92: Hexaploid paleotropical woody bamboos PMT Transcriptome; Figure S93: Diploid Herbaceous Bamboo STP Transcriptome; Figure S94: Tetraploid temperate woody bamboo STP Transcriptome; Figure S95: Tetraploid neotropical woody bamboos STP Transcriptome; Figure S96: Hexaploid paleotropical woody bamboos STP Transcriptome; Figure S97: Diploid Herbaceous Bamboo TMT Transcriptome; Figure S98: Tetraploid temperate woody bamboo TMT Transcriptome; Figure S99: Tetraploid neotropical woody bamboos TMT Transcriptome; Figure S100: Hexaploid paleotropical woody bamboos TMT Transcriptome; Figure S101: Diploid Herbaceous Bamboo VGT Transcriptome; Figure S102: Tetraploid temperate woody bamboo VGT Transcriptome; Figure S103: Tetraploid neotropical woody bamboos VGT Transcriptome; Figure S104: Hexaploid paleotropical woody bamboos VGT Transcriptome; Table S1: Summary of genome assembly statis-tics for the 14 bamboo species used in this study; Table S2: Copy number distribution of SUT and MST gene families across 14 bamboo species with their lineage and ploidy information; Table S3: Summary of gene duplication types for eight sugar transporter families across 14 bamboo species; Table S4: Ka/Ks values for orthologous sugar transporter gene pairs between Ol. lati-folia and other bamboo species; Table S5: Ka/Ks values for within-species paralogs of sugar transporter genes in four bam-boo genomes; Table S6: RNA-seq sample information for 12 bamboo species; Table S7: Unified nomenclature of SUT and MST genes in 14 bamboo genomes. Data S1: 21_groups_of_identical_protein_sequences. CDS sequences of 21 groups of identical pro-teins; Data S2: Gene_family_sequences. Protein sequences of 8 gene families from 14 bamboo species; Data S3: HMM_seed. Seed sequences for hmmsearch constructed based on rice and Arabidopsis data; Data S4: Phylogenetic_tree. Phylogenetic tree of 8 gene families from 14 bamboo species; Data S5: Expression_matrix. Expression matrix of 8 gene families from 14 bamboo species; Data S6: MCScanX. Gene duplication types of 8 gene families across 14 bamboo species; Data S7: kaks-Paralogous. KaKs results of paralogous genes from 8 gene families across 4 bamboo species. Data S8: ksks-Orthologous. KaKs results of Orthologous genes from 8 gene families across 4 bamboo species.

## Abbreviations

The following abbreviations are used in this manuscript:

WGD	whole-genome duplication
HB	herbaceous bamboos
NWB	neotropical woody bamboos
PWB	paleotropical woody bamboos
TWB	temperate woody bamboos
Alu	*Ampelocalamus luodianensis*
Bam	*Bonia amplexicaulis*
Dha	*Dendrocalamus brandisii*
Del	*Dendrocalamus latiflorus*
Dsi	*Dendrocalamus sinicus*
Gan	*Guadua angustifolia*
Hca	*Hsuehochloa calcarea*
Mhu	*Melocanna baccifera*
Ol	*Olyra latifolia*
Ogl	*Otatea glauca*
Ped	*Phyllostachys edulis*
Rdi	*Raddia distichophylla*
Rgu	*Raddia guianensis*
Rhi	*Rhipidocladum racemiflorum*
MSTs	Monosaccharide Transporters
SUTs	Sucrose Transporters
MFS	Major Facilitator Superfamily
HMM	Hidden Markov Model
