# Supplementary material for: Genomic Expansion and Subgenome Expression Divergence of Sugar Transporters During Polyploid Evolution of Bamboos
Source: Biology (Basel). 2026 Sep 13;15(18):1614. doi: 10.3390/biology15181614 (PMC13603930; doi:10.3390/biology15181614)
Supplement: Supplementary file 1 [file biology-15-01614-s001.zip › supplementary figure and table/Supplementary Figures S1-104.pdf]

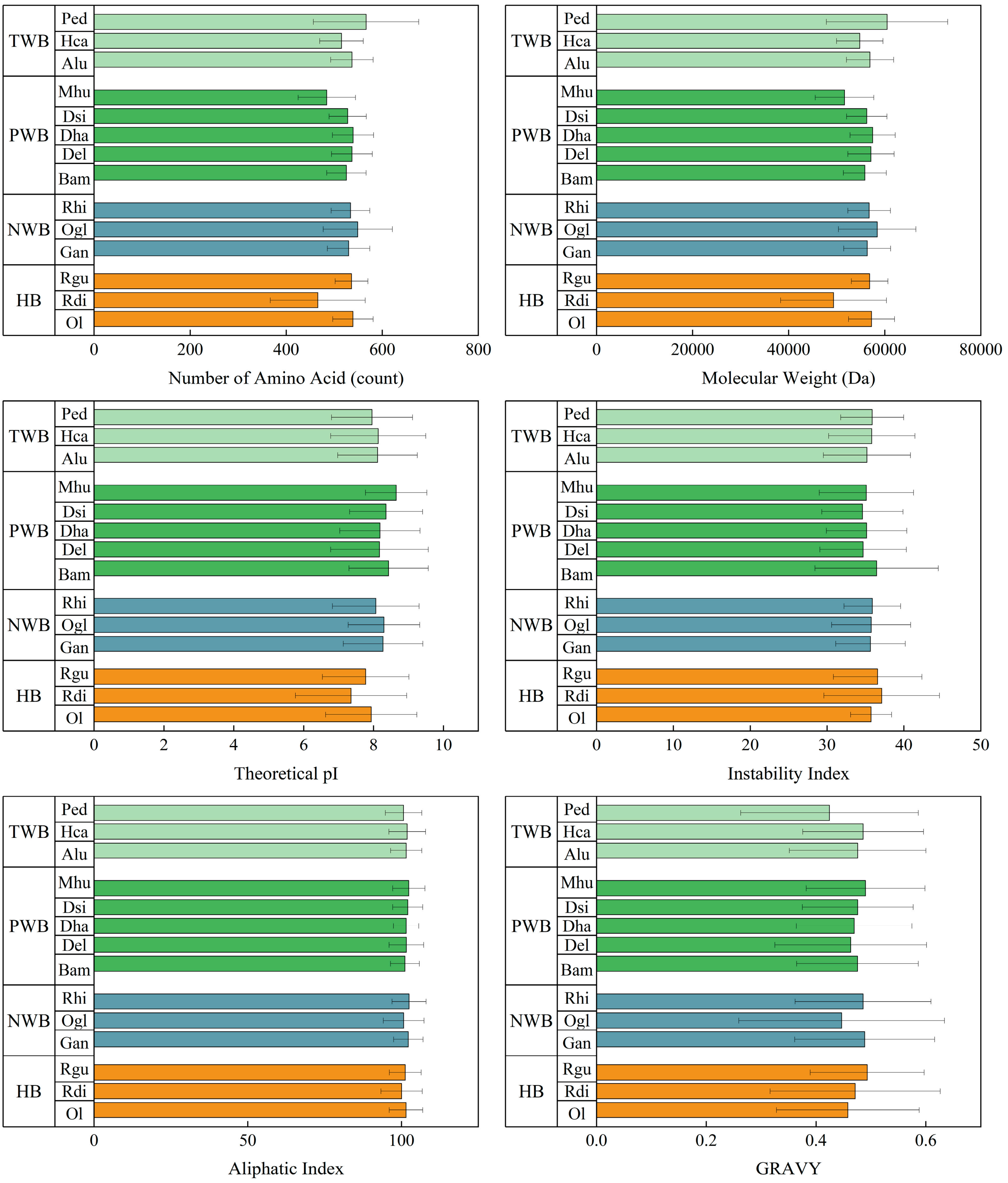

Supplementary Figure S1 Physicochemical properties of SUT gene family proteins in 14 bamboo species

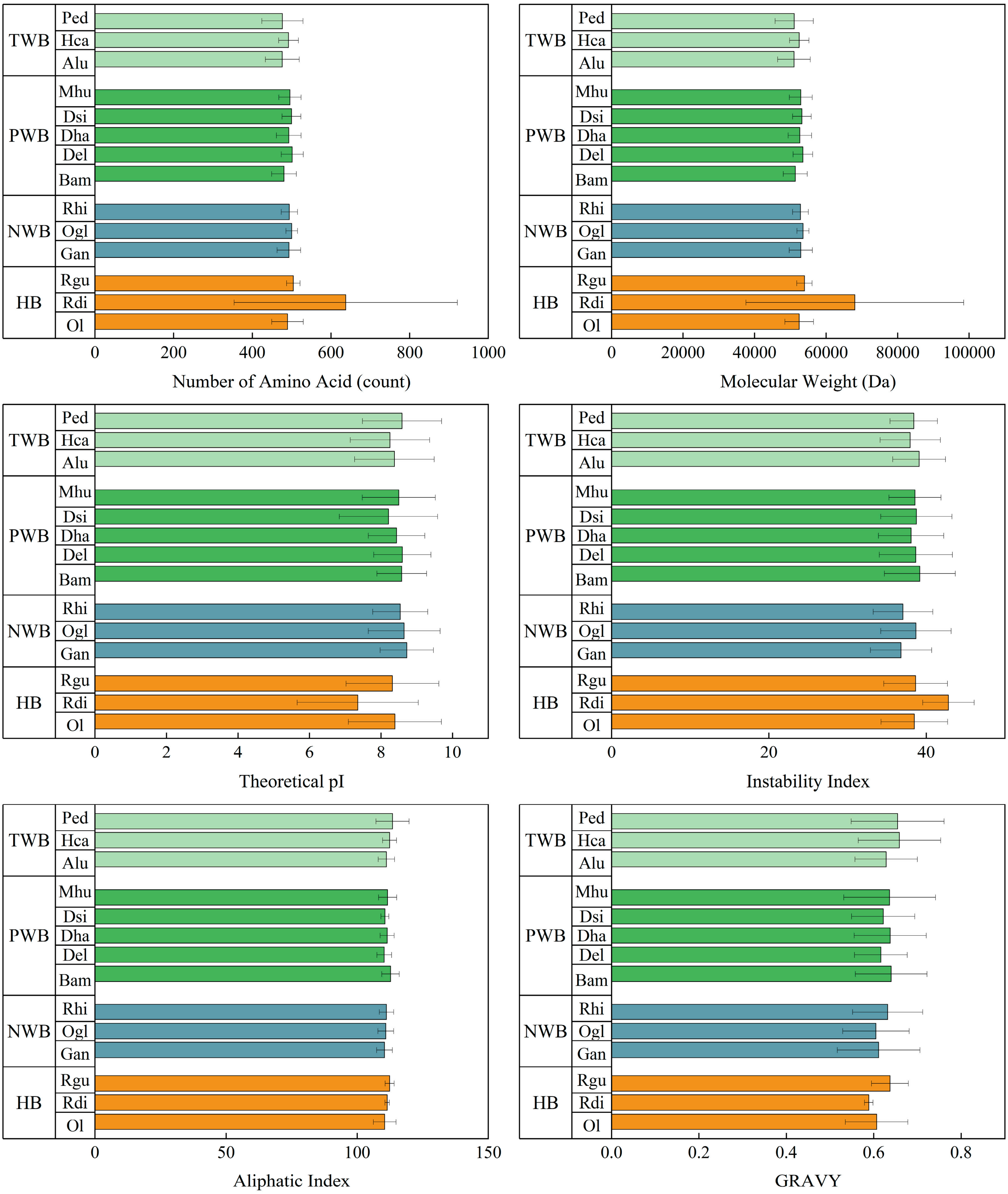

Supplementary Figure S2 Physicochemical properties of ERDL6 gene family proteins in 14 bamboo species

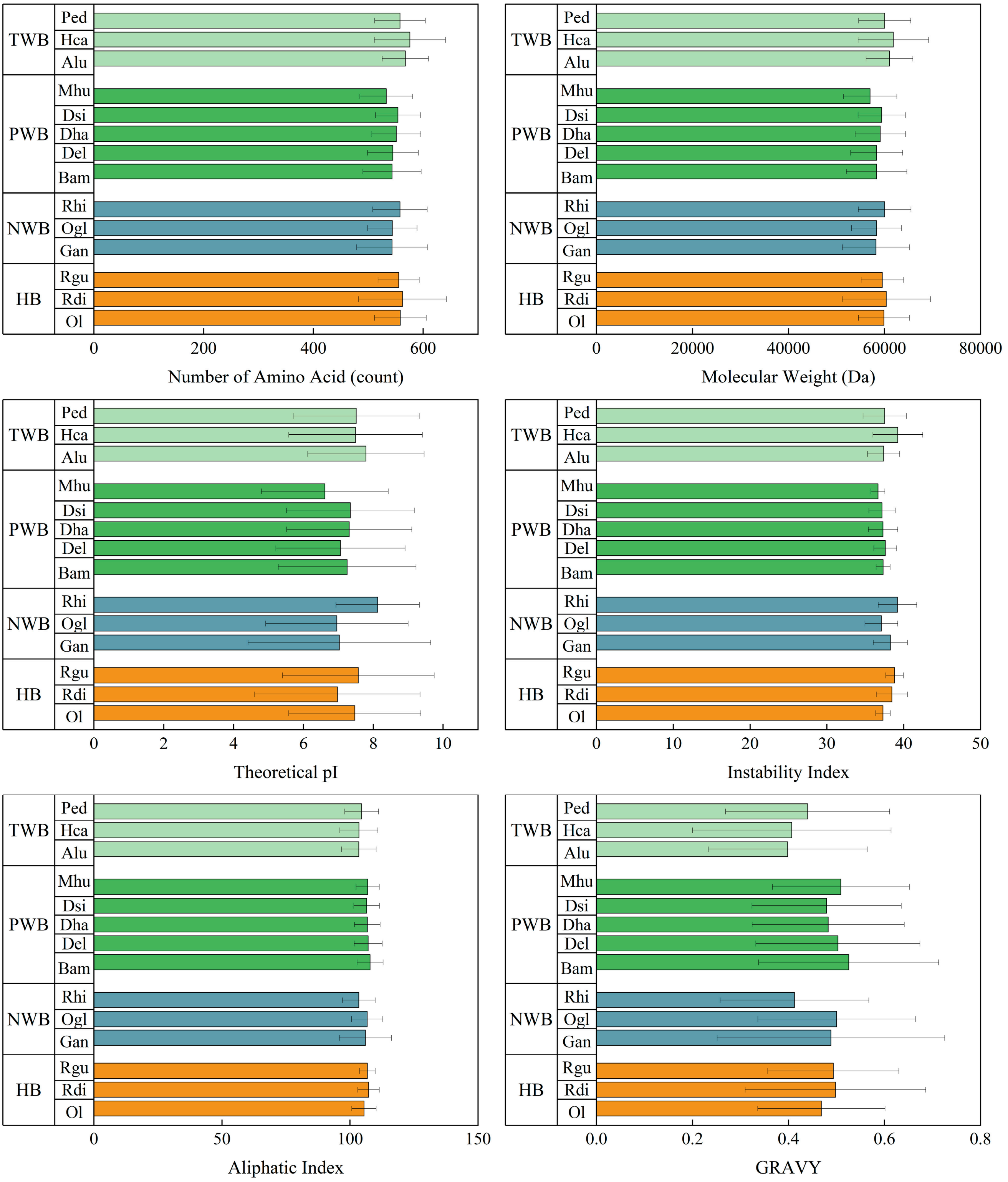

Supplementary Figure S3 Physicochemical properties of INT gene family proteins in 14 bamboo species

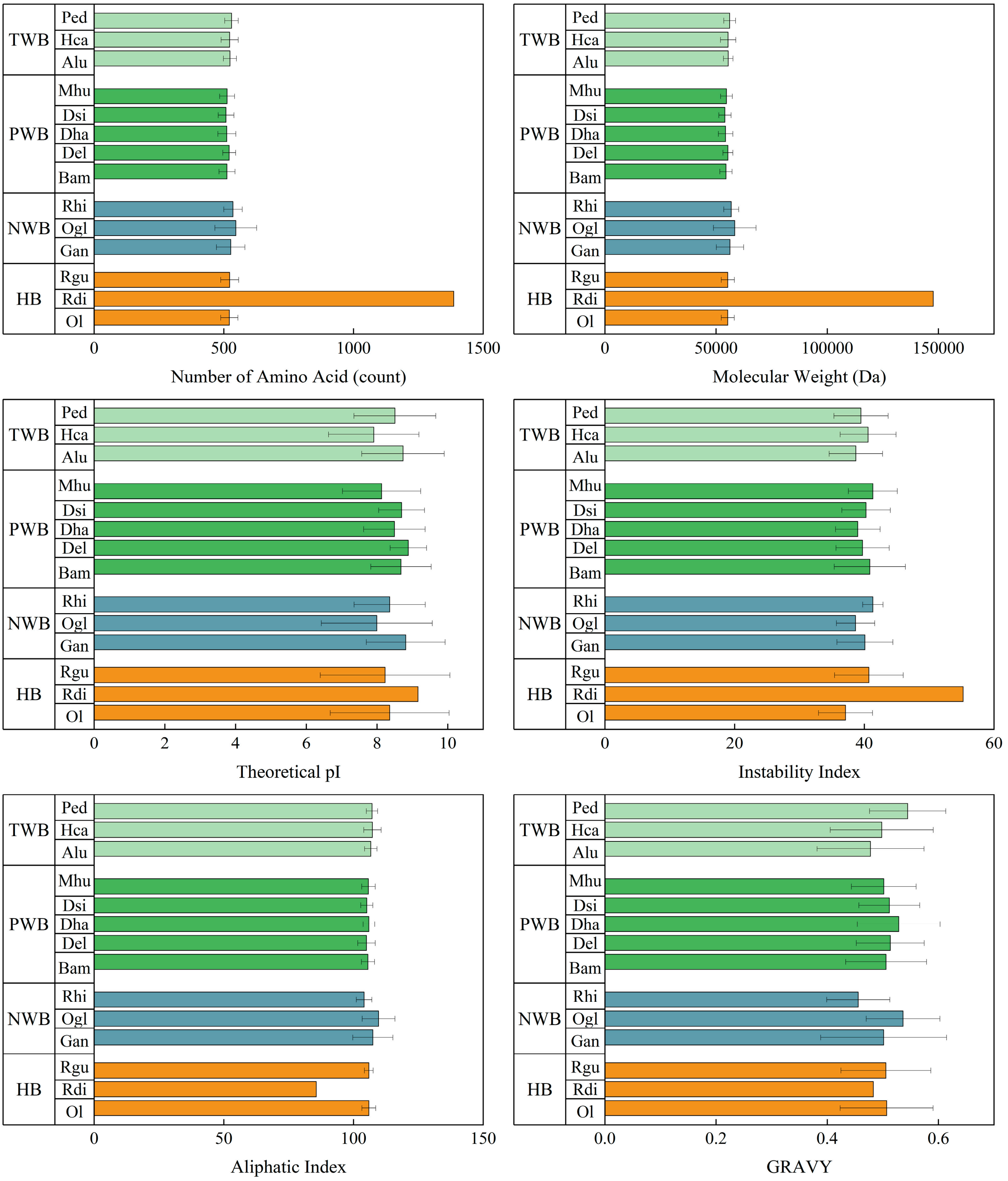

Supplementary Figure S4 Physicochemical properties of pGlcT gene family proteins in 14 bamboo species

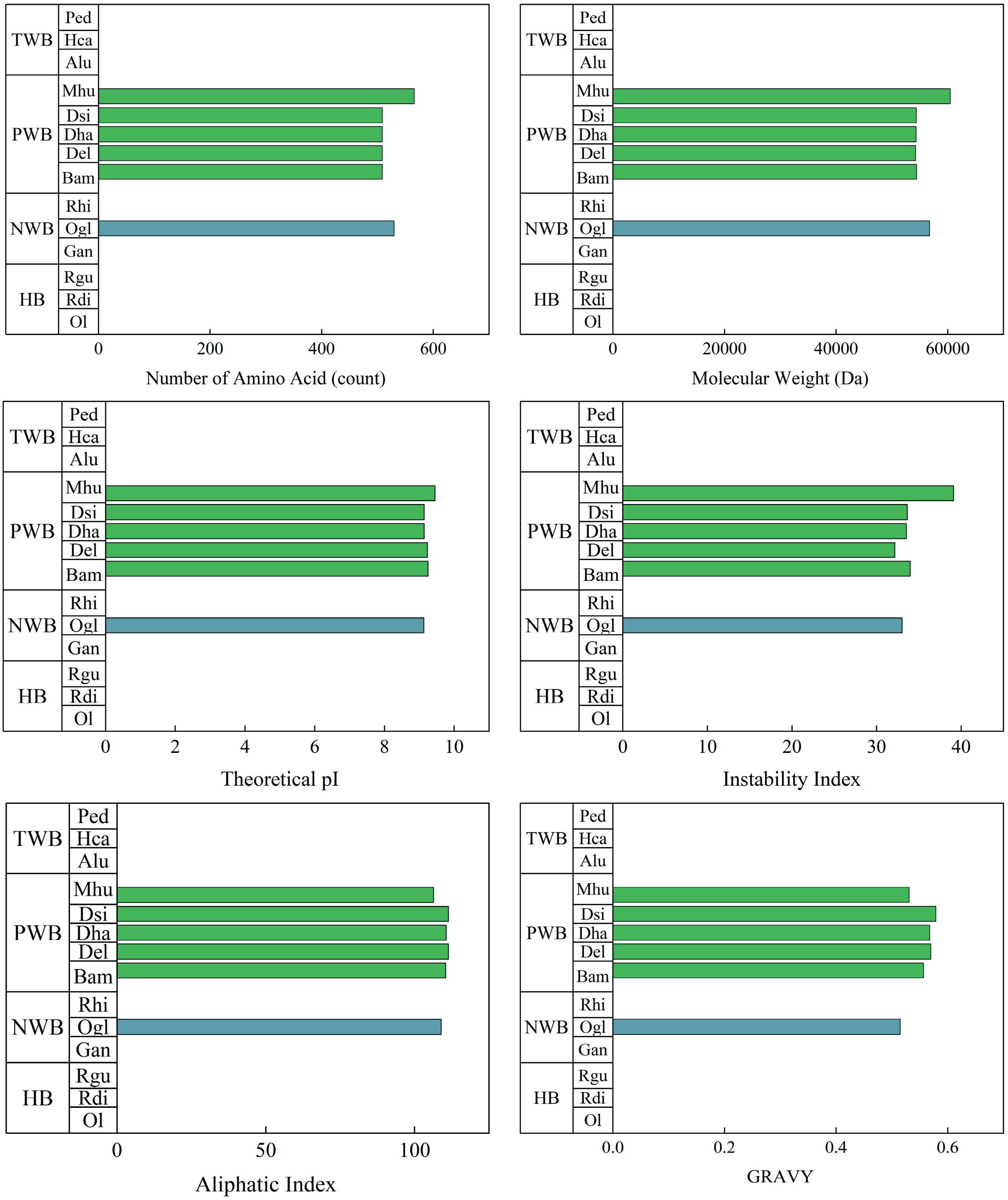

Supplementary Figure S5 Physicochemical properties of PMT gene family proteins in 14 bamboo species

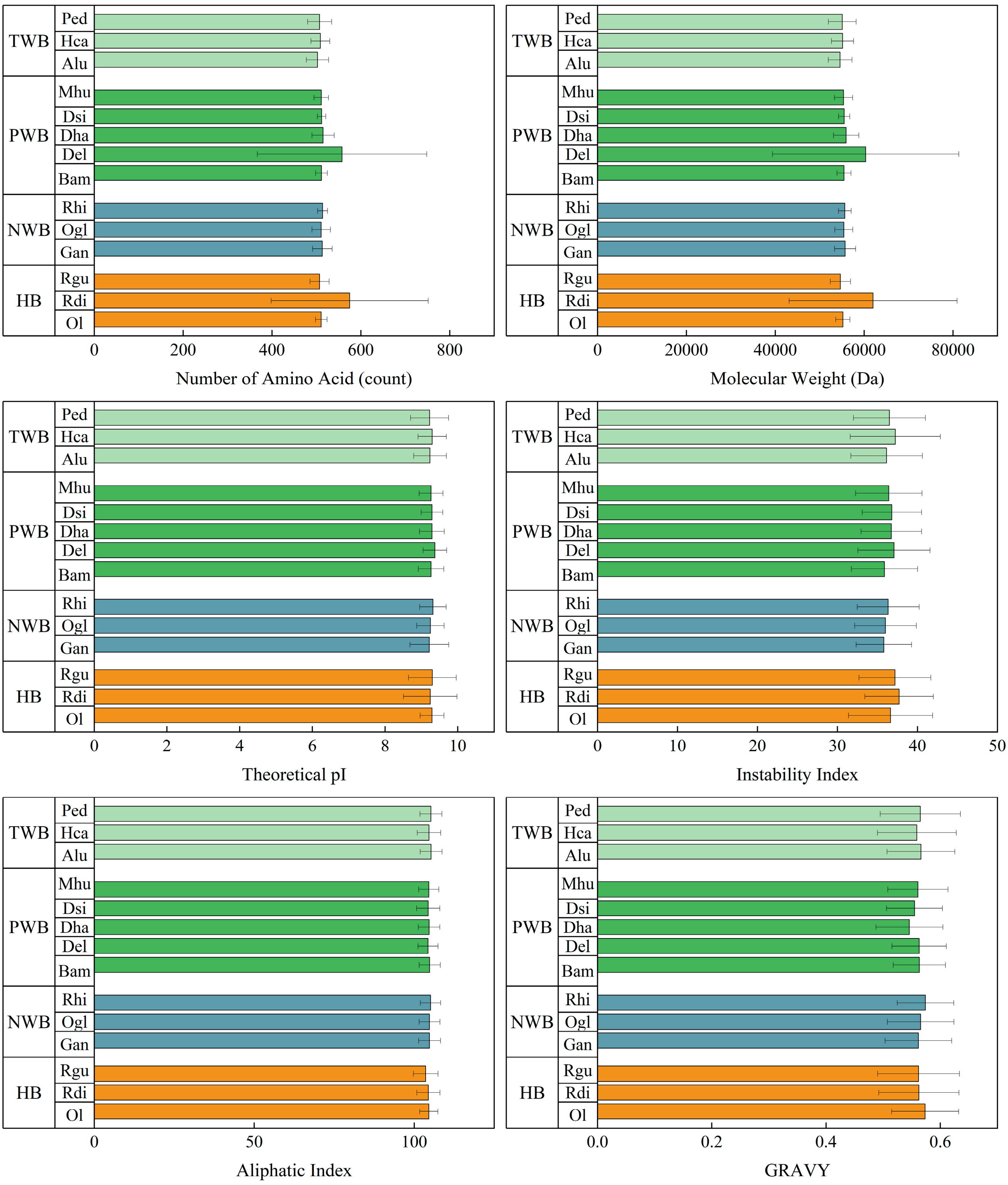

Supplementary Figure S6 Physicochemical properties of STP gene family proteins in 14 bamboo species

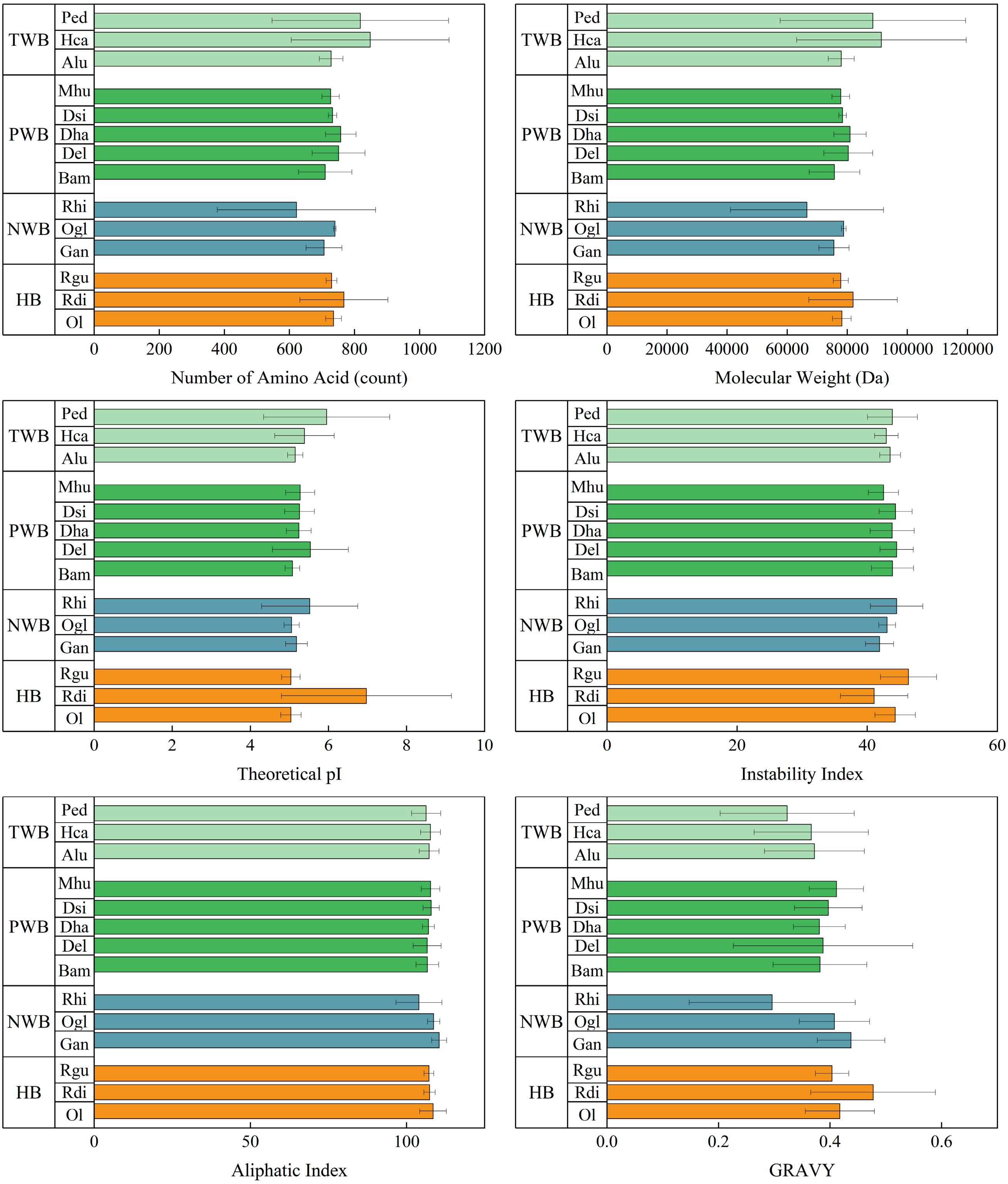

Supplementary Figure S7 Physicochemical properties of TMT gene family proteins in 14 bamboo species

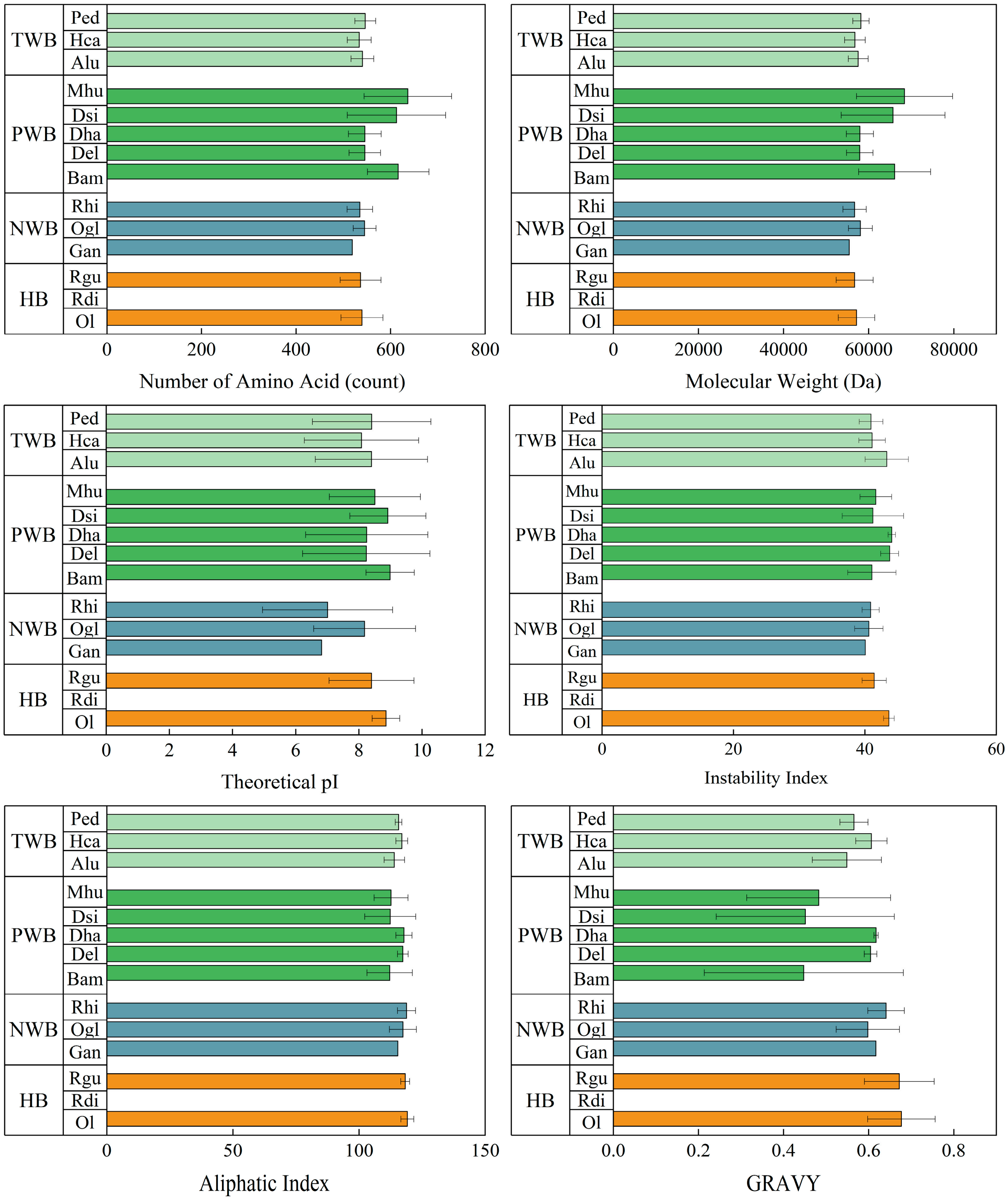

Supplementary Figure S8 Physicochemical properties of VGT gene family proteins in 14 bamboo species

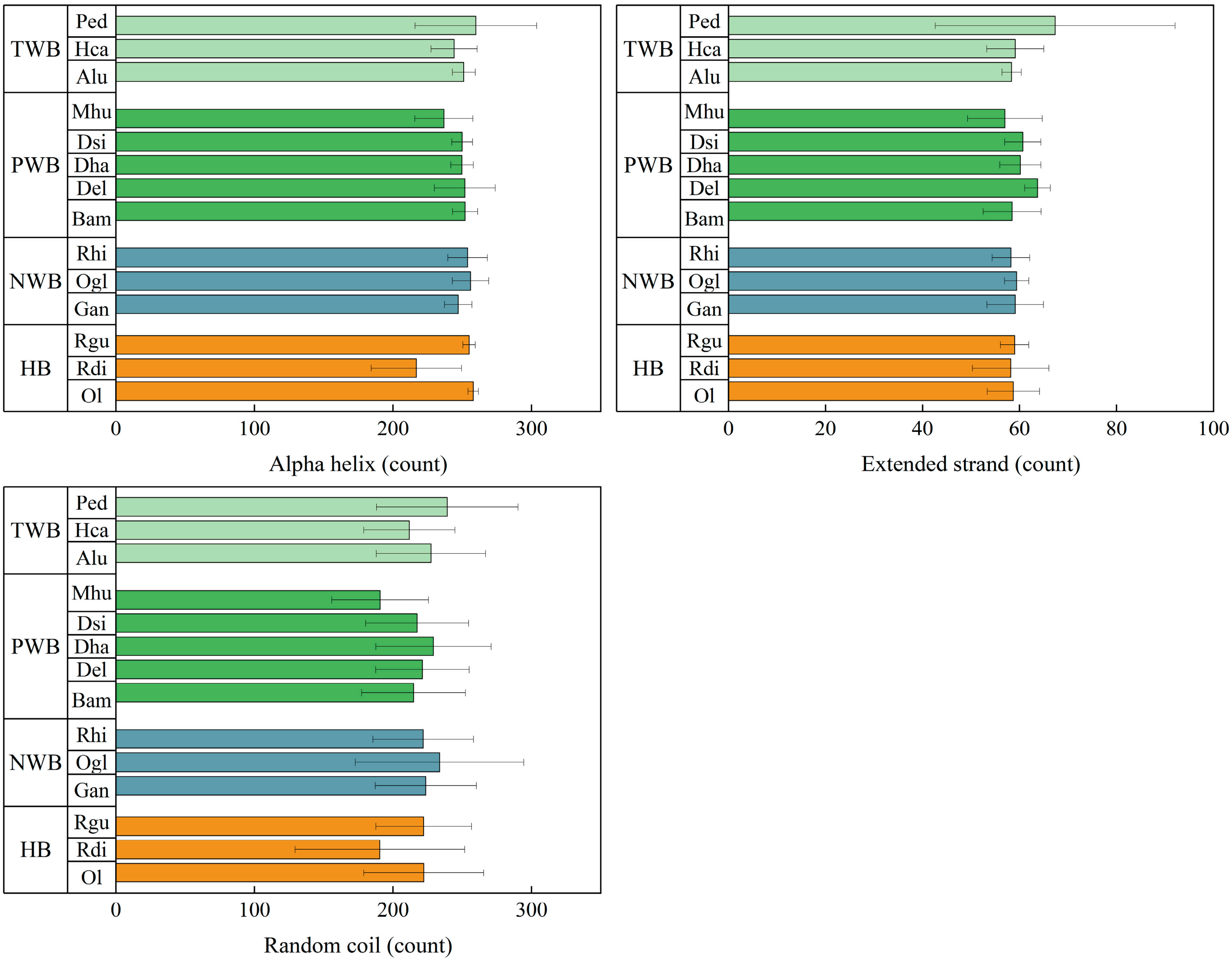

Supplementary Figure S9 Secondary structure of SUT gene family proteins in 14 bamboo species

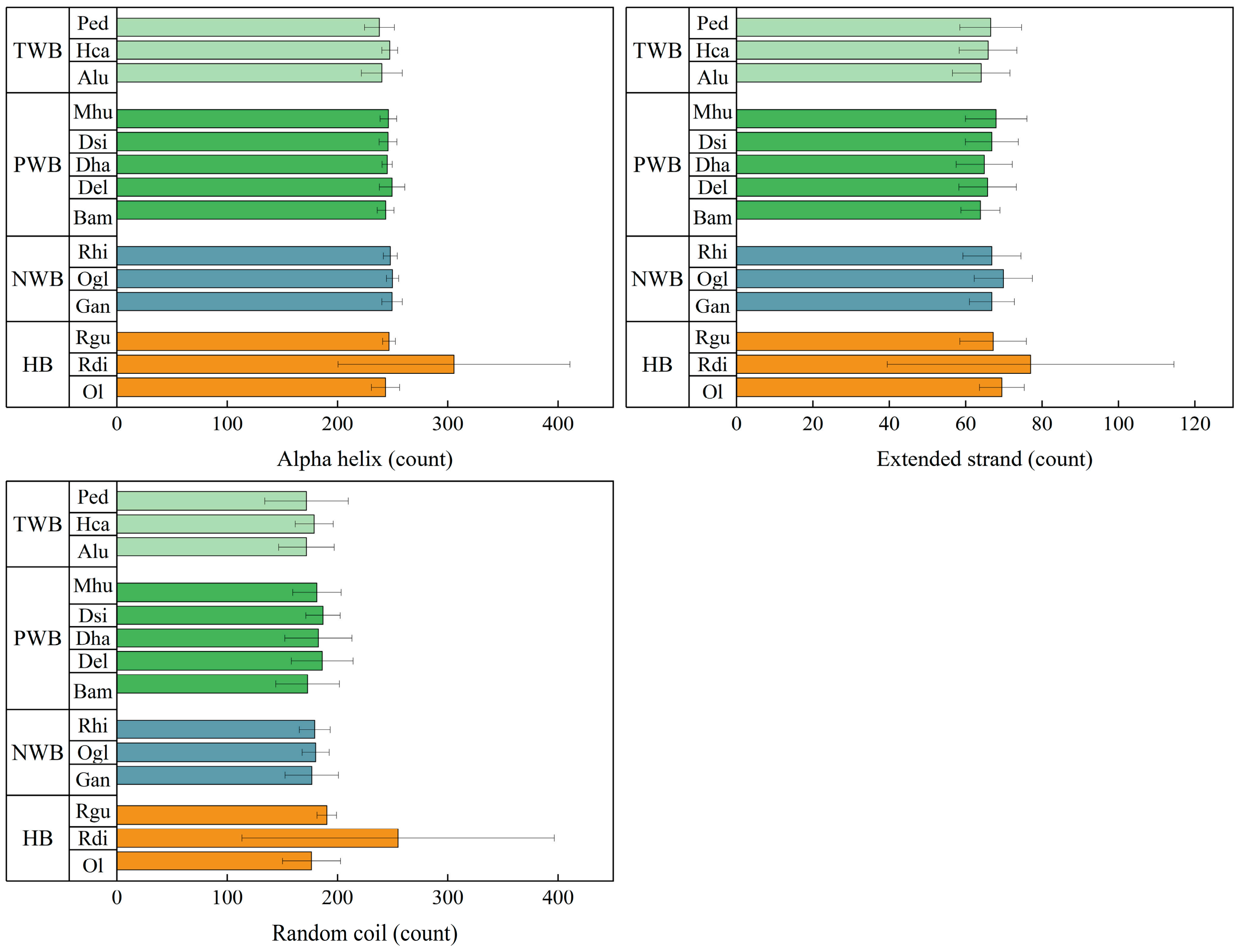

Supplementary Figure S10 Secondary structure of ERDL6 gene family proteins in 14 bamboo species

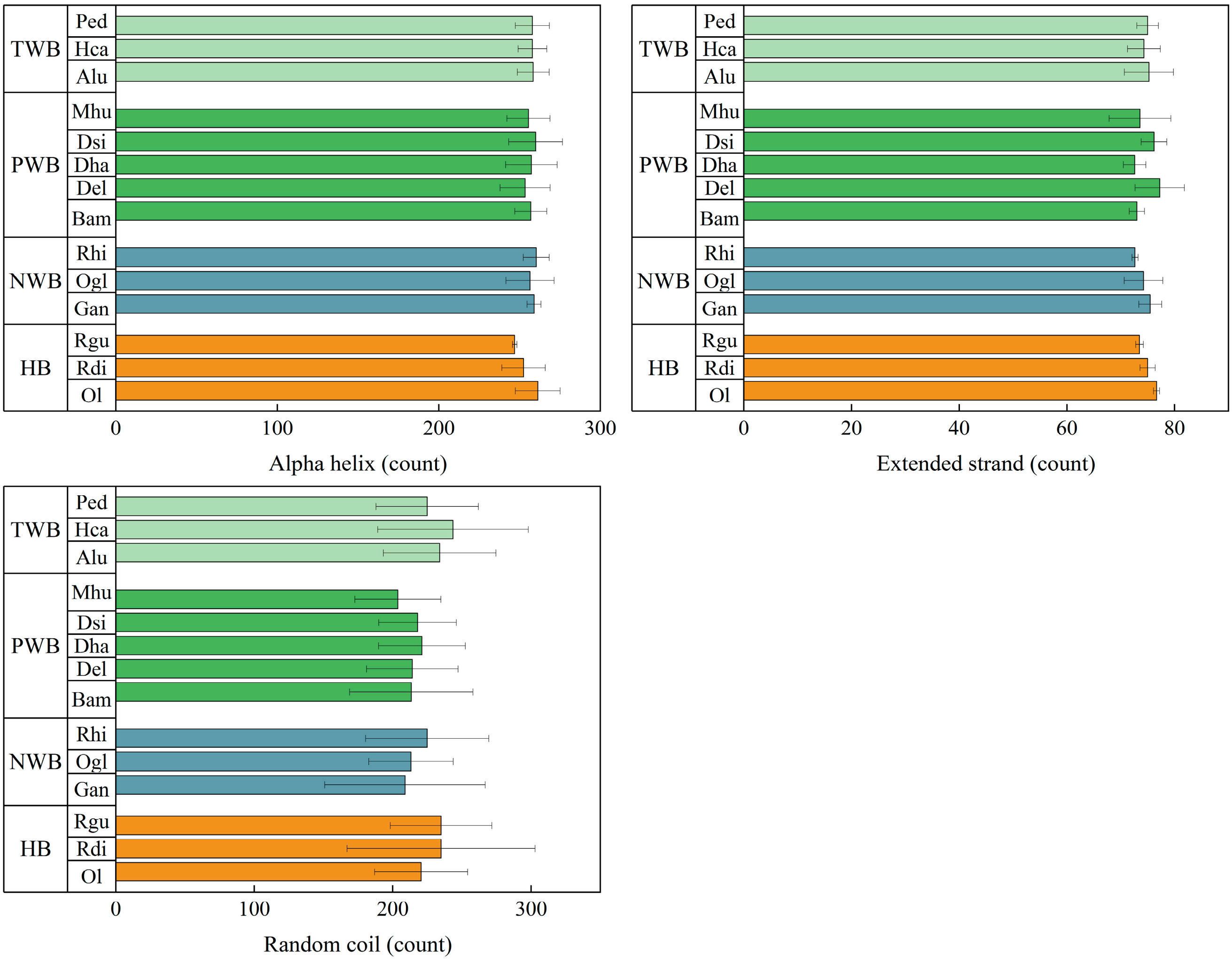

Supplementary Figure S11 Secondary structure of INT gene family proteins in 14 bamboo species

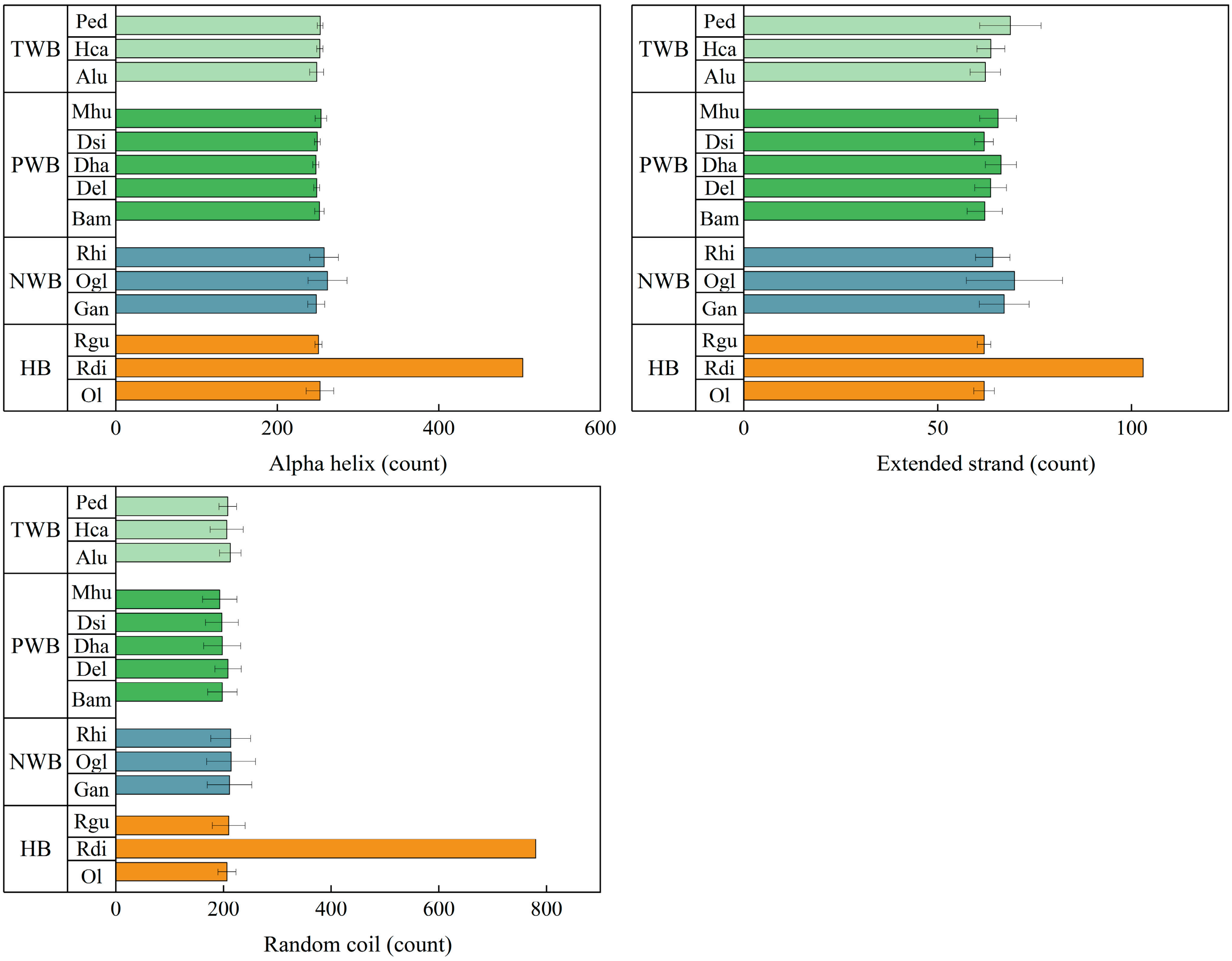

Supplementary Figure S12 Secondary structure of pGlcT gene family proteins in 14 bamboo species

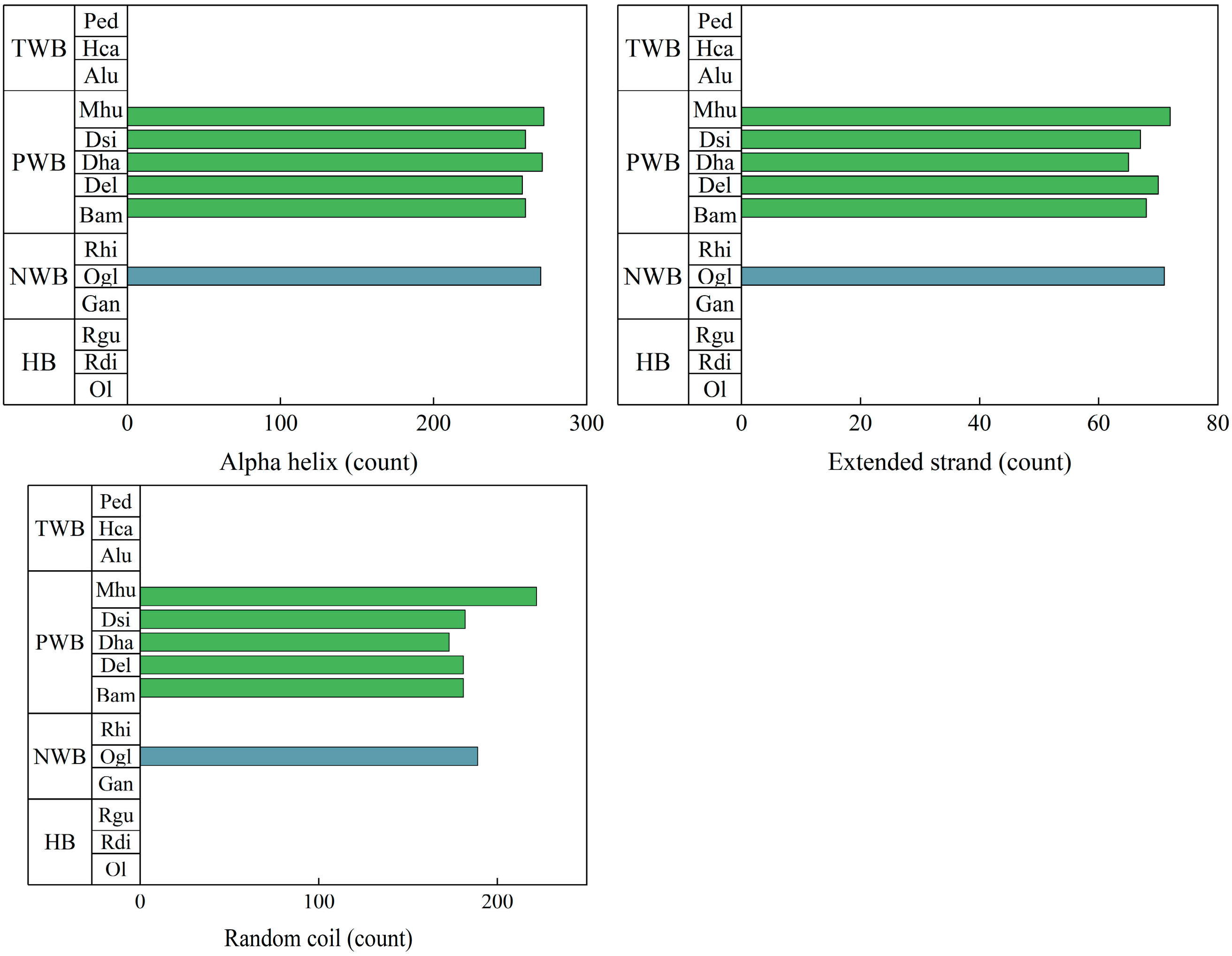

Supplementary Figure S13 Secondary structure of PMT gene family proteins in 14 bamboo species

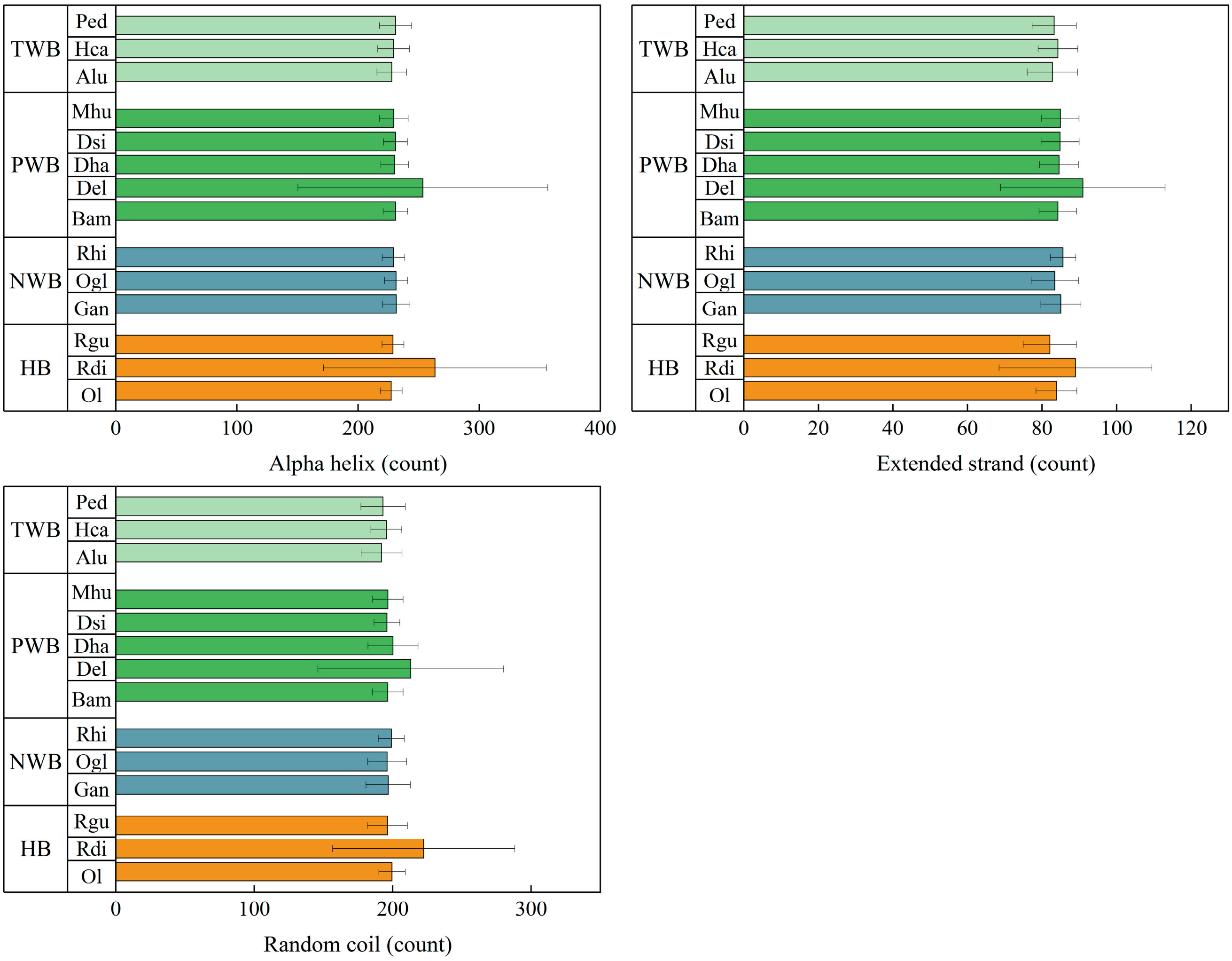

Supplementary Figure S14 Secondary structure of STP gene family proteins in 14 bamboo species

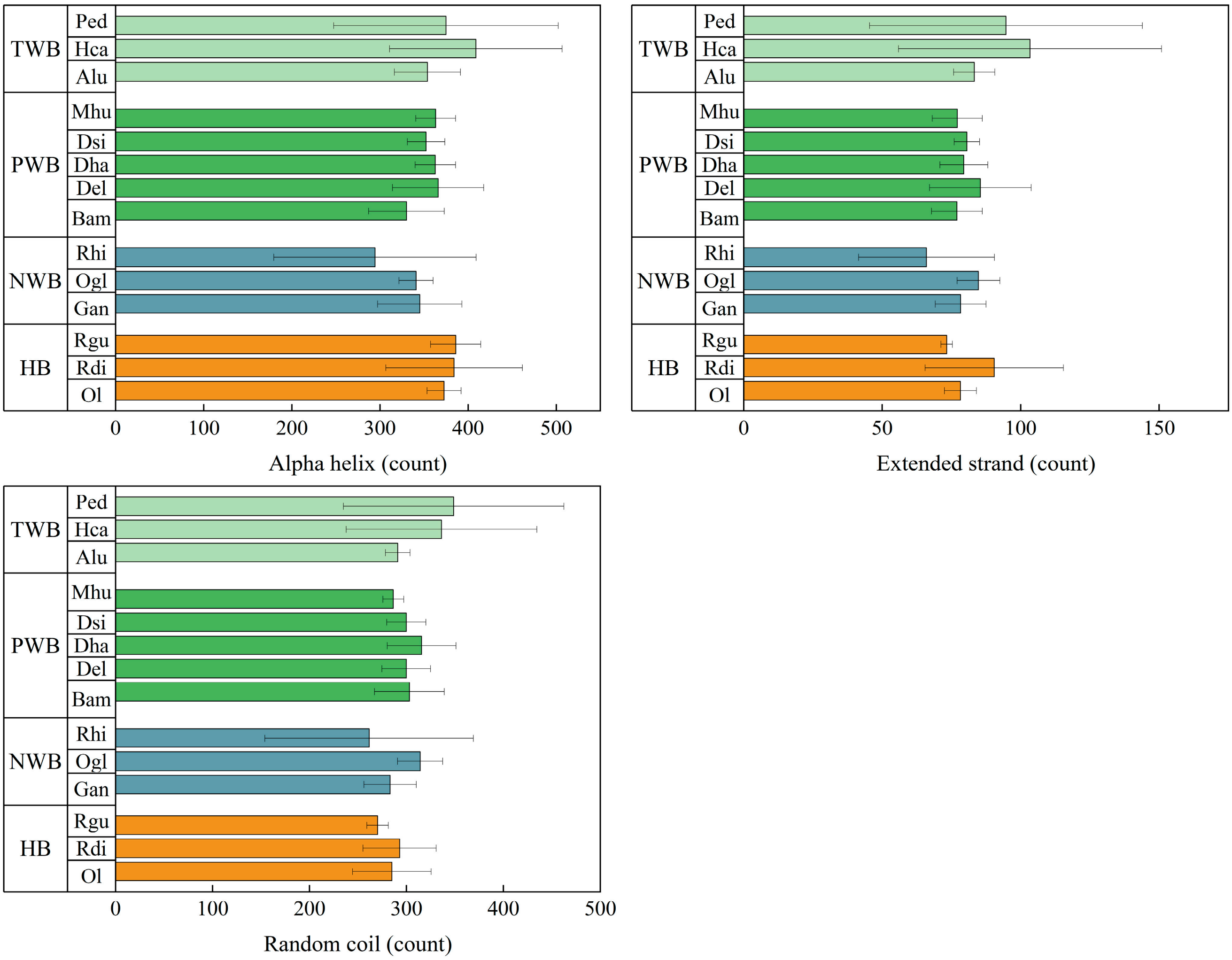

Supplementary Figure S15 Secondary structure of TMT gene family proteins in 14 bamboo species

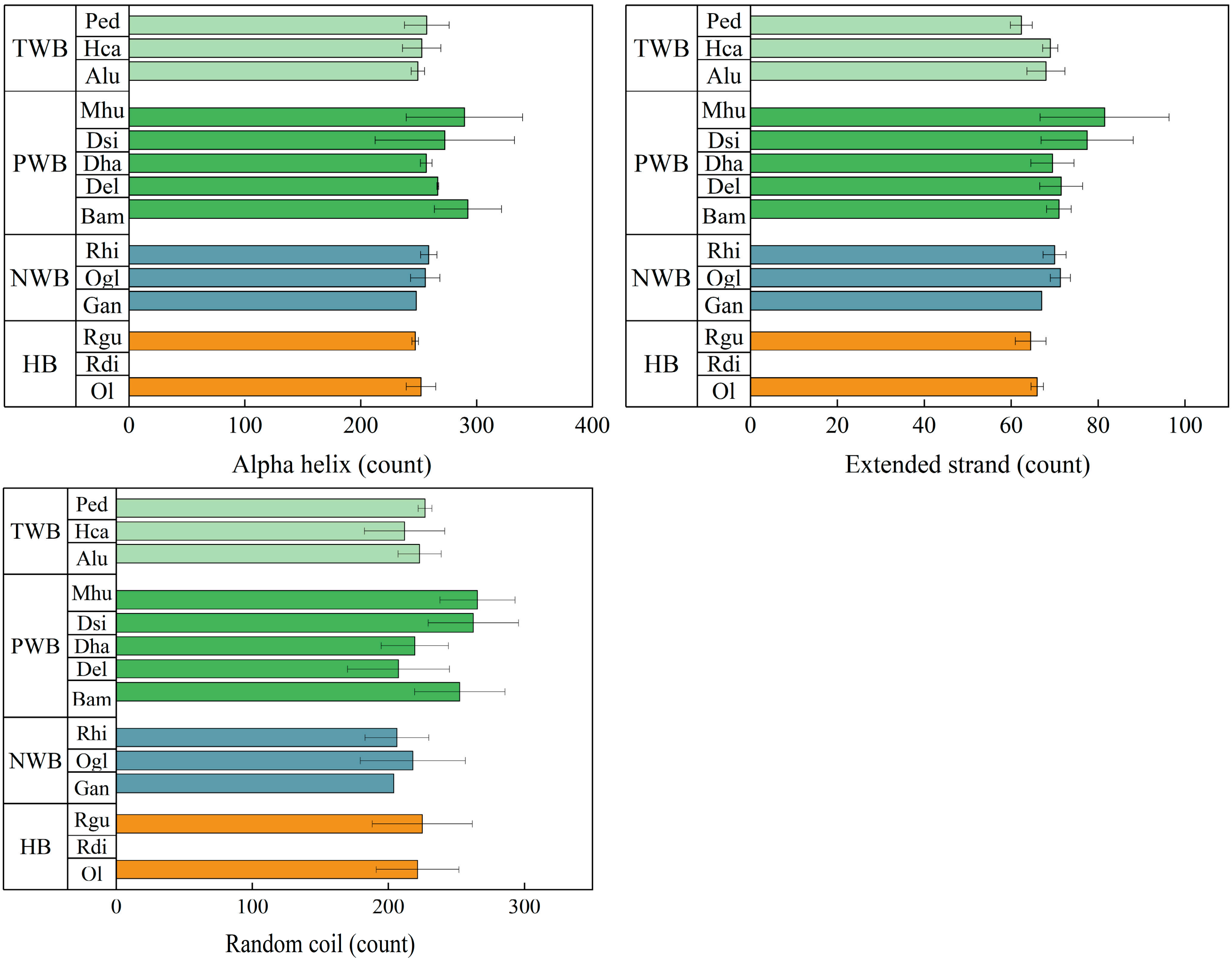

Supplementary Figure S16 Secondary structure of VGT gene family proteins in 14 bamboo species

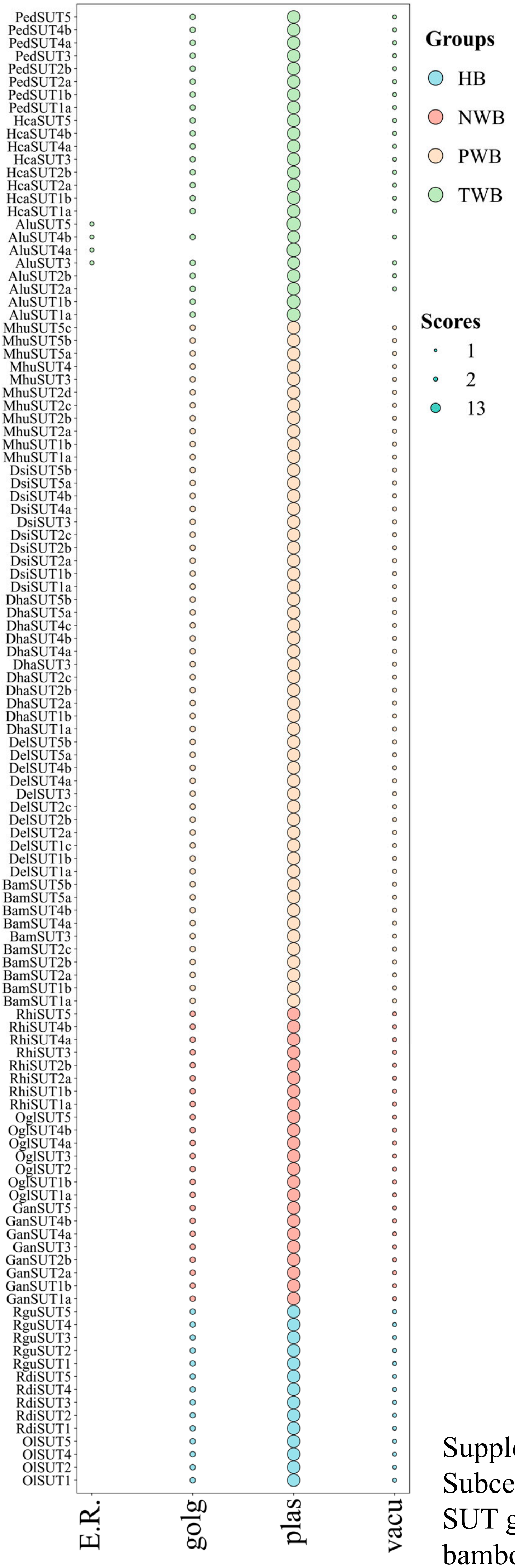

Supplementary Figure S17
Subcellular localization prediction of SUT gene family proteins in 14 bamboo species

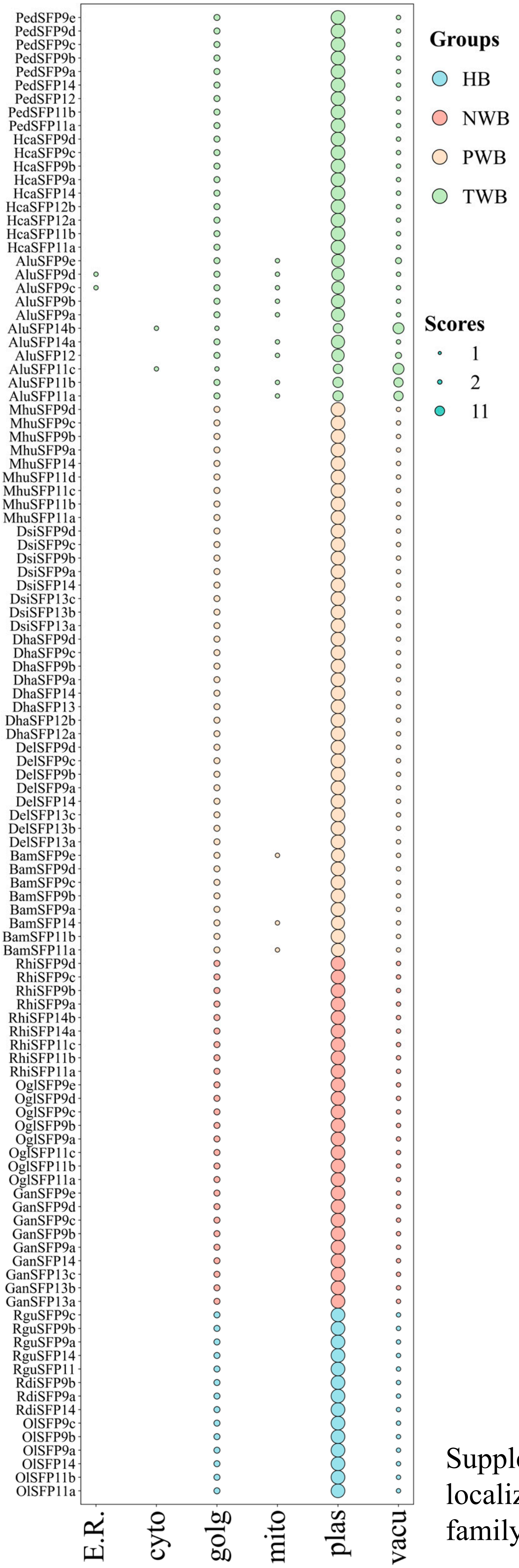

Supplementary Figure S18 Subcellular localization prediction of ERDL6 gene family proteins in 14 bamboo species

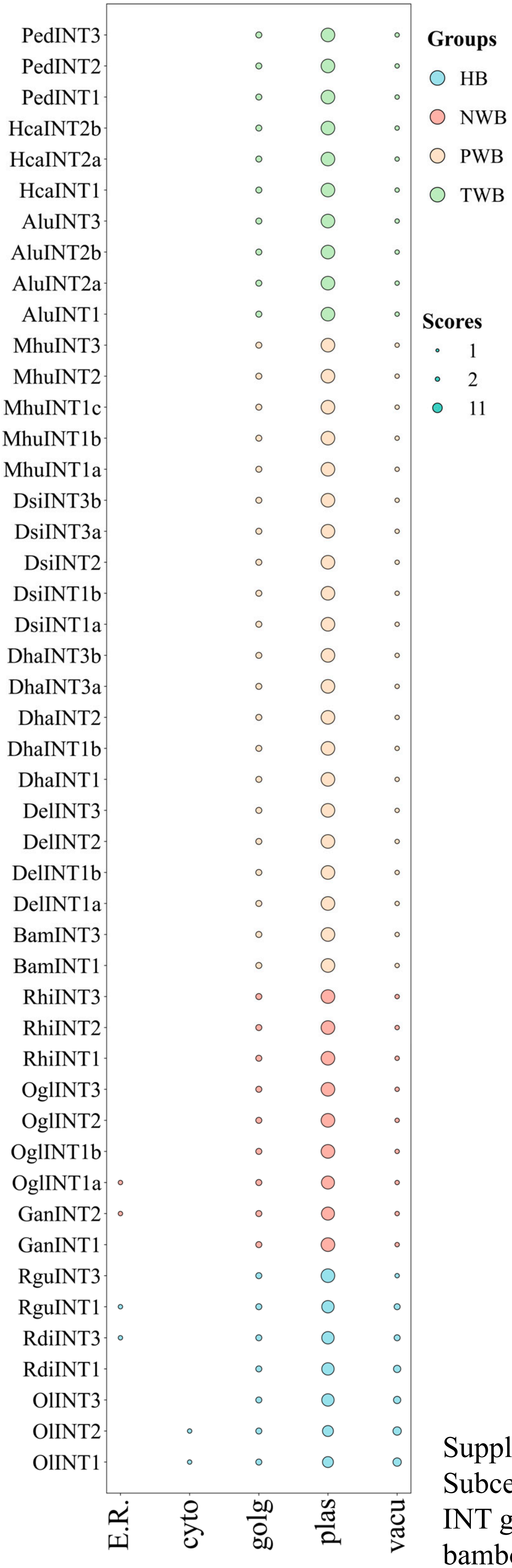

Supplementary Figure S19 Subcellular localization prediction of INT gene family proteins in 14 bamboo species

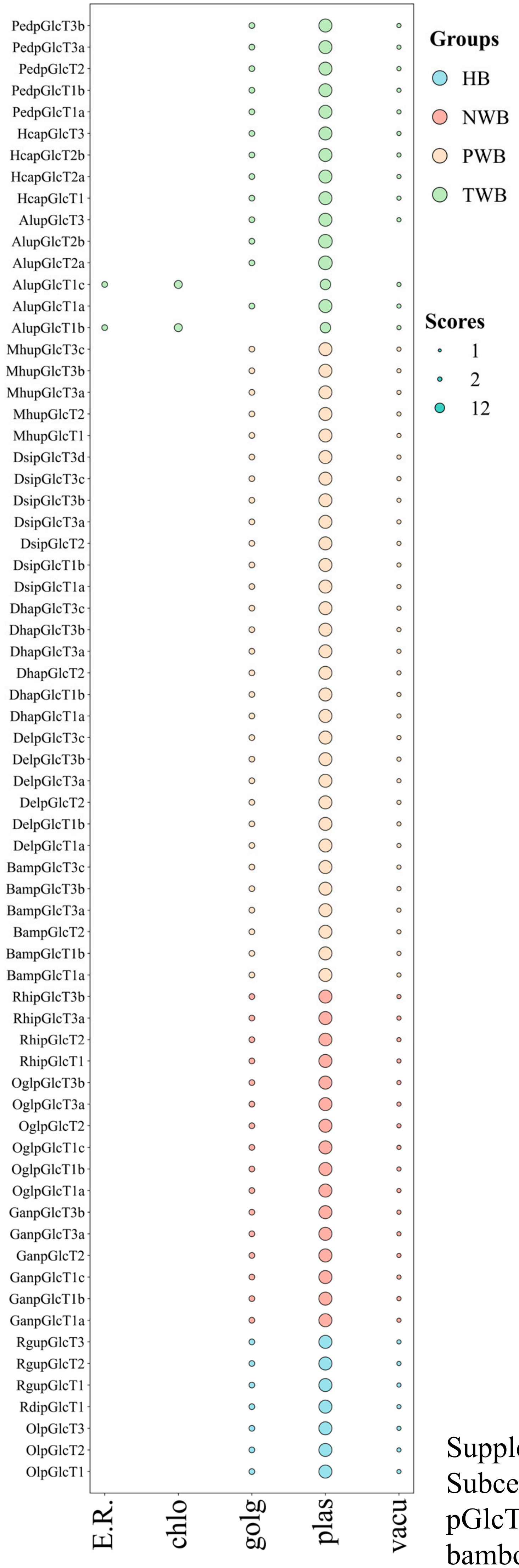

Supplementary Figure S20
Subcellular localization prediction of pGlcT gene family proteins in 14 bamboo species

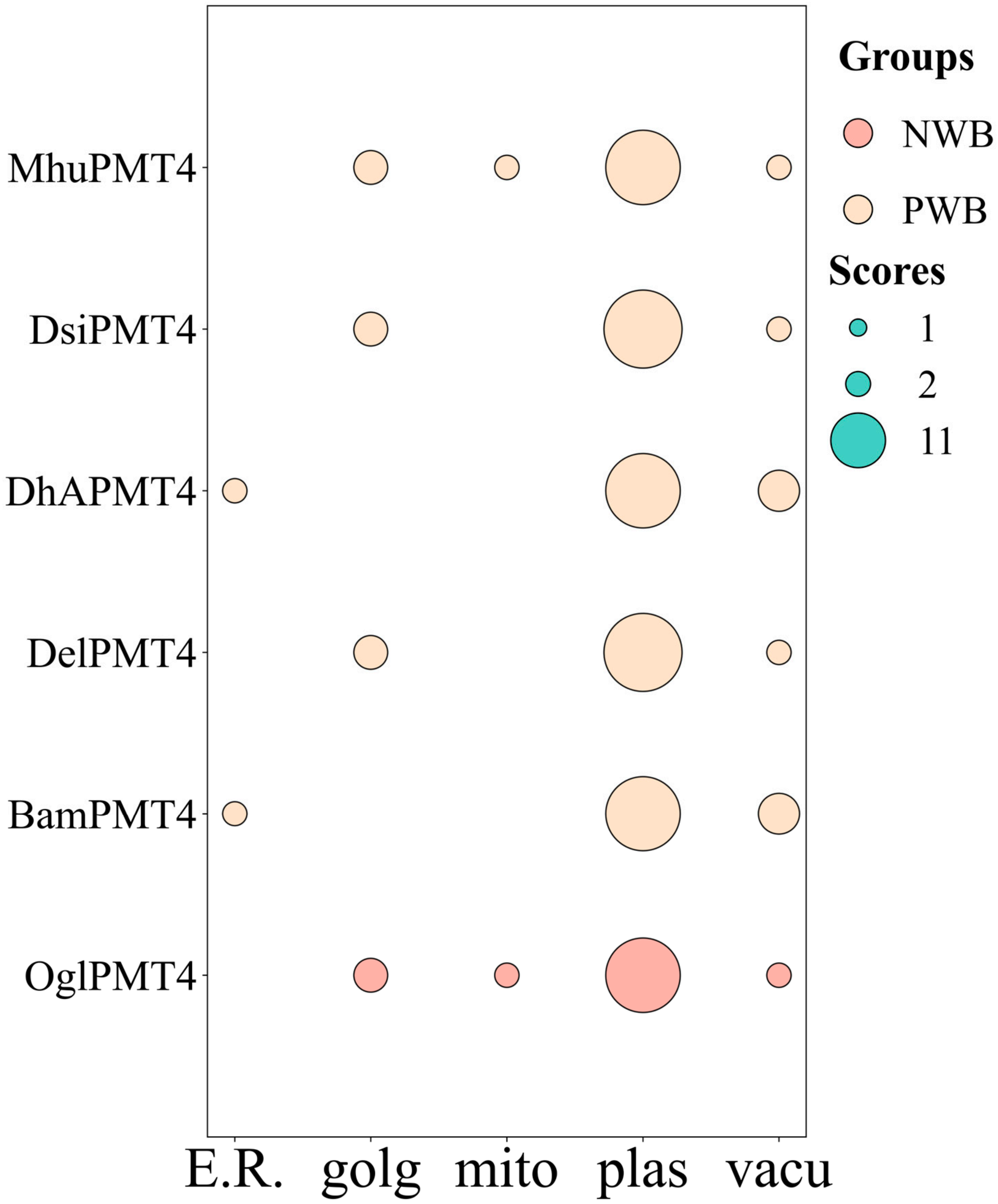

Supplementary Figure S21 Subcellular localization prediction of PMT gene family proteins in 14 bamboo species

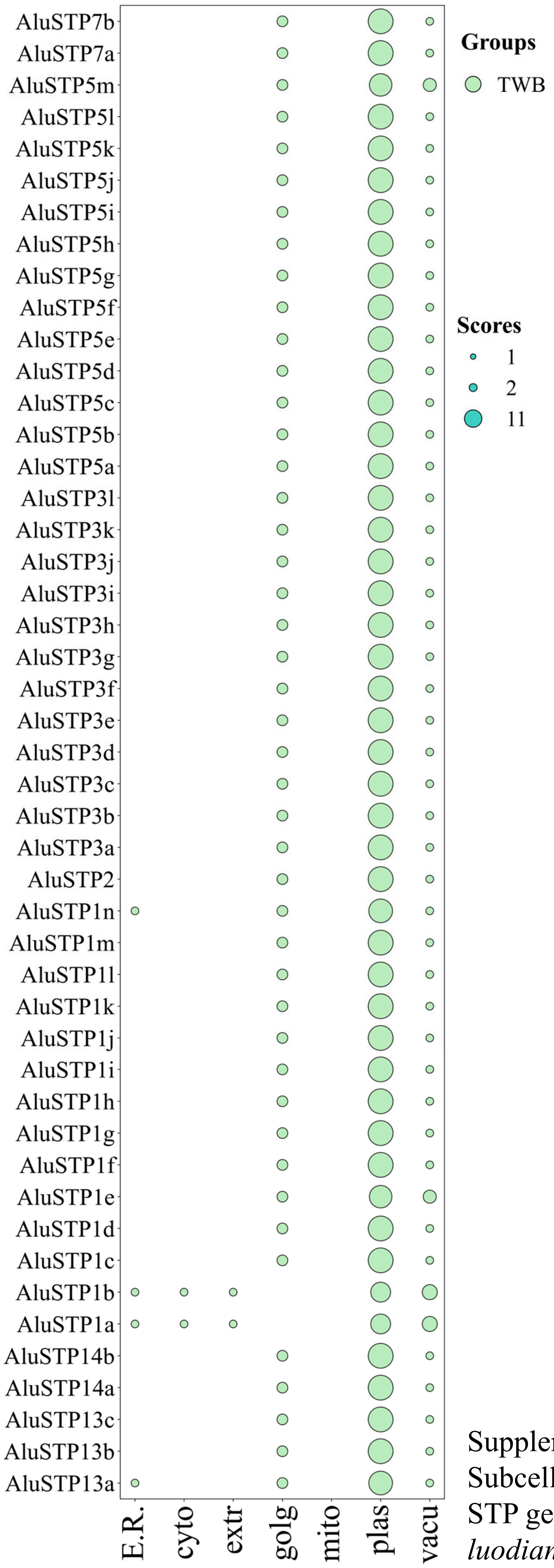

Supplementary Figure S22 Subcellular localization prediction of STP gene family proteins in *A. luodianensis*

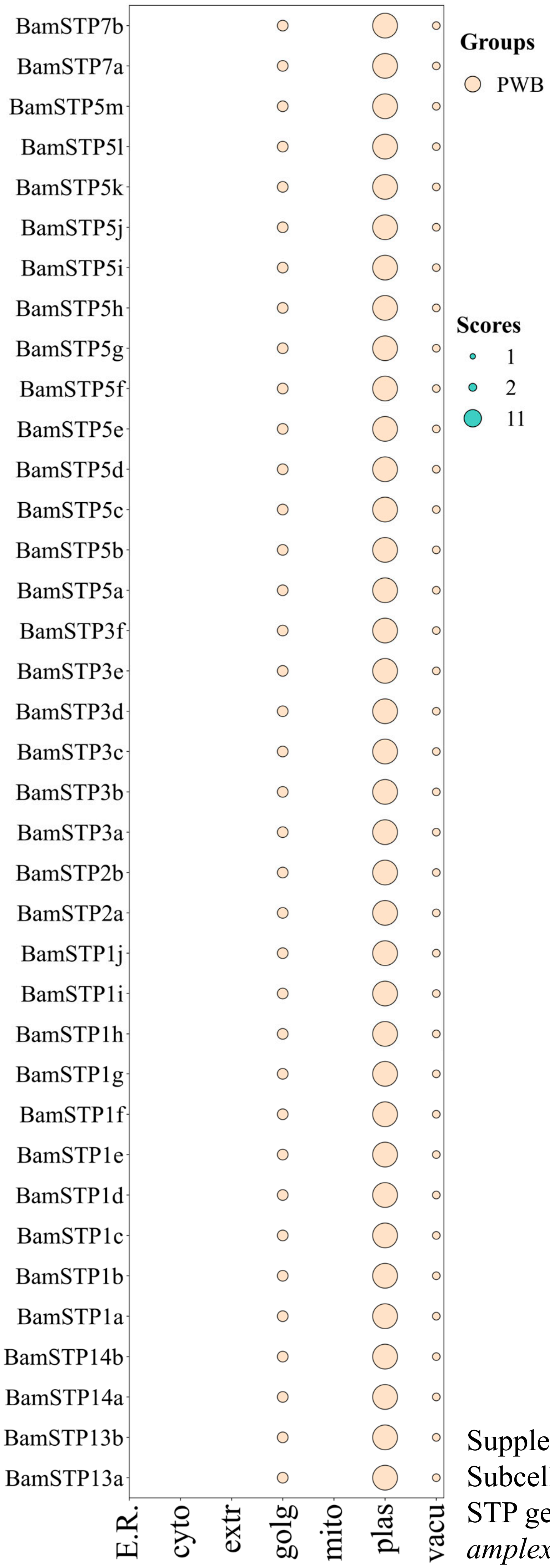

Supplementary Figure S23 Subcellular localization prediction of STP gene family proteins in *B. amplexicaulis*

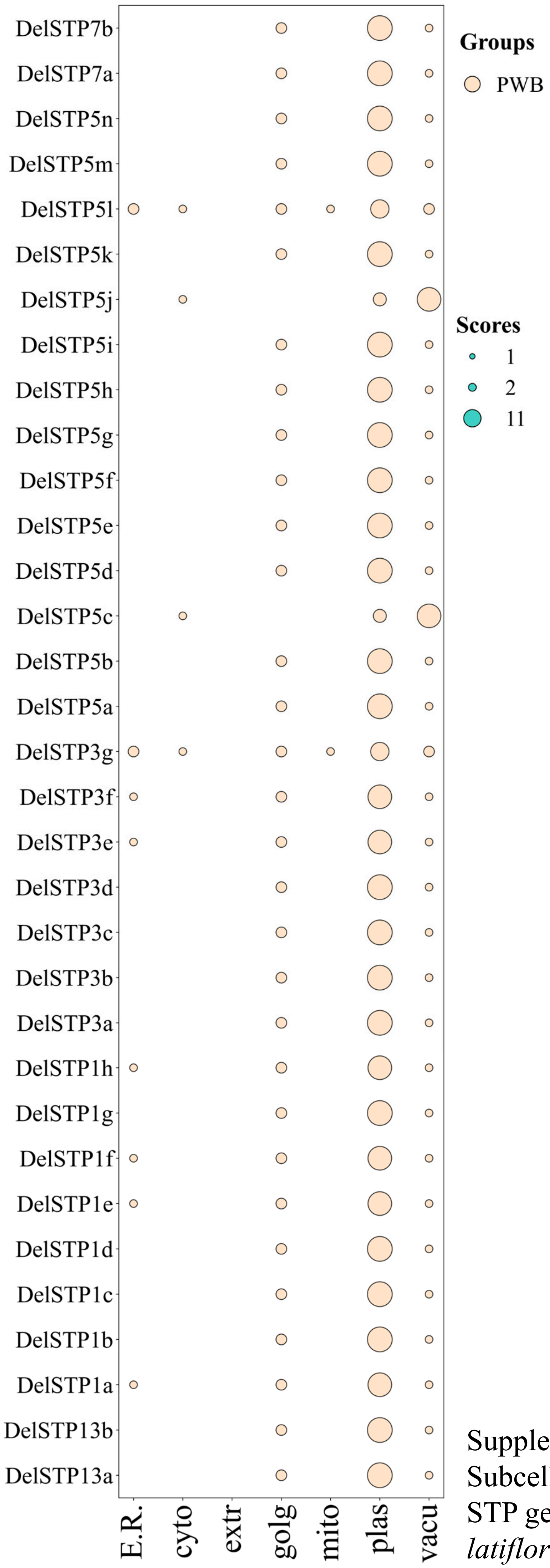

Supplementary Figure S24 Subcellular localization prediction of STP gene family proteins in *D. latiflorus*

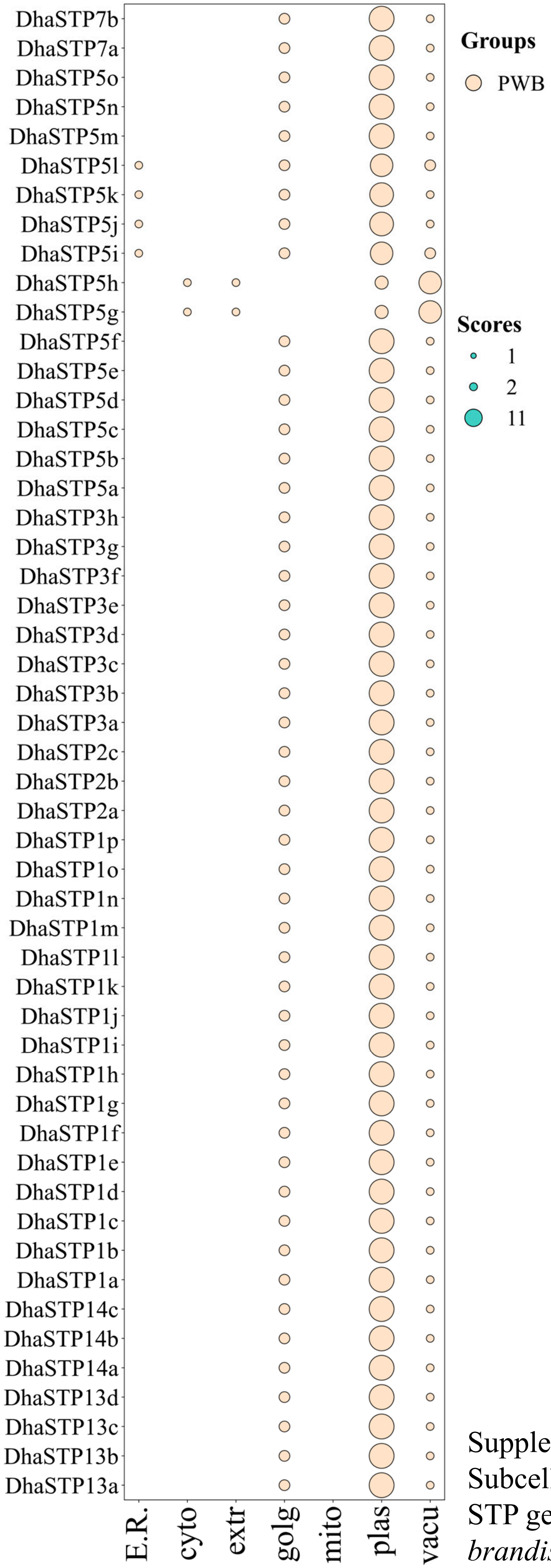

Supplementary Figure S25
Subcellular localization prediction of STP gene family proteins in *D. brandisii*

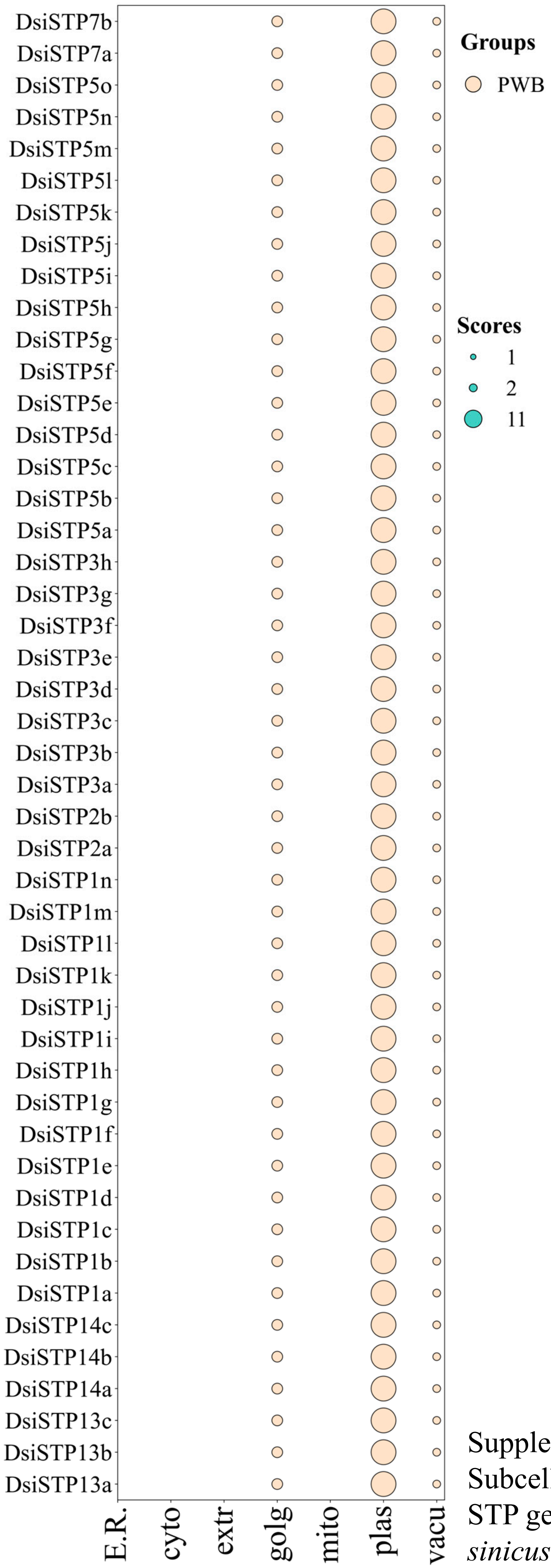

Supplementary Figure S26 Subcellular localization prediction of STP gene family proteins in *D. sinicus*

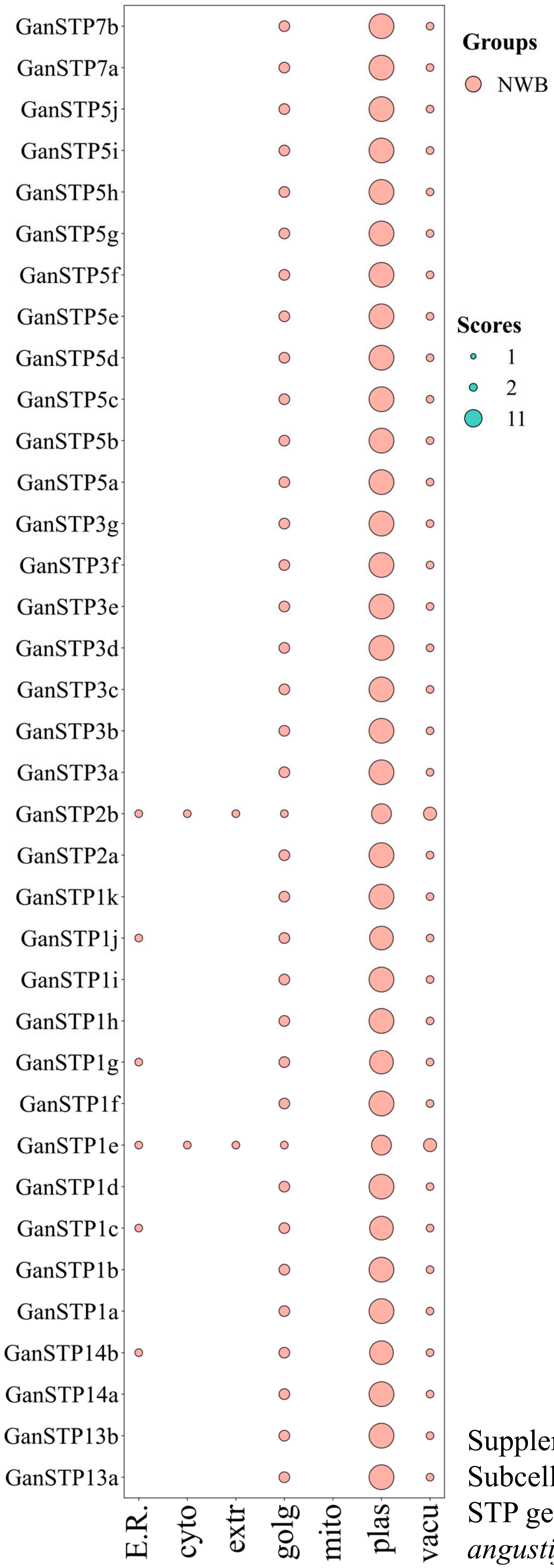

Supplementary Figure S27
Subcellular localization prediction of STP gene family proteins in *G. angustifolia*

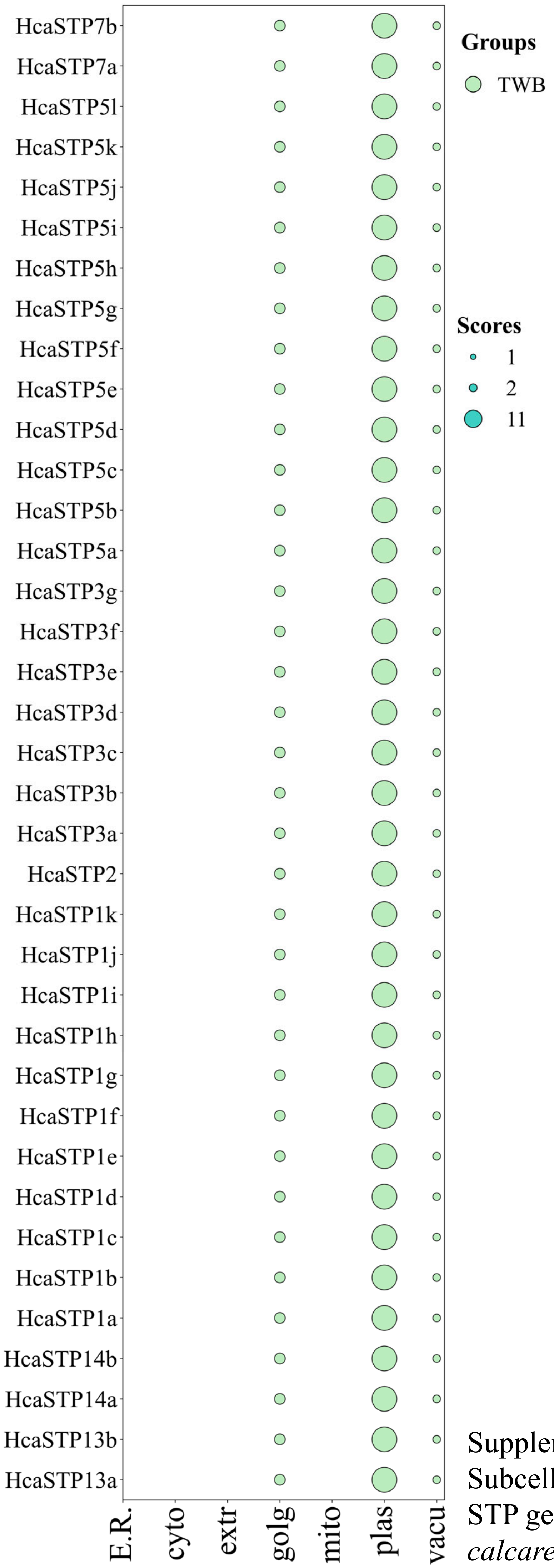

Supplementary Figure S28
Subcellular localization prediction of STP gene family proteins in *H. calcarea*

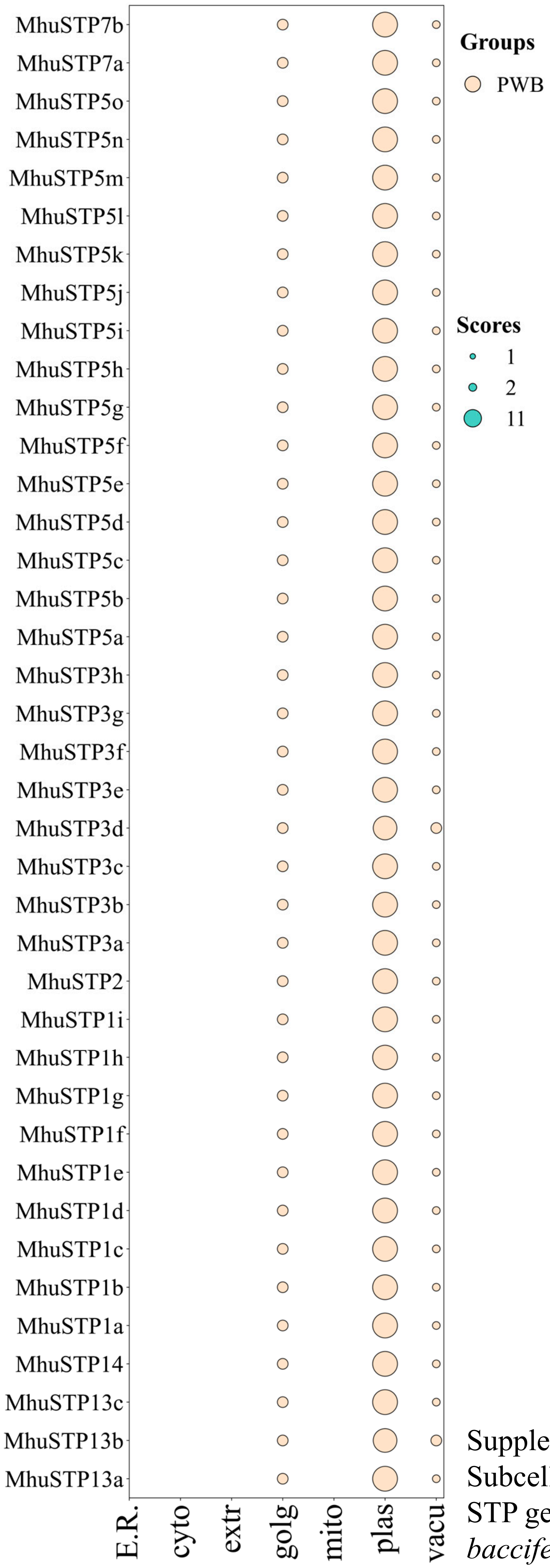

Supplementary Figure S29 Subcellular localization prediction of STP gene family proteins in *M. baccifera*

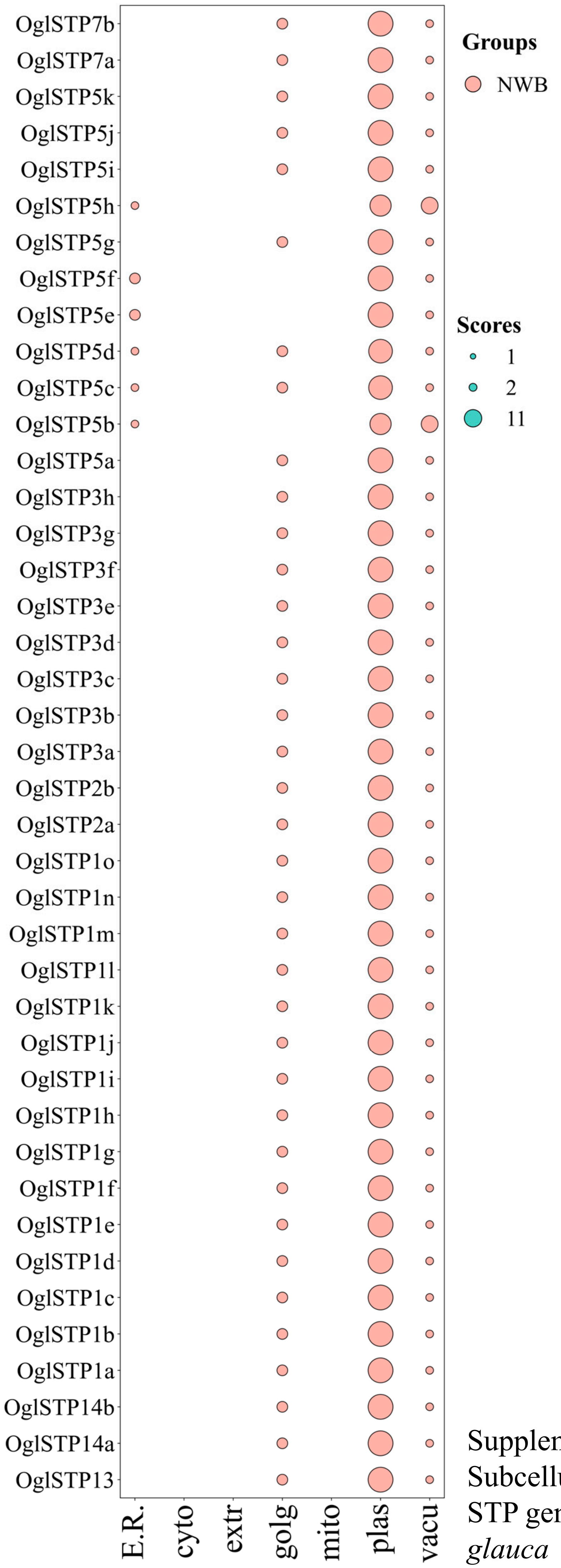

Supplementary Figure S30
Subcellular localization prediction of STP gene family proteins in *Ot. glauca*

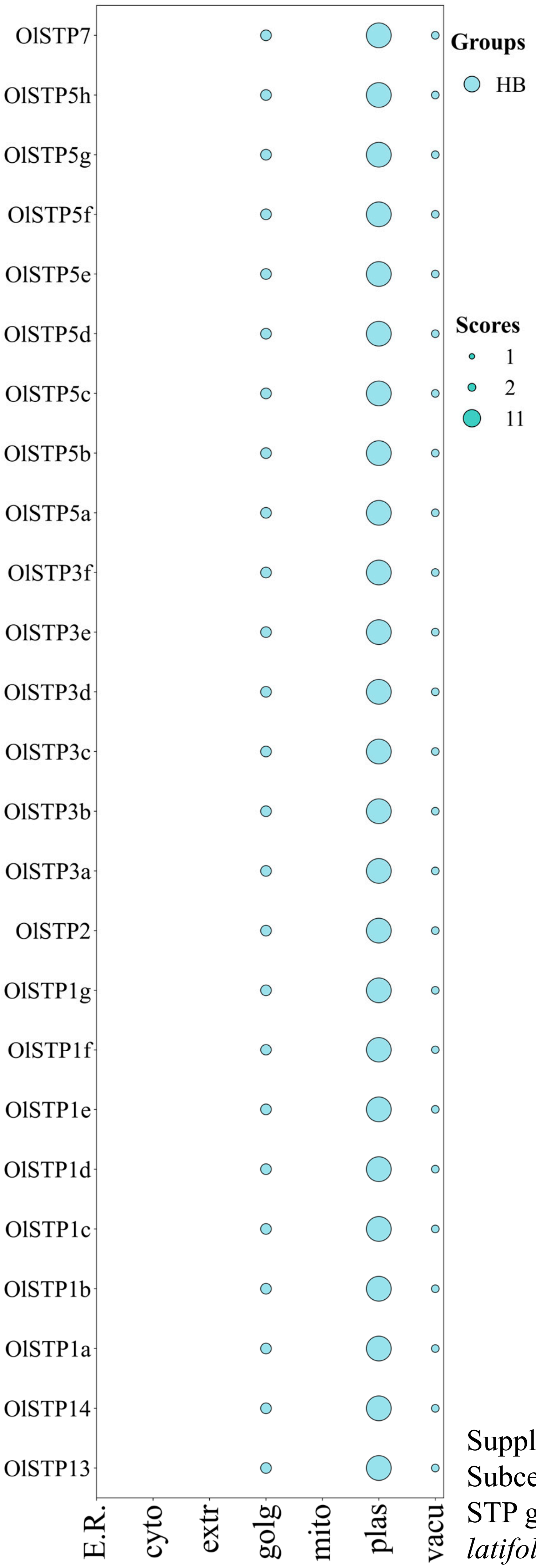

Supplementary Figure S31 Subcellular localization prediction of STP gene family proteins in *Ol. latifolia*

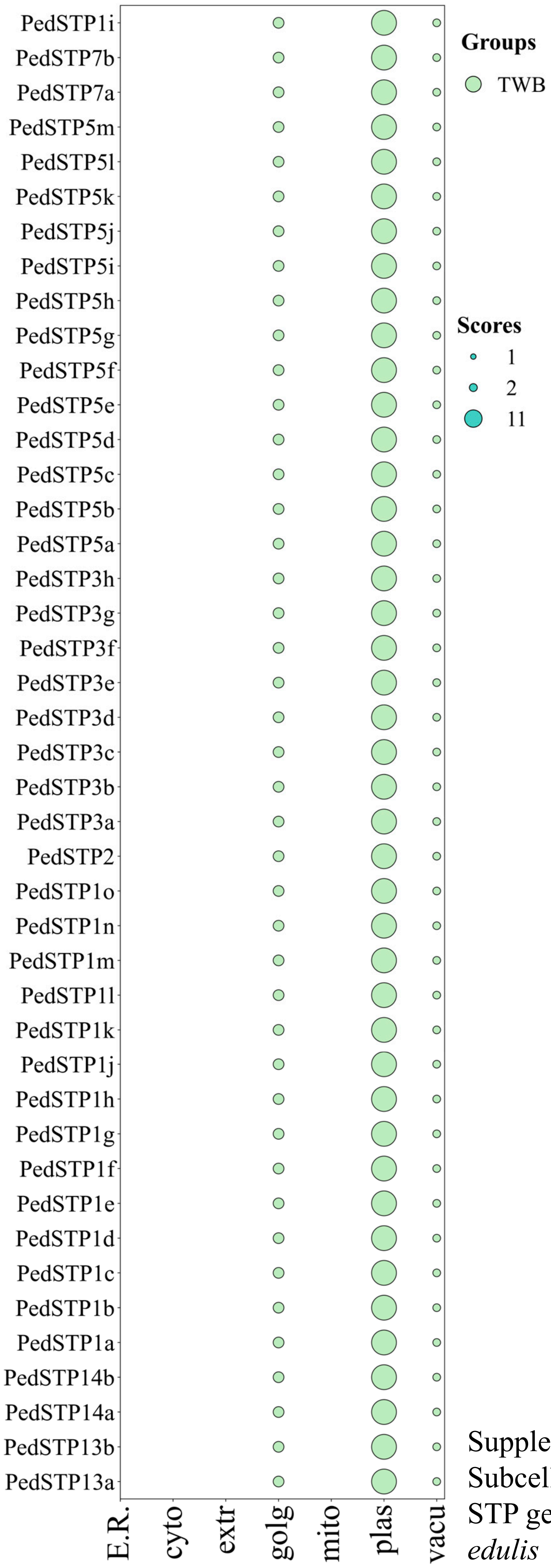

Supplementary Figure S32
Subcellular localization prediction of STP gene family proteins in *Ph. edulis*

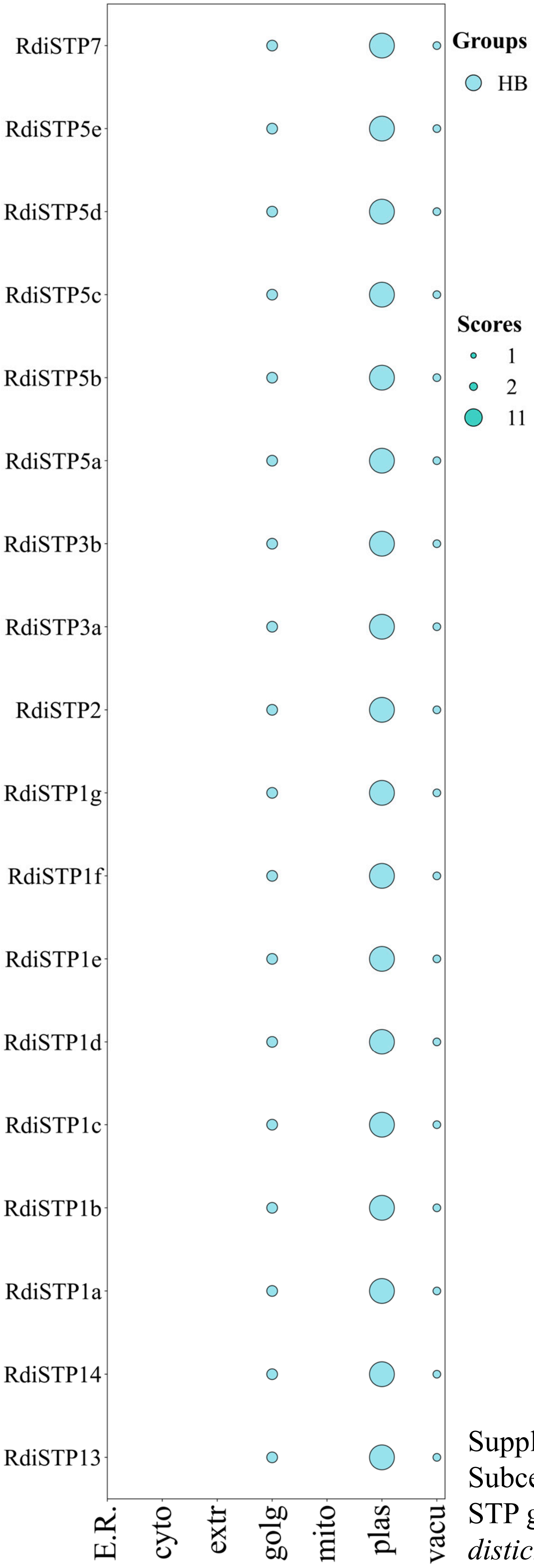

Supplementary Figure S33
Subcellular localization prediction of STP gene family proteins in *Ra. distichophylla*

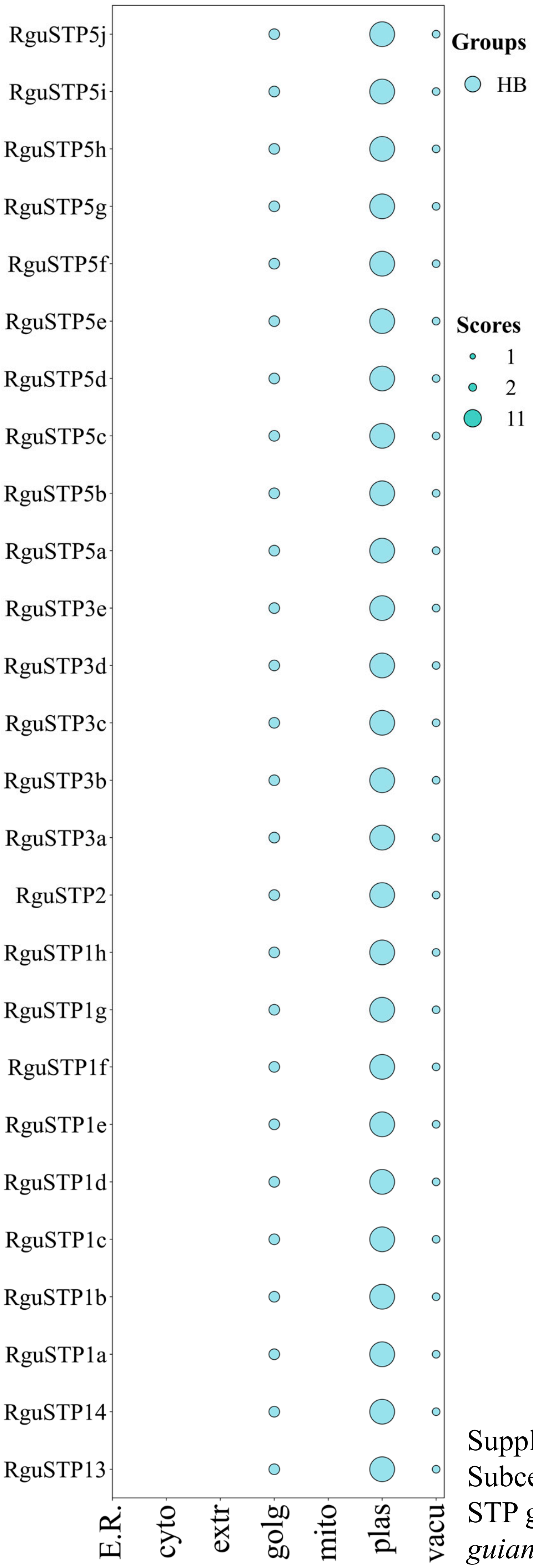

Supplementary Figure S34 Subcellular localization prediction of STP gene family proteins in *Ra. guianensis*

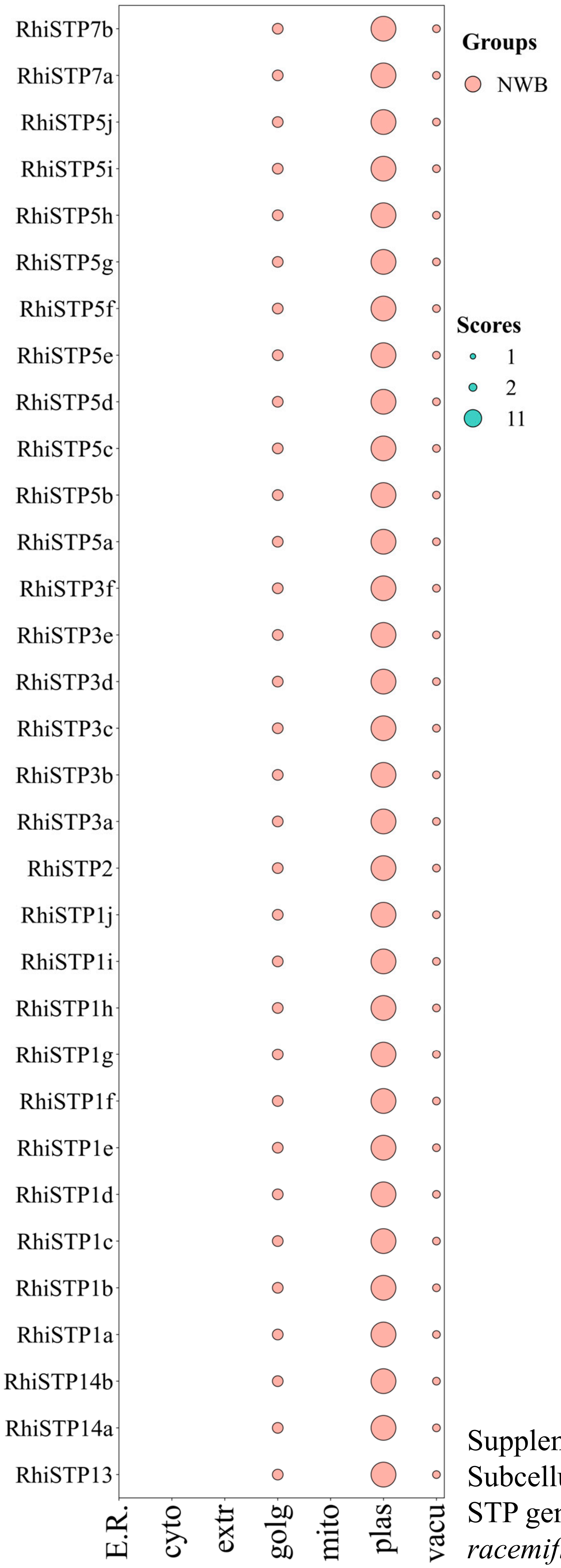

Supplementary Figure S35
Subcellular localization prediction of STP gene family proteins in *Rh. racemiflorum*

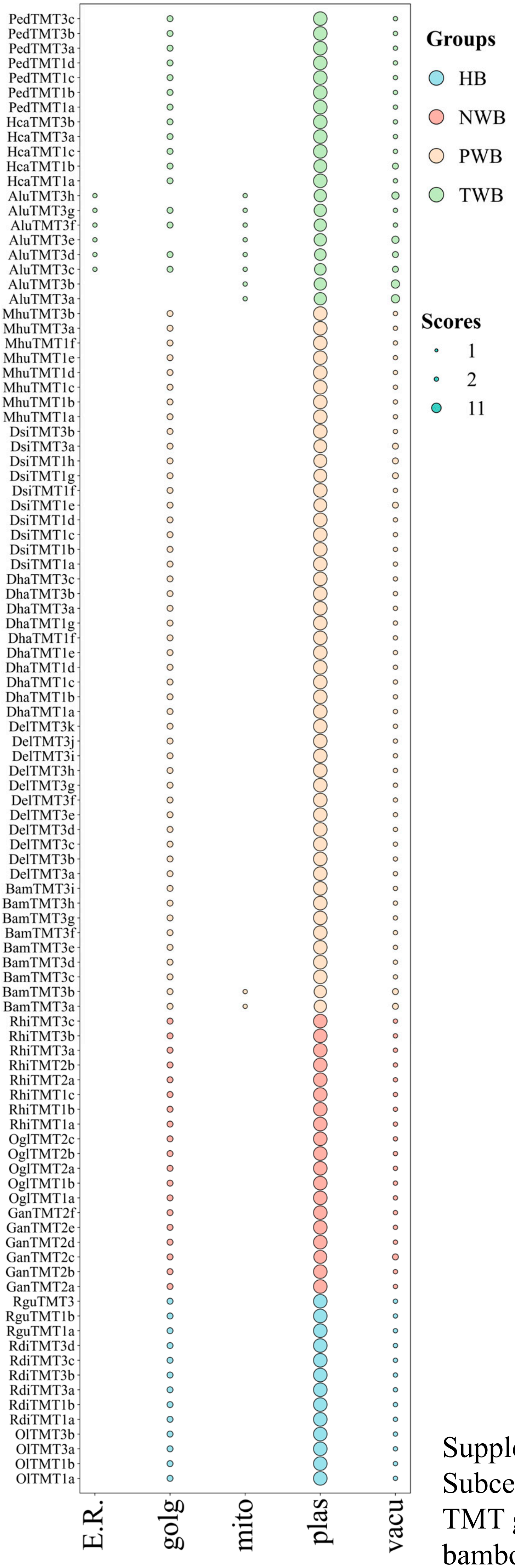

Supplementary Figure S36
Subcellular localization prediction of TMT gene family proteins in 14 bamboo species

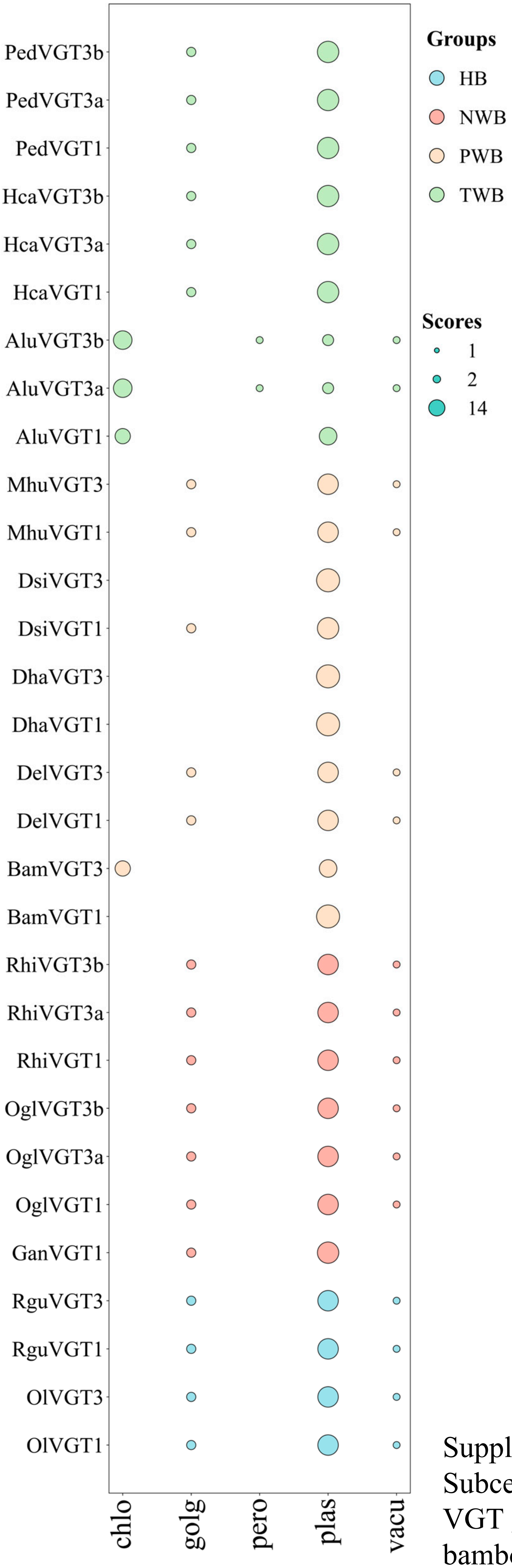

Supplementary Figure S37 Subcellular localization prediction of VGT gene family proteins in 14 bamboo species

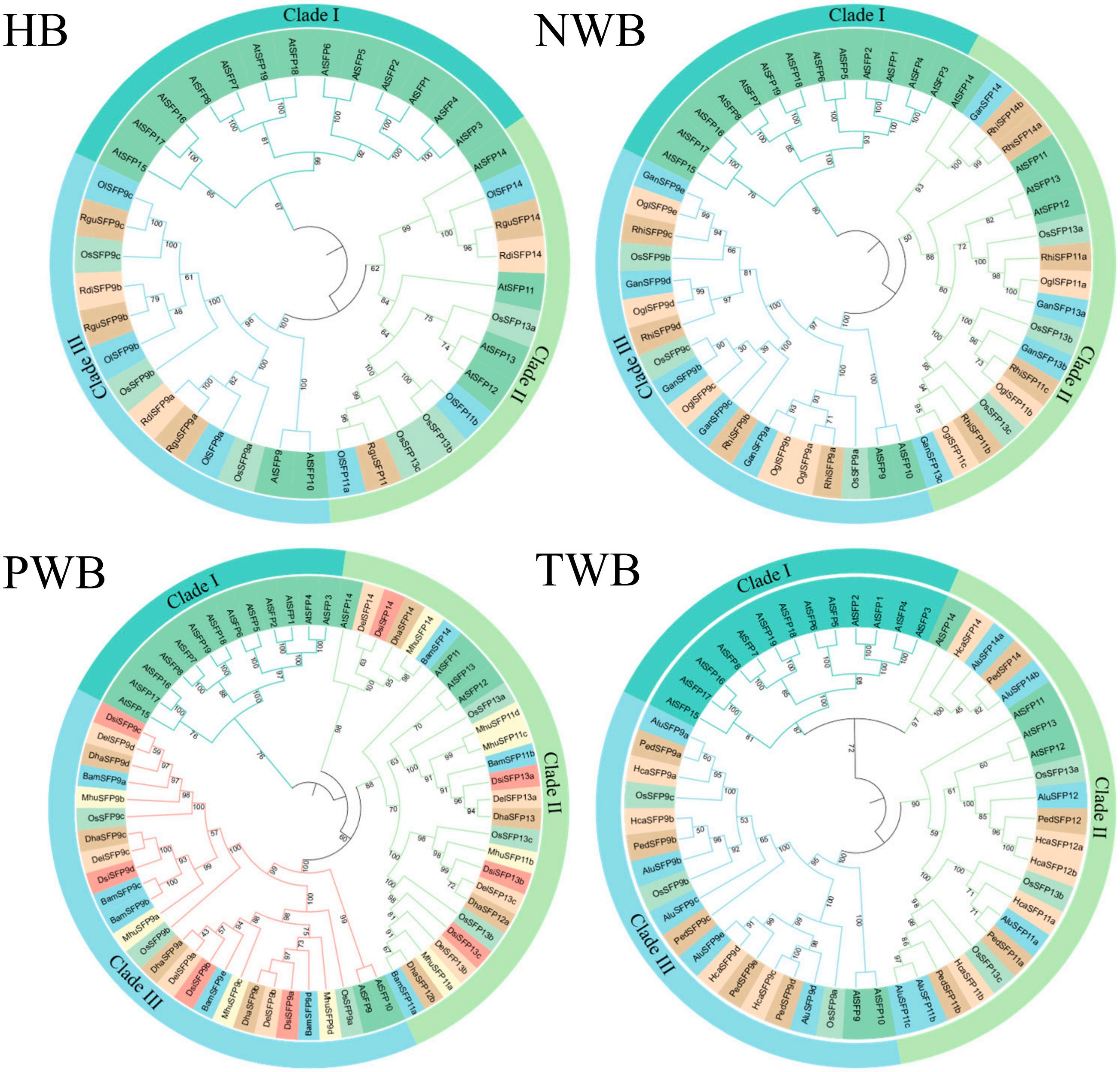

Supplementary Figure S38 Phylogenetic tree of the ERDL6 gene family

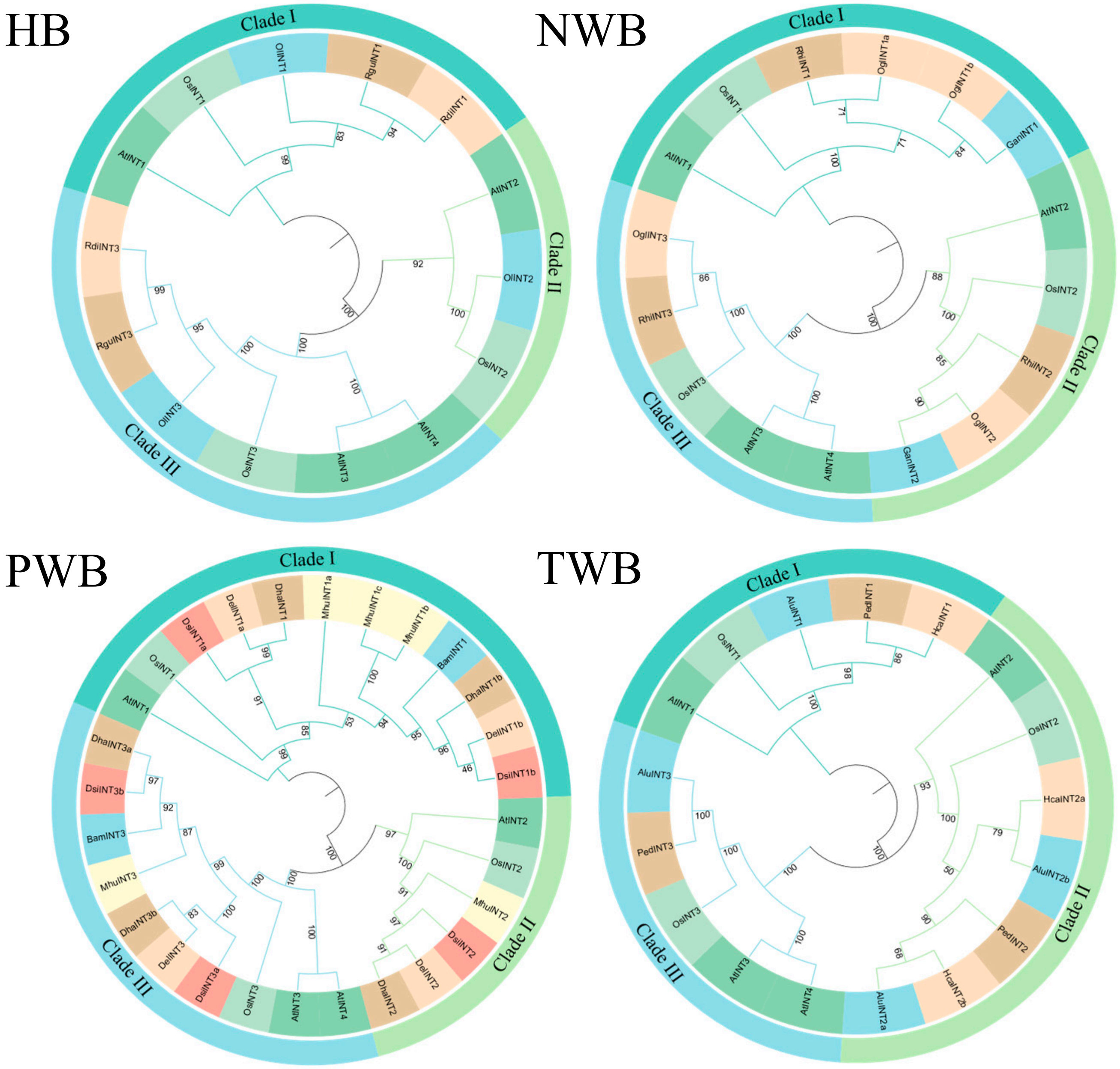

Supplementary Figure S39 Phylogenetic tree of the INT gene family

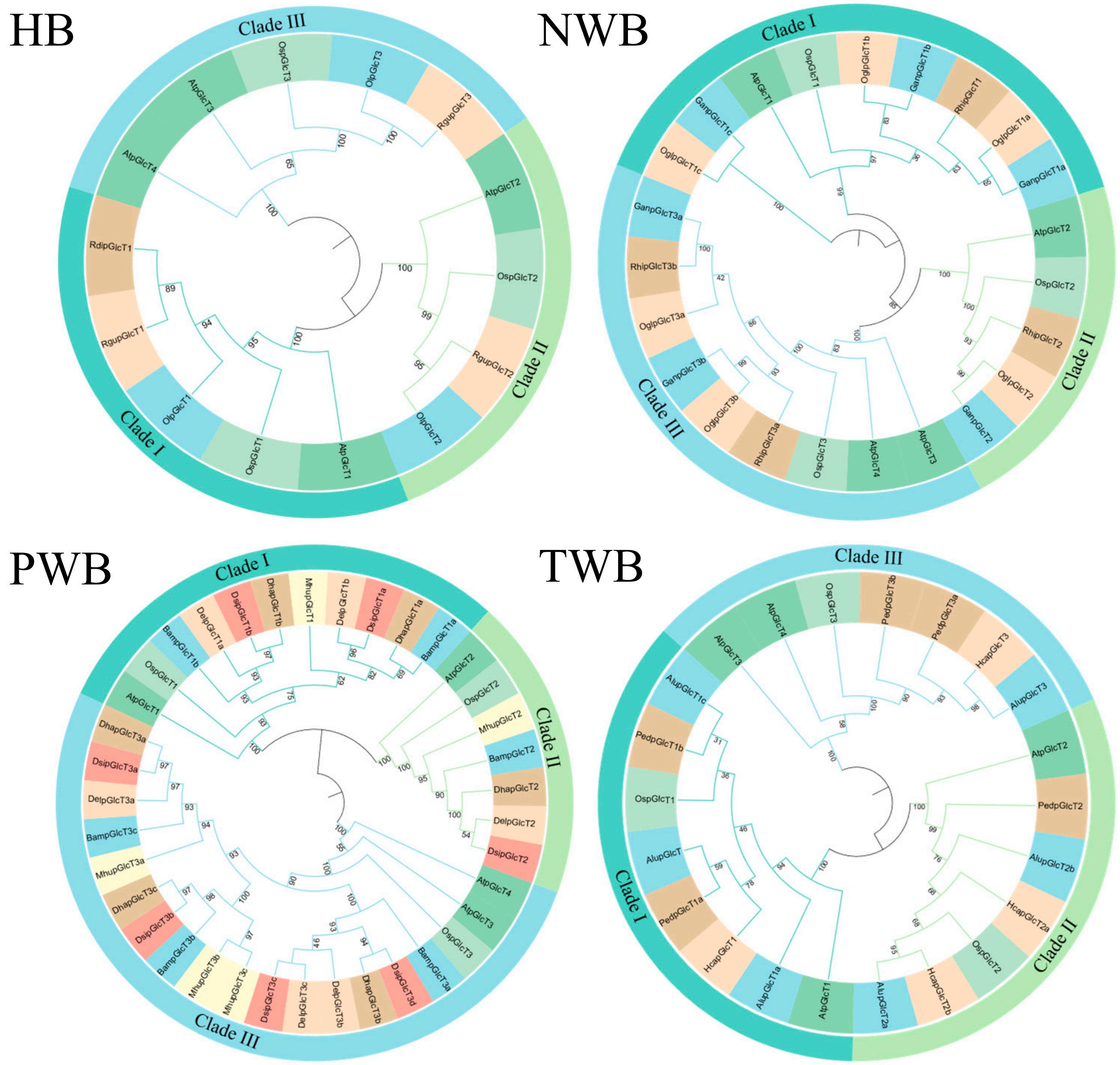

Supplementary Figure S40 Phylogenetic tree of the pGlcT gene family

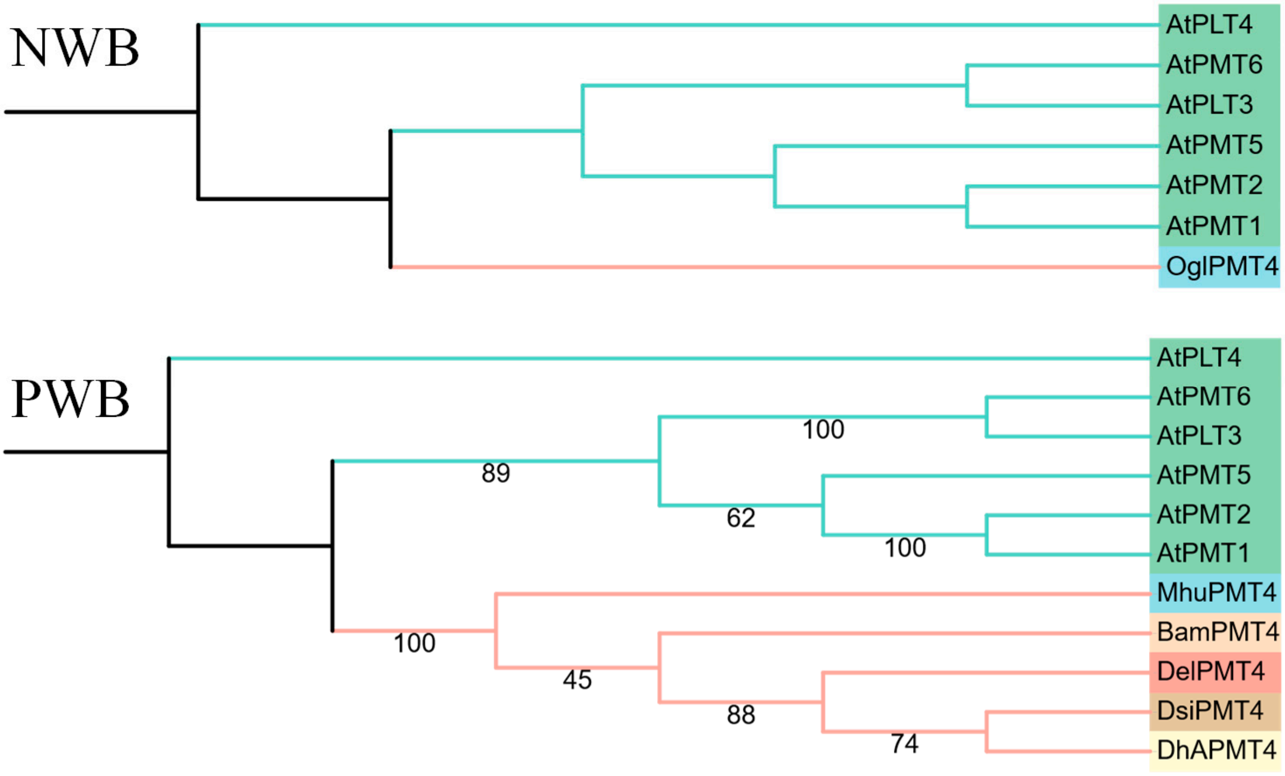

Supplementary Figure S41 Phylogenetic tree of the PMT gene family

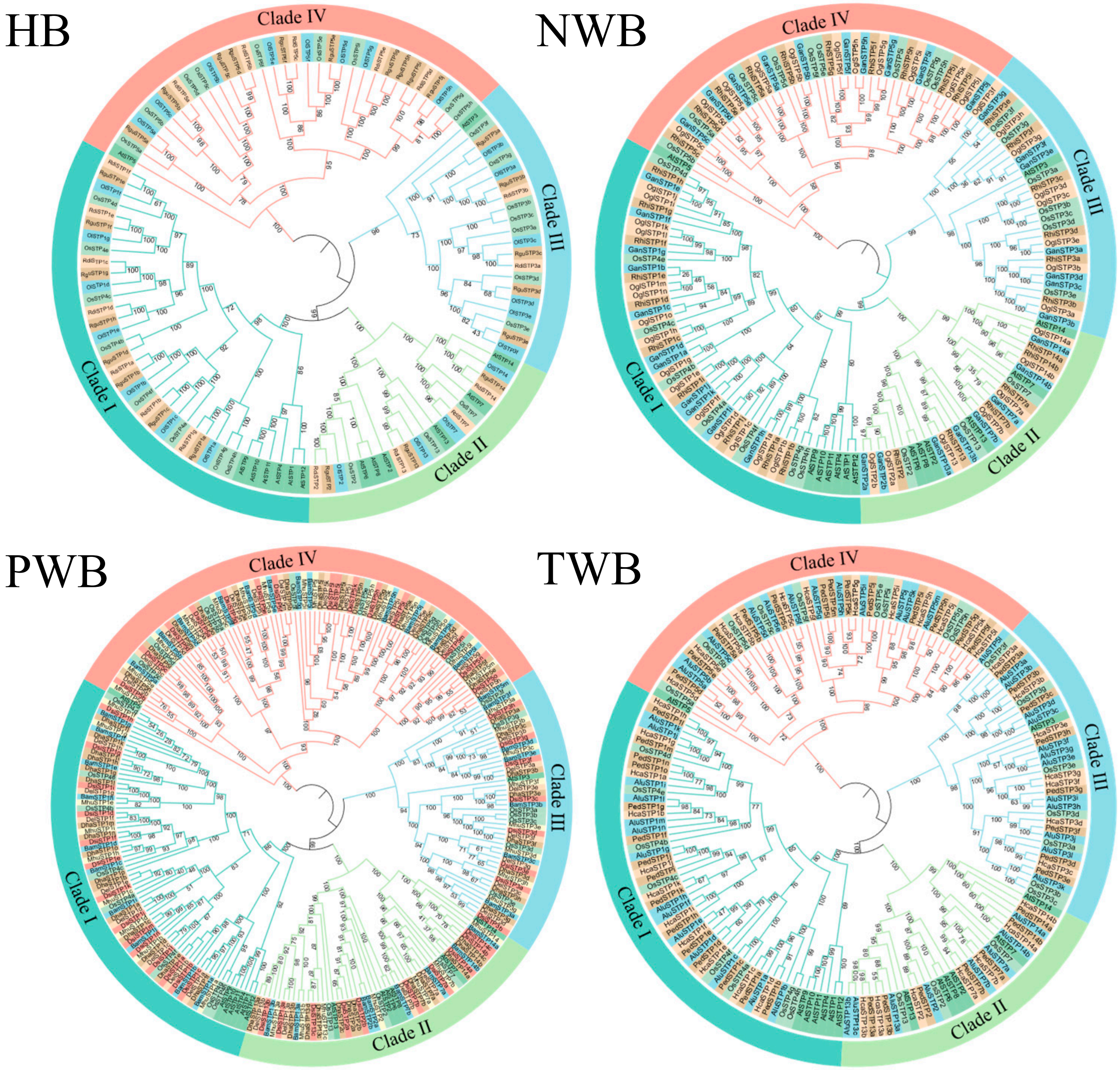

Supplementary Figure S42 Phylogenetic tree of the STP gene family

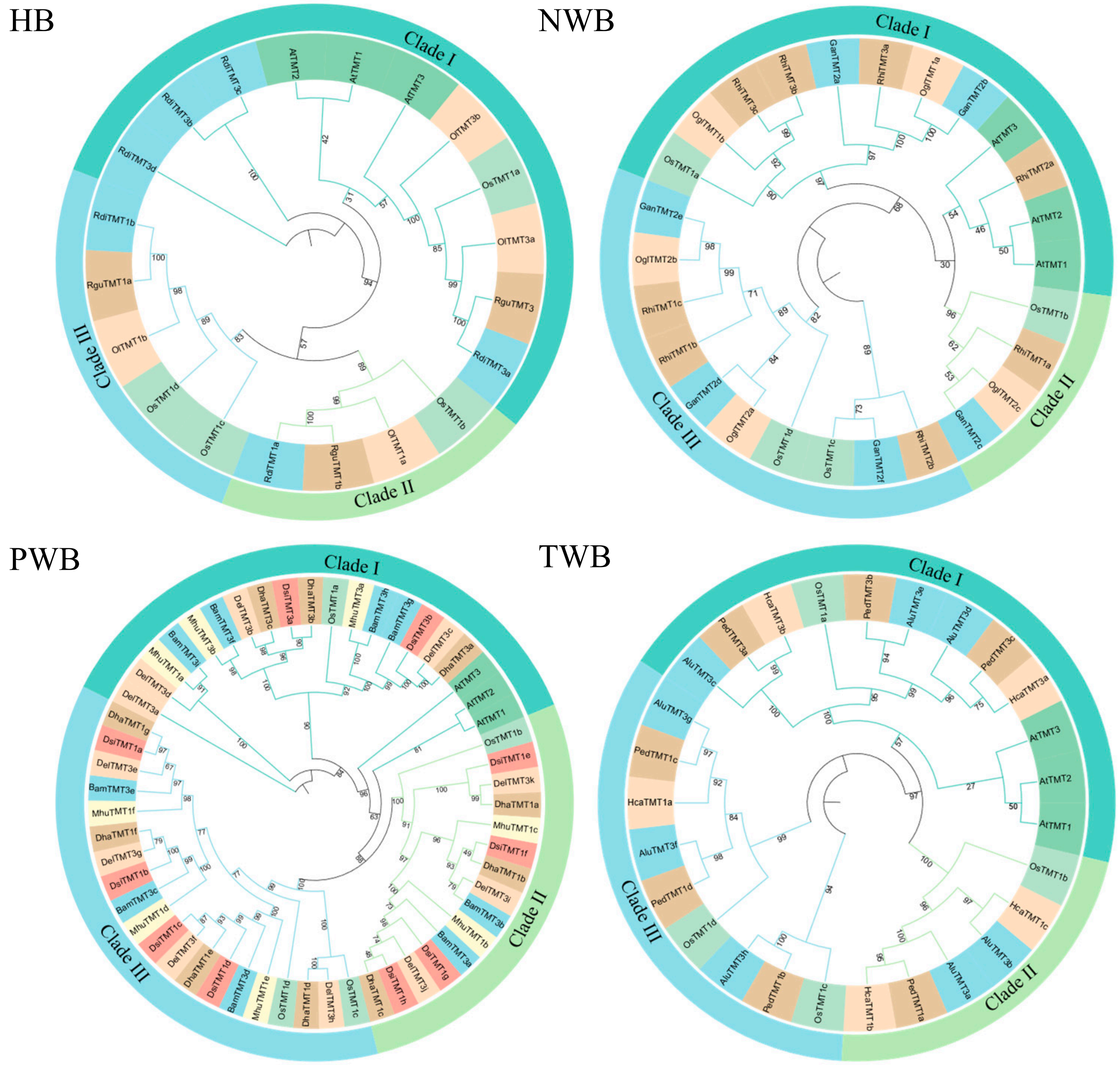

Supplementary Figure S43 Phylogenetic tree of the TMT gene family

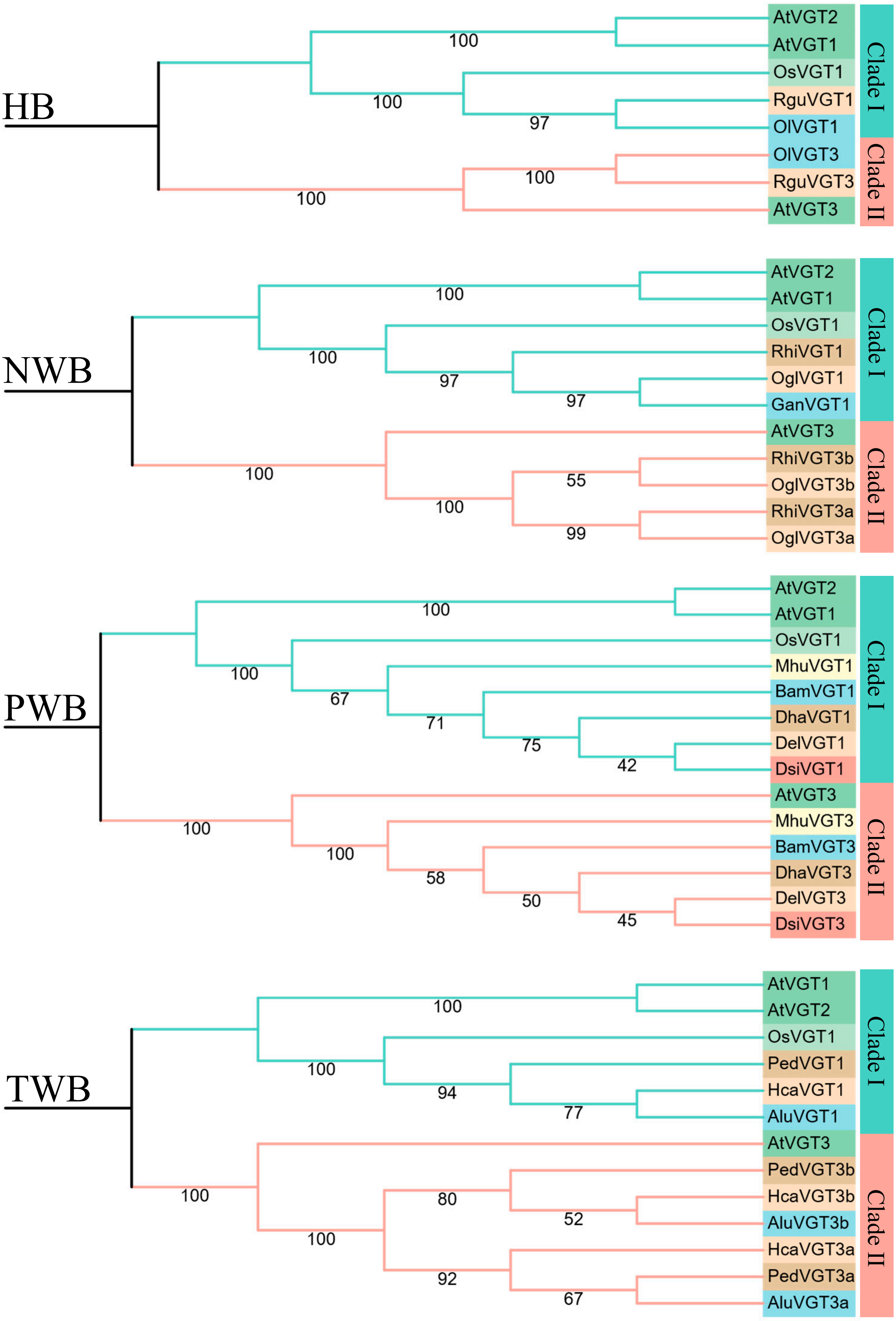

Supplementary Figure S44 Phylogenetic tree of the VGT gene family

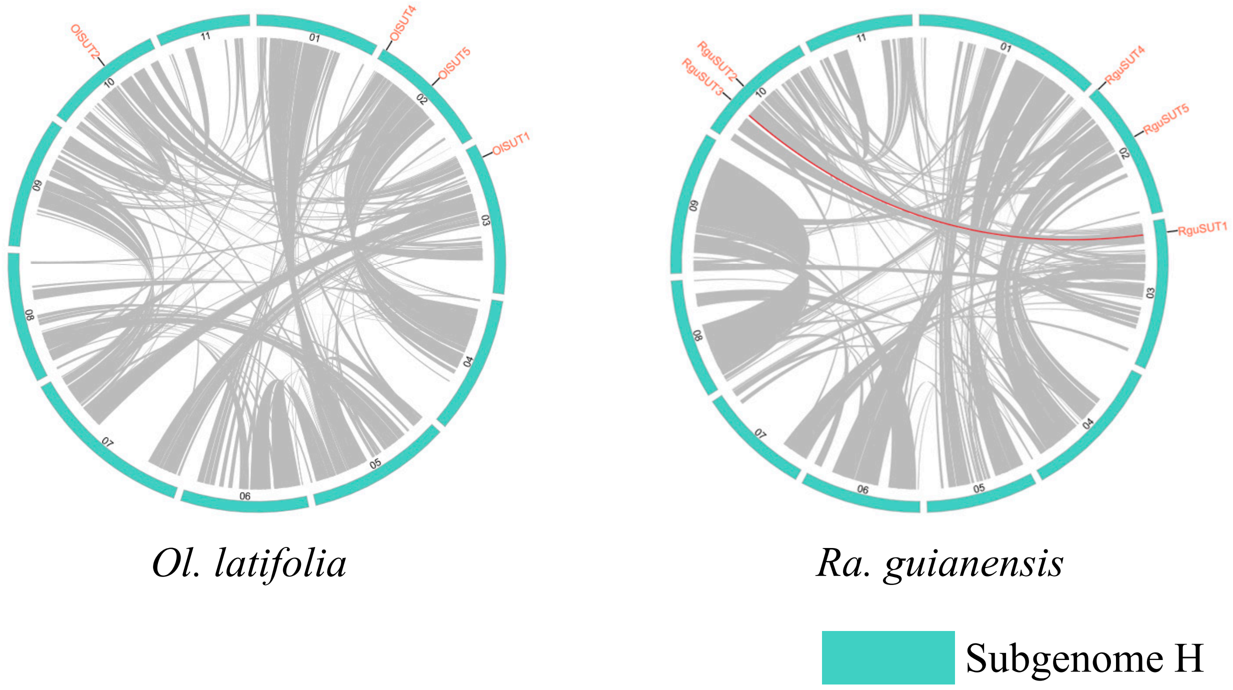

Supplementary Figure S45 Chromosomal location and synteny of the SUT gene family in diploid herbaceous bamboos

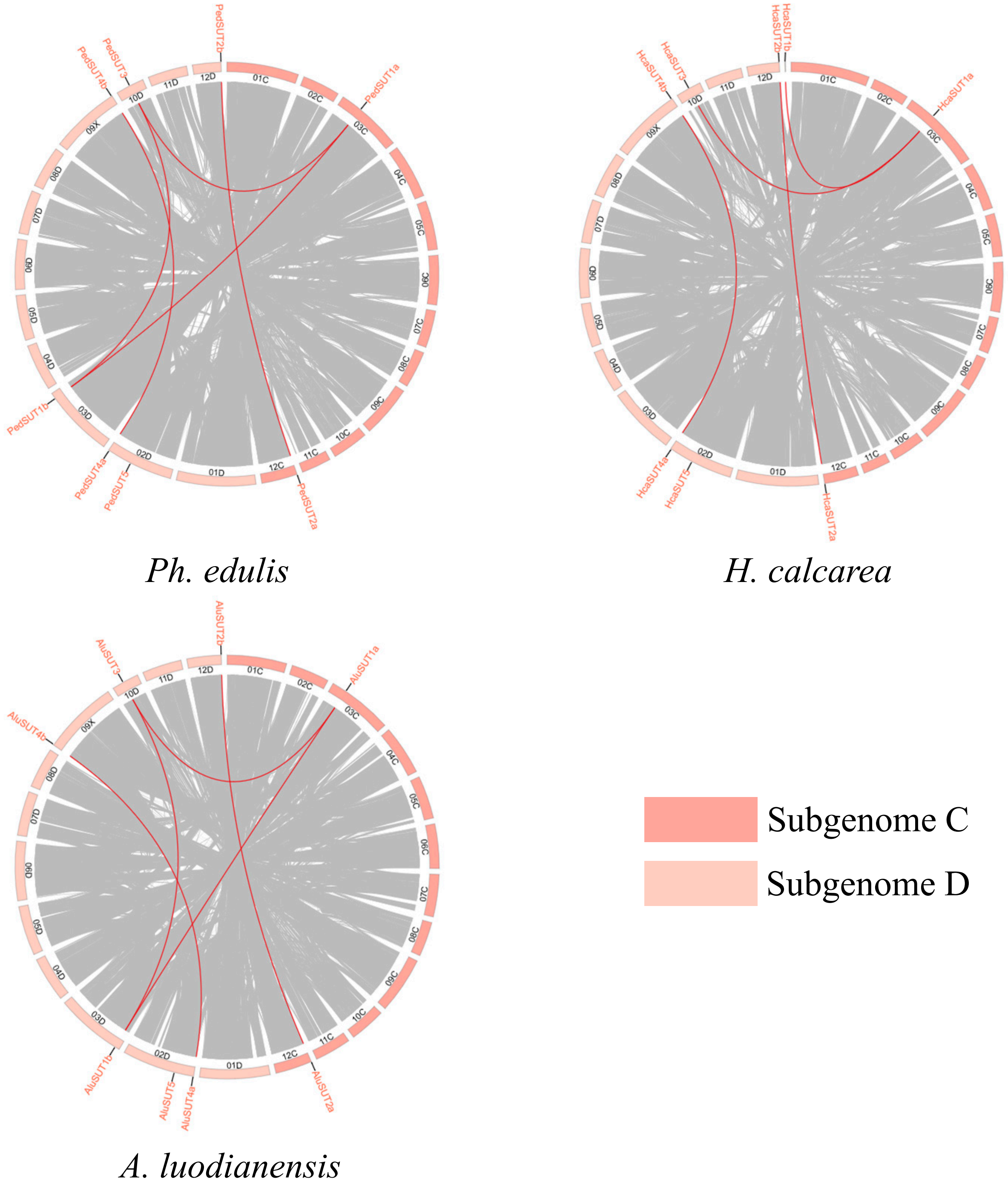

Supplementary Figure S46 Chromosomal location and synteny of the SUT gene family in tetraploid temperate woody bamboos

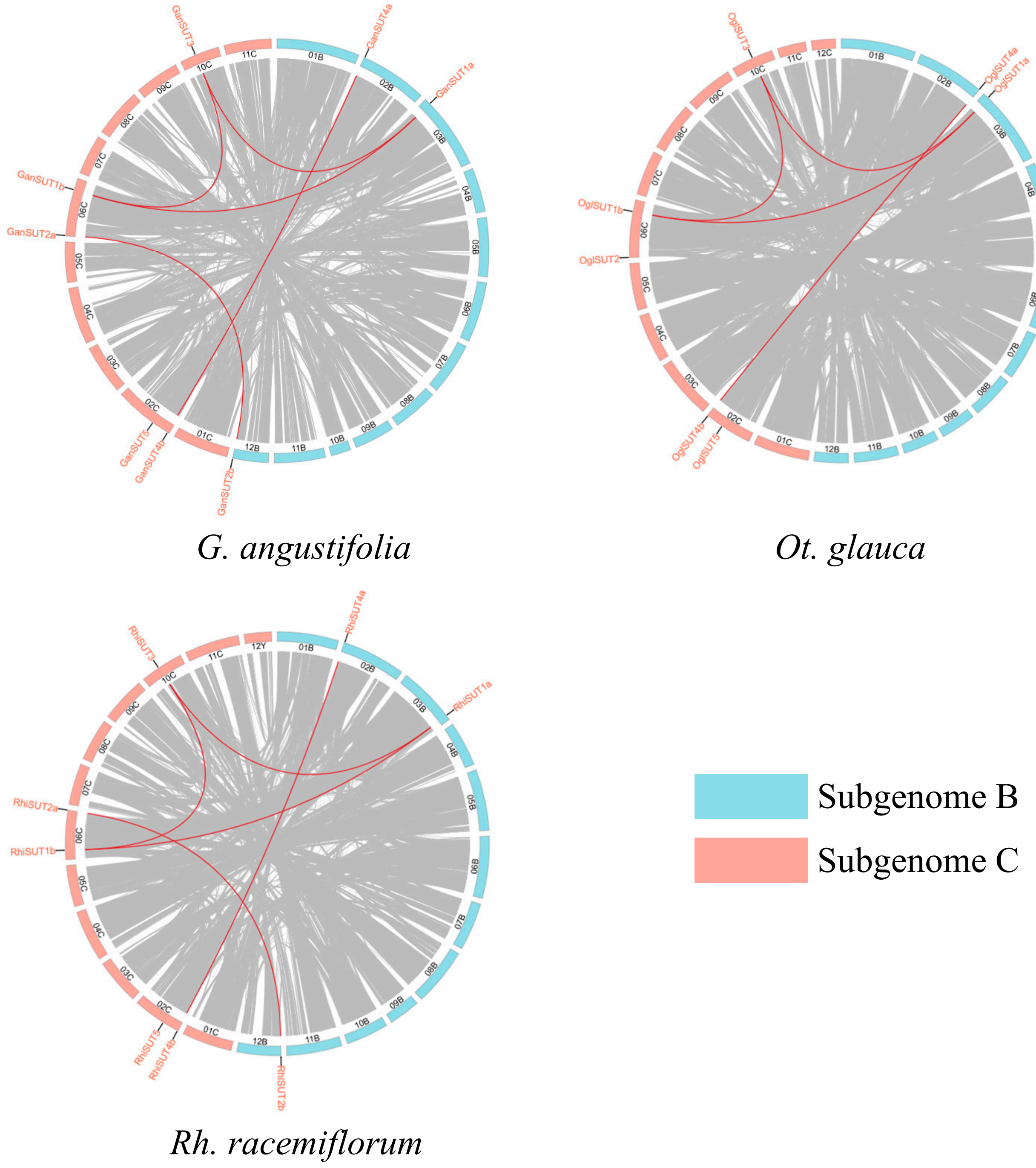

Supplementary Figure S47 Chromosomal location and synteny of the SUT gene family in tetraploid neotropical woody bamboos

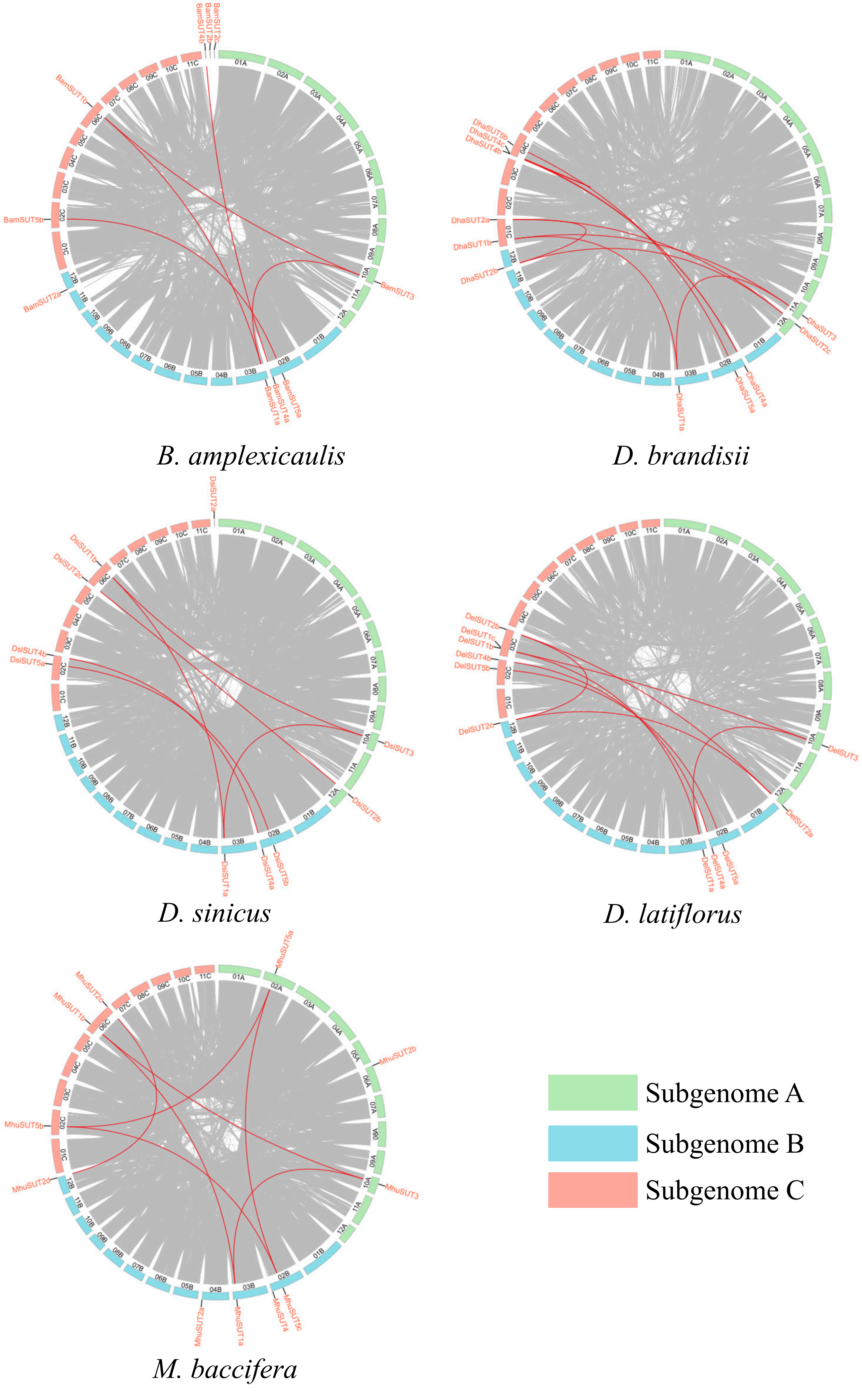

Supplementary Figure S48 Chromosomal location and synteny of the SUT gene family in hexaploid paleotropical woody bamboos

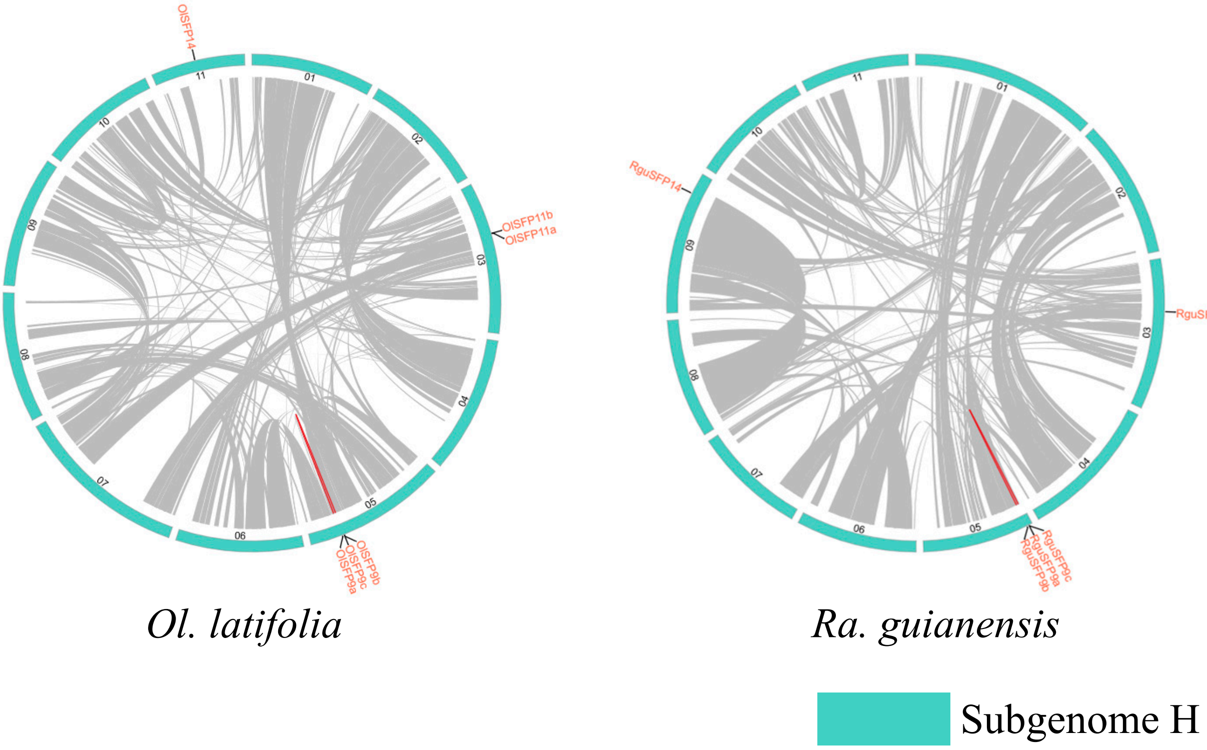

Supplementary Figure S49 Chromosomal location and synteny of the ERDL6 gene family in diploid herbaceous bamboos

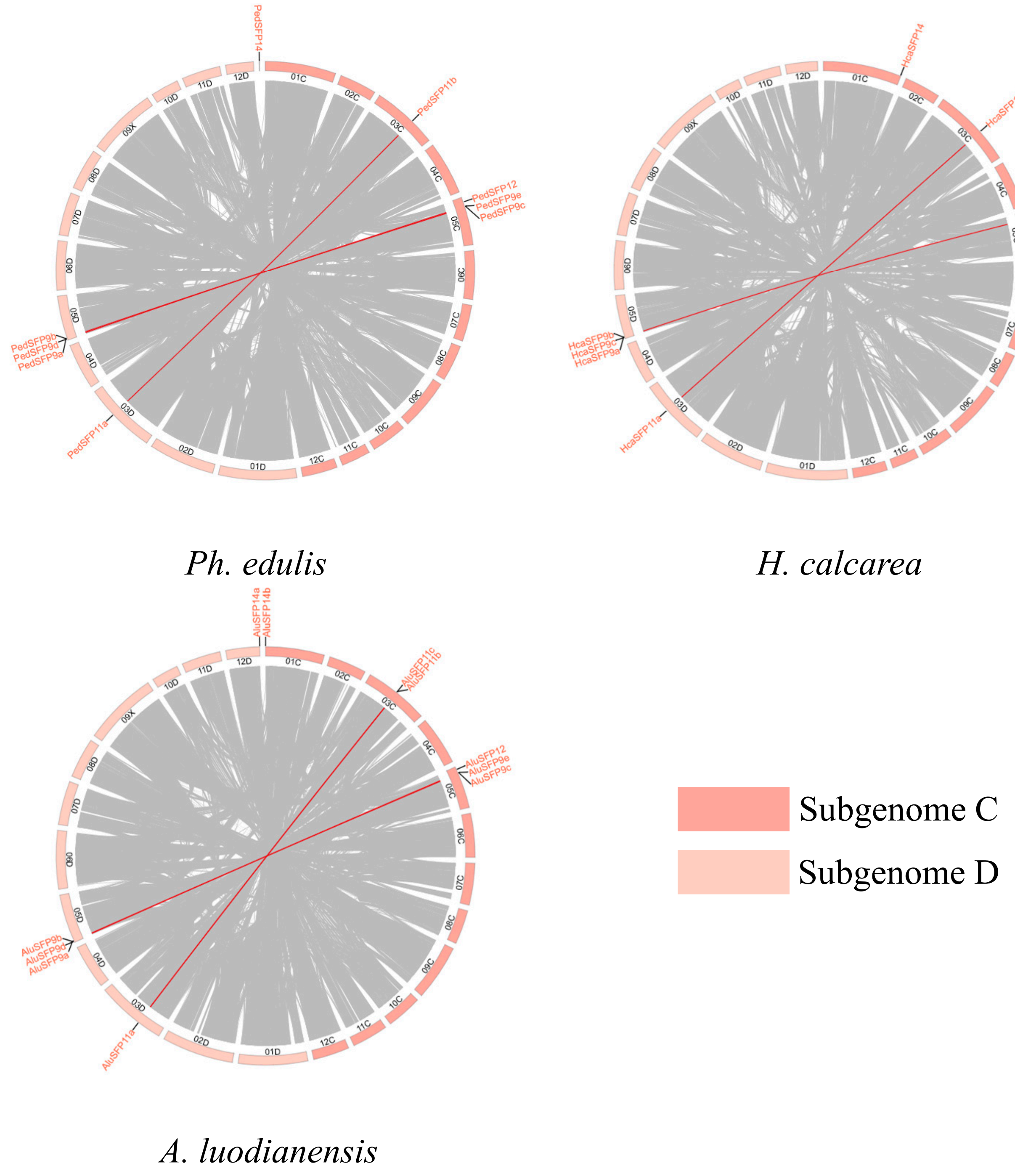

Supplementary Figure S50 Chromosomal location and synteny of the ERDL6 gene family in tetraploid temperate woody bamboos

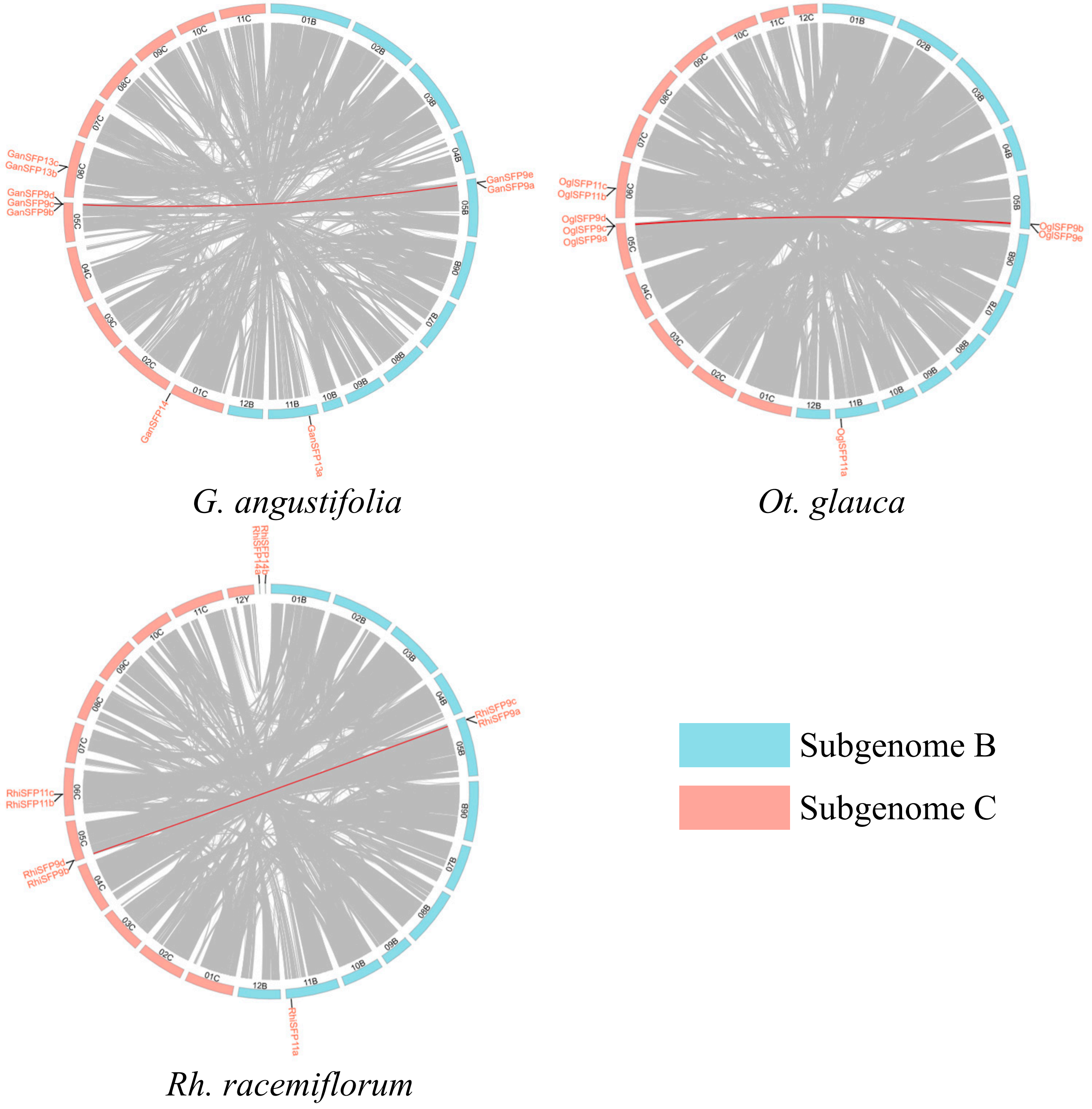

Supplementary Figure S51 Chromosomal location and synteny of the ERDL6 gene family in tetraploid neotropical woody bamboos

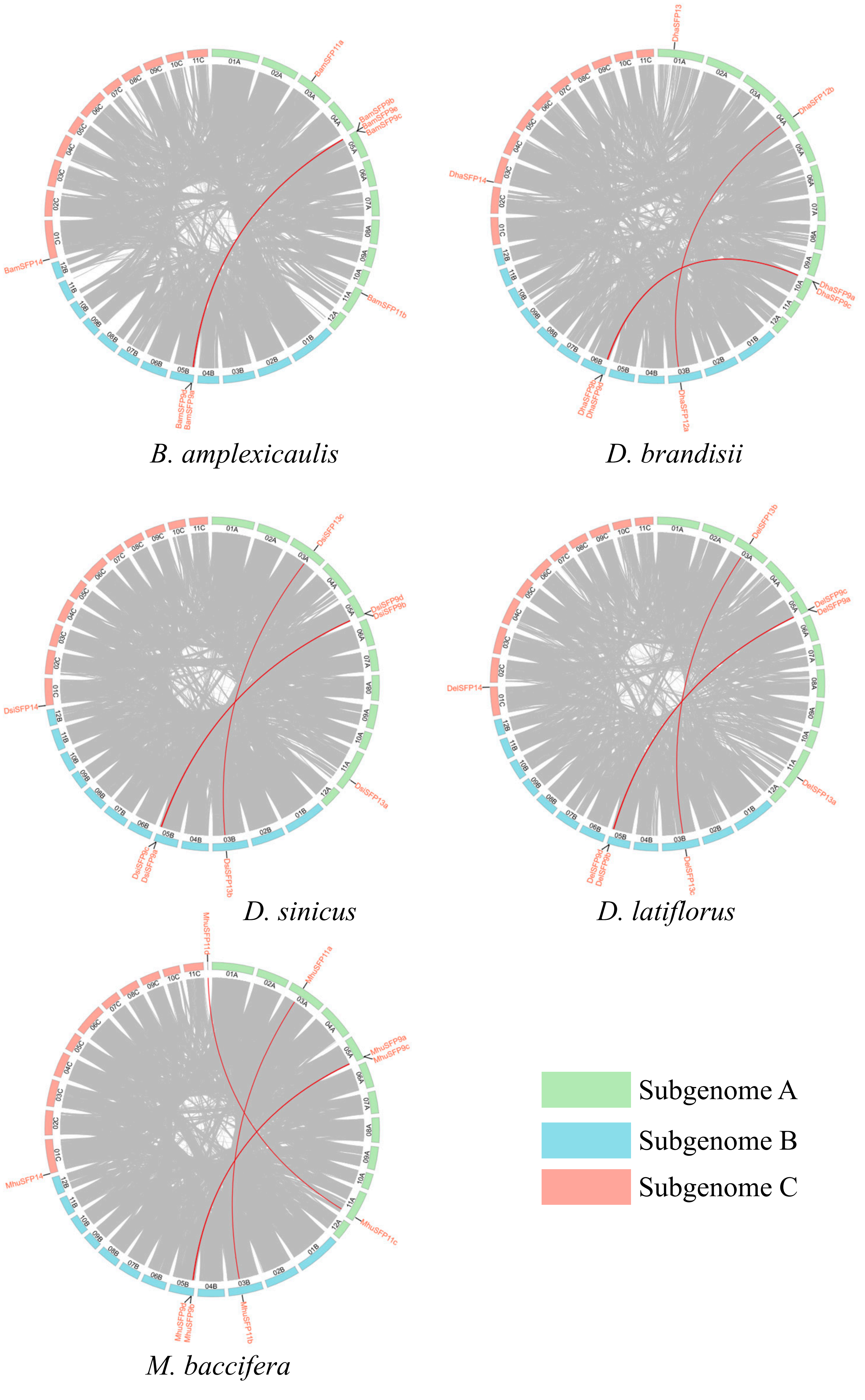

Supplementary Figure S52 Chromosomal location and synteny of the ERDL6 gene family in hexaploid paleotropical woody bamboos

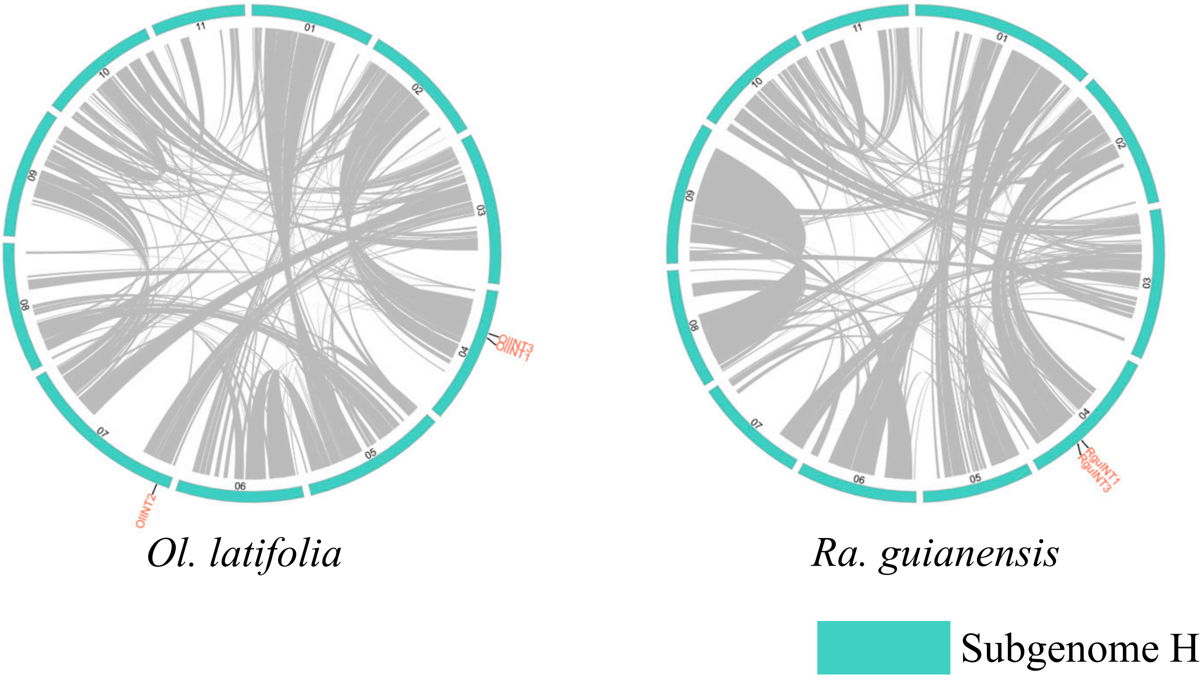

Supplementary Figure S53 Chromosomal location and synteny of the INT gene family in diploid herbaceous bamboos

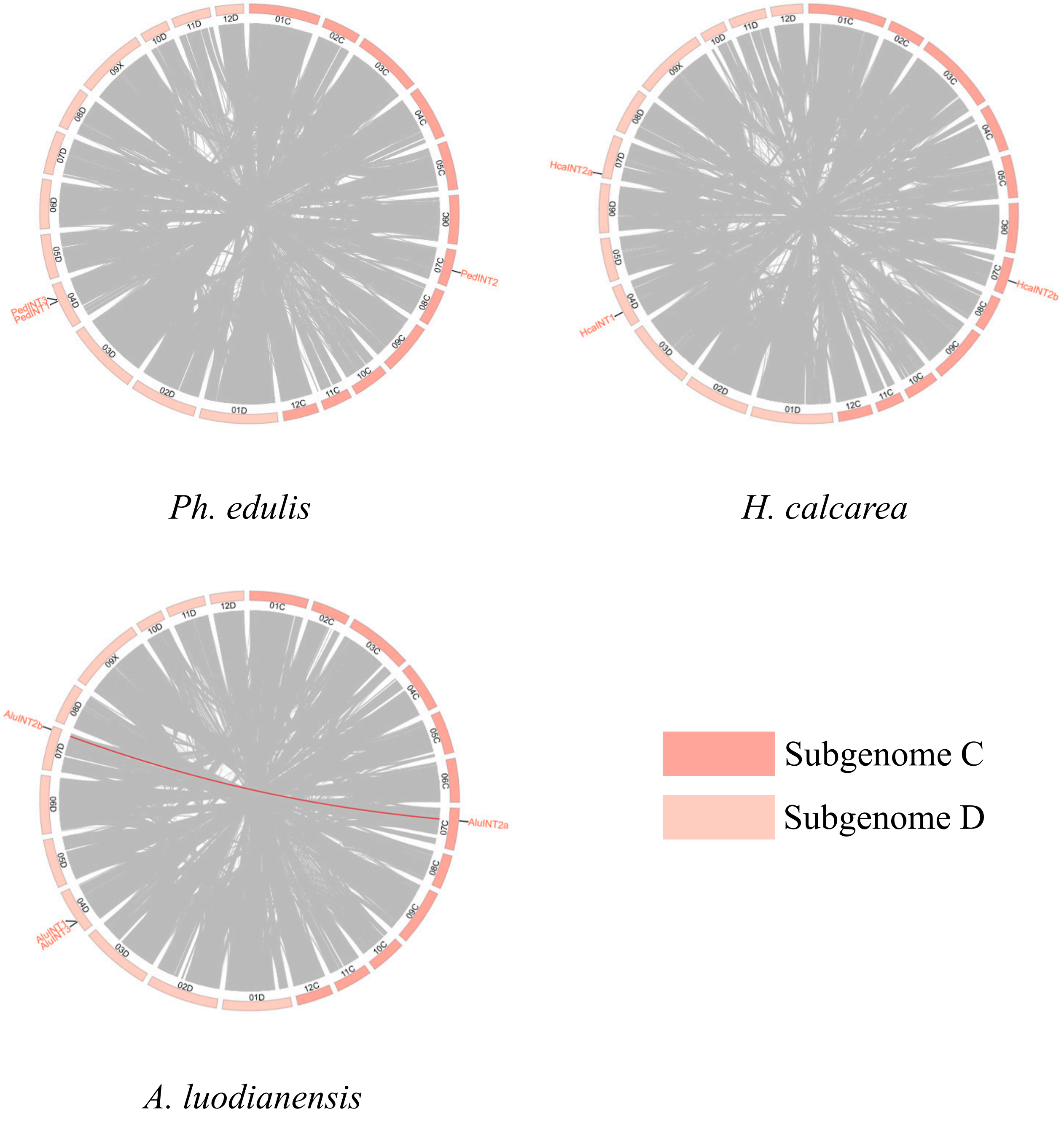

Supplementary Figure S54 Chromosomal location and synteny of the INT gene family in tetraploid temperate woody bamboos

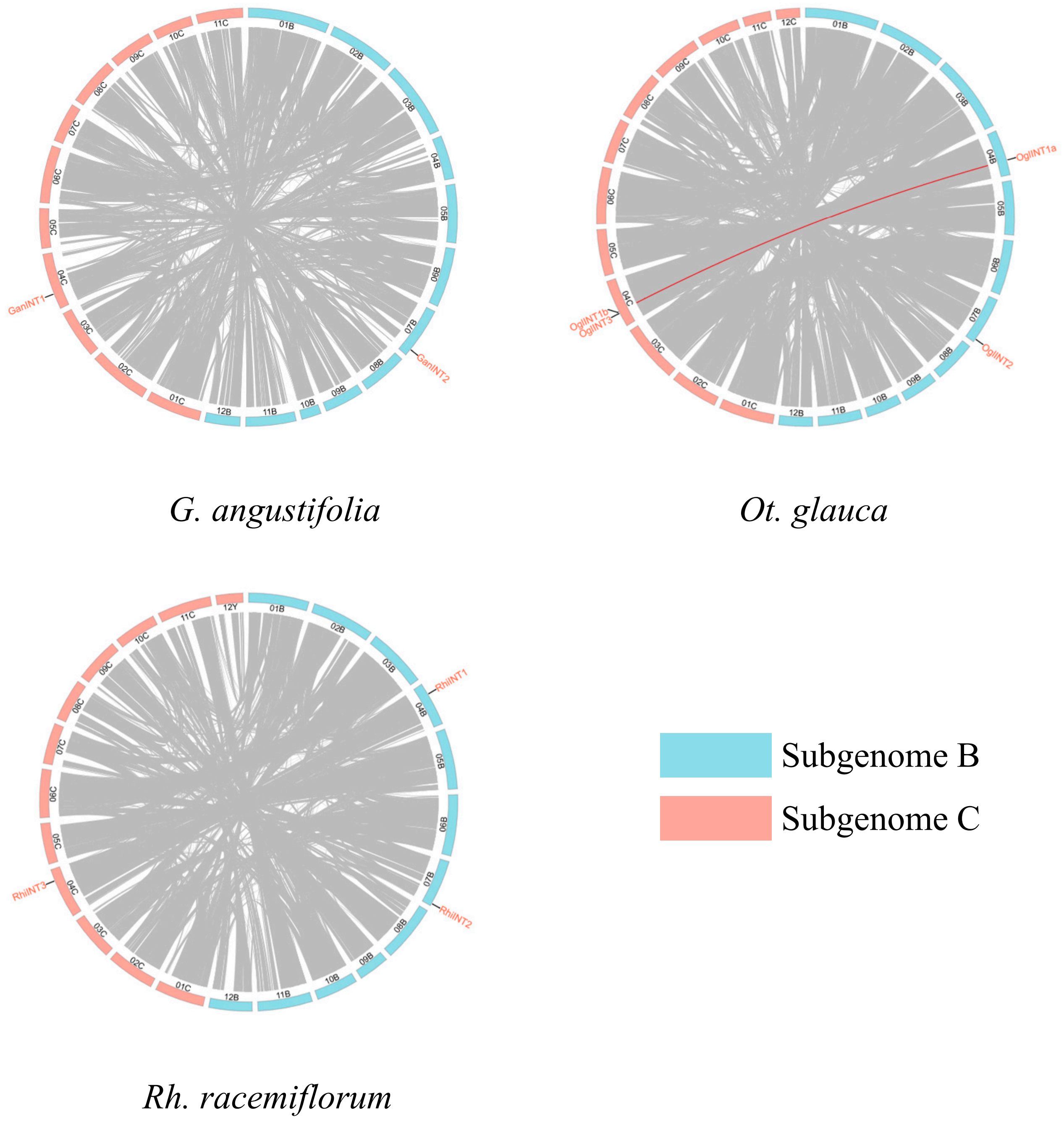

Supplementary Figure S55 Chromosomal location and synteny of the INT gene family in tetraploid neotropical woody bamboos

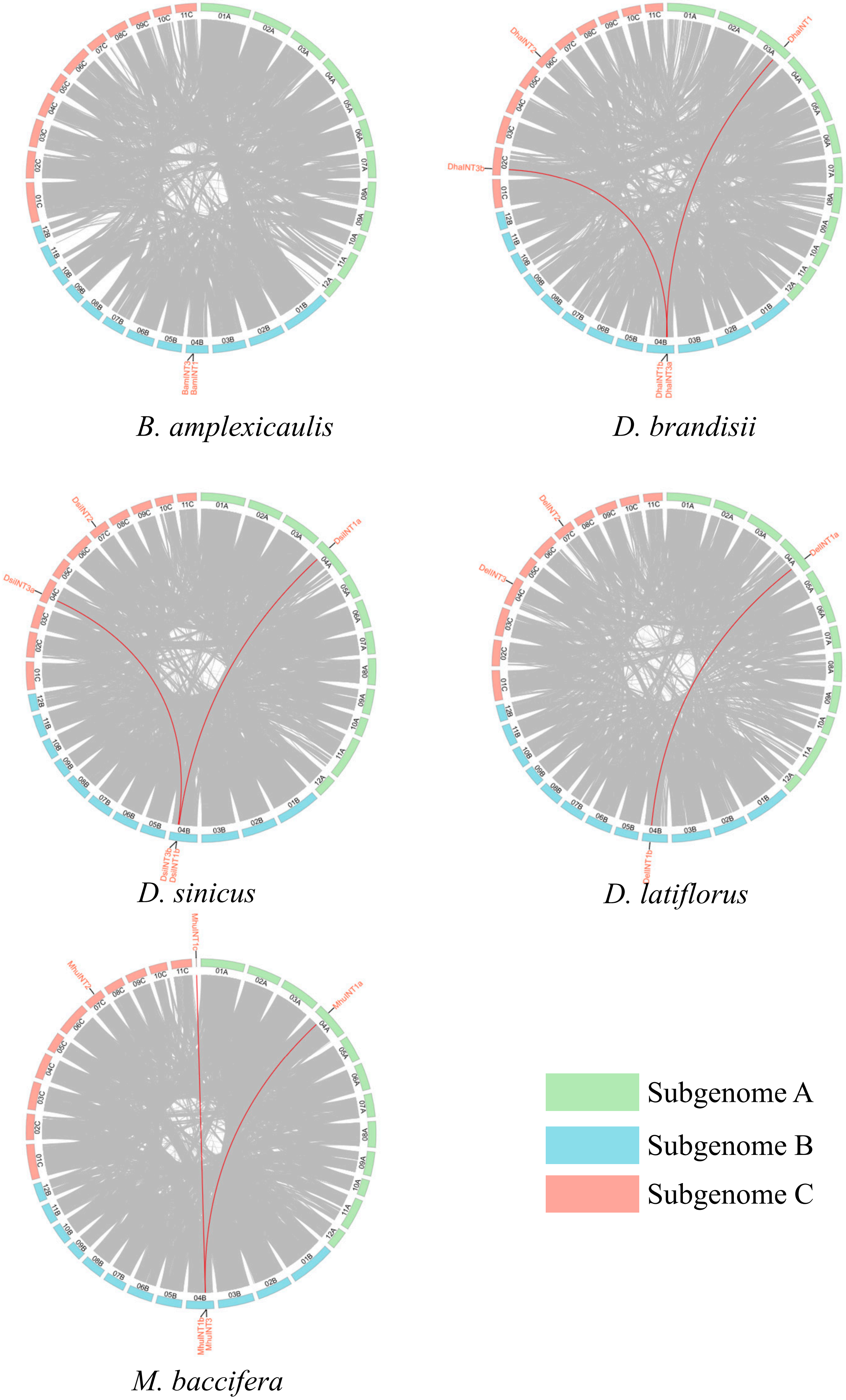

Supplementary Figure S56 Chromosomal location and synteny of the INT gene family in hexaploid paleotropical woody bamboos

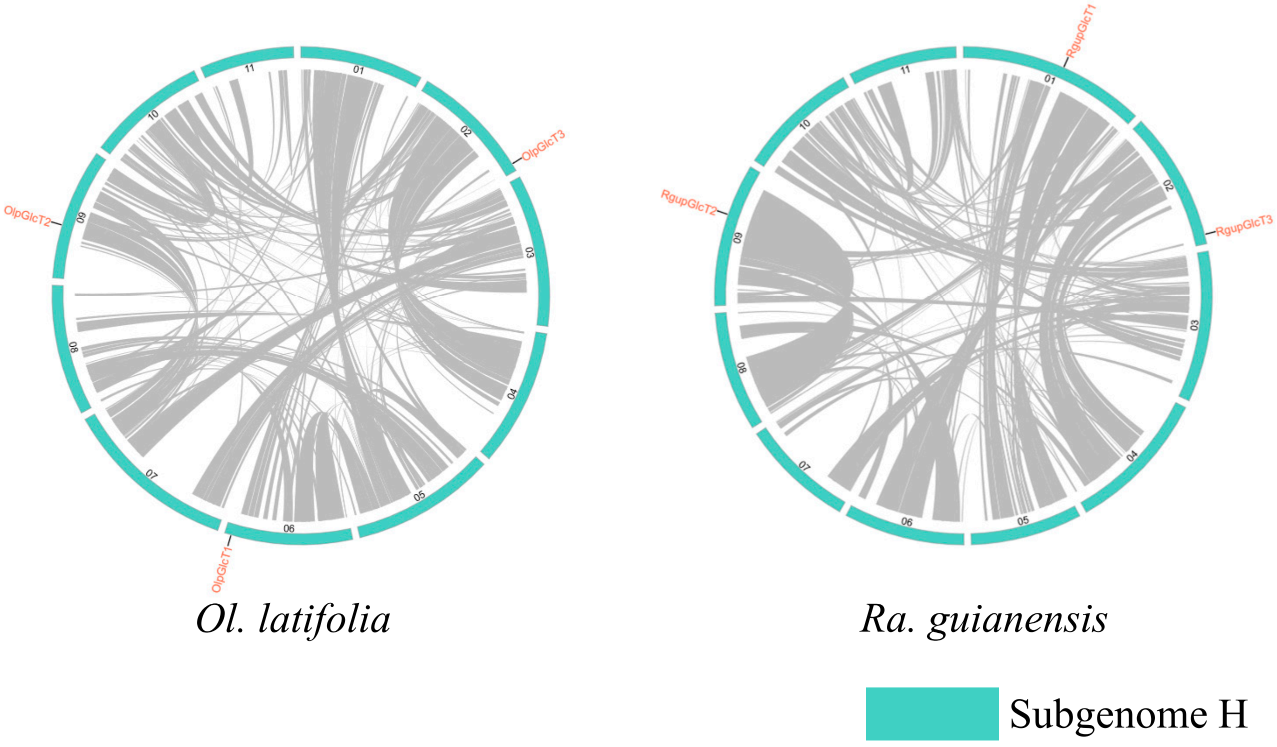

Supplementary Figure S57 Chromosomal location and synteny of the pGlcT gene family in diploid herbaceous bamboos

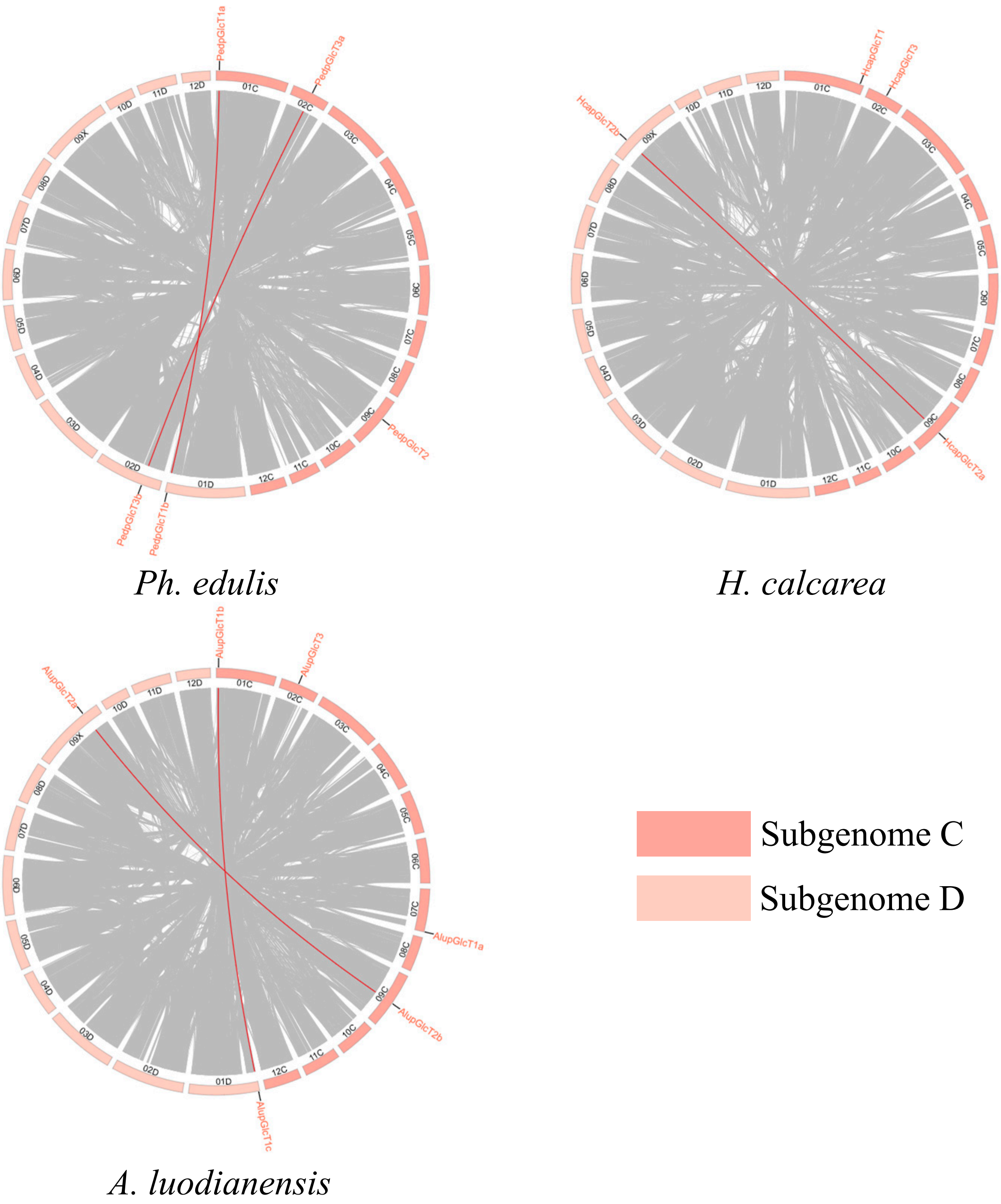

Supplementary Figure S58 Chromosomal location and synteny of the pGlcT gene family in tetraploid temperate woody bamboos

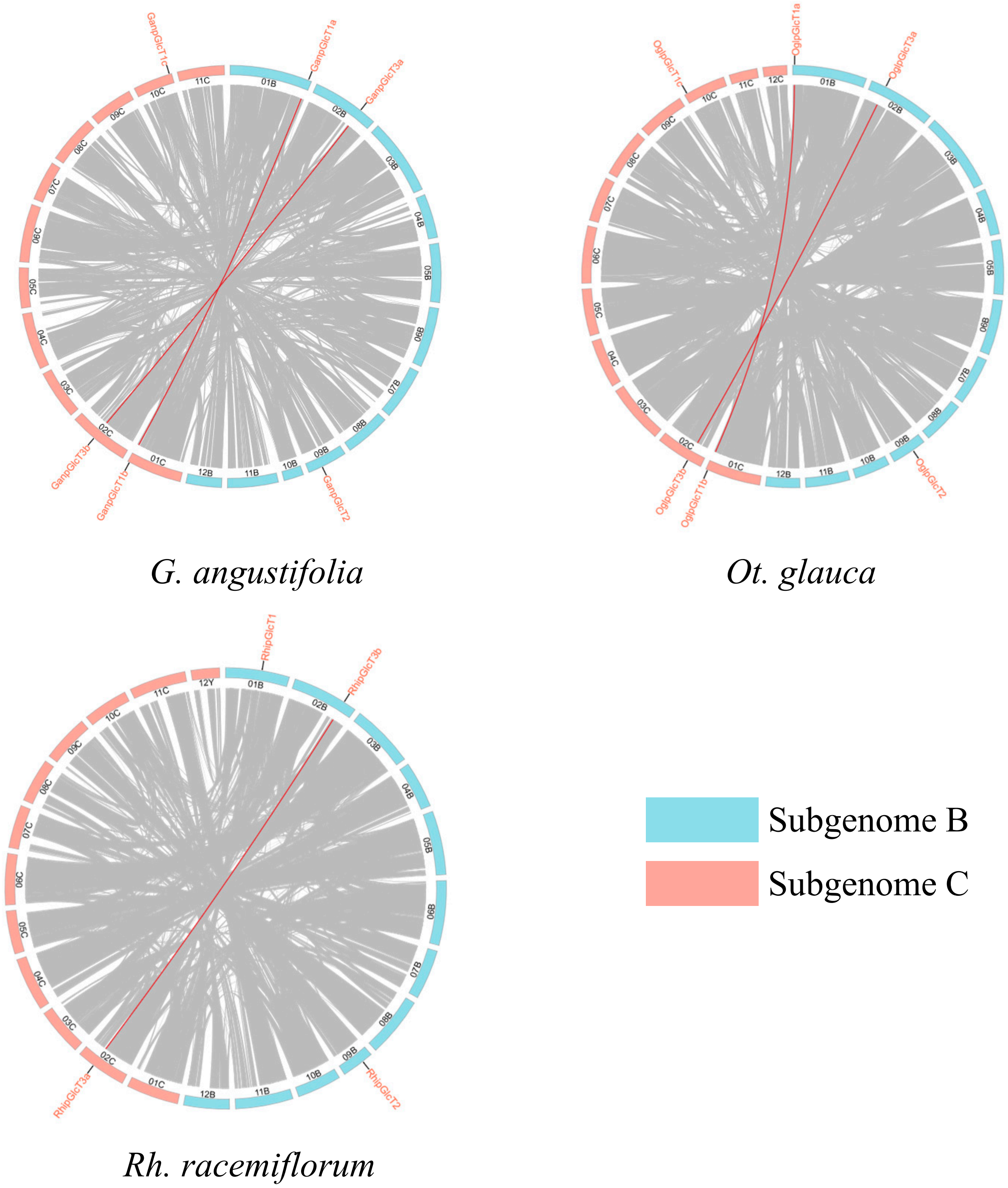

Supplementary Figure S59 Chromosomal location and synteny of the pGlcT gene family in tetraploid neotropical woody bamboos

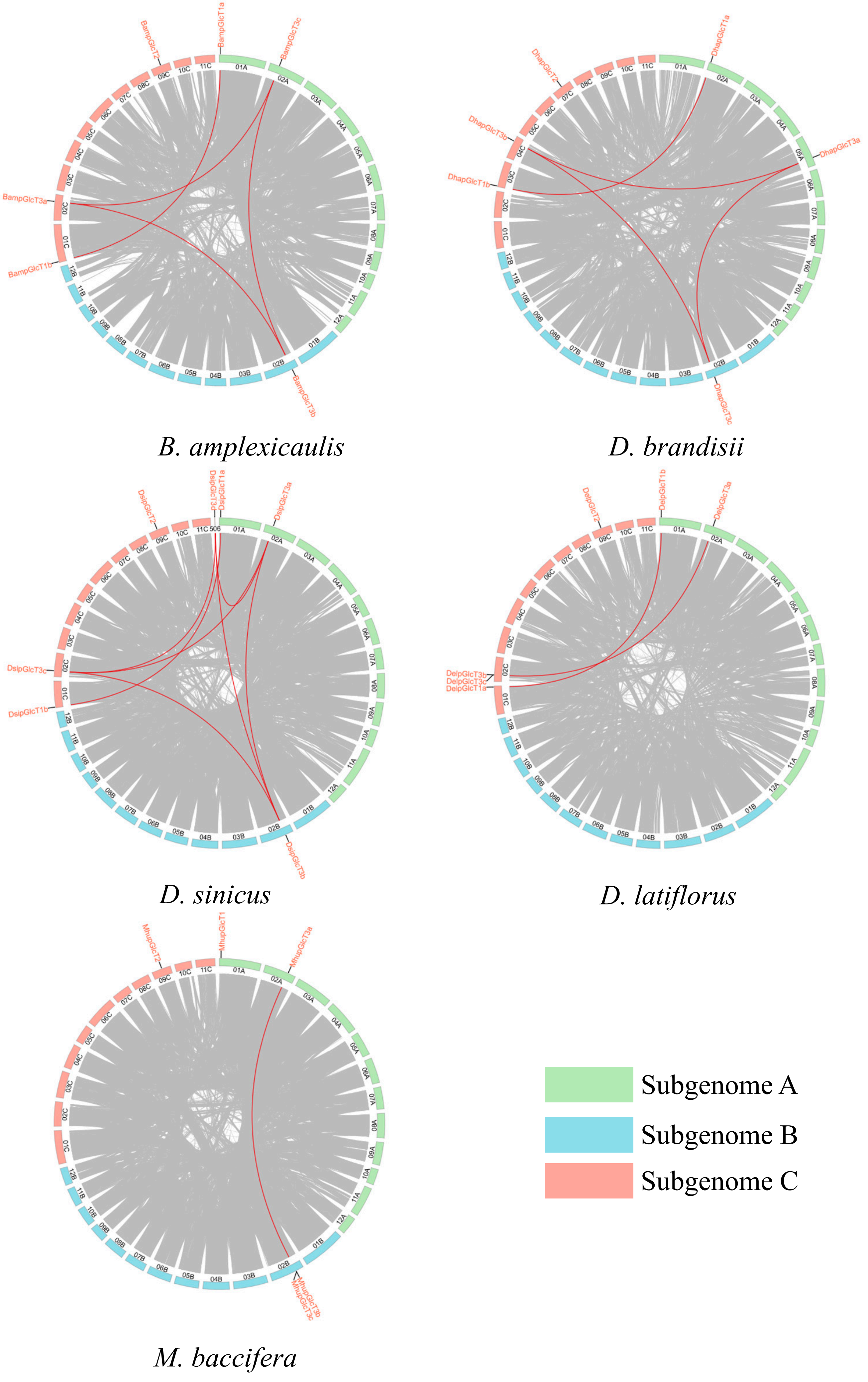

Supplementary Figure S60 Chromosomal location and synteny of the pGlcT gene family in hexaploid paleotropical woody bamboos

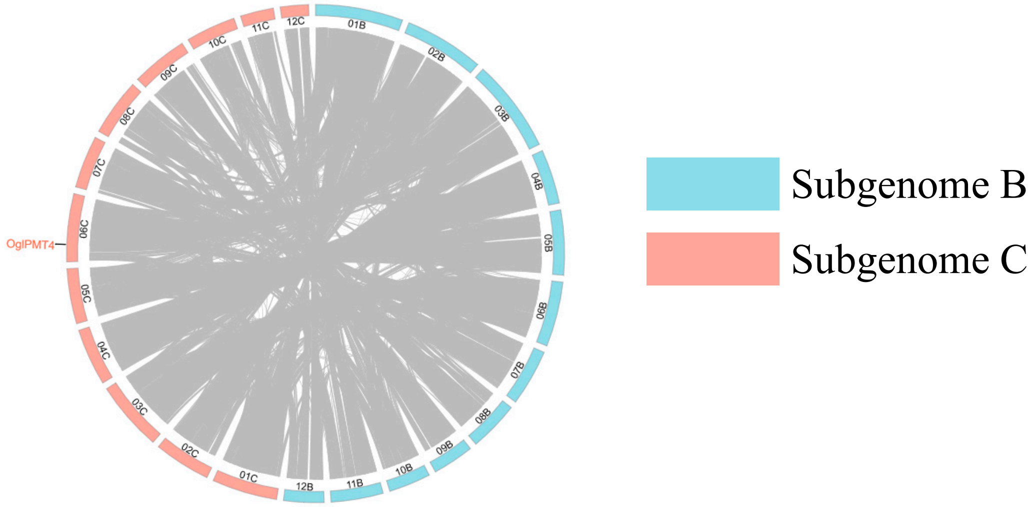

Ot. glauca

Supplementary Figure S61 Chromosomal location and synteny of the PMT gene family in tetraploid neotropical woody bamboos

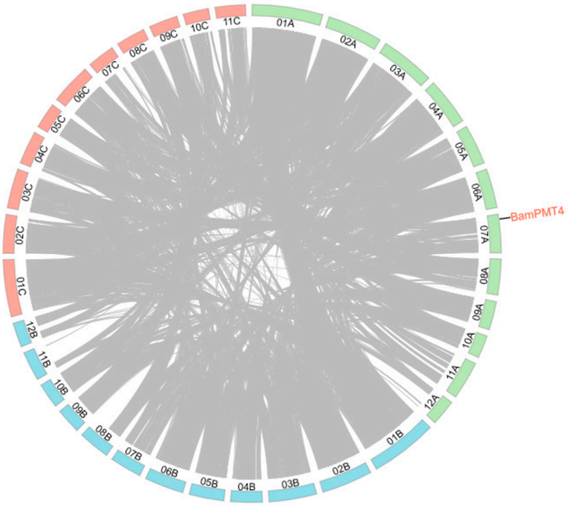

B. amplexicaulis

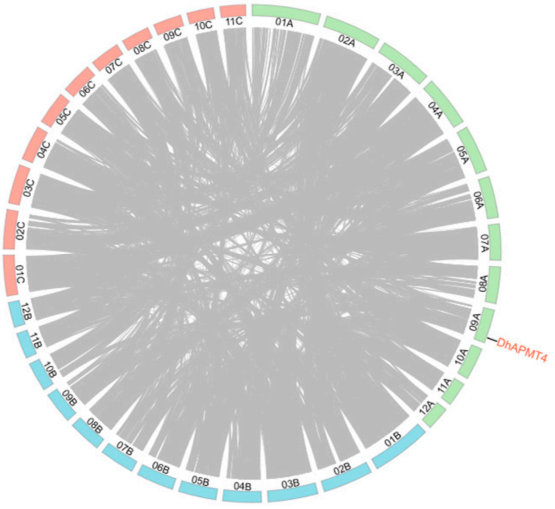

D. brandisii

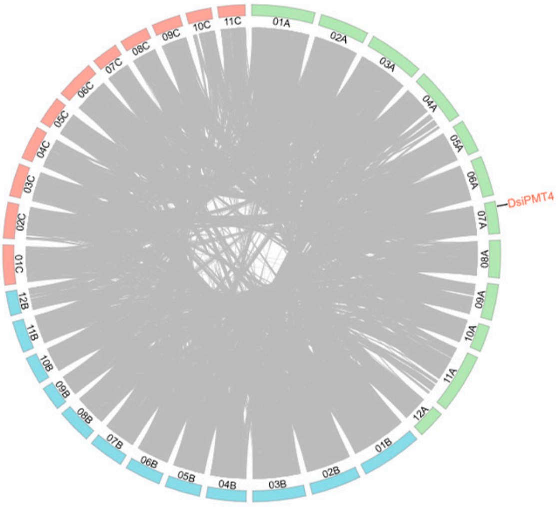

D. sinicus

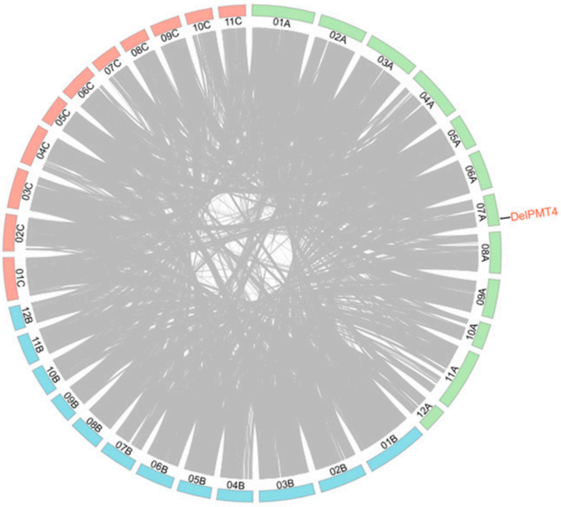

D. latiflorus

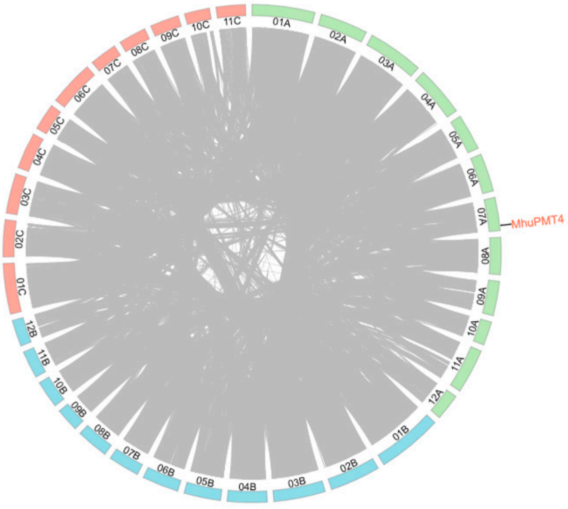

M. baccifera

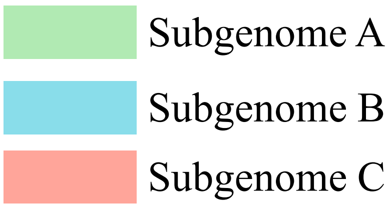

Supplementary Figure S62 Chromosomal location and synteny of the PMT gene family in hexaploid paleotropical woody bamboos

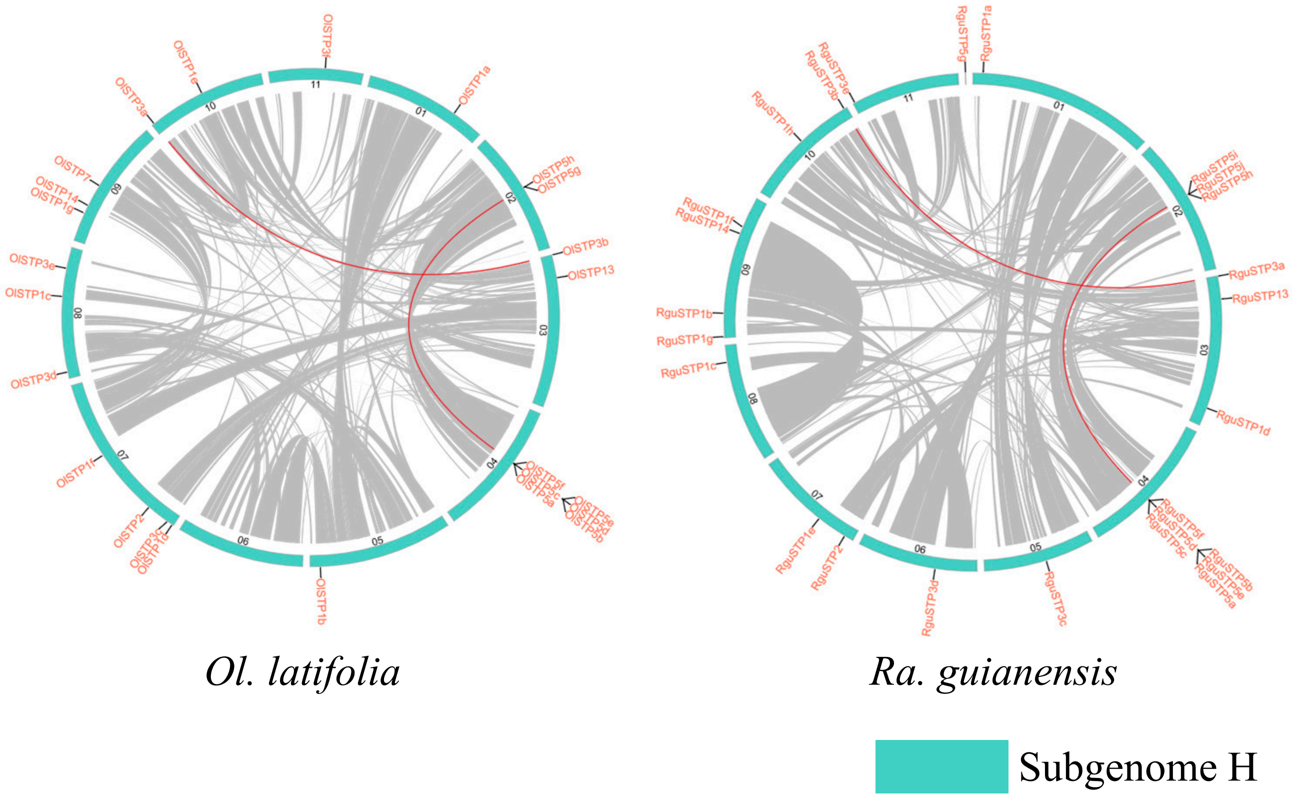

Supplementary Figure S63 Chromosomal location and synteny of the STP gene family in diploid herbaceous bamboos

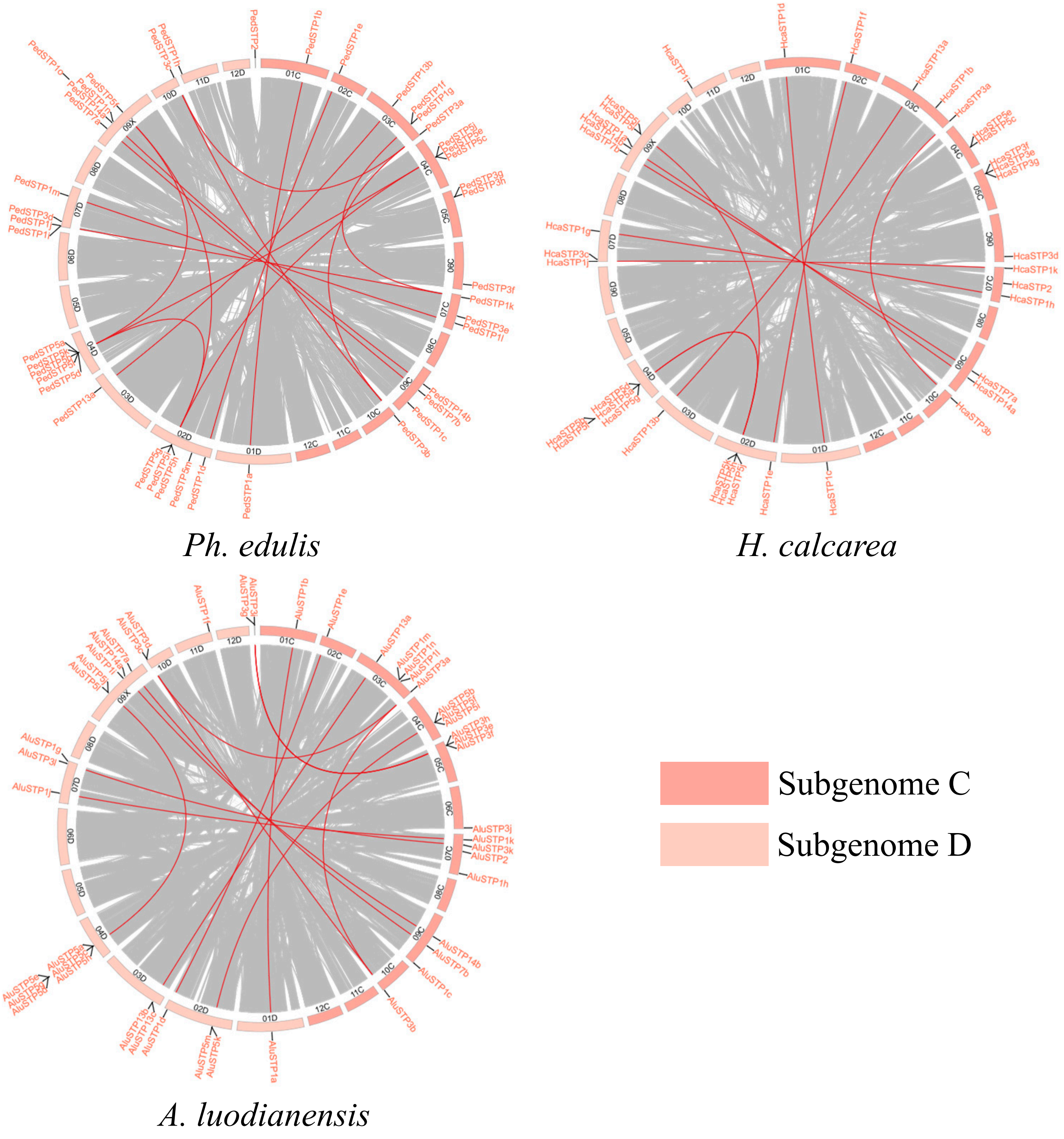

Supplementary Figure S64 Chromosomal location and synteny of the STP gene family in tetraploid temperate woody bamboos

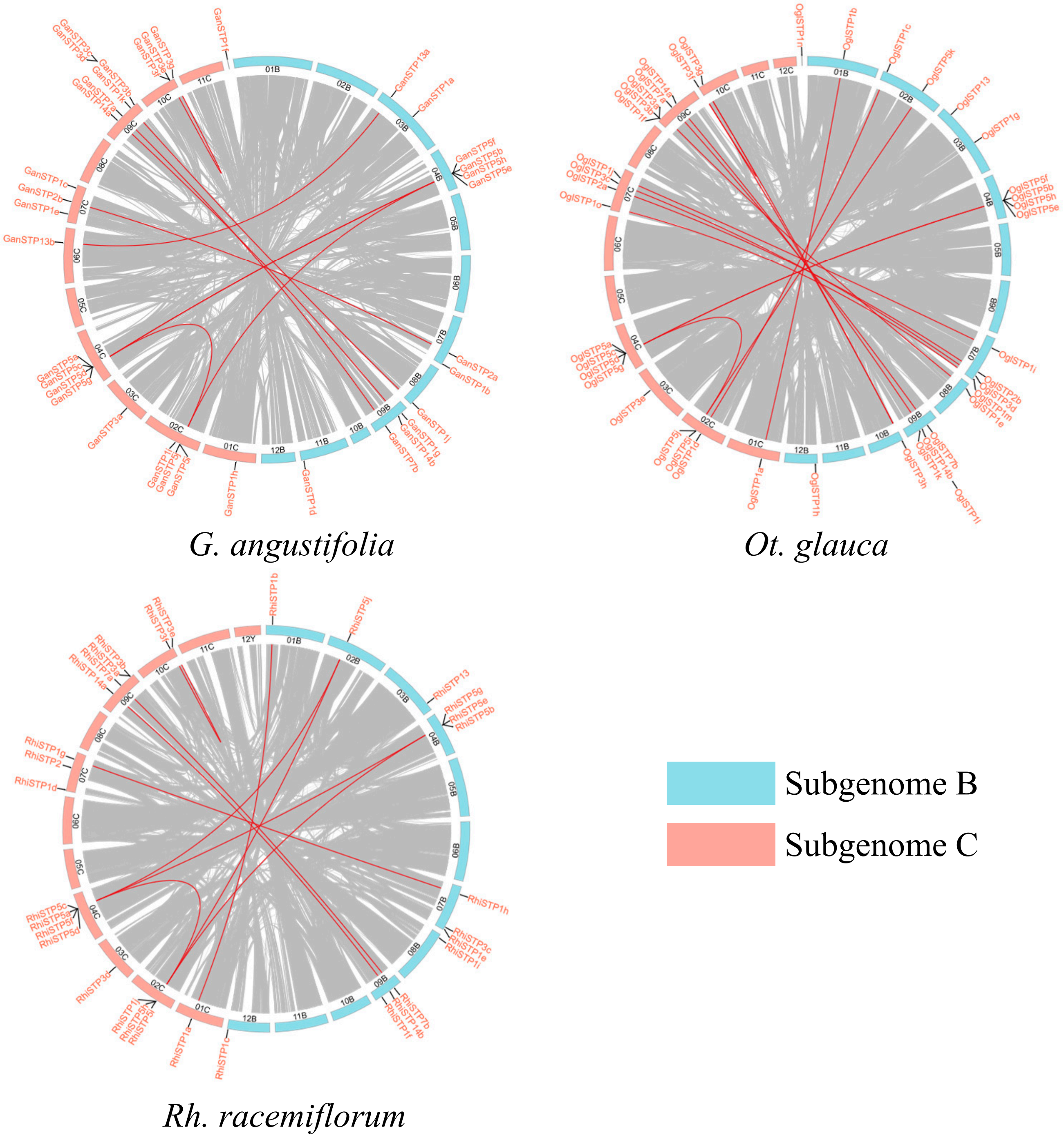

Supplementary Figure S65 Chromosomal location and synteny of the STP gene family in tetraploid neotropical woody bamboos

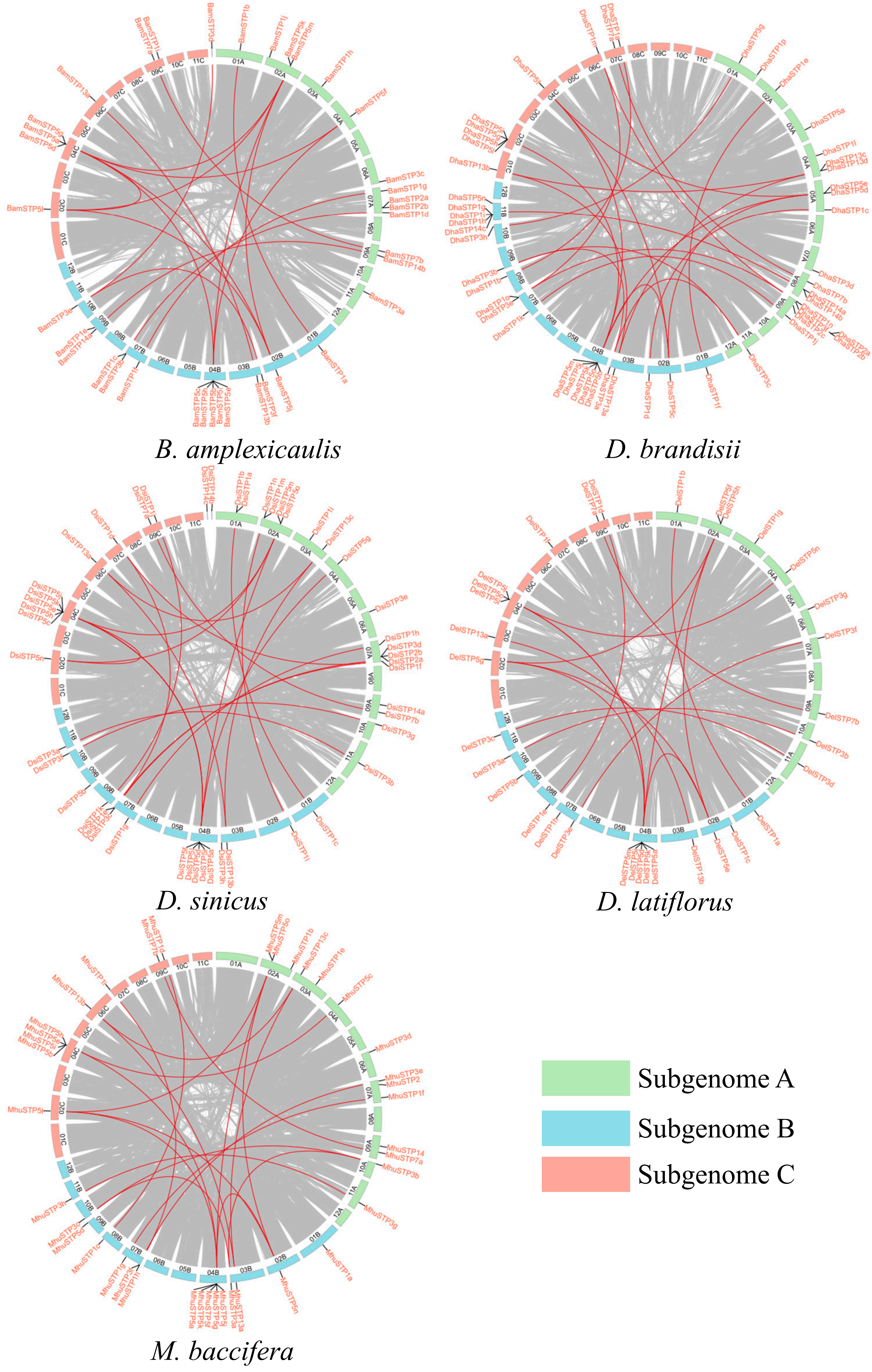

Supplementary Figure S66 Chromosomal location and synteny of the STP gene family in hexaploid paleotropical woody bamboos

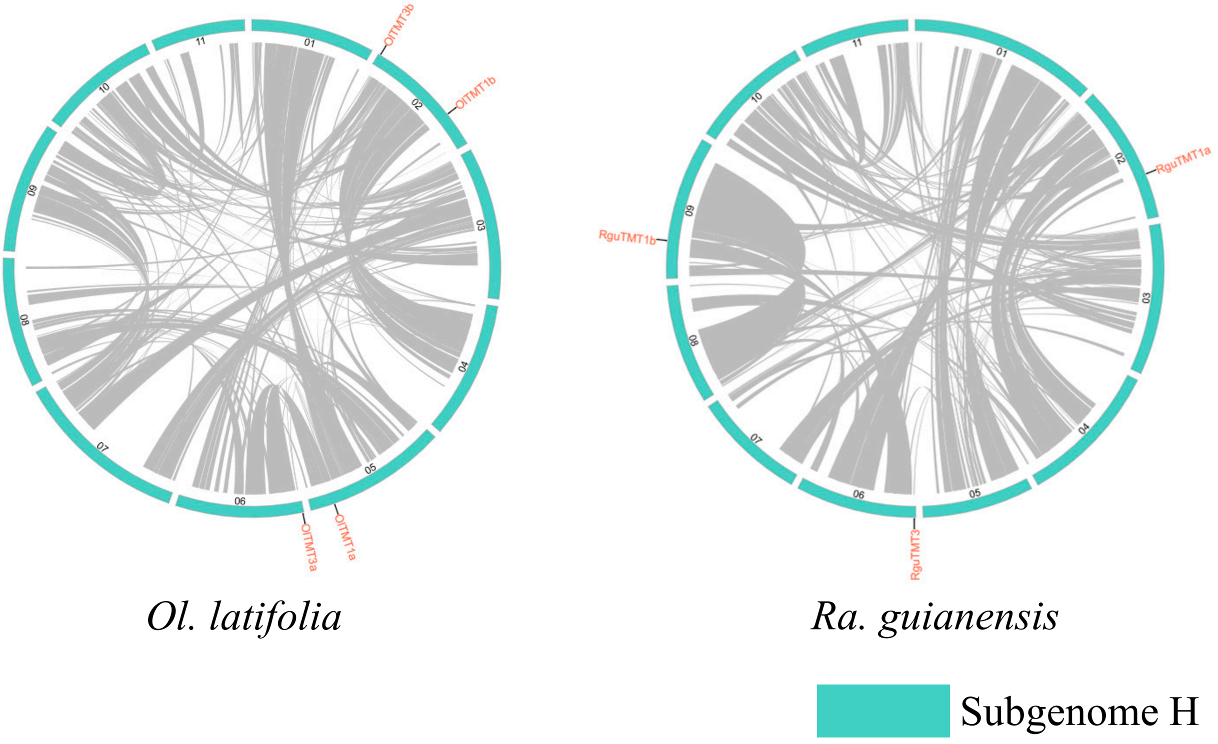

Supplementary Figure S67 Chromosomal location and synteny of the TMT gene family in diploid herbaceous bamboos

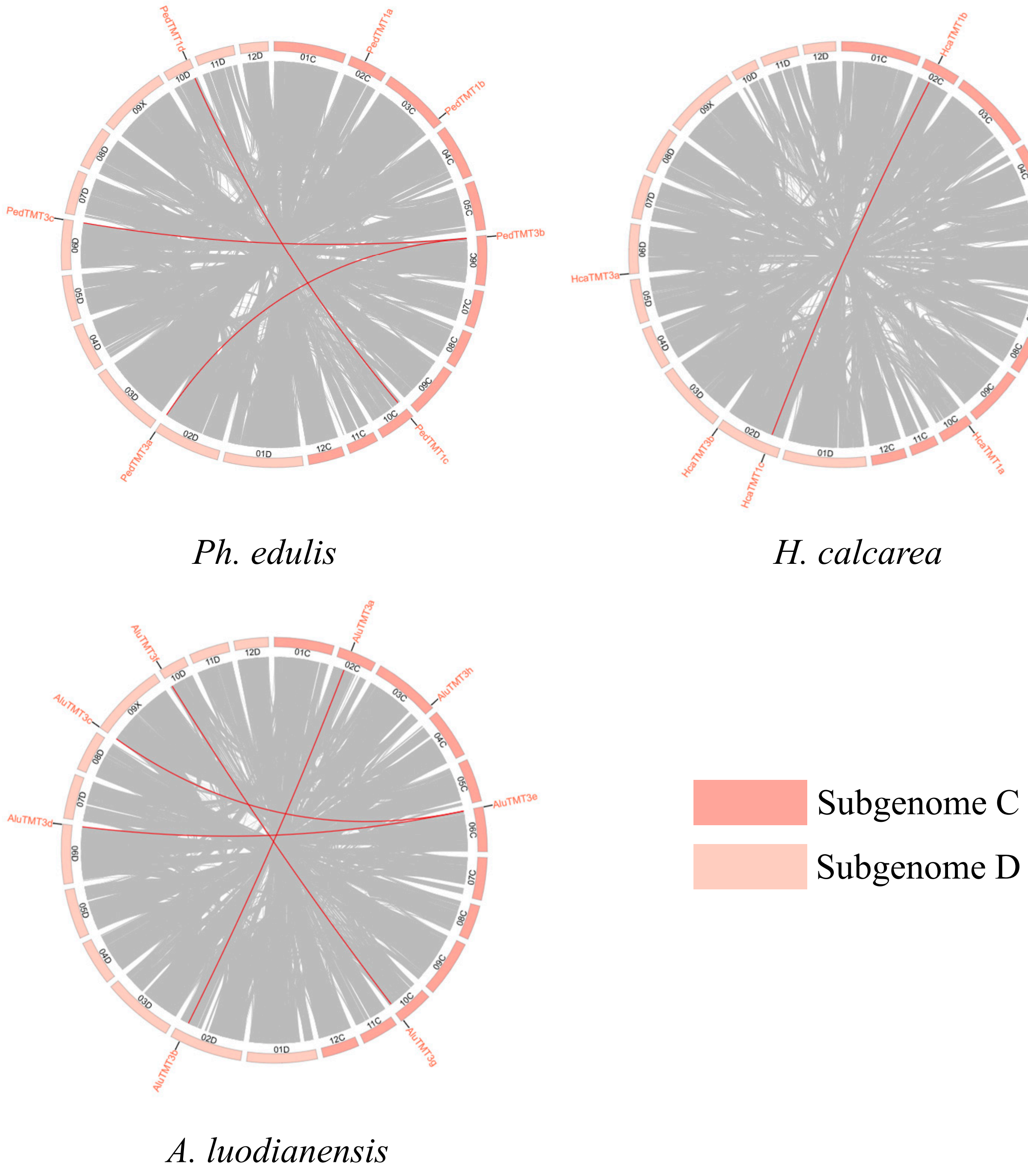

Supplementary Figure S68 Chromosomal location and synteny of the TMT gene family in tetraploid temperate woody bamboos

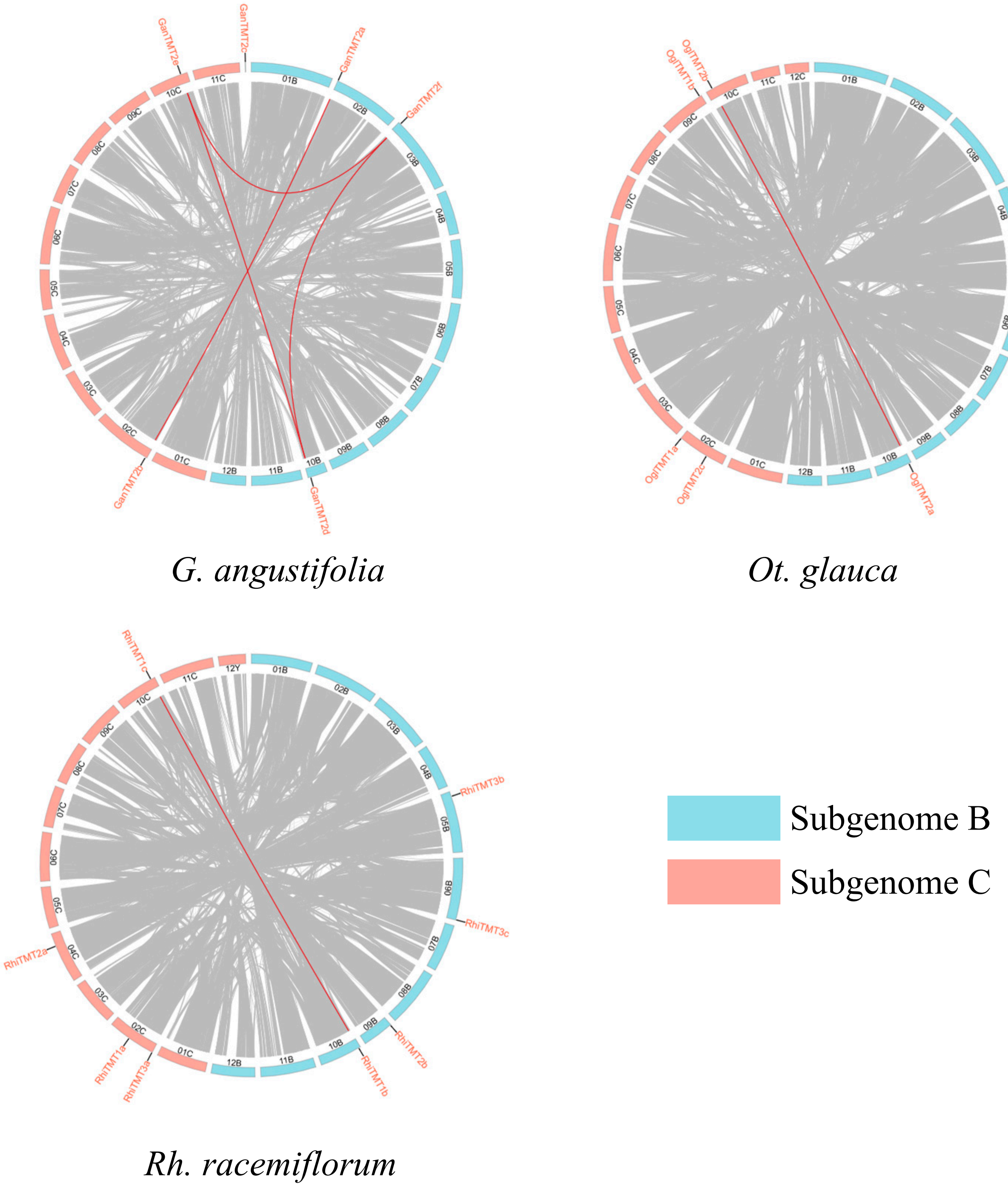

Supplementary Figure S69 Chromosomal location and synteny of the TMT gene family in tetraploid neotropical woody bamboos

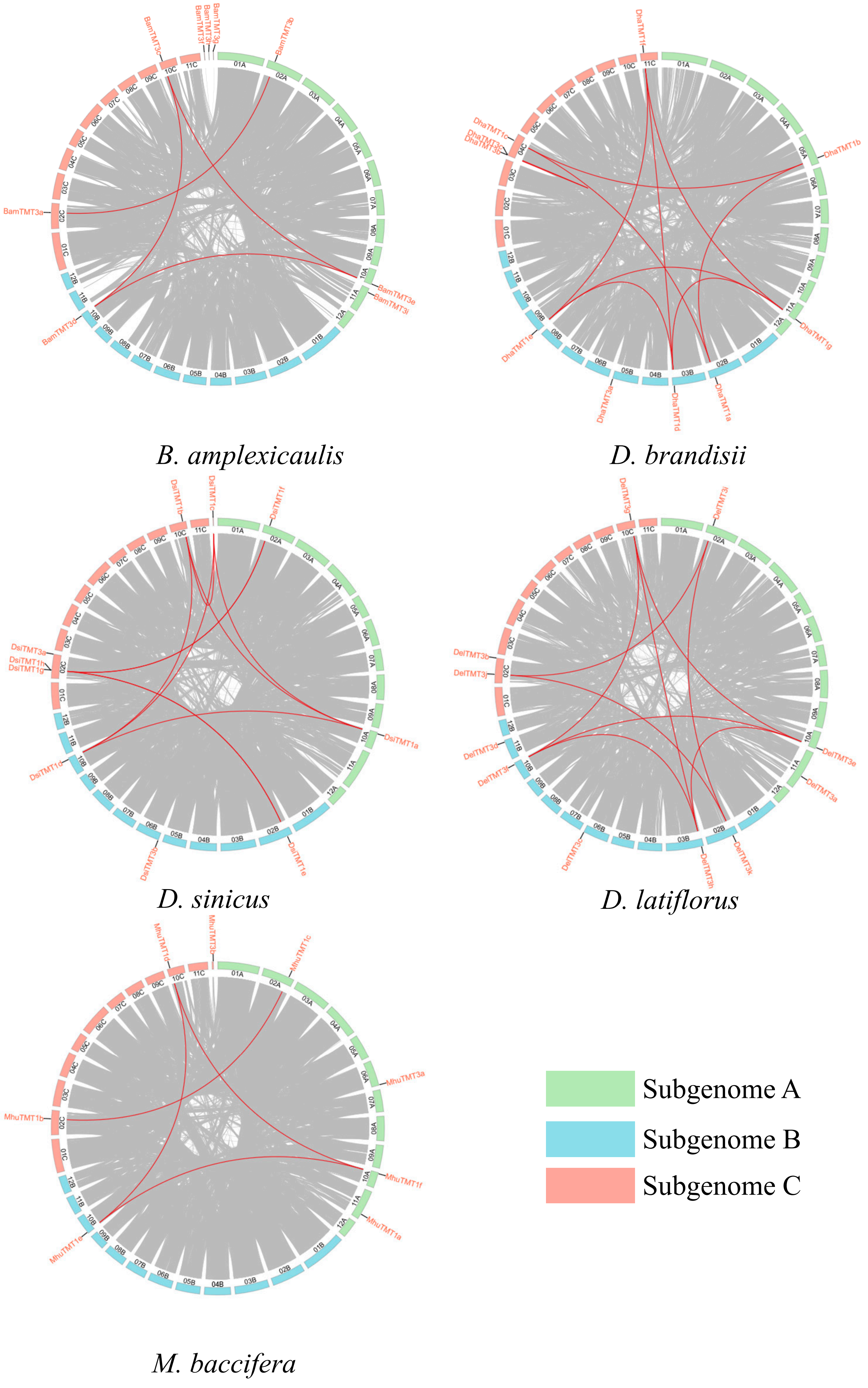

Supplementary Figure S70 Chromosomal location and synteny of the TMT gene family in hexaploid paleotropical woody bamboos

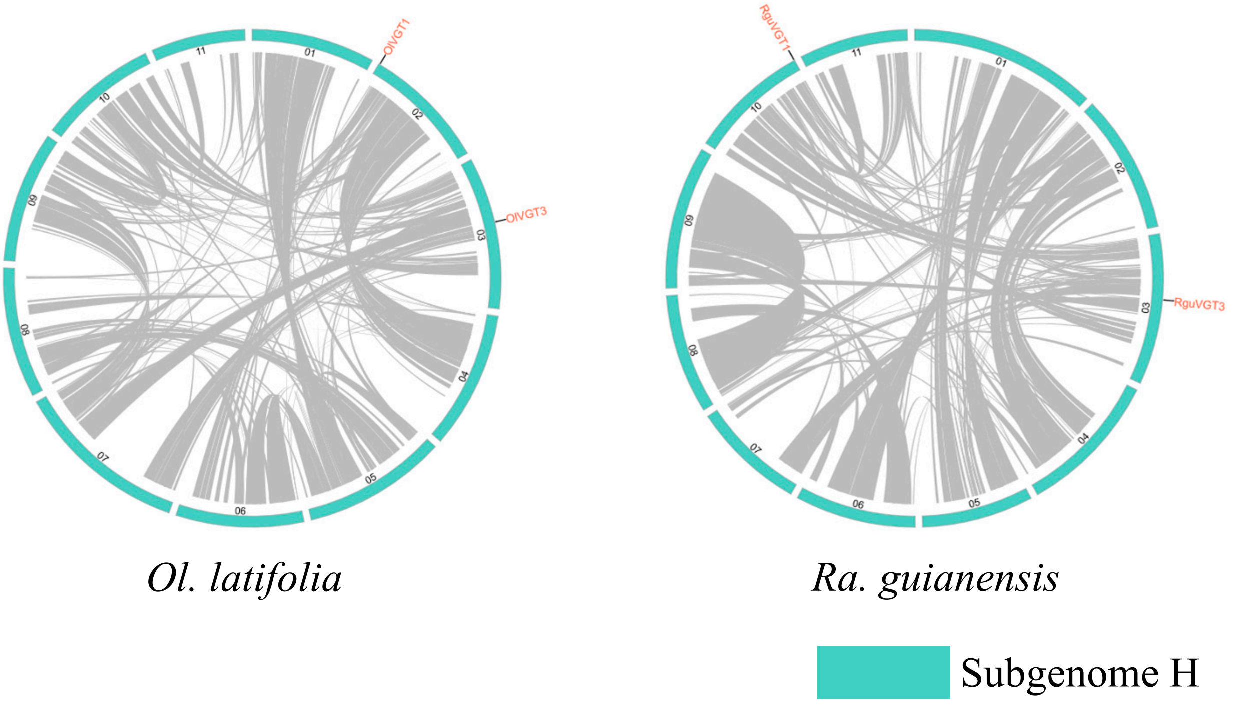

Supplementary Figure S71 Chromosomal location and synteny of the VGT gene family in diploid herbaceous bamboos

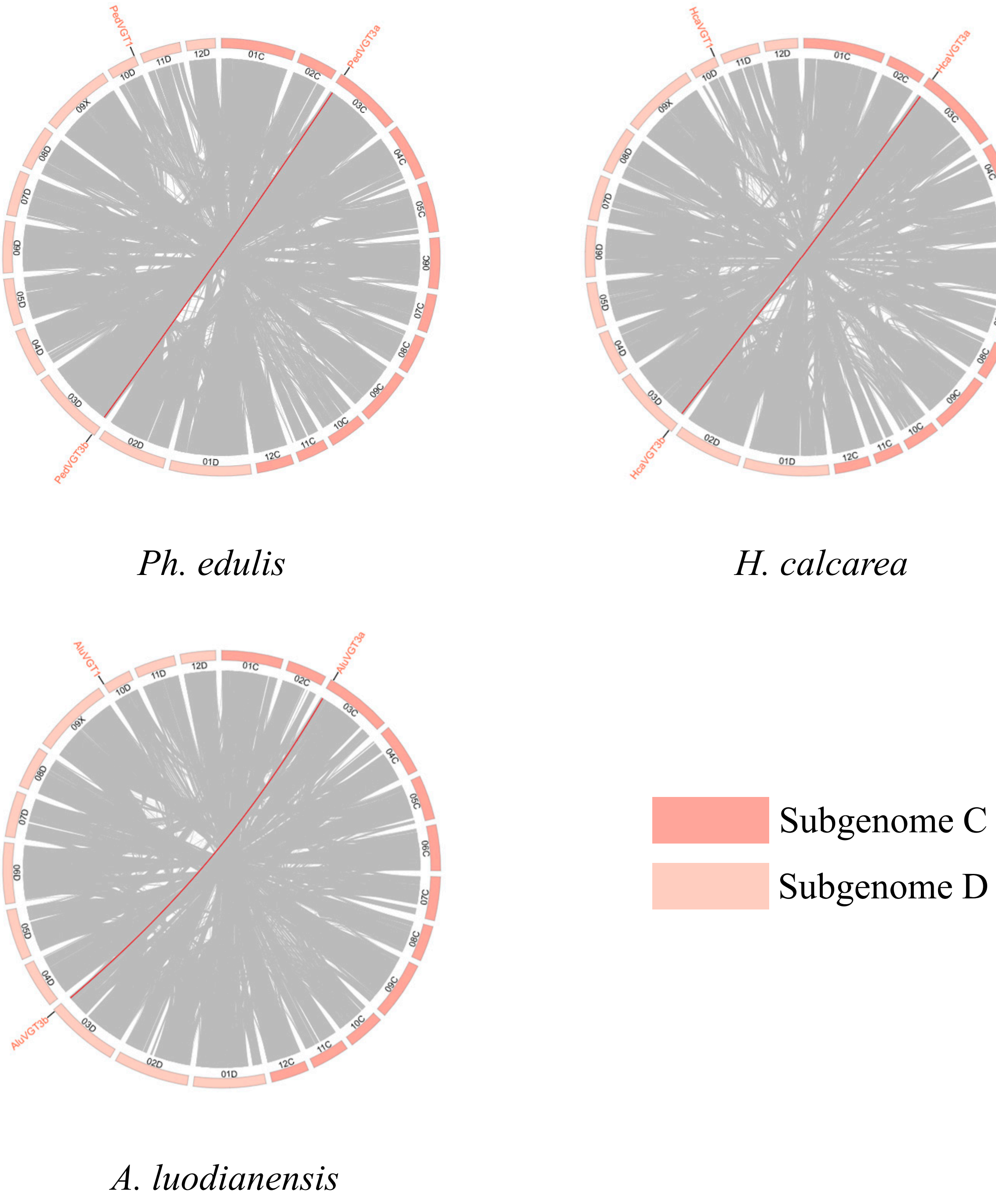

Supplementary Figure S72 Chromosomal location and synteny of the VGT gene family in tetraploid temperate woody bamboos

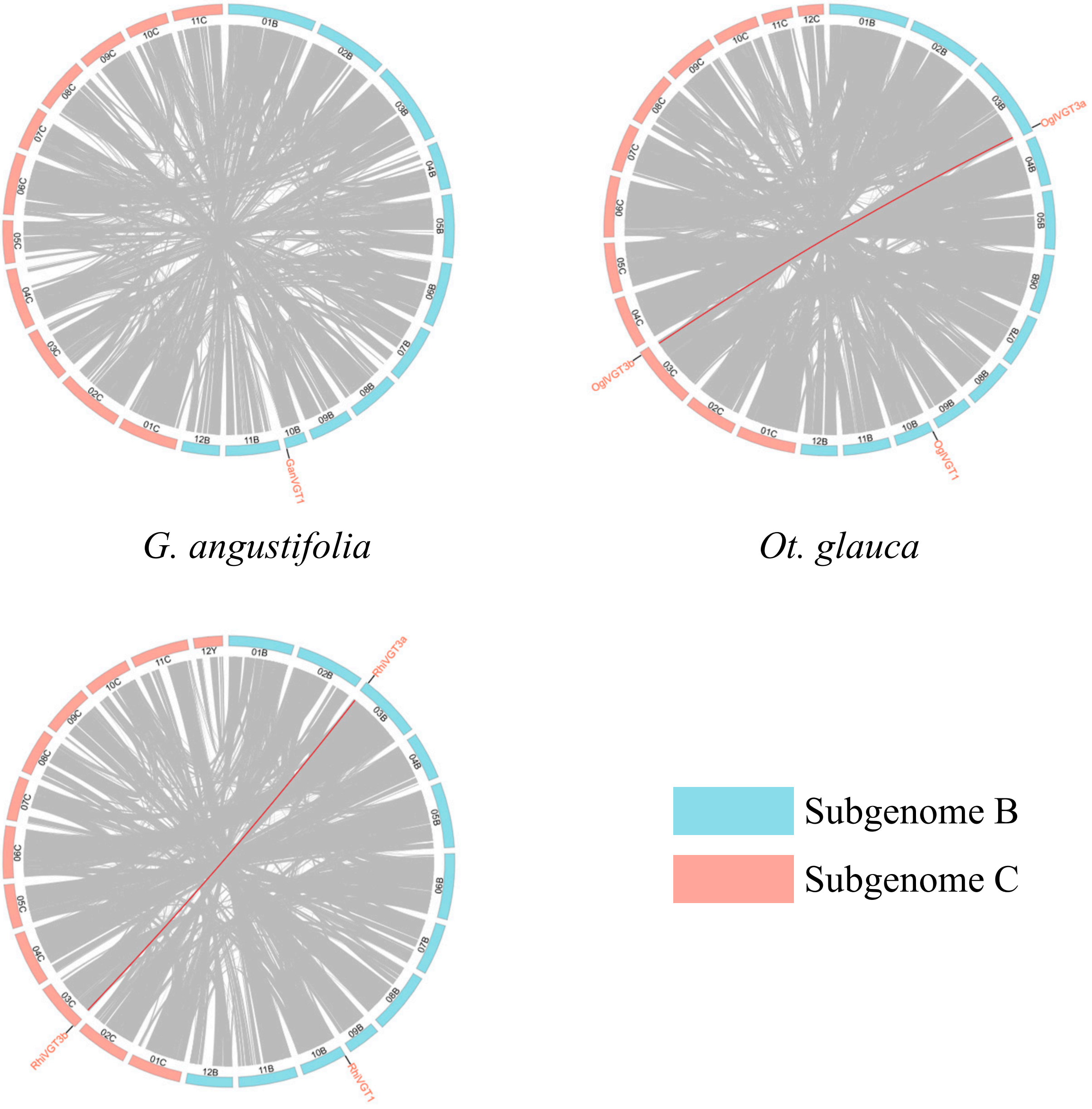

Rh. racemiflorum

Supplementary Figure S73 Chromosomal location and synteny of the VGT gene family in tetraploid neotropical woody bamboos

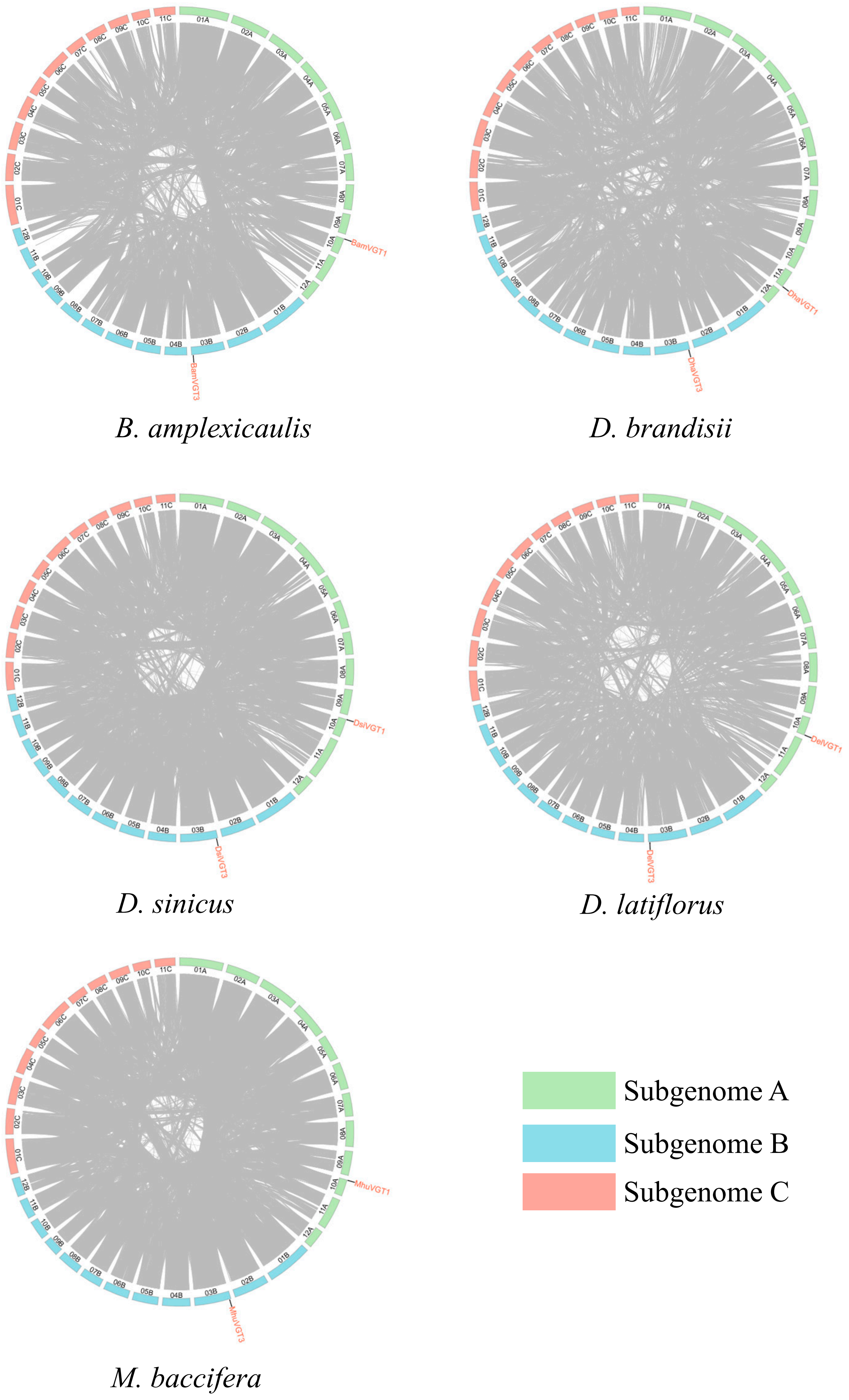

Supplementary Figure S74 Chromosomal location and synteny of the VGT gene family in hexaploid paleotropical woody bamboos

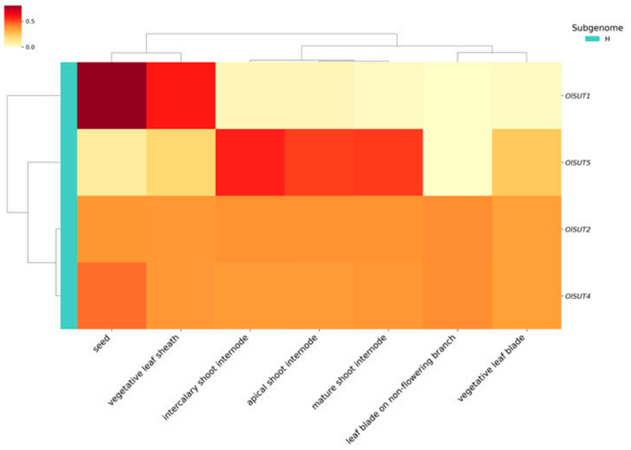

Ol. latifolia

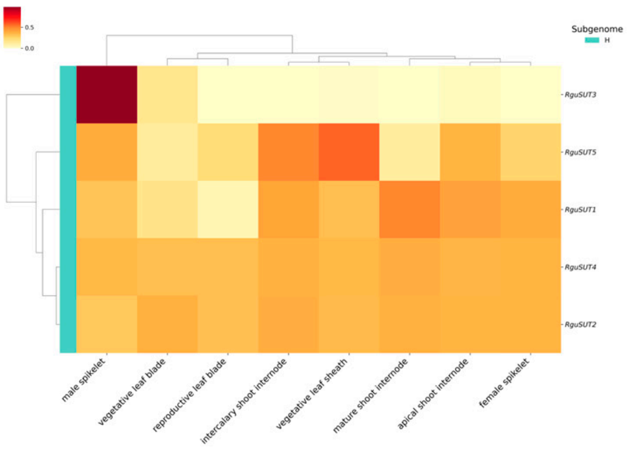

Ra. guianensis

Supplementary Figure S75 Diploid Herbaceous Bamboo SUT Transcriptome

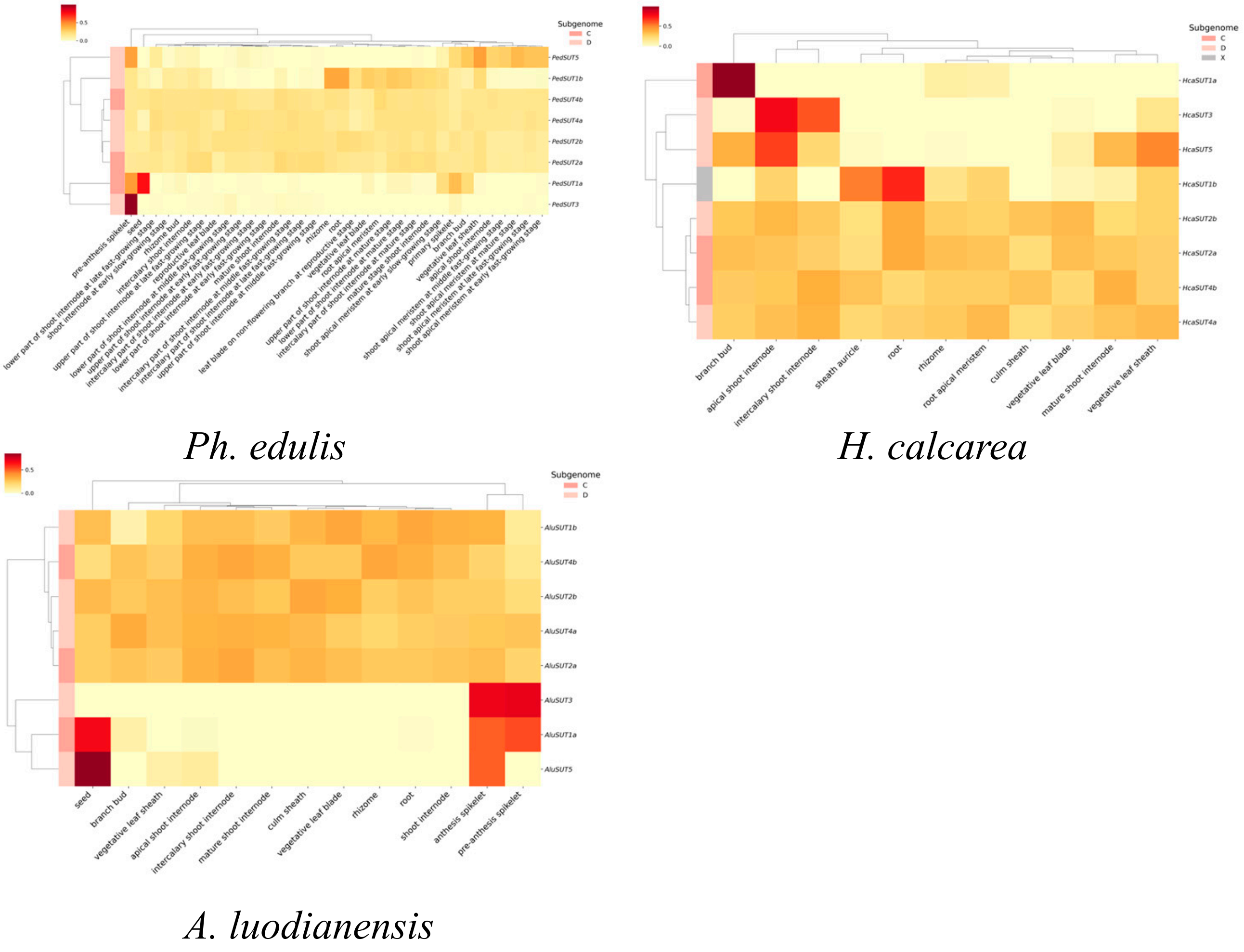

Supplementary Figure S76 Tetraploid temperate woody bamboo SUT Transcriptome

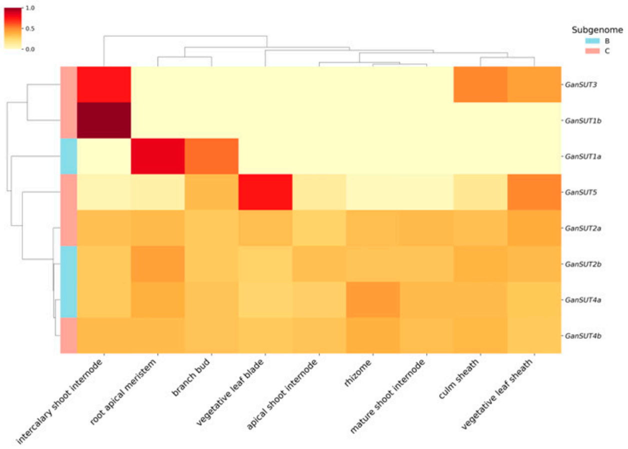

G. angustifolia

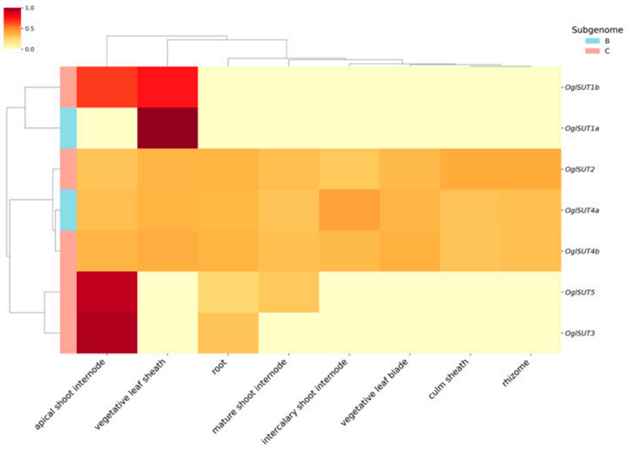

Ot. glauca

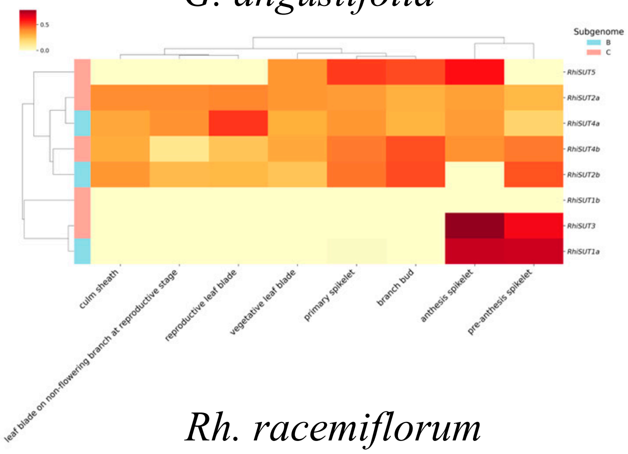

Rh. racemiflorum

Supplementary Figure S77 Tetraploid neotropical woody bamboos SUT Transcriptome

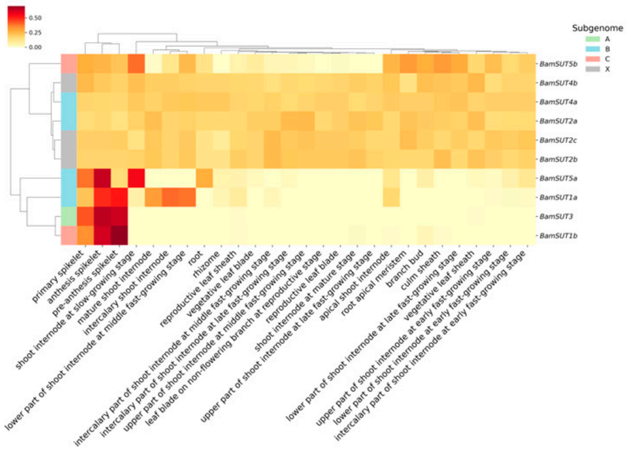

B. amplexicaulis

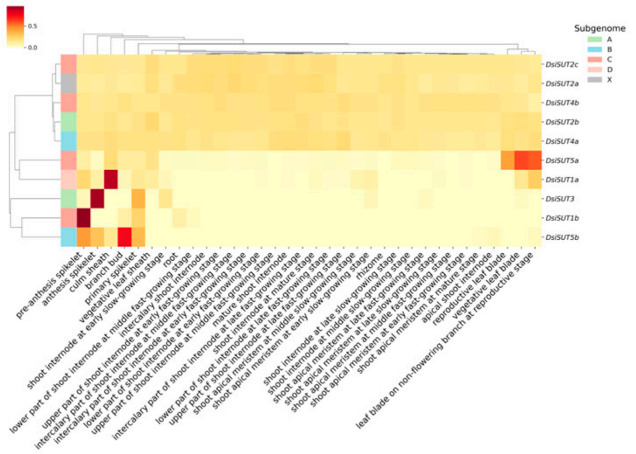

D. sinicus

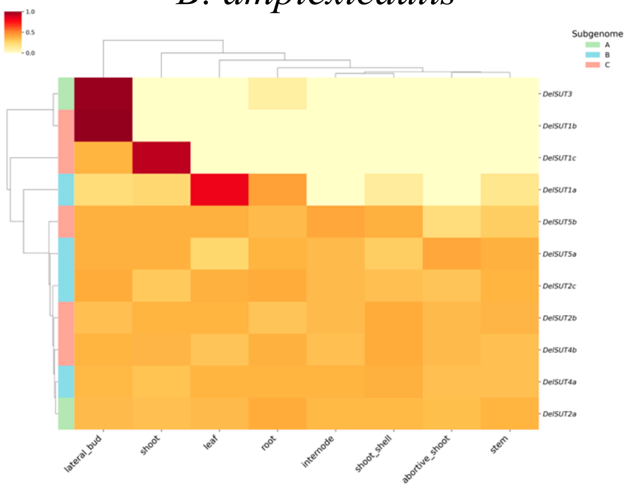

D. latiflorus

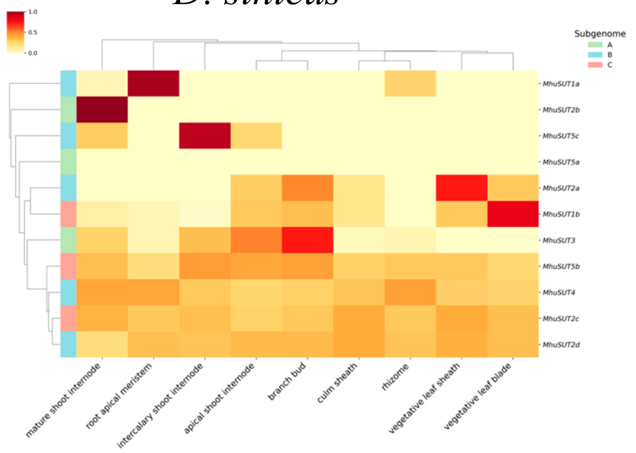

M. baccifera

Supplementary Figure S78 Hexaploid paleotropical woody bamboos SUT Transcriptome

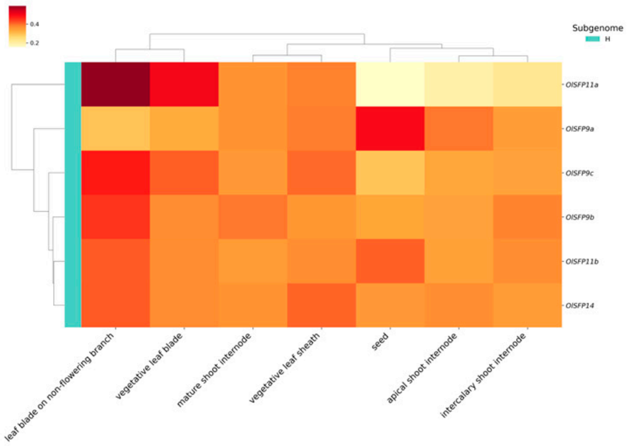

Ol. latifolia

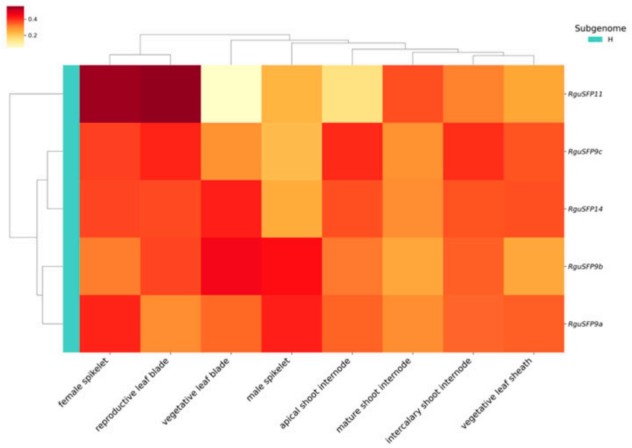

Ra. guianensis

Supplementary Figure S79 Diploid Herbaceous Bamboo ERDL6 Transcriptome

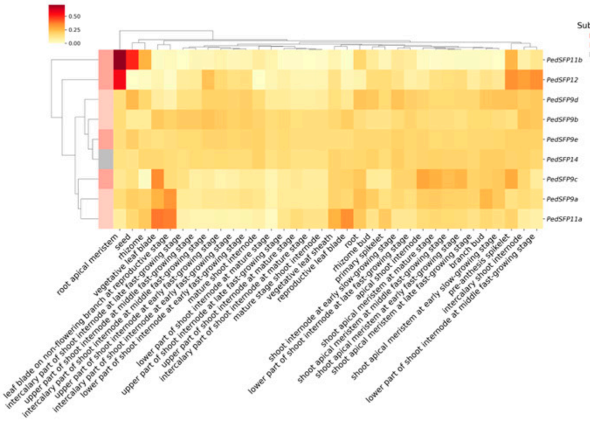

Ph. edulis

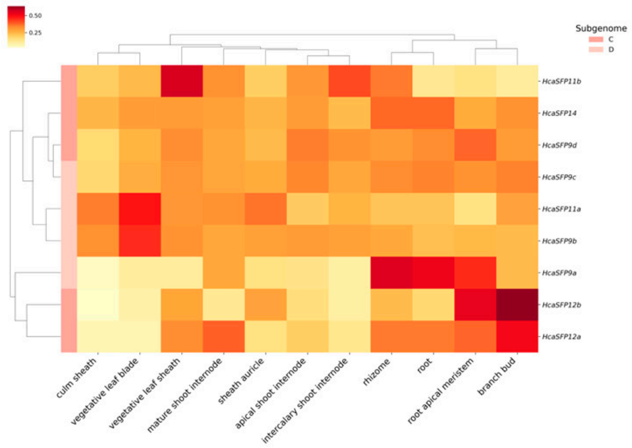

H. calcarea

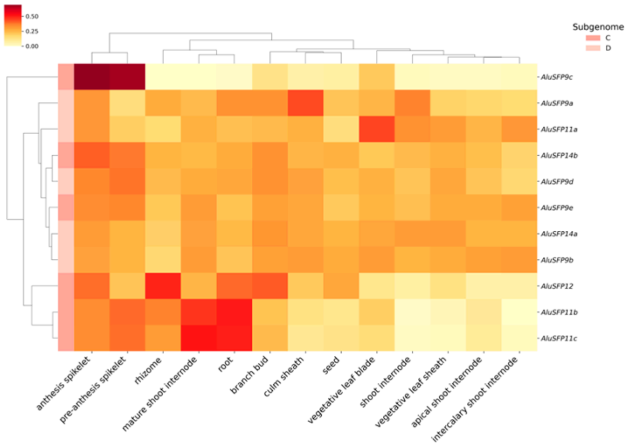

A. luodianensis

Supplementary Figure S80 Tetraploid temperate woody bamboo ERDL6 Transcriptome

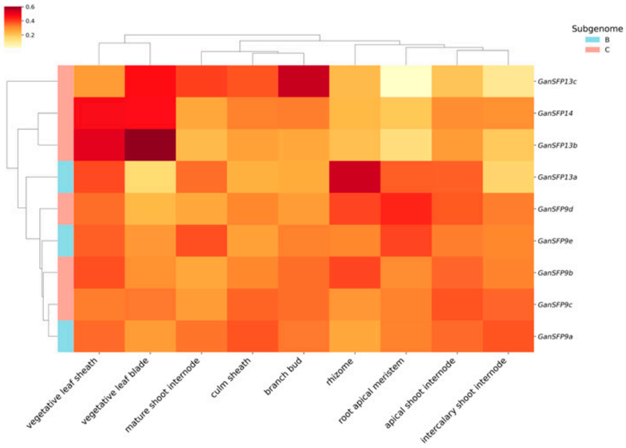

G. angustifolia

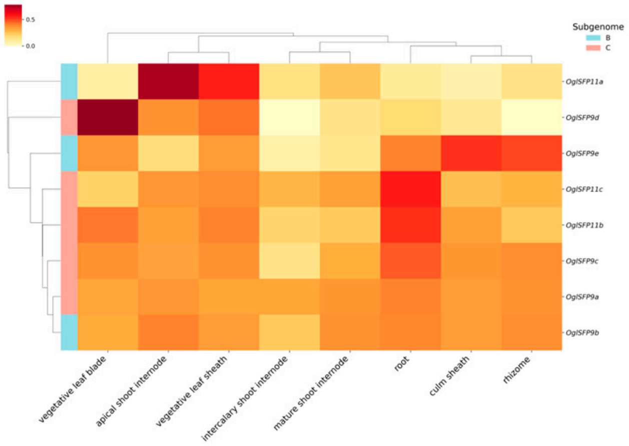

Ot. glauca

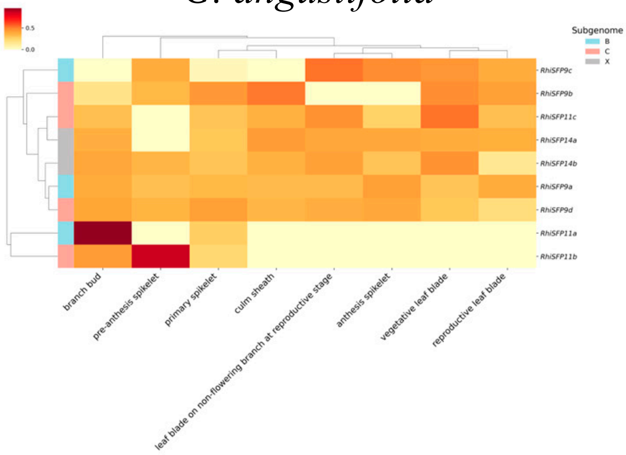

Rh. racemiflorum

Supplementary Figure S81 Tetraploid neotropical woody bamboos ERDL6 Transcriptome

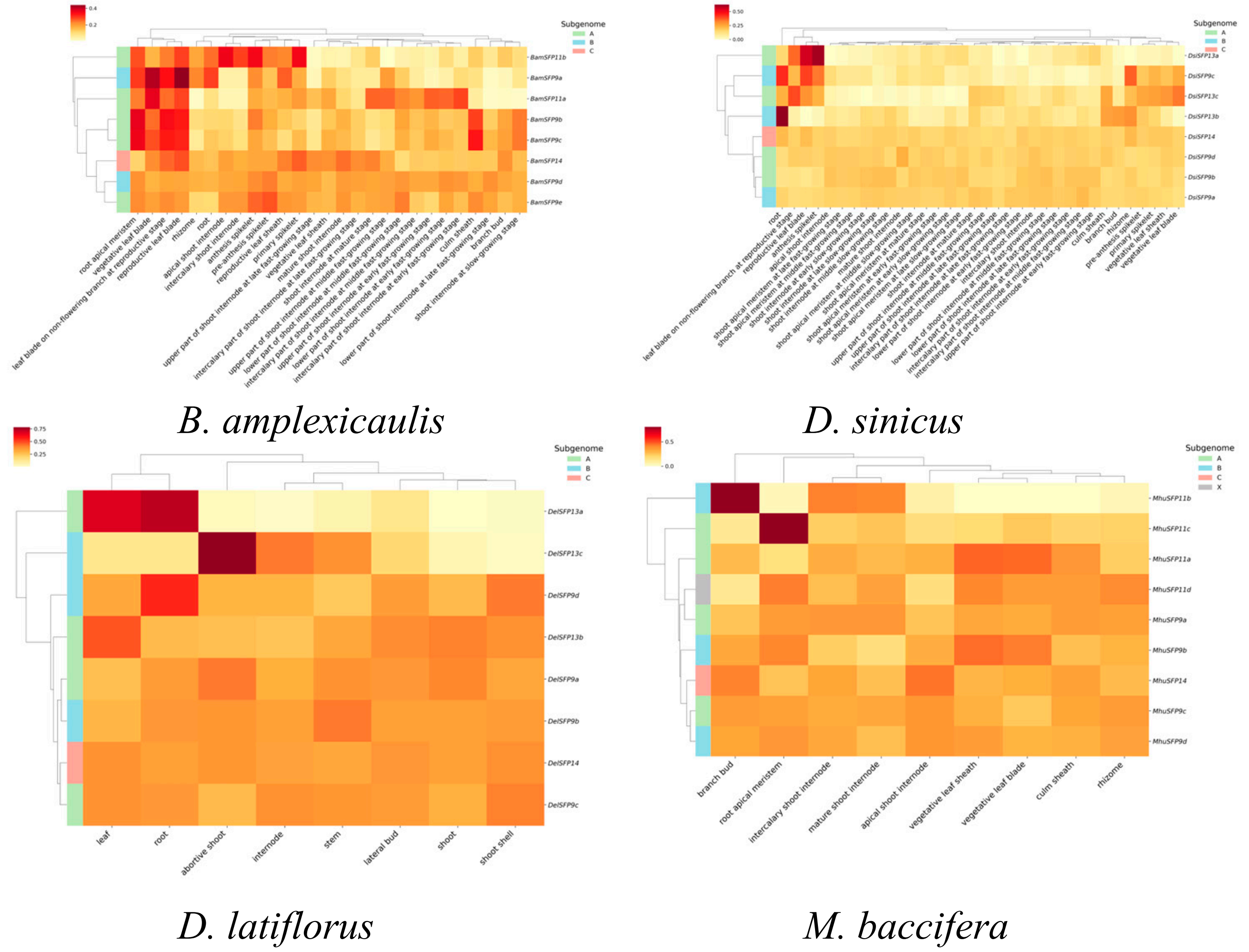

Supplementary Figure S82 Hexaploid paleotropical woody bamboos ERDL6 Transcriptome

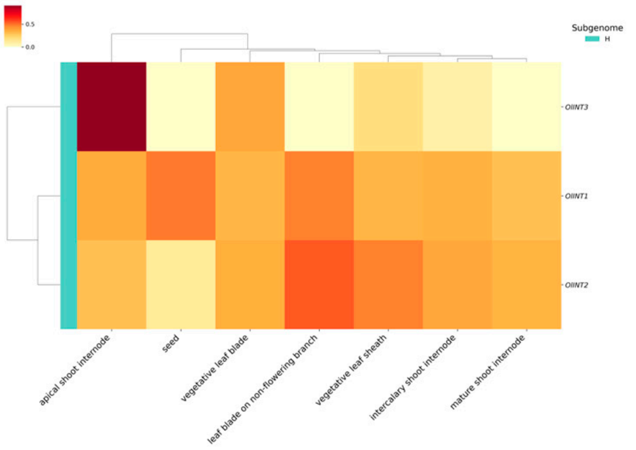

Ol. latifolia

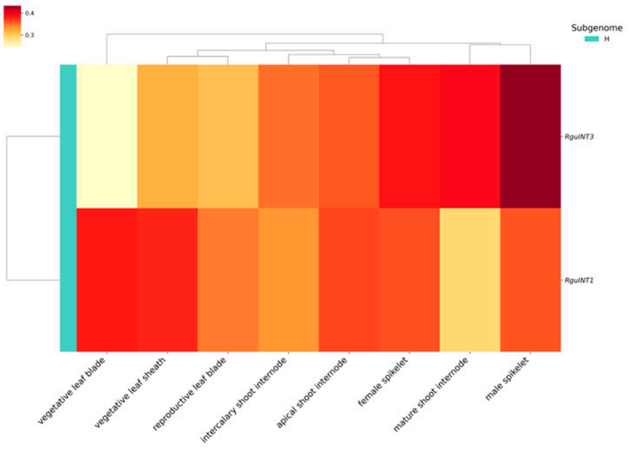

Ra. guianensis

Supplementary Figure S83 Diploid Herbaceous Bamboo INT Transcriptome

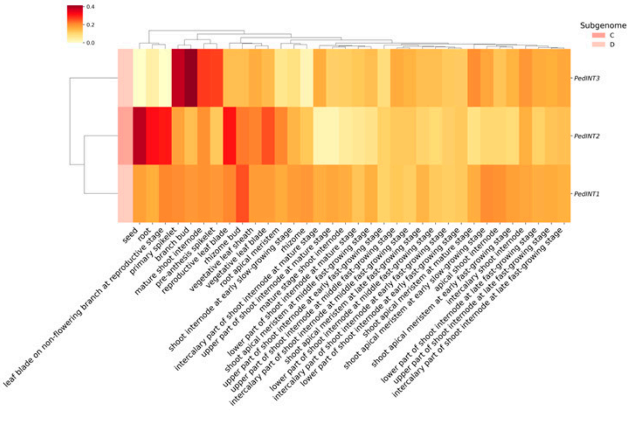

Ph. edulis

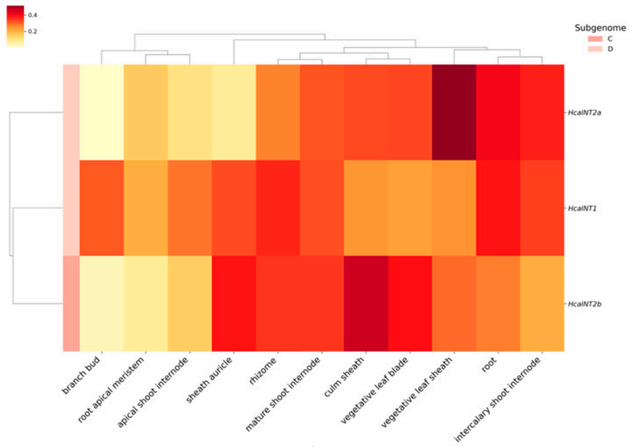

H. calcarea

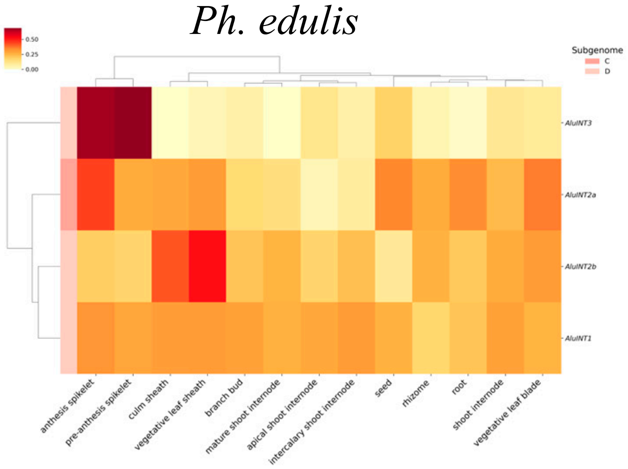

A. luodianensis

Supplementary Figure S84 Tetraploid temperate woody bamboo INT Transcriptome

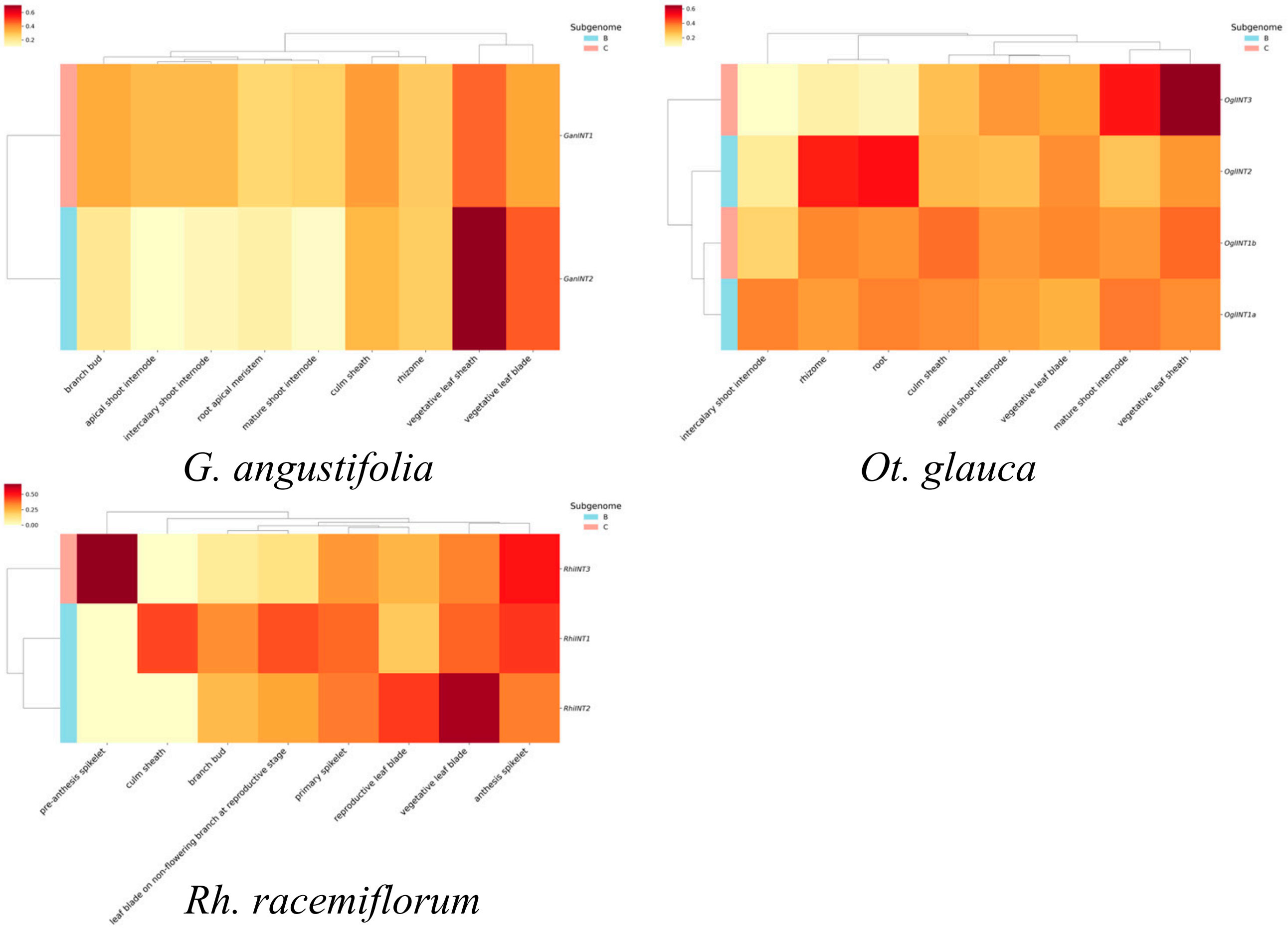

Supplementary Figure S85 Tetraploid neotropical woody bamboos INT Transcriptome

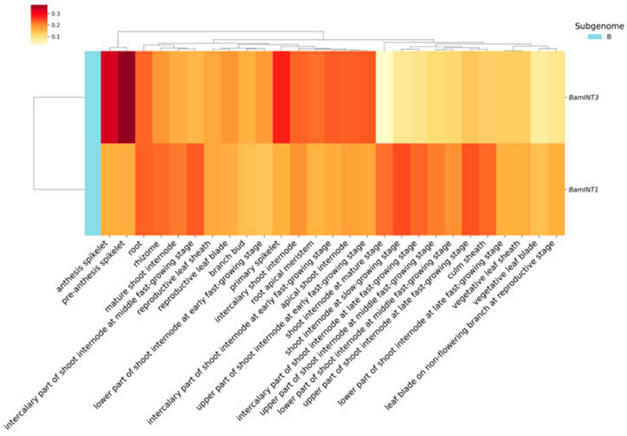

B. amplexicaulis

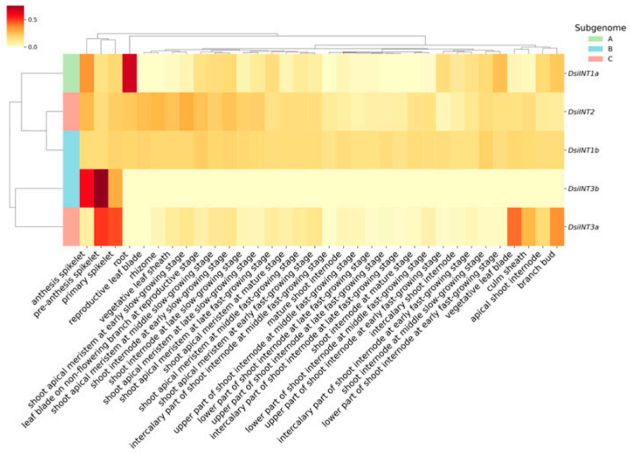

D. sinicus

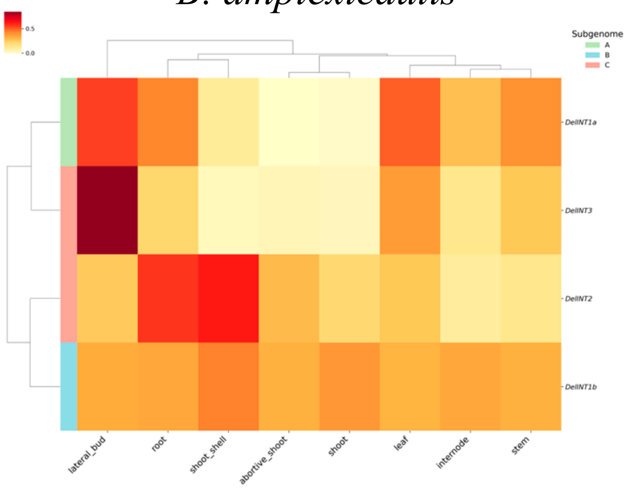

D. latiflorus

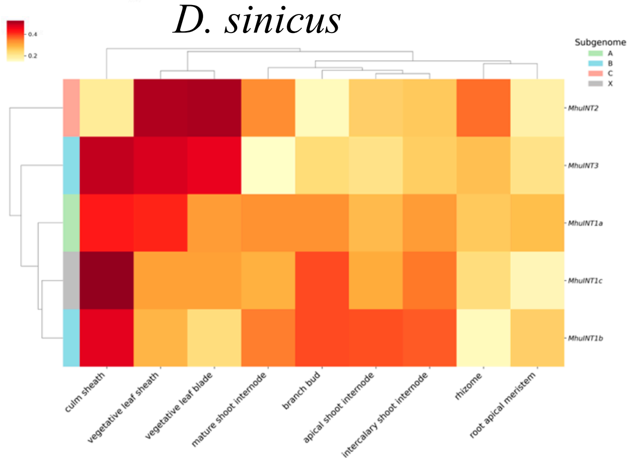

M. baccifera

Supplementary Figure S86 Hexaploid paleotropical woody bamboos INT Transcriptome

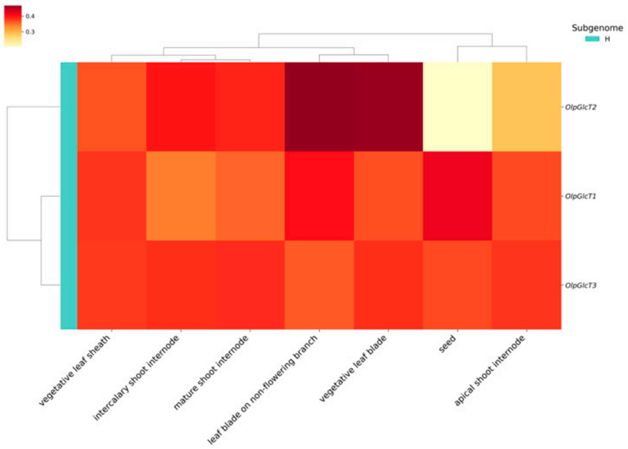

Ol. latifolia

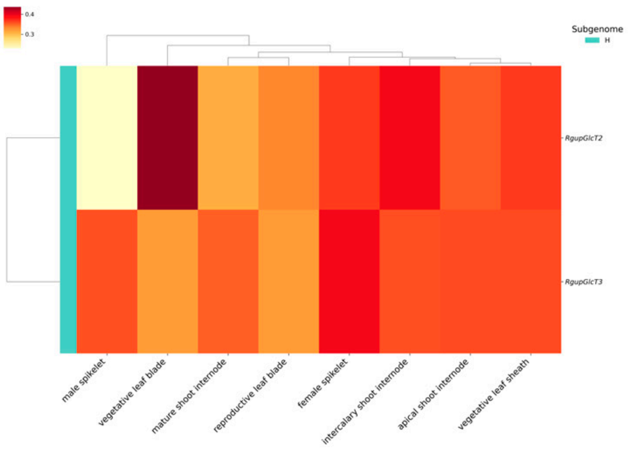

Ra. guianensis

Supplementary Figure S87 Diploid Herbaceous Bamboo pGlcT Transcriptome

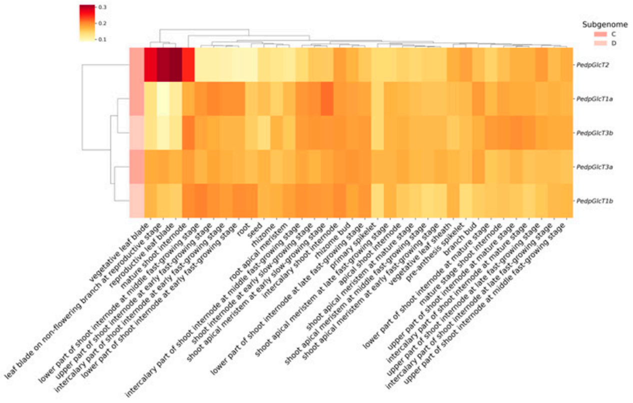

Ph. edulis

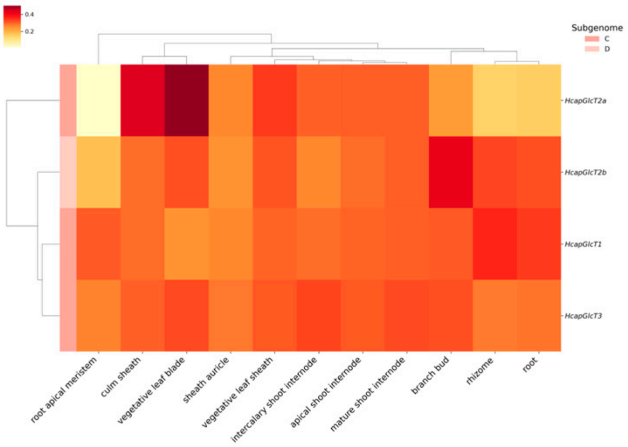

H. calcarea

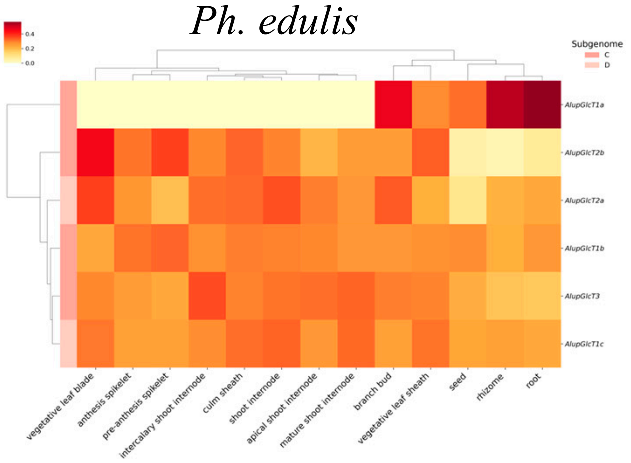

A. luodianensis

Supplementary Figure S88 Tetraploid temperate woody bamboo pGlcT Transcriptome

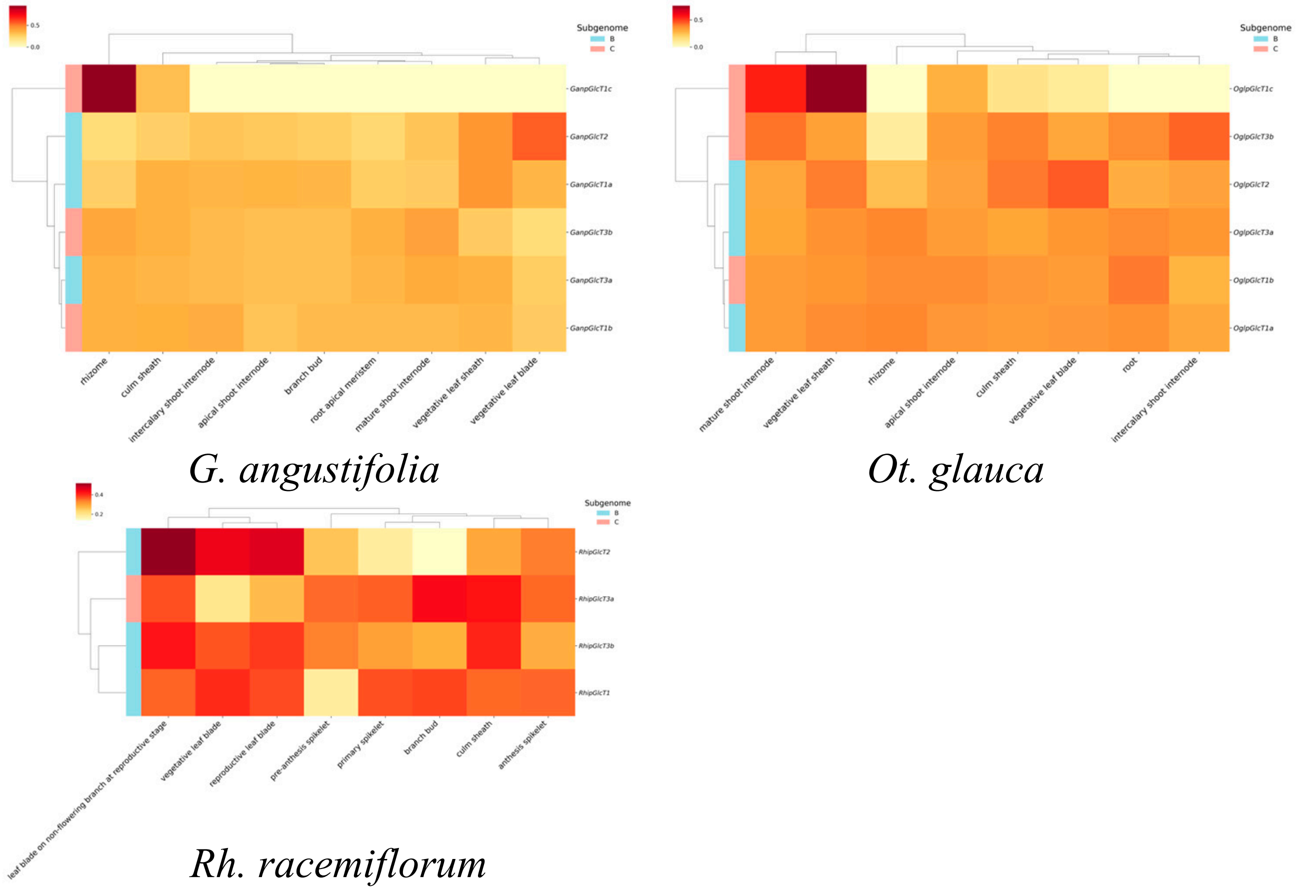

Supplementary Figure S89 Tetraploid neotropical woody bamboos pGlcT Transcriptome

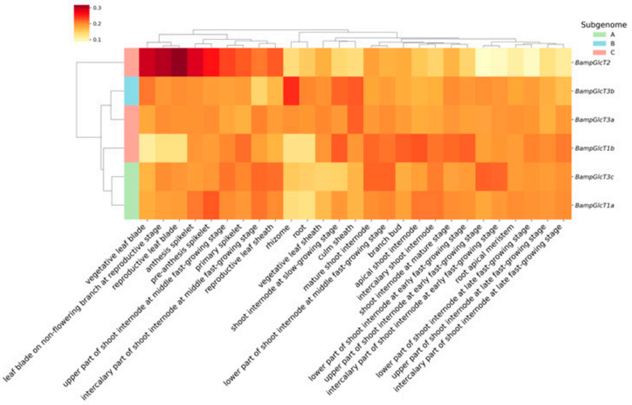

B. amplexicaulis

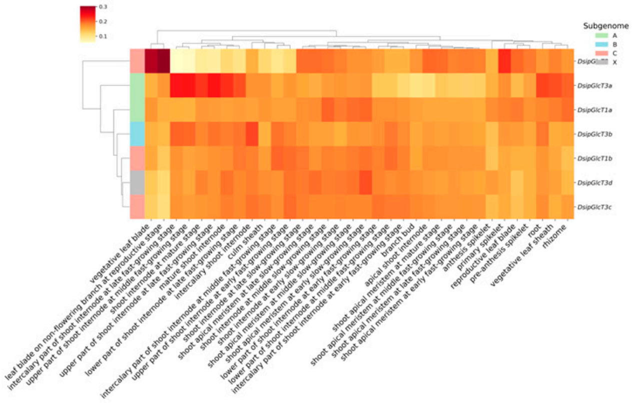

D. sinicus

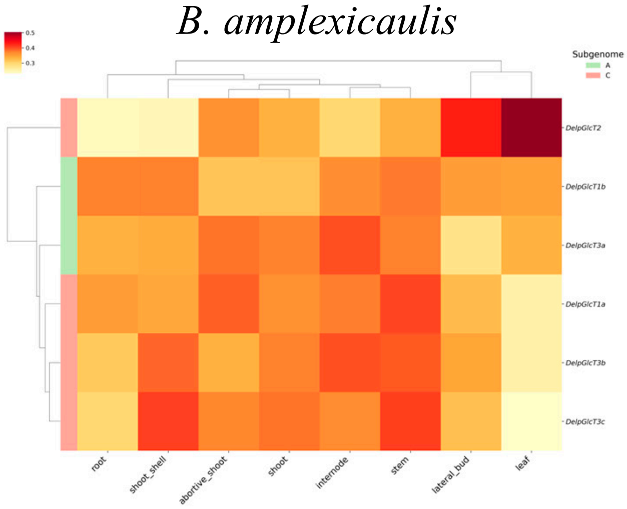

D. latiflorus

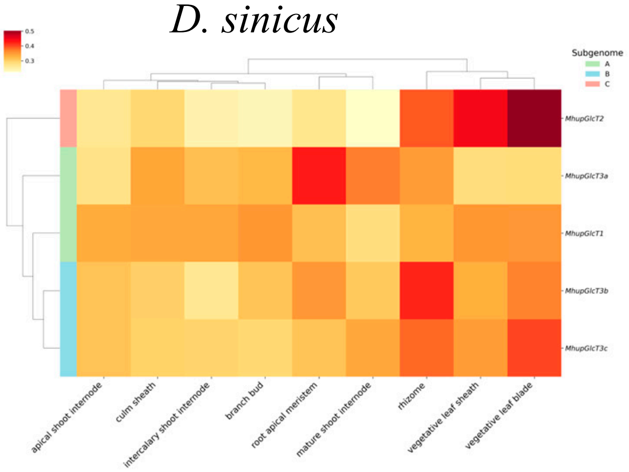

M. baccifera

Supplementary Figure S90 Hexaploid paleotropical woody bamboos pGlcT Transcriptome

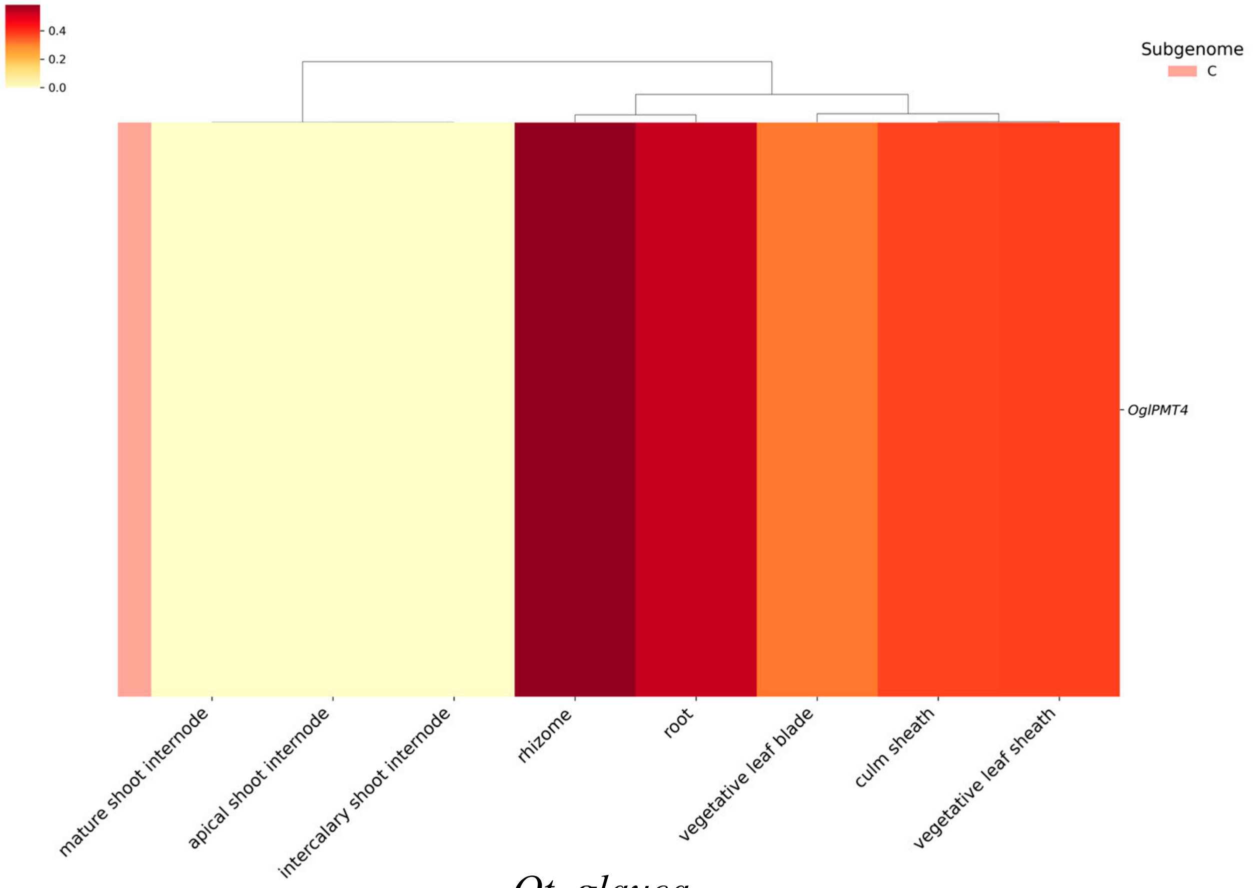

Ot. glauca

Supplementary Figure S91 Tetraploid neotropical woody bamboos PMT Transcriptome

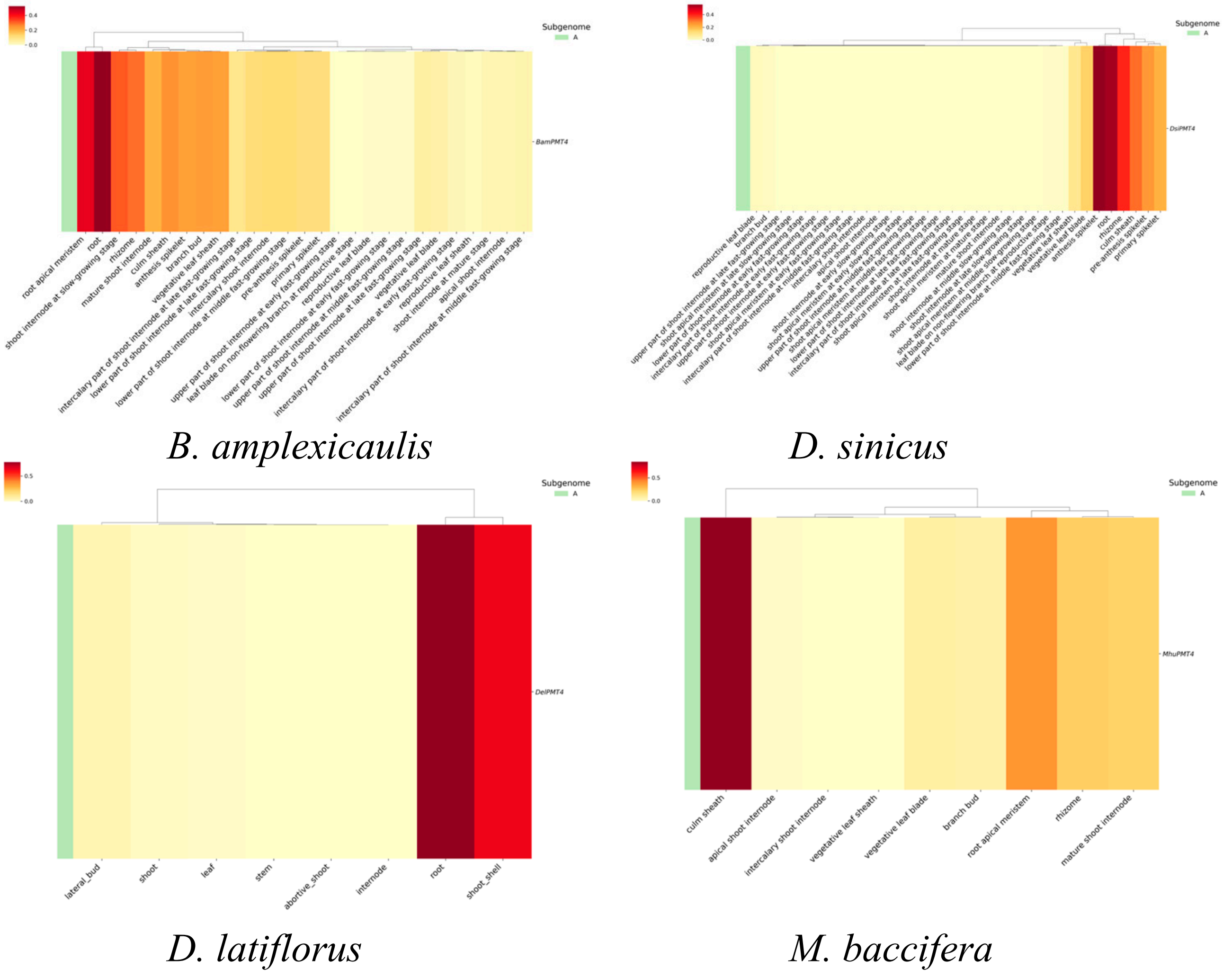

Supplementary Figure S92 Hexaploid paleotropical woody bamboos PMT Transcriptome

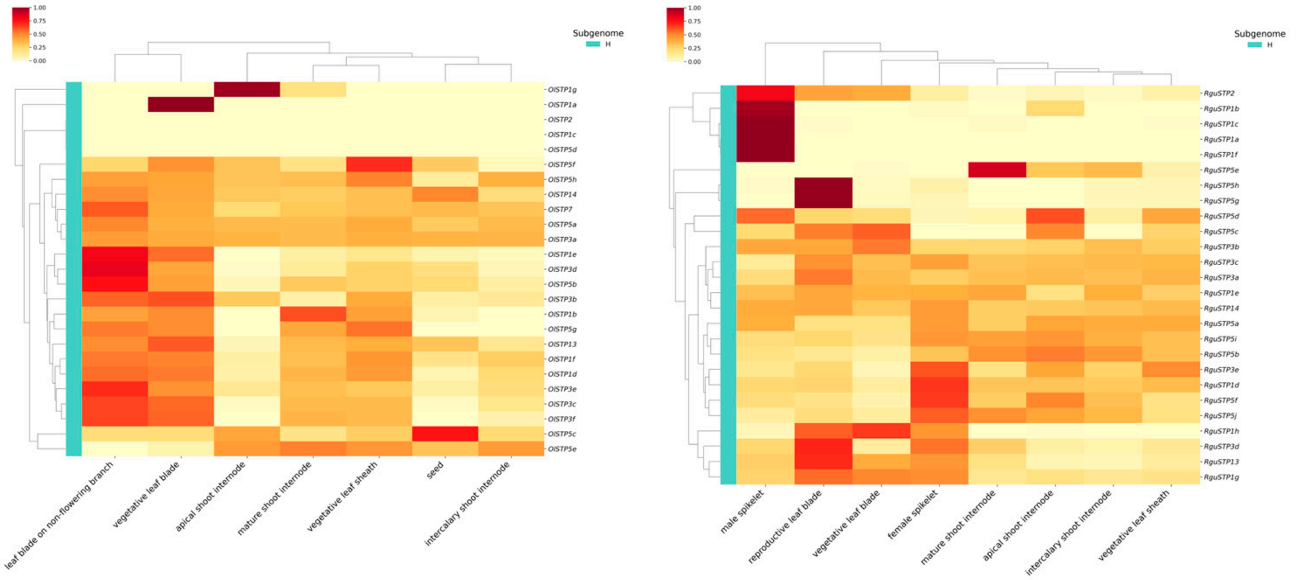

Ol. latifolia *Ra. guianensis*

Supplementary Figure S93 Diploid Herbaceous Bamboo STP Transcriptome

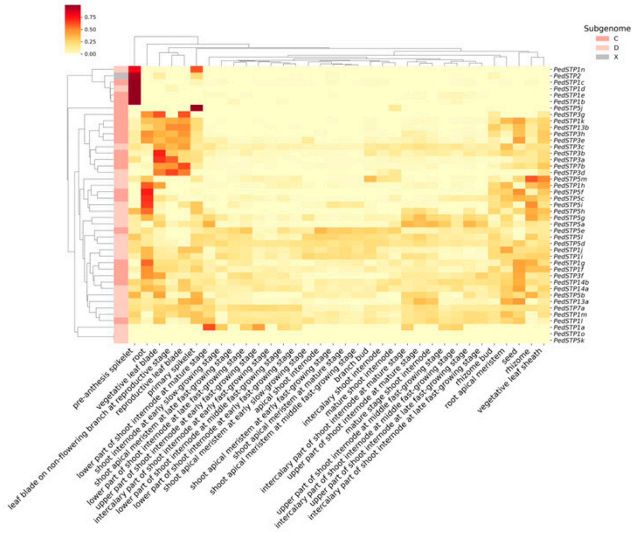

Ph. edulis

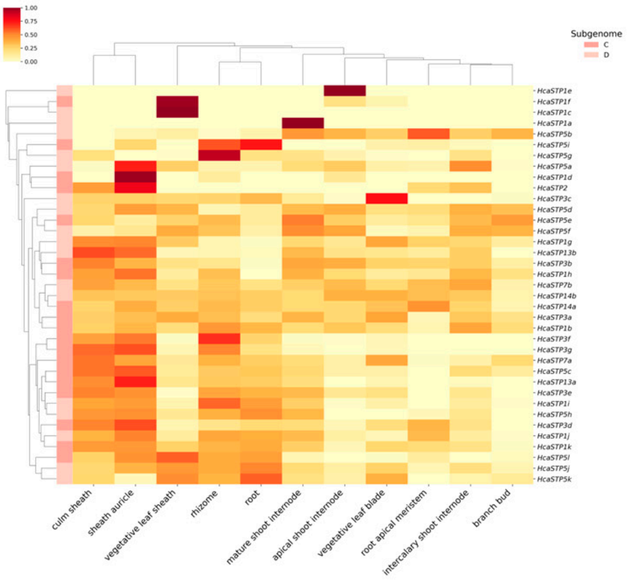

H. calcarea

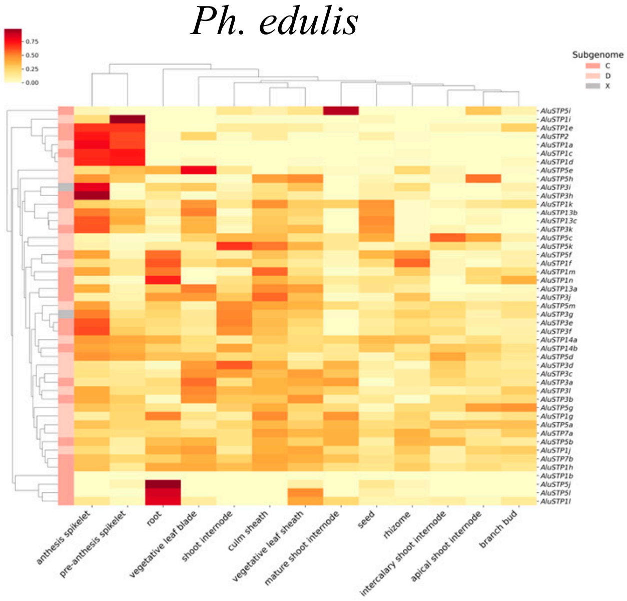

A. luodianensis

Supplementary Figure S94 Tetraploid temperate woody bamboo STP Transcriptome

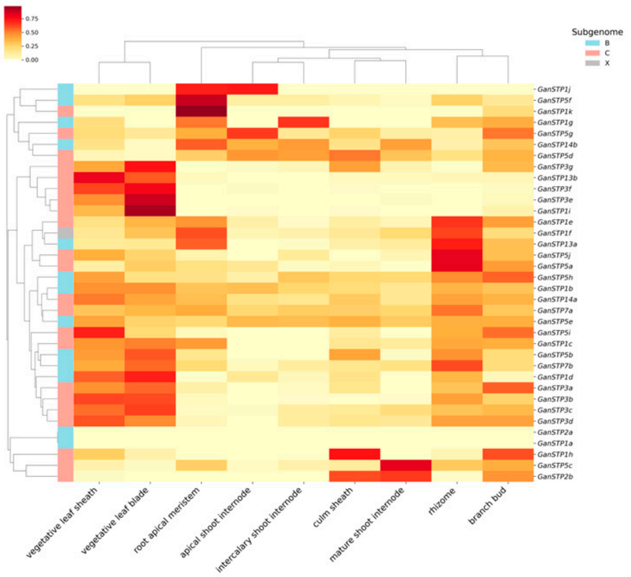

G. angustifolia

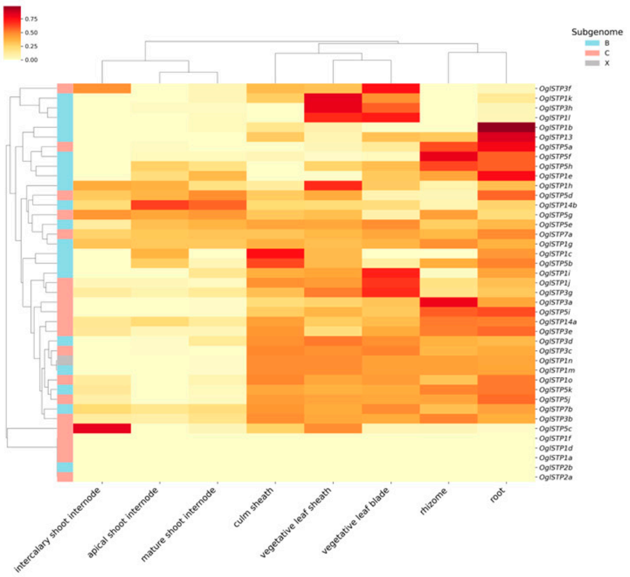

Ot. glauca

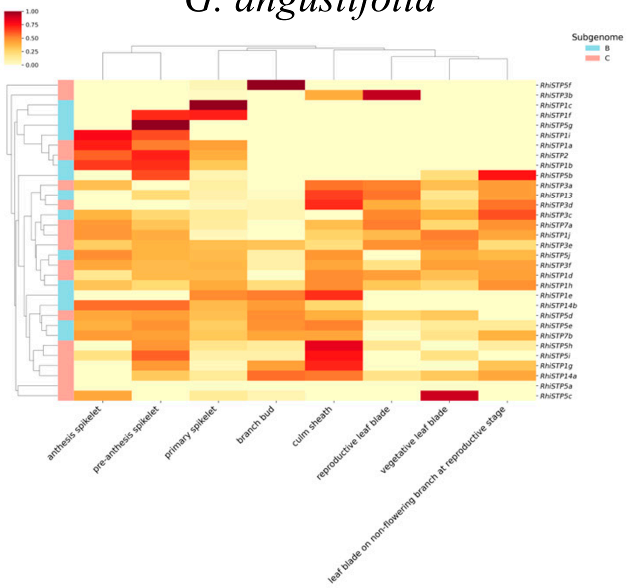

Rh. racemiflorum

Supplementary Figure S95 Tetraploid neotropical woody bamboos STP Transcriptome

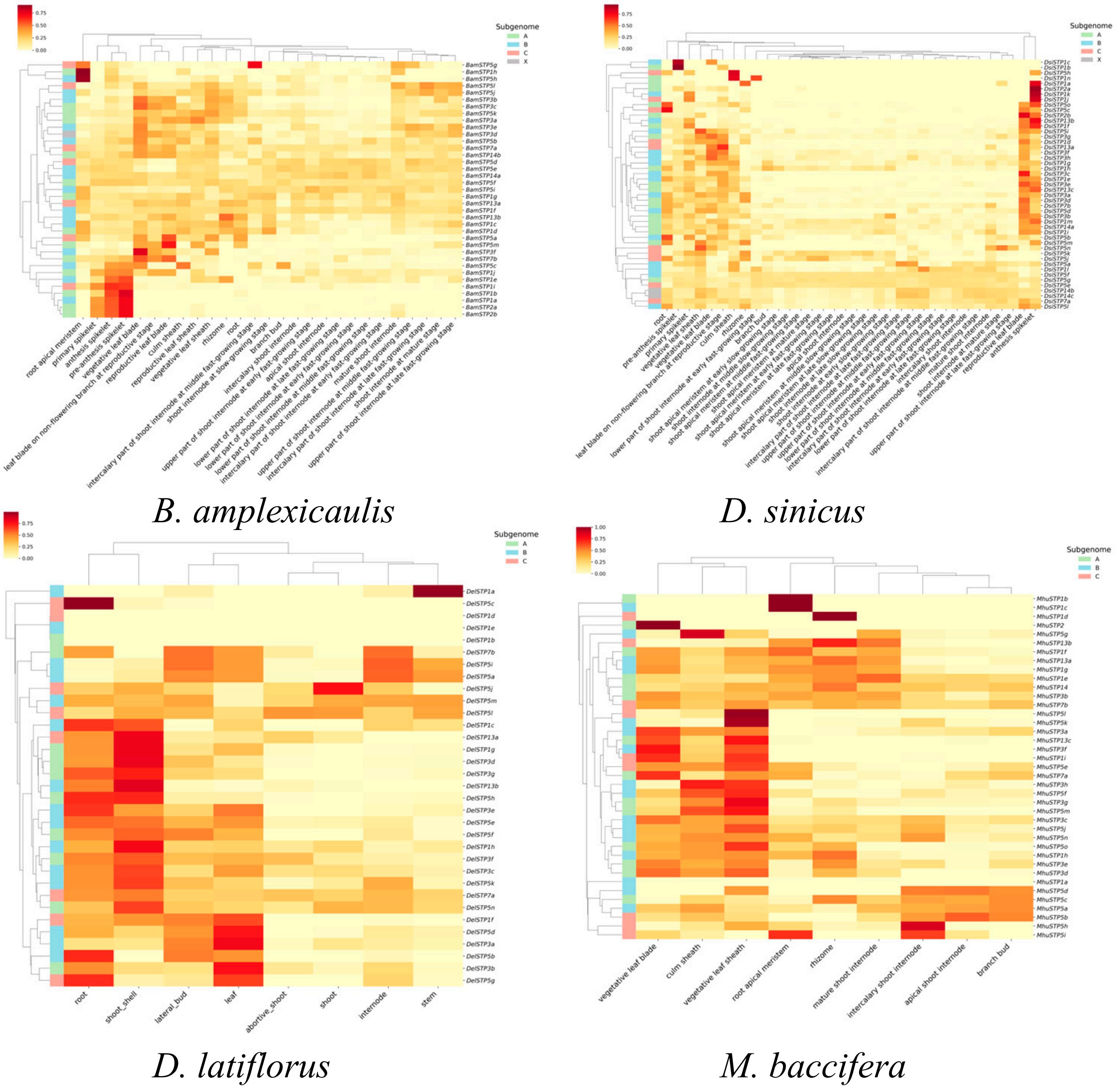

Supplementary Figure S96 Hexaploid paleotropical woody bamboos STP Transcriptome

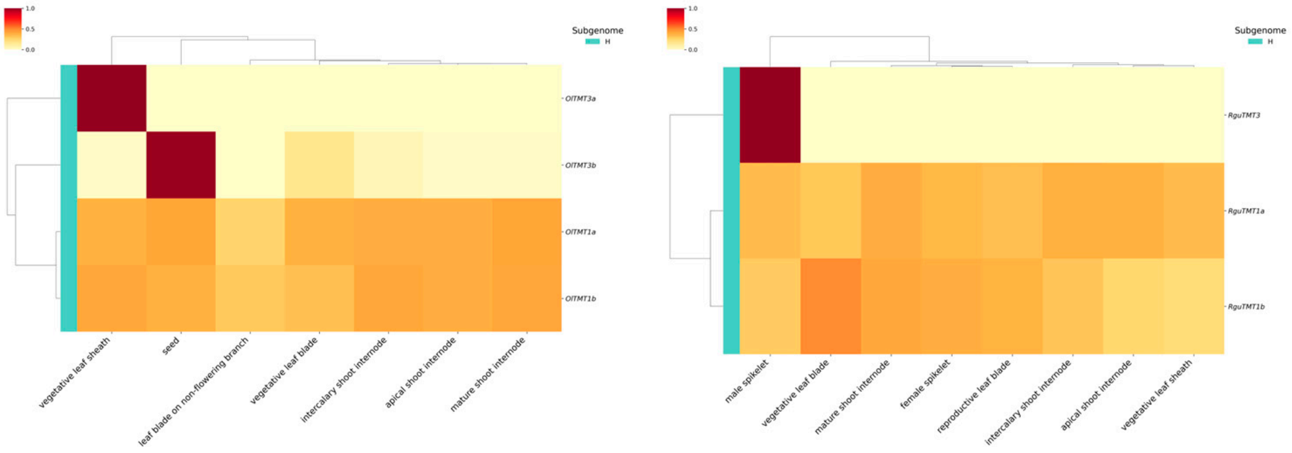

Ol. latifolia *Ra. guianensis*

Supplementary Figure S97 Diploid Herbaceous Bamboo TMT Transcriptome

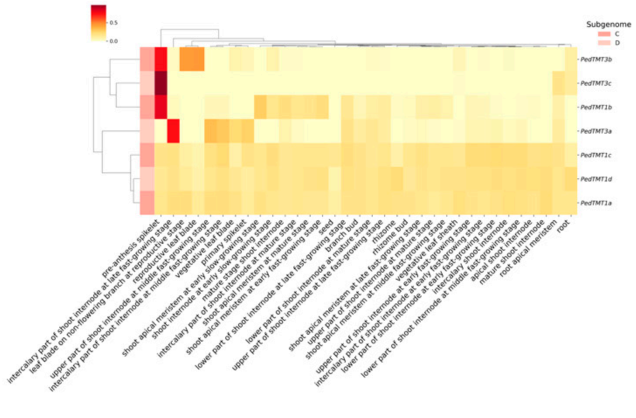

Ph. edulis

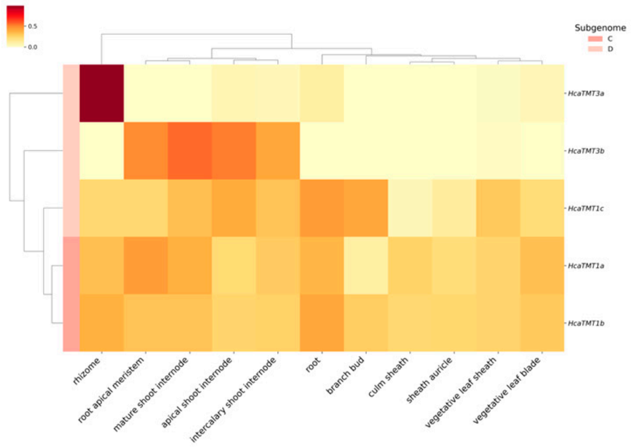

H. calcarea

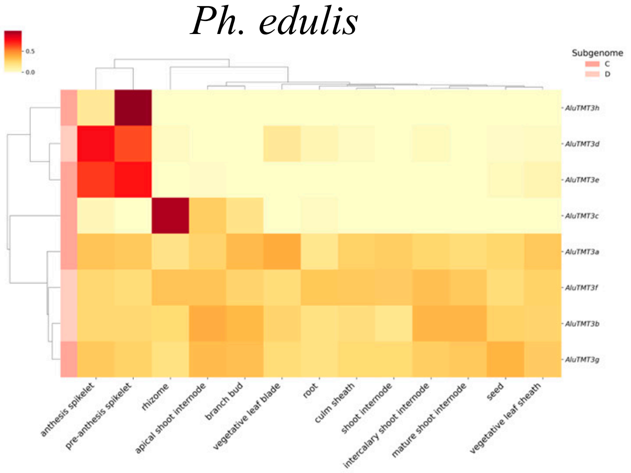

A. luodianensis

Supplementary Figure S98 Tetraploid temperate woody bamboo TMT Transcriptome

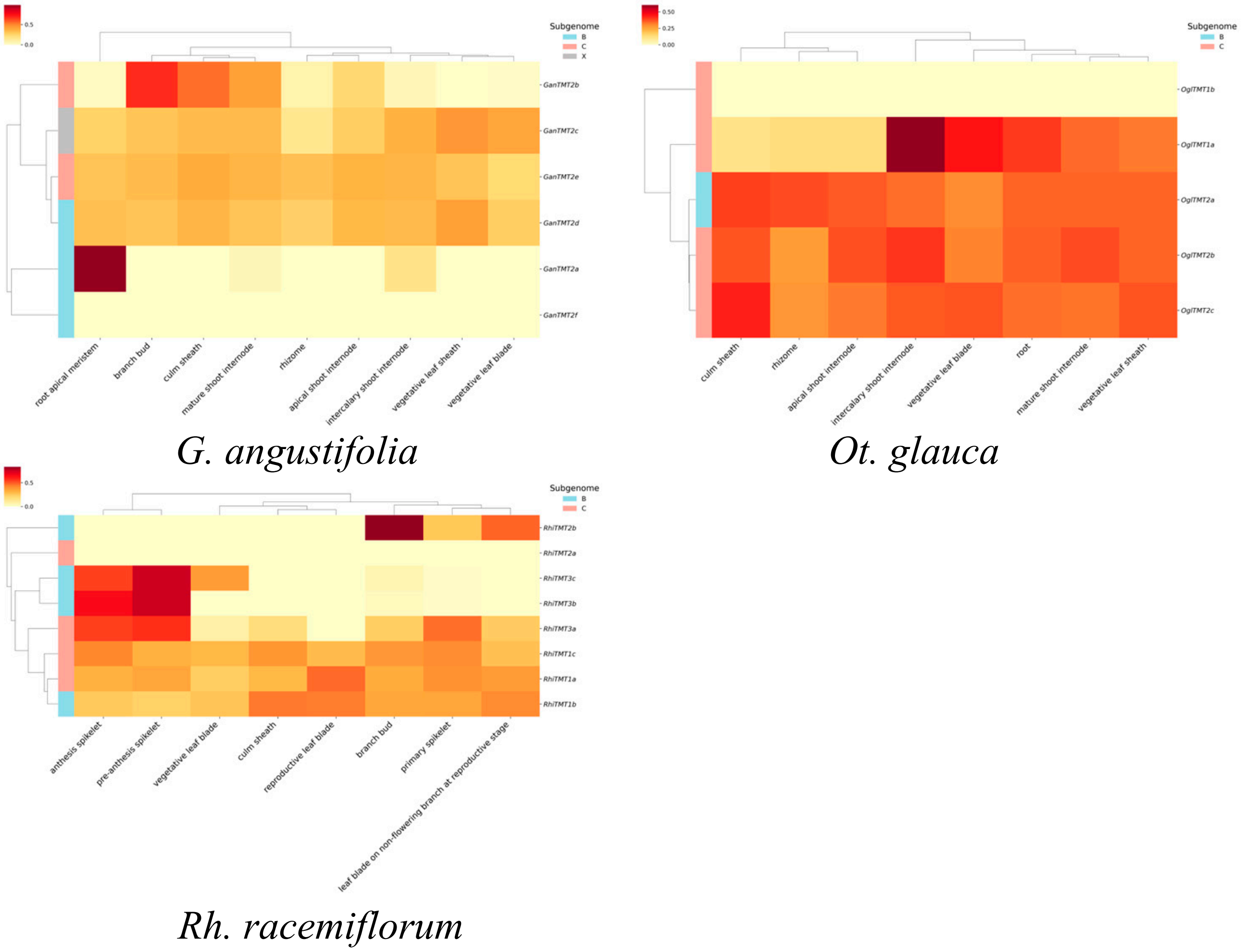

Supplementary Figure S99 Tetraploid neotropical woody bamboos TMT Transcriptome

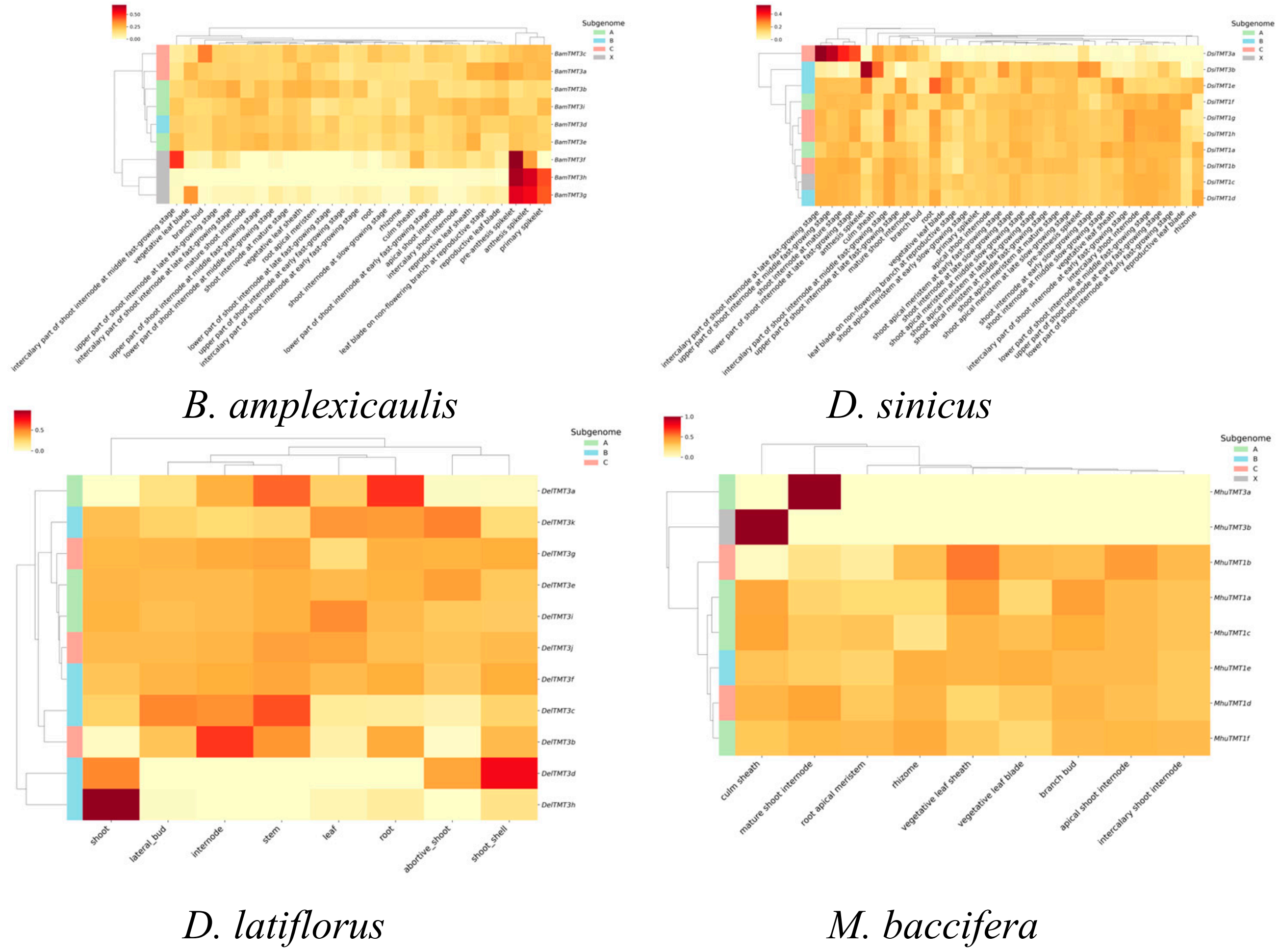

Supplementary Figure S100 Hexaploid paleotropical woody bamboos TMT Transcriptome

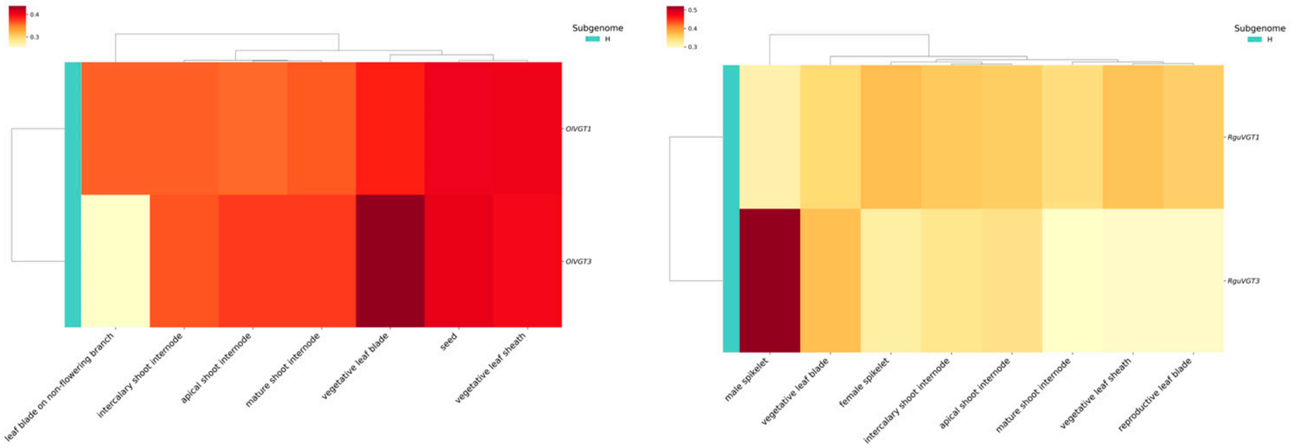

Ol. latifolia *Ra. guianensis*

Supplementary Figure S101 Diploid Herbaceous Bamboo VGT Transcriptome

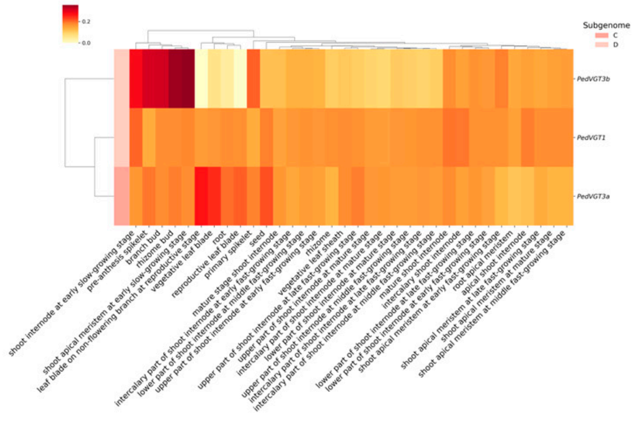

Ph. edulis

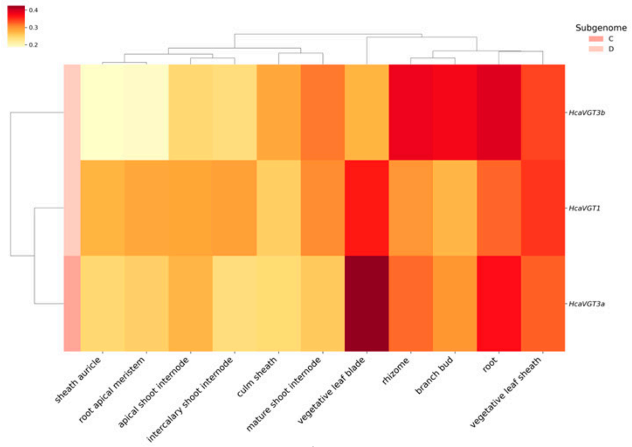

H. calcarea

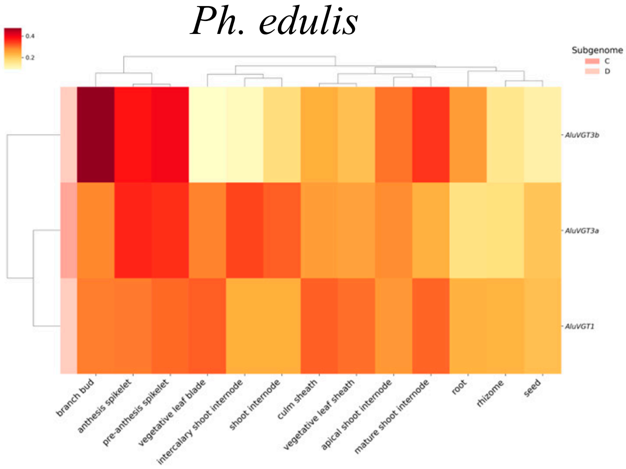

A. luodianensis

Supplementary Figure S102 Tetraploid temperate woody bamboo VGT Transcriptome

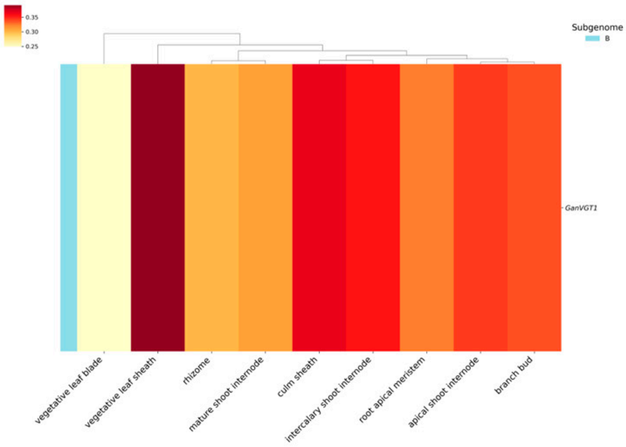

G. angustifolia

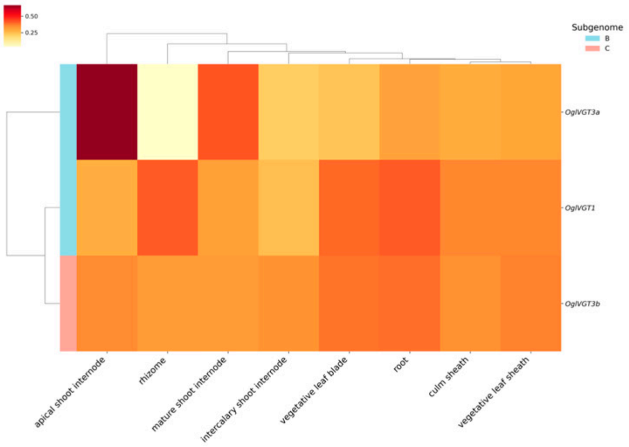

Ot. glauca

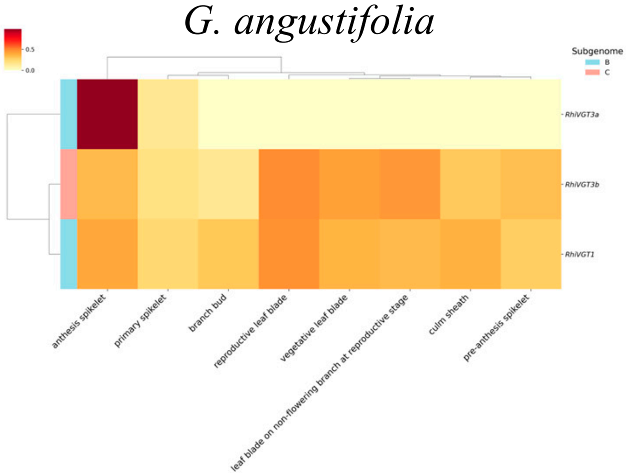

Rh. racemiflorum

Supplementary Figure S103 Tetraploid neotropical woody bamboos VGT Transcriptome

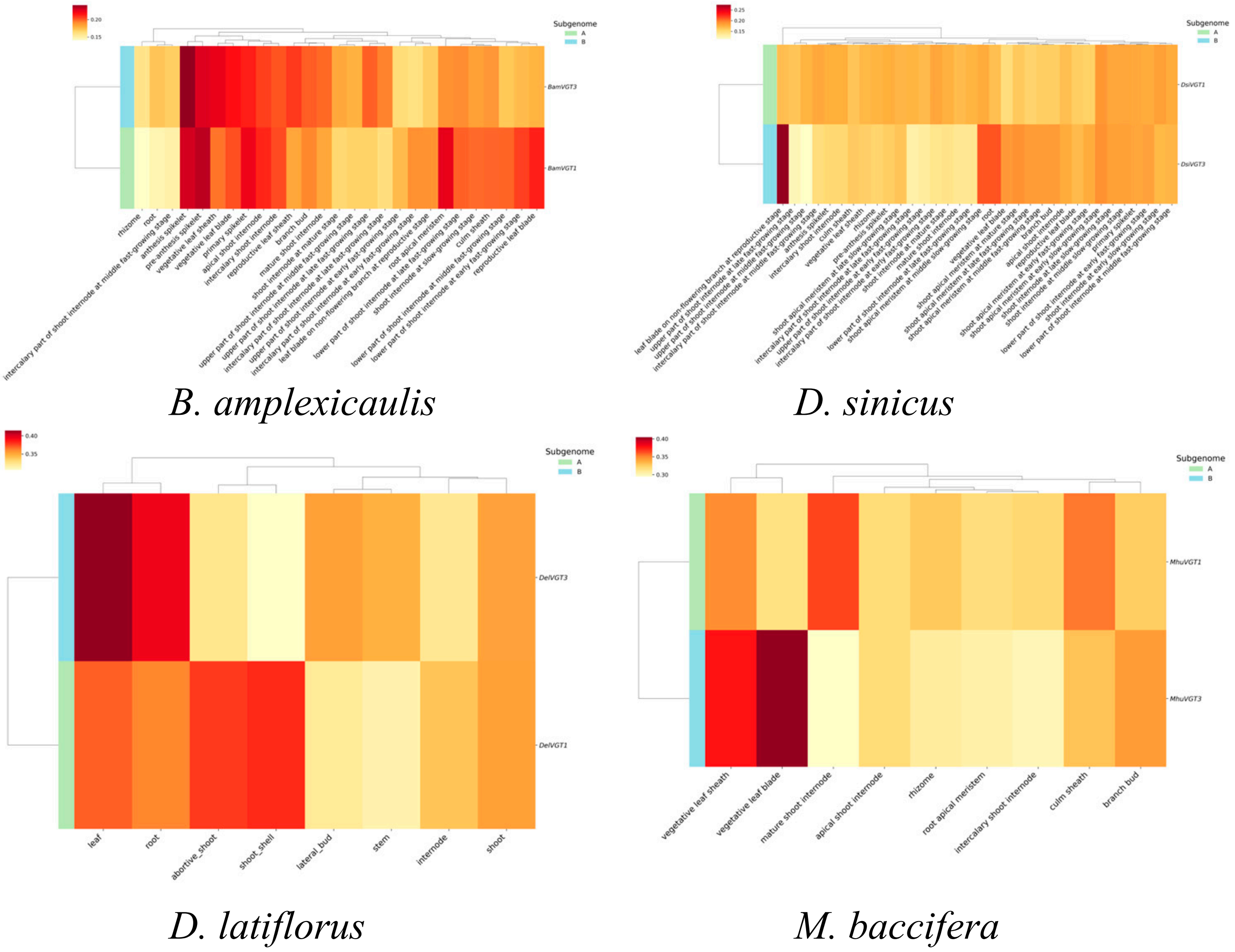

Supplementary Figure S104 Hexaploid paleotropical woody bamboos VGT Transcriptome
